# Cancer cell-selective modulation of mitochondrial respiration and metabolism by potent organogold(iii) dithiocarbamates†

**DOI:** 10.1039/d0sc03628e

**Published:** 2020-09-18

**Authors:** Randall T. Mertens, Sean Parkin, Samuel G. Awuah

**Affiliations:** Department of Chemistry, University of Kentucky Lexington KY 40506 USA awuah@uky.edu; Center for Pharmaceutical Research and Innovation, College of Pharmacy and Department of Pharmaceutical Sciences, College of Pharmacy, University of Kentucky Lexington Kentucky 40536 USA

## Abstract

Metabolic reprogramming is a key cancer hallmark that has led to the therapeutic targeting of glycolysis. However, agents that target dysfunctional mitochondrial respiration for targeted therapy remains underexplored. We report the synthesis and characterization of ten (10) novel, highly potent organometallic gold(iii) complexes supported by dithiocarbamate ligands as selective inhibitors of mitochondrial respiration. The structure of dithiocarbamates employed dictates the biological stability and cellular cytotoxicity. Most of the compounds exhibit 50% inhibitory concentration (IC_50_) in the low-micromolar (0.50–2.9 μM) range when tested in a panel of aggressive cancer types with significant selectivity for cancer cells over normal cells. Consequently, there is great interest in the mechanism of action of gold chemotherapeutics, particularly, considering that DNA is not the major target of most gold complexes. We investigate the mechanism of action of representative complexes, **1a** and **2a** in the recalcitrant triple negative breast cancer (TNBC) cell line, MDA-MB-231. Whole-cell transcriptomics sequencing revealed genes related to three major pathways, namely: cell cycle, organelle fission, and oxidative phosphorylation. **2a** irreversibly and rapidly inhibits maximal respiration in TNBC with no effect on normal epithelial cells, implicating mitochondrial OXPHOS as a potential target. Furthermore, the modulation of cyclin dependent kinases and G1 cell cycle arrest induced by these compounds is promising for the treatment of cancer. This work contributes to the need for mitochondrial respiration modulators in biomedical research and outlines a systematic approach to study the mechanism of action of metal-based agents.

## Abstract 2

Modulating mitochondrial metabolism is a fruitful arena to target metabolic diseases and cancer. Here, we demonstrate that organometallic gold compounds inhibit mitochondrial OXPHOS to selectively kill aggressive TNBC cancer cells.

## Graphical abstract

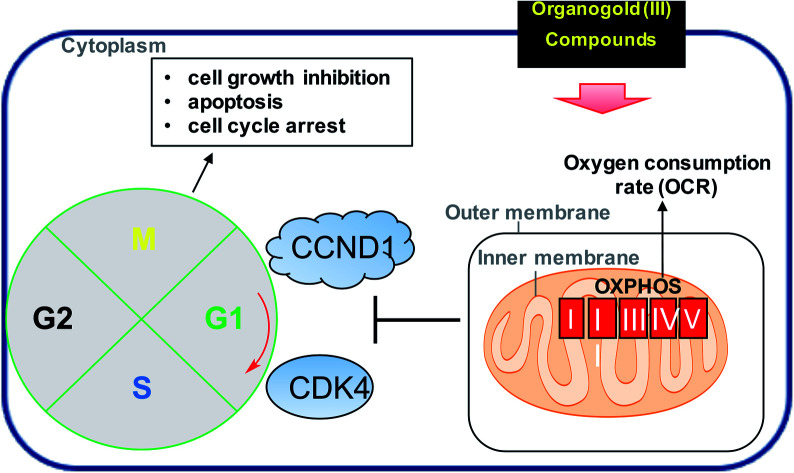

## Introduction

Developing next generation metal-based drugs that exploit the cancer cell vulnerabilities will offer more effective therapies and tools to study the disease. Tumors harbor inherent complexities that present therapeutic challenges and evasion of the physiological machinery to fix the neoplastic disease state.^1^ The biological hallmarks of cancer, which define alterations in cell physiology to promote tumor growth include, (i) insensitivity to growth-inhibitory (antigrowth) signals, (ii) evasion of programmed cell death (apoptosis), (iii) limitless replicative potential, (iv) sustained angiogenesis, (v) tissue invasion & metastasis, (vi) avoiding immune destruction, (vii) reprogramming of energy metabolism^2,3^ with genetic instability and tumor-promoting inflammation as primary drivers. The complexities however present opportunities for targeted therapy. The redox properties of transition metal constructs, can influence metabolic reprogramming of cancer cells^4,5^ given the inherent redox activity associated with bioenergetics and mitochondrial processes such as the electron transport chain (ETC).^6^ Metabolically active tumors are addicted to glycolysis (known as the Warburg effect)^7,8^ and this has been exploited therapeutically through inhibition of glucose metabolism and the use of 2-deoxy-2-fluoro-d-glucose (FDG-PET) to detect tumors.^9,10^ Additionally, many tumor types depend on OXPHOS (oxidative phosphorylation), an electron transfer chain driven by substrate oxidation that is coupled to the synthesis of ATP through an electrochemical transmembrane gradient to coordinate their bioenergetic states and promote proliferation.^11,12^

Over 50% of cancer patients receive platinum agents worldwide.^13^ Platinum are particularly effective in bladder and testicular cancers.^14,15^ Although widely used in other cancer types such as colon and ovarian, their response rate is stymied by recurrence due to acquired and innate mechanisms.^16–18^ The discovery of new transition metal complexes with new biological targets and mechanism of action have become alternate strategies to improve treatment outcomes.^19,20^ Gold (Au) is a third-row transition metal just like platinum which can be tolerated in humans as displayed in the FDA approved drug, auranofin.^21–23^ Both Au(i) and (III) complexes have gained considerable interest over the years as anticancer agents with preferential targeting of mitochondria,^24^ thioredoxin proteasome, and inducing endoplasmic reticulum stress.^24–32^ Gold complexes with multifaceted target mechanisms to evade resistance pathways are attractive.

Renewed interest in Au(iii) as antitumor agents in recent reports since the mid-1970s attest to their high cytotoxicity, reduced cross-resistance with cisplatin, and tolerance in tumor-bearing mice.^27,33–36^ Among such complexes are Au(iii)-2-pyridylmethanol,^37^ Au(iii)dichloro(*N*-ethylsalicylaldiminate),^38^ Au(iii)-phosphine,^39–41^ cyclometalated Au(iii),^27,34,42–46^ and Au(iii)-dithiocarbamates.^45,47–52^ Whereas different classes of Au(iii)-dithiocarbamate display promising anticancer activity *in vitro* and *in vivo* the mechanism of action is not well elucidated.^45,53^

Herein, we report the synthesis of Au(iii) dithiocarbamates that incorporate aryl-pyridine cyclometallation [C^N] ligands to improve complex stability and different aryl/alkyl dithiocarbamate ligands for structure activity relationship (SAR). The compounds display higher potency in a panel of cancer cells than cisplatin. Using detailed HPLC, spectrometry, and cyclic voltammetry we identified the active gold(i) cytotoxic agent. We investigated the mechanism of action using a systems and functional biology approach that established induction of mitochondria membrane depolarization with subsequent modulation of oxidative phosphorylation in cancer cells. Altogether, our studies show that compound **2a** exploits cancer cell vulnerability *via* disruption of mitochondrial respiration. These compounds exhibit significant depolarization of the mitochondrial membrane and as such, ROS production is observed as a byproduct. It is possible that compound **2a** reacts with a nucleophilic side chain of its protein target within the oxidative phosphorylation machinery.

## Results and discussion

### Synthesis and characterization

**Scheme 1:**
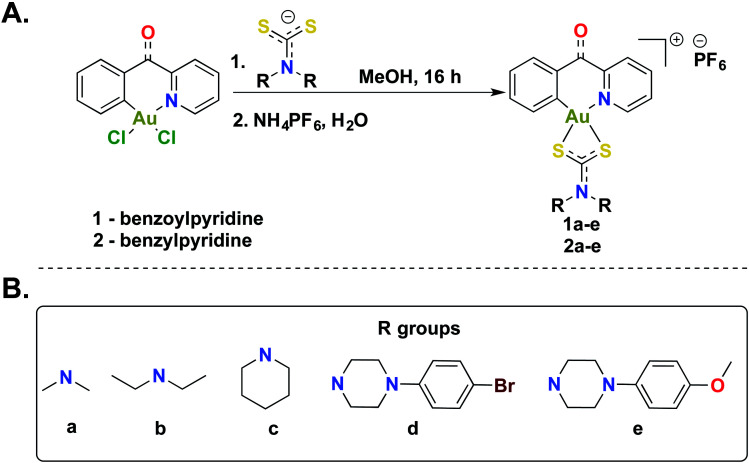
Synthesis of gold(iii) dithiocarbamates. (A) Synthetic scheme to generate cyclometalated (C^N) gold(iii) complexes bearing dithiocarbamate ligands. (B) Diversity of dithiocarbamate ligands used in this study.

The [C^N]-cyclometalated gold(iii) compounds were synthesized from previously reported methods.^54^ Treatment of [C^N]-Au(iii)Cl_2_ with dithiocarbamate ligands in methanol for 16 h at room temperature followed by treatment with an aqueous saturated solution of NH_4_PF_6_ gave the desired compounds in respectable yields (Scheme 1). We characterized all 10 compounds (**1a–e** and **2a–e**) by ^1^H-NMR, ^13^C-NMR, and ^19^F-NMR and high-resolution mass spectrometry (HRMS) (Fig. S1–S46†). The purity of the compounds was verified by HPLC (>95%) (Fig. S47–56†). In this study, we sought to expand the structural architecture of Au(iii) dithiocarbamate complexes to include cyclic and aromatic side chains in addition to cyclometalation, which provide stabilization by strong σ-donation to the gold center.^55,56^ We and others have demonstrated that this stabilization strategy has positive implications on the solution chemistry, biological and electrochemical behavior of complexes.^57^ Two different [C^N]Au(iii)Cl_2_ complexes with electronic variations were used in the synthesis with the aim of establishing structural diversity. Complexes **1a–e** possess a carbonyl at the methine bridge of the benzylpyridine framework, whereas **2a–e** does not. Studies from our laboratory show that the difference impart unique reactivity and kinetics towards nucleophiles. Alternate metal-based drugs to platinum, the first-line of chemotherapy for several cancer types are desperately needed to overcome the toxicity and resistance associated with platinum drugs.^58–62^ Gold compounds including auranofin have been used in preclinical and clinical trials with great promise.^63–67^ A more systematic design and the elucidation of the mechanism of action of gold compounds will expand the utility of well-defined gold anticancer drug candidates with reduced susceptibility to resistance and toxic side effects. In this work, we developed a small library of gold(iii) compounds supported by different cyclometalated and dithiocarbamate ligands to obtain cationic complexes complemented by hexafluorophosphate ions. Structural diversity was achieved by using two different cyclometalated gold(iii) starting materials and five dithiocarbamate ligands of distinct structural and electronic variety. Consequently, the library enabled us to explore the biological activity of novel gold(iii) dithiocarbamates in the context of cancer as described in this report.

### Single crystal X-ray diffraction

**Fig. 1:**
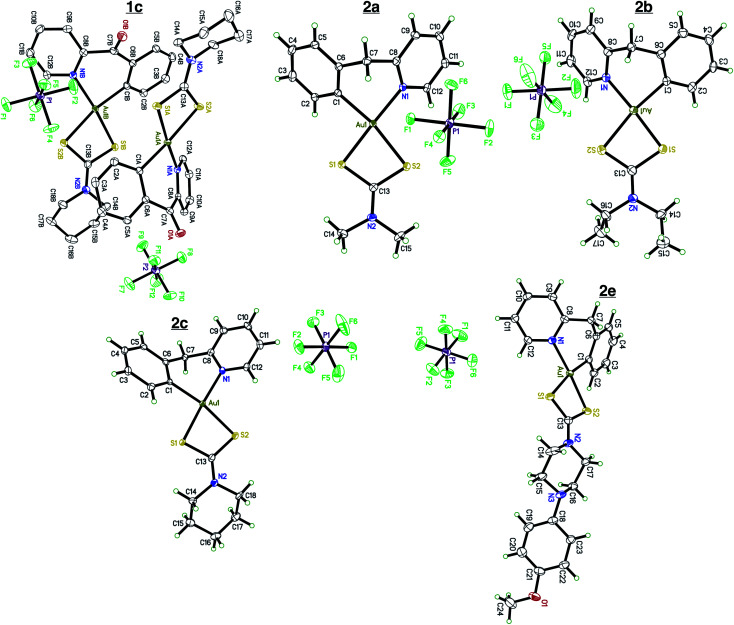
X-ray crystallography. Single crystal X-ray diffraction of complexes **1c**, **2a**, **2b**, **2c**, and **2e**. Complex **1c** contains two cation/anion pairs in the asymmetric unit. Complex **2e** crystalized out in the solid state with a molecule of Et_2_O. All atoms are drawn at the 50% probability level. Solvents were omitted for clarity. Full crystallographic parameters can be found in the ESI Tables S1–S5.†

Single crystals of compounds **1c**, **2a**, **2b**, **2c**, and **2e** (Fig. 1) were grown by slow diffusion of Et_2_O into a concentrated MeCN solution at room temperature.

Compounds **2a**, **2b**, **2c**, and **2e** crystallize out in the solid state with one molecule per asymmetric unit while compound **1c** contains two cation/anions pair. Each cyclometalated ligand, 2-benzylpyridine and 2-benzoylpyridine have a slightly puckered twist. The bridging methine carbon allows for the formation of a 6-membered ring containing the Au atom, leading to a less strained cyclometalated system. Each molecule is distorted around the Au–C and Au–N bonds resulting in half occupancy among the two atoms. Analyzing the bonding motif of the [C^N] framework reveals a significant *trans*-effect on the binding dithiocarbamate ligand. The Au–S bond *trans* to the nitrogen of the [C^N] ligand is significantly shorter (on average 0.156 Å) than the Au–S bond *trans* to the carbon (Table 1). This results in a slightly distorted square planar geometry around the Au(iii) center in comparison to other Au(iii) bis-dithiocarbamate complexes bearing symmetrical dithiocarbamate ligands which contain four Au–S bonds equidistant to one another (∼2.33 Å).^47,68^

**Table 1:** Selected interatomic distances. Bond distances of complexes **1c**, **2a**, **2b**, **2c**, and **2e**. Bond lengths were obtained from X-ray crystallography dataset

Bond (Å)	Compound				
	**1c**	**2a**	**2b**	**2c**	**2e**
Au–C	2.042 (19)	2.043 (3)	2.061 (5)	2.042 (3)	2.045 (19)
Au–N	2.062 (18)	2.071 (2)	2.065 (5)	2.065 (3)	2.069 (17)
Au–S_(*trans* to N)_	2.283 (8)	2.280 (7)	2.278 (16)	2.276 (9)	2.298 (5)
Au–S_(*trans* to C)_	2.376 (9)	2.404 (8)	2.392 (14)	2.499 (8)	2.379 (5)

### Photophysical and solution chemistry

**Fig. 2:**
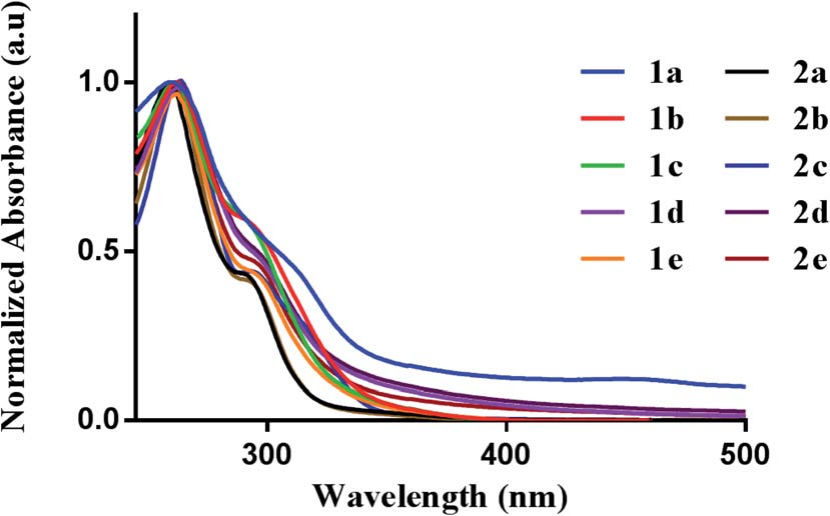
Photophysical behavior. UV-vis spectra of **1a–e** (50 μM) and **2a–e** (50 μM) in DMSO.

We first evaluated the photophysical properties of all ten complexes by scanning their absorption profile in DMSO (Fig. 2). A stock solution of each complex was prepared and diluted to a final concentration of (50 μM). The UV-vis profile was then obtained by scanning from 600 to 200 nm. Each complex displayed similar absorption profiles with a high-energy transition at 260 nm and a corresponding lower energy transition at 295–305 nm. The high-energy transition at 260 nm can be attributed to intraligand transitions of the 2-benzylpyridine or 2-benzoylpyridine fragments in the [C^N] ligands. The low-energy transition is attributed to ligand-to-metal-charge transfer from the donor dithiocarbamate ligand to the electrophilic Au(iii) metal center.^39^ The different dithiocarbamate ligands utilized had minimal effect on the measured absorbance.

**Fig. 3:**
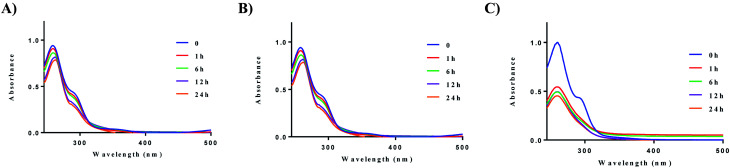
Solution chemistry of **2a**. (A) Stability of **2a** (50 μM) in PBS over 24 h, (B) stability of **2a** (50 μM) in DMEM over 24 h, (C) stability of **2a** (50 μM) in RPMI-1640 over 24 h. Experiments were conducted at 37 °C.

We next evaluated the stability of all ten complexes in three relevant biological media, namely, PBS (phosphate-buffered saline), DMEM (Dulbecco's modified eagle medium), and RPMI-1640 (Roswell Park Memorial Institute-1640). RPMI-1640 contain several biological nucleophiles such as amino acids and extracellular relevant levels of GSH, which are common sources of reductants. Development of new drug molecules require proper characterization of compound stability in solution. To evaluate their stability, the complexes were prepared as a stock solution in DMSO (5 mM) and diluted with PBS or DMEM at room temperature to a final concentration of 50 μM. Upon addition of the stock solution to the aqueous based medium, no precipitation was observed. Complex **2a** displayed higher stability in PBS, and DMEM at 37 °C in comparison to RPMI-1640 (Fig. 3). The solution stability of the other 8 complexes, **1b–e** and **2b–e** can be found in the ESI (Fig. S57–S64† (PBS), Fig. S65–S72† (DMEM), and Fig. S73–S83† (RPMI-1640)).

After 24 h, no significant decrease in absorbance was observed for compounds **1a** and **2a**. The cyclic dithiocarbamate ligands used, especially complexes bearing the phenyl-substituted piperazine moiety demonstrated reduced stability in comparison to the alkyl substituted dithiocarbamates over 24 h. Although no deposition of elemental gold was observed, UV-vis analysis showed a significant decrease in absorption. We attribute the reduced stability of these complexes to the longer Au–S bond distances (2.298 Å (5)) trans to the aromatic nitrogen of the cyclometalated ligand, indicative of a weaker bond. This in addition to the other electronic factors promote facile reactivity with nucleophiles, hence the instability. Also, X-ray crystallography reveals the (4-methoxyphenyl)piperazine dithiocarbamate as an elongated moiety with a puckered square planar geometry. Elongation of the ligand likely weakens the Au–S bonds, which reduces stability to nucleophiles in the medium used. Overall, alkyl substituted gold dithiocarbamates, **1a** and **2a** show stability in biologically relevant media over extended periods of time and are promising candidates for further studies.

### Reactivity of gold(iii) dithiocarbamates with cysteine thiols

**Fig. 4:**
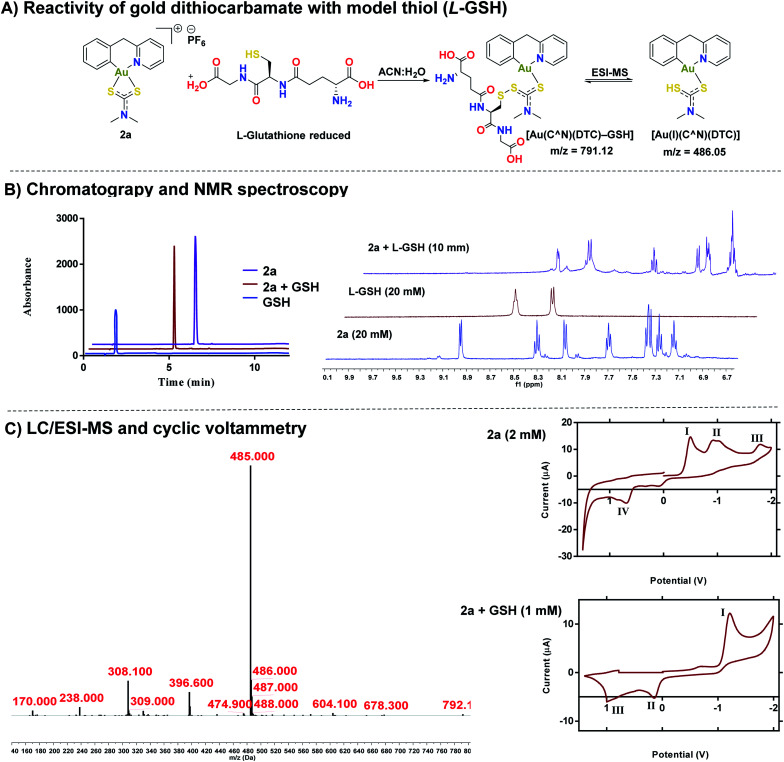
Elucidation of the Au(iii) – thiol adduct. (A) Proposed reaction scheme of compound **2a** and GSH in an equimolar ratio, (B) HPLC chromatograms of **2a**, GSH, and the reaction of **2a** + GSH adduct (*λ* = 240 nm) (see ESI† for HPLC method and sample preparation) with ^1^H NMR of the same mixture in DMSO (see ESI† for full spectra), (C) LC-MS of the **2a** + GSH adduct (*λ* = 280 nm) (see ESI† for HPLC method and sample preparation), cyclic voltammograms of **2a** (I = Au(i) to Au(0) reduction,^70^ II = disproportionation of a one electron reduction from Au(iii)/Au(ii) and Au(ii)/Au(i),^73,74^ III = Au(iii) to Au(i) reduction,^46,73,74^ IV = oxidation of the dithiocarbamate ligand)and **2a + GSH**(I = Au(i) to Au(0), II = oxidation of the dithiocarbamate ligand, III = oxidation of GSH).

After establishing the stability of complexes, **1a** or **2a** in biological buffer, DMEM, and RPMI-1640 solutions, we investigated the reactivity of **2a** with cysteine thiols using l-glutathione (GSH) and l-*N*-acetyl cysteine as a models (Fig. 4). Reactivity of **1a–1e** and **2b–2e** with both GSH and NAC can be found in the ESI (Fig. S84–S103†). Whereas gold dithiocarbamates are known to react with thiols,^45,49,51,69^ detailed studies to unravel the potential mechanism of activity do not exist. HPLC trace of the reaction solution revealed one distinct band at a different retention time (*R*_t_ – 5.00 minutes) from **2a** (*R*_t_ – 6.03 minutes) or GSH (*R*_t_ – 1.72 minutes) (Fig. 4B). We subjected the reaction solution of **2a** with GSH to LC-MS analysis (Fig. 4C), which supported the formation of a new species with a mass peak at *m*/*z* 791, attributable to a gold(i) disulfide species, [Au(C^N)(DTC)–GSH] (Fig. 4C), which is short-lived under ESI-MS conditions due to the labile disulfide bond formed between the thiol of *L*-GSH and the dithiocarbamate ligand of the gold complex (Fig. 4A). Thus, a more dominant mass peak at *m*/*z* 485, attributable to an intact gold complex with liberated *L*-GSH is observed. ^1^H NMR spectroscopy revealed the farthest downfield-shifted peak (*δ* = 9.03 ppm), which is the hydrogen located next to the nitrogen in the cyclometalated-ligand, was significantly shifted upfield in the adduct solution (Fig. 4B and S105†). We attribute this to the loss of coordination from the nitrogen of the cyclometalated ligand to gold, a key signature of the proposed adduct. We then confirmed the formation of the gold(i) complex by cyclic voltammetry (Fig. 4C), displaying a reduction potential of −1.20 V that is consistent with a gold(i) species.^70–72^ We evaluated the effect of the **2a-GSH** adduct on breast cancer cells and found similar toxicity (IC_50_ – 0.53 μM (Fig. S110† as reported in Table 2 below. Taken together, we have further elucidated the reactivity of [C^N] gold(iii) dithiocarbamates with representative thiols. Interestingly, the reduced gold(i) compound display high anticancer activity.

**Table 2:** *In vitro* antiproliferative activity. IC_50_ values for **1a–e** and **2a–e** across a panel of cell lines. Cells were seeded at a density of 2000 cells per well and treated for 72 h. IC_50_ values are plotted as the mean ± s.e.m (*n* = 3). Full dose response curves can be found in the ESI

	IC_50_ (μM), 72 h				
	MDA-MB-175	MDA-MB-231	A2780	RPE-NEO	MRC5
**1a**	0.926 ± 0.10	0.531 ± 0.103	0.521 ± 0.081	10.29 ± 2.61	>50
**1b**	1.26 ± 0.307	0.935 ± 0.16	0.924 ± 0.187	25.4 ± 1.98	>50
**1c**	1.85 ± 0.194	0.951 ± 0.135	1.19 ± 0.126	12.11 ± 1.59	>50
**1d**	0.842 ± 0.671	1.14 ± 0.08	0.816 ± 0.123	15.13 ± 2.19	>50
**1e**	1.12 ± 0.431	1.36 ± 0.04	1.13 ± 0.065	14.8 ± 2.15	>50
**2a**	0.618 ± 0.080	0.773 ± 0.117	0.741 ± 0.086	17.1 ± 1.86	>50
**2b**	0.831 ± 0.099	2.11 ± 0.100	0.820 ± 0.073	15.1 ± 1.86	>50
**2c**	1.031 ± 0.091	1.04 ± 0.101	1.20 ± 0.33	21.1 ± 18.6	>50
**2d**	0.937 ± 0.399	0.849 ± 0.067	2.962 ± 0.71	14.5 ± 2.45	>50
**2e**	1.17 ± 0.487	1.31 ± 0.088	0.811 ± 0.892	19.2 ± 2.81	>50

### Cellular uptake

**Fig. 5:**
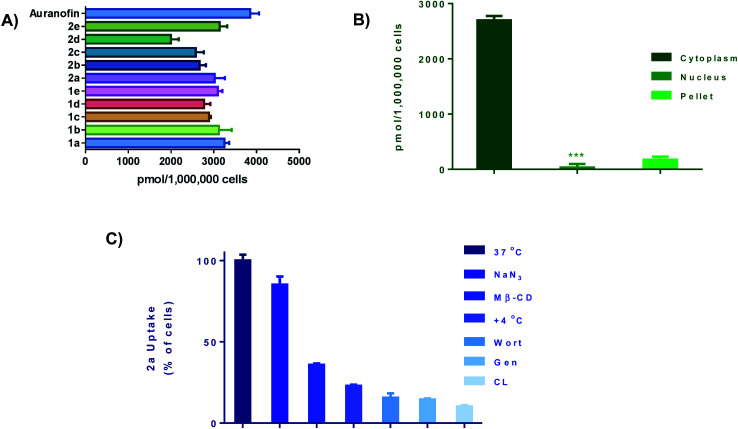
Cellular uptake and uptake mechanism. (A) Intracellular uptake of **1a–e** and **2a–e** as well as Auranofin. Cells were seeded at a density of 1 × 10^6^ and incubated overnight. Cells were incubated with gold drug or compounds for 24 h (5 μM), data is plotted as the mean ± s.e.m (*n* = 3), (B) cell fraction uptake of **2a** at 5 μM for 24 hours, data are plotted as the mean ± s.e.m (*n* = 3), ***denotes value lower than detection limit of the instrument (5 μg L^−1^), (C) whole cell uptake of **2a** (5 μM for 24 h) against pre-treatment with uptake inhibitors, data are plotted as the mean ± s.e.m (*n* = 3). Calibration curves can be found in Fig. S133–S135.†

We next performed cellular uptake studies to determine what percentage of the compounds were getting into the cell after treatment. Given that most therapeutic targets are localized receptors on the plasma membrane, quantifying the amount of gold compounds in cellular fractions and investigating their mode of uptake is crucial. For whole-cell uptake studies, MDA-MB-231 were treated with all 10 compounds and Auranofin at 5 μM for 24 h (Fig. 5A). Auranofin is the only gold-based therapeutic to be clinically approved, therefor we used this drug as a benchmark for comparison.^67^ After treatment, the cells were washed with PBS, centrifuged, and digested with 0.5 mL of conc. HNO_3_ and diluted with DI water and subjected to ICP-OES analysis. All ten compounds exhibited intracellular uptake above 2000 pmol per million cells. In comparison to Auranofin, none of the complexes exhibited higher uptake, however; the complexes **1a** and **2a** had uptakes (3017 pmol per million cells) and (3267 pmol per million cells) respectively. Complexes **1c–e** and **2c–e**, which contain more hydrophobic R groups such as the piperadine and phenyl substituted piperazine handles, exhibited lower intracellular uptake than the complexes with the less hydrophobic shorter alkyl (methyl and ethyl) handles. Further evaluation revealed that >90% of the Au content was found localized in the cytoplasm (Fig. 5B) and total membrane fraction (pellet). Given that these complexes readily react with nucleophilic biological molecules and the lack of accumulation in the nuclear fraction, it is evident that the mechanism of action of this class of compounds is differentiated from the clinically available platinum agents.

To understand cell uptake mechanisms, detailed uptake studies using GF-AAS were conducted whereby MDA-MB-231 cells were incubated with gold compound, **2a** after cellular pretreatment with one of a number of known inhibitors of cellular uptake. Sodium azide (NaN_3_), which is known to inhibit mitochondrial oxidative phosphorylation, was used as a general inhibitor of energy (ATP)-dependent (active) uptake; chlorpromazine (CL) was utilized as an inhibitor of clathrin-dependent endocytosis (CDE); β-cyclodextrin (Me-β-CD) and genestein (Gen) were used as inhibitors of clathrin-independent endocytosis (CIE); and, wortmannin (Wort) was employed as a known inhibitor of micropinocytosis. We found that the uptake of **2a** was significantly decreased with all inhibitors with the exception of NaN_3_ (Fig. 5C). Being an ATP-dependent pathway illustrates that these compounds are not dependent on oxidative phosphorylation for uptake, despite; their ability to modulate OXPHOS. Overall, these ten Au(iii) complexes demonstrate relatively high intracellular uptake which can explain the high *in vitro* cytotoxicity as well as the rate at which cellular bioenergetics are affected.

### Anticancer activity *in vitro*

All the gold(iii) dithiocarbamate compounds display remarkable cell killing potential in a panel of breast, ovarian, lung and leukemia cancers. Preliminary studies focused on different cancer cell lines (MDA-MB-231, MDA-MB-175, and A2780), an immortalized normal retinal epithelial cell line (RPE-NEO) and human fetal lung fibroblasts (MRC5) (Fig. S111–S129†). These adherent cells were exposed to a serial-diluted concentration of all ten compounds for 72 h. The cells were subsequently treated with 3-(4,5-dimethylthiazol-2-yl)-2,5-diphenyltetrazolium bromide (MTT) solutions, dissolved with DMSO and the absorbance at 570 nm was measured with a plate-reader. Our initial screen showed promising half-maximal inhibitory concentration (IC_50_) values for all complexes across the three cancer cell lines (Table 2). Comparison of IC_50_ values with uptake revealed no significant correlation.

**Fig. 6:**
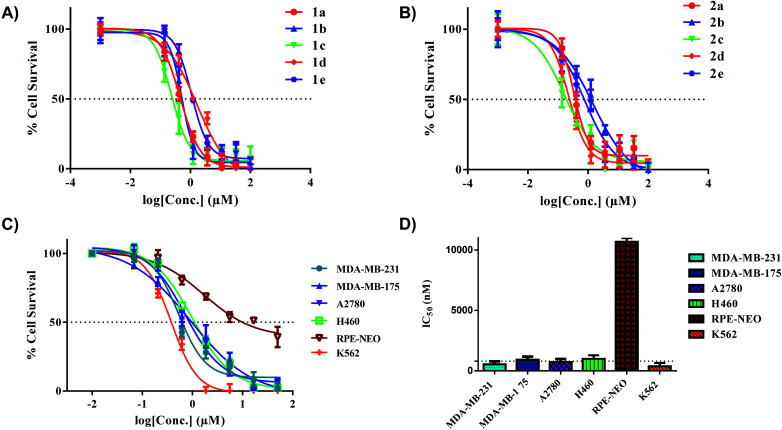
*In vitro* activity of complexes **1a–e** and **2a–e** in multiple cancer cell lines. (A) IC_50_ plot for complexes **1a–e** in MDA-MB-231 (72 hours), (B) IC_50_ plot for complexes **2a–e** in MDA-MB-231 (72 hours), (C) extended panel of cell lines for complex **2a**, (D) representative bar graph illustrating the IC_50_ values of **2a** across a panel of cancer cell lines. The dotted line indicates a threshold of 1 μM. % Cell survival was determined with MTT (adherent cells) and CellTiter-Glo (suspended cells). Data are plotted as the mean ± s.e.m (*n* = 3).

Specifically, the complexes exhibited high toxicity (0.5–1.5 μM) towards the TNBC cell line MDA-MB-231 (Fig. 6A and B). These complexes collectively are more cytotoxic towards the cell line MDA-MB-231 in comparison to the [C^N]Au(iii)DTC complexes prepared by Bochmann *et al.*^45^ The different [C^N] backbone presented here proves to be a critical component in drug design and overall improvement of cytotoxic efficacy. The cytotoxicity of complexes **1a** and **2a** towards TNBC's is quite promising as these cell lines are typically cisplatin resistant.^75,76^ Drug resistance is an ever-increasing problem in medicine so developing therapeutics for refractory tumours is of great importance.^77^ To test the selectivity of compounds **1a** and **2a** for cancer cells over normal cells, we evaluated these complexes in the normal retinal epithelial cells using the MTT assay. We found up to ∼30-fold selectivity for cancer cells over normal cells, indicative of compounds with potential for reduced side effects.

With the completion of preliminary cytotoxicity screening, we set out to explore the efficacy of compound **2a** in different cancer tissue type beyond breast cancer. Both H460 (human large cell lung carcinoma) and K562 (human chronic myelogenous leukemia) were utilized (Fig. 6C and D). We found comparable cytotoxicity in these cell lines to those in our preliminary screen. With **2a** displaying IC_50_ values of 1.5 μM in H460 and 1.0 μM in K562. With a promising candidate in hand, we pursued further biological testing to gain mechanistic insight of this class of Au(iii) dithiocarbamates.

### Differential gene expression and biological pathway analysis

**Fig. 7:**
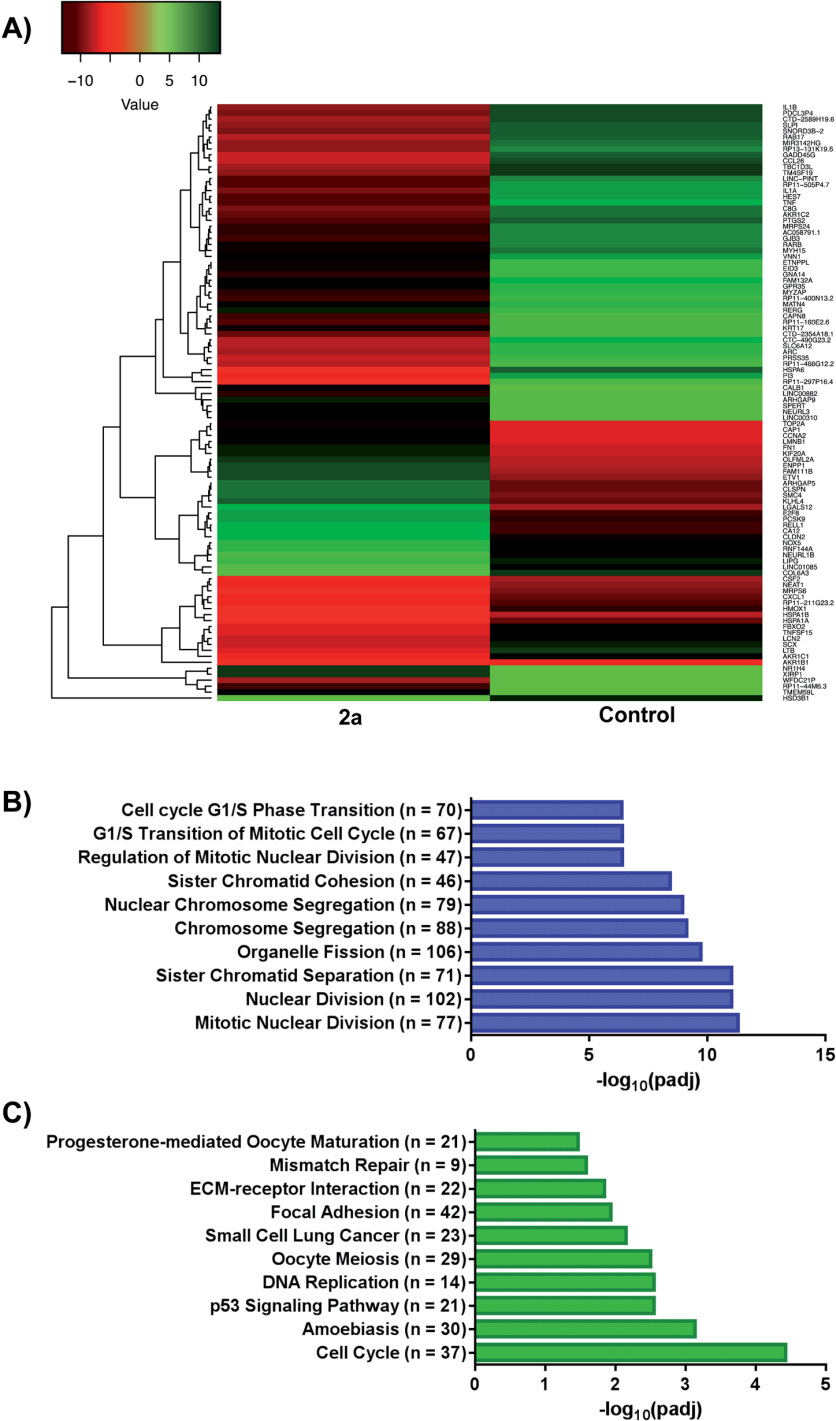
RNA-sequencing and pathway analysis. (A) Representative heat map of differentially expressed genes (DEGs) in response to **2a**, (B and C) gene ontology (GO) and Kyoto Encyclopaedia of Genes and Genomes (KEGG) plots outlining varying pathways perturbed upon treatment with **2a**. For GO; GeneRatio = *n*/2005 and = BgRatio *n*/17107. For KEGG; GeneRatio = *n*/727 and BgRatio = *n*/5824.

We investigated the whole-cell effect of compound **2a** by analysing differentially expressed genes (DEG) from RNA-seq. We treated MDA-MB-231 cells with 1 μM of **2a** for 12 h followed by the isolation of high-purity RNA for Illumina Hi-seq. We found 3019 DEG with 1596 upregulated and 1423 downregulated genes in response to **2a** (Fig. 7A). Subsequent use of gene ontology (GO)^78,79^ (Fig. 7B) and Kyoto encyclopaedia of genes and genomes (KEGG)^80–82^ (Fig. 7C) pathway analysis software led to potential processes perturbed by **2a**. The pathway analysis software employed are an extensive library database capable of integrating chemical and biological pathway perturbation processes and is well suited for drug development studies. For the identified processes, we examined the corresponding downstream canonical pathways and corroborated activated or inhibited pathways with functional biology experiments (*vide infra*). Notable pathways identified include, mitotic nuclear division, organelle fission, cell-cycle, progesterone oocyte maturation, and focal adhesion processes. Interestingly, a significant number of these processes are regulated by the mitochondria. It is well established that a number of aggressive tumor types including TNBCs exhibit impaired mitochondria function, which present a vulnerability.^83,84^ We therefore, hypothesized that this class of gold(iii) dithiocarbamates disrupt mitochondria function in MDA-MB-231 cells. This was tested by detailed mitochondrial investigations.

### Gold(iii) dithiocarbamate disrupts TNBC cell metabolism

**Fig. 8:**
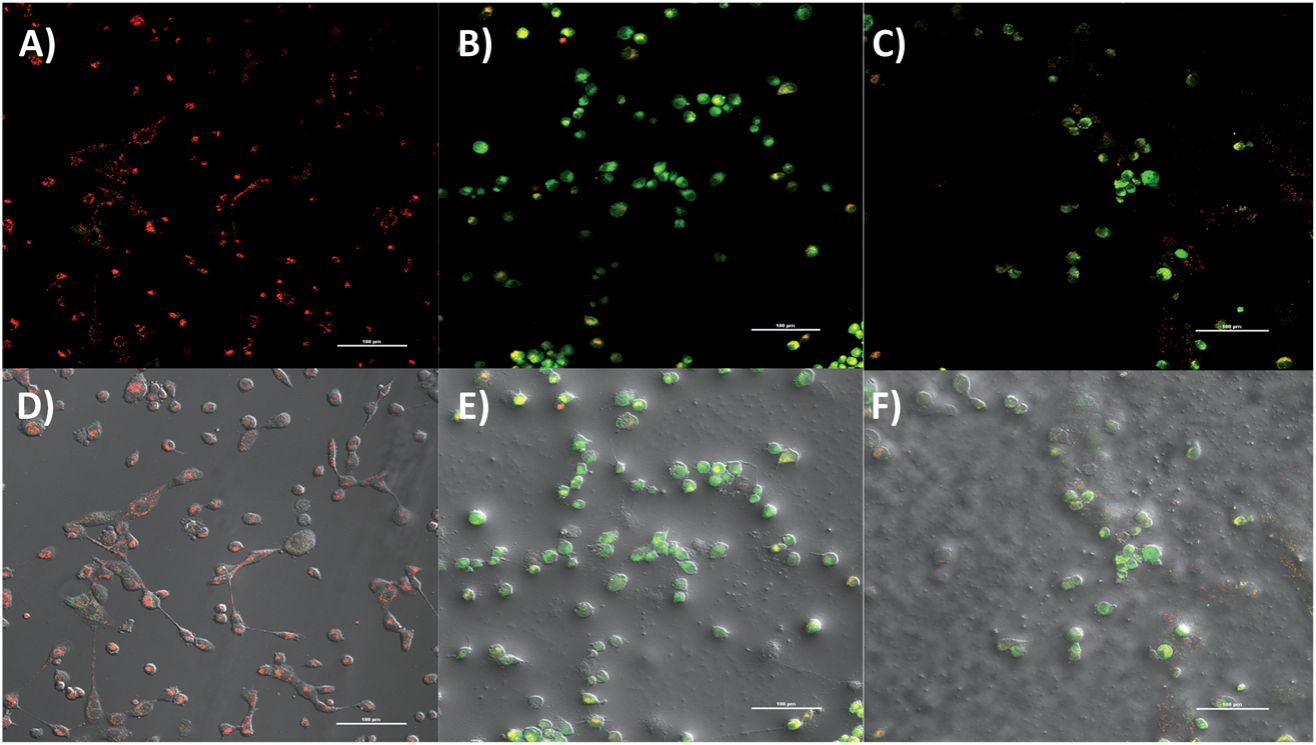
Mitochondrial membrane potential of **2a** in MDA-MB-231 cells. (A) DMSO vehicle, (B) **2a** at 10 μM for 6 h, (C) CCCP as a positive control at 100 μM for 30 minutes, (D–F) corresponding bright field image: DMSO vehicle, **2a**, and CCCP. Compound **2a** significantly depolarizes the mitochondria membrane upon treatment within 6 h. MDA-MB-231 cells were seeded at a density of 5 × 10^5^ cells per glass bottom dish with a #1.5 coverslip. J-Monomers shown as green fluorescence (exc. 488 nm) and J-aggregates shown as red fluorescence (exc. 520 nm). Images are representative of three independent treatments.

The RNA-seq showed transcriptional suppression of key genes involved in the catalytic conversion of long-chain fatty acids such as ACSL4. Further analysis of DEG revealed the modulation of several genes within the mitochondria respiratory chain or oxidative phosphorylation. Activated DEG included *NDUFS7*, [complex I], COX7A1 gene [complex IV], and ATP5O, ATP5I genes [complex V] whereas the inhibited DEG were *MT-ND4L* [complex I], *SDHD* gene [complex II], and *ATP5C*, *ATP5F* [complex V]. Moreover, an uncoupling protein related gene, *UCP3* was found to be upregulated in response to **2a**. These findings prompted functional biology experiments to further corroborate the effect of **2a** or its mechanism of action in TNBC. We examined the effect of **2a** on mitochondrial membrane potential, assayed by JC-1 dye.^85^ Following an extended **2a** treatment and JC-1 staining (Fig. 8), we found that **2a** exhibits strong depolarization of the MMP (J-monomers) in comparison to the DMSO treatment (J-aggregates) in MDA-MB-231 cells. We used carbonyl cyanide *m*-chlorophenyl hydrazine (CCCP), an uncoupler, as a positive control to validate the experiment. The MMP is a key driving force in ATP synthesis and overall a key factor in maintaining redox homeostasis throughout the cell.^86^ Uncoupling of the mitochondrial membrane results in acute cell death as the mitochondrial become dysfunctional in this aggregative state.

### Bioenergetics

**Fig. 9:**
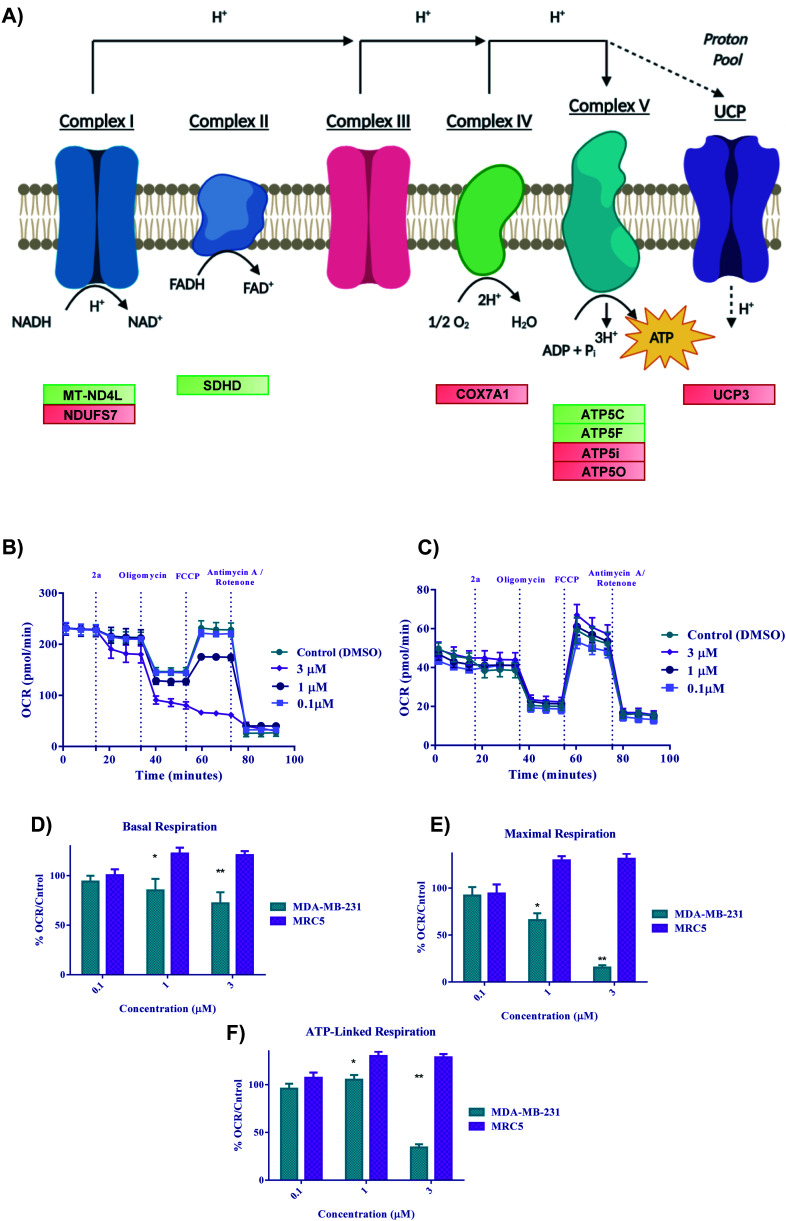
Bioenergetics of **2a** in cells. (A) Schematic illustration of the electron transport chain and corresponding DEGs for **2a**, (B–F) Bioenergetic monitoring of MDA-MB-231 (green bars) and MRC5 (purple bars) cells following acute *in vitro* treatment with complex **2a**. MDA-MD-231 were seeded at a density of 3 × 10^4^ and MRC5 at a density of 5 × 10^4^ and analyzed with a Seahorse XF96 analyzer to assess the effect on key bioenergetic parameters. (B and C) Mitochondria stress test was performed with pneumatic injections of compound **2a** at concentrations ranging from (0.1–3 μM) and response to injections of oligomycin, FCCP, and antimycin A/rotenone. Data is plotted as the mean ± s.e.m (8 technical replicates). (D–F) Key bioenergetic parameters have significant statistical response to treatment with compound **2a** at concentrations as low as 3 μM within 30 minutes in MDA-MB-231 with selectivity over MRC5 cells. Data are plotted as the mean ± s.e.m, *p < 0.05, **p < 0.01.

With the previous biological data pointing towards metabolic changes, we sought to explore further the effect **2a** had on redox metabolism. The ETC is a complicated biological system that is constantly changing in response to external stress in order to achieve redox homeostasis within the cell. With the DEG data alluding to involvement of ETC genes (Fig. 9A), we then decided to look in depth at the effect of complexes on these biological parameters. We next performed oxygen consumption rate (OCR) experiments by Seahorse 96XF to quantify the effect of **2a** on mitochondrial bioenergetics or stress (MitoStress) (Fig. 9B and C).^87^ A series of known inhibitors of the various parts of the ETC that allow for the measurement of specific parameters were used. We conducted a pneumatic injection of **2a** into wells containing adhered MDA-MB-231 cells followed by subsequent injections of oligomycin, a complex V inhibitor, to view the basal OCR; FCCP, an uncoupler used to observe the maximum OCR, and rotenone/antimycin A, a complex I/III inhibitor to completely shut down the ETC. The first step was to determine the optimal MDA-MB-231 cell density and FCCP concentration, which was 30 000 cells per well and (0.6 μM) FCCP (ESI†) respectively. At a concentration of 3 μM, the basal OCR is diminished by more than 20% after only 17 minutes from time of injection of **2a** (Fig. 9D). We found that at a low concentration of **2a** (1 μM), maximal OCR (Fig. 9E) is diminished by more than 25% within 50 minutes of treatment compared to the control. The overall decrease in maximal OCR at 1 and 3 μM implies acute depletion of mitochondrial respiration. ATP linked respiration was also calculated and found to be decreased by more than 80% at 3 μM treatment (Fig. 9F). Overall, the rapid decline in OCR suggests that these complexes are severely impacting the ETC and subsequently OXPHOS, thus causing cell death. Despite the acute dose-dependent OCR depletion induced by **2a** in MDA-MB-231 cancer cells (Fig. 9B), **2a** did not impact the OCR of human fetal lung fibroblast cells, MRC5 (Fig. 9C). These data indicate that **2a** causes rapid irreversible inhibition of OXPHOS in the TNBC, MDA-MB-231 but not in the normal lung epithelial fibroblast, MRC5.

### Apoptosis evaluation

To further assess the mechanistic pathway upon treatment with **2a**, we analysed the apoptotic effect in MDA-MB-231.

**Fig. 10:**
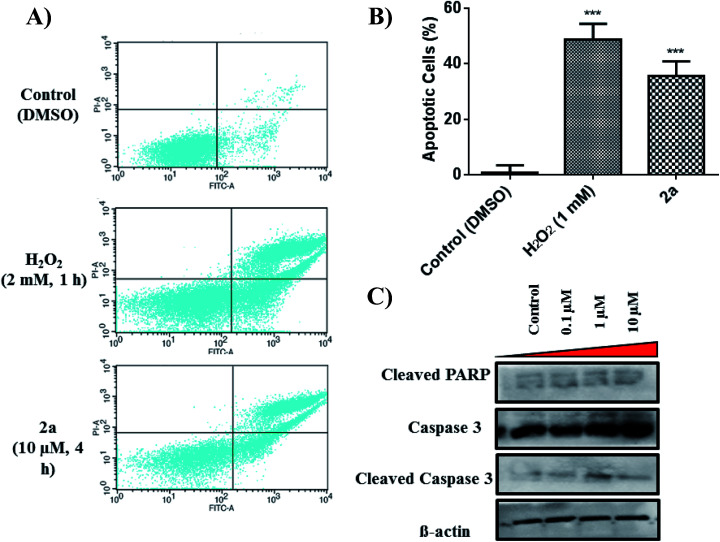
Effect of **2a** on apoptosis. (A) Quadrants displaying apoptotic population of MDA-MB-231 within 4 h of treatment with **2a**. Cells were seeded at a density of 5 × 10^5^ per well. Data is representative of three individual experiments. H_2_O_2_ was used a positive control, (B) bar graph illustrating the early-stage apoptotic cell population. Data are plotted as the mean ± s.e.m (*n* = 3), ***p < 0.001. (C) Immunoblots of MDA-MB-231 treated in a concentration dependant manner.

Apoptosis is a common cell death pathway for chemotherapeutics which can be characterized by distinct morphological features and biochemical mechanisms.^88,89^ Apoptosis occurs normally in healthy cells to maintain a healthy population of cells during aging or development of tissues. Some transition metal-based drugs can trigger apoptosis due to inhibition of p53 dependant pathway (a tumour suppressor gene).^90,91^ Populations of apoptotic cells can be determined by containing cells with Annexin V and PI.^92,93^ Cells undergoing apoptosis contain ample amount of phosphatidylserine (PS) which can be bound by Annexin V. The Annexin is then labelled with FITC, a green fluorogenic dye which can be visualized by fluorescence activated cell sorting (FACS). PI is used to stain damaged DNA to distinguish apoptotic from necrotic cells. Such staining gives four separate quadrants upon analysis; (i) lower left, healthy cells which are negative for both markers, (ii) lower right, pre-apoptotic cells which are positive for FITC but not for PI, (iii) upper right, apoptotic cells which are positive for both markers, (iv) upper left, necrotic cells which are positive for only PI. Fig. 10A illustrates that **2a** at 10 μM induced significant apoptosis of MDA-MB-231 cells. Experimental data shows a 36% increase (extrapolated from Fig. 10B) in apoptotic cells in comparison to the control in just 4 h, indicative of apoptosis as a possible mode of cell death. It is possible that **2a** triggers either caspase dependant- or caspase independent-apoptosis.^94,95^ Immunoblotting was used to assess the effect of **2a** on proteins involved in caspase related apoptosis. In a concentration dependant manner, both caspase 3 and cleaved caspase 3 were found to be upregulated in comparison to the control (Fig. 10C). Upregulation of these proteins is indicative of a caspase dependant apoptotic pathway. Caspase 3 is considered to be an executioner caspase in apoptosis as it coordinates the destruction of cellular structures including DNA fragmentation and degradation of the cytoskeleton.^96,97^ Immunoblotting of cleaved PARP in response to **2a** showed unaltered protein expression, which is likely due to an uninvolved role of PARP DNA damage response.^98–102^

### ROS analysis

We examined the status of intracellular ROS induced by **2a**. For this experiment, we employed the used 2′,7′-dichlorofluoresceindiacetate (DCF-DA),^103^ a fluorogenic dye with an excitation/emission wavelength of 495/525 nm. DCF-DA enters into the cell and is subsequently deacetylated by cellular esterases where it is then oxidized by ROS to produce a fluorescent compound that can detected with flow cytometry (FACS) using the FITC channel.^104^

**Fig. 11:**
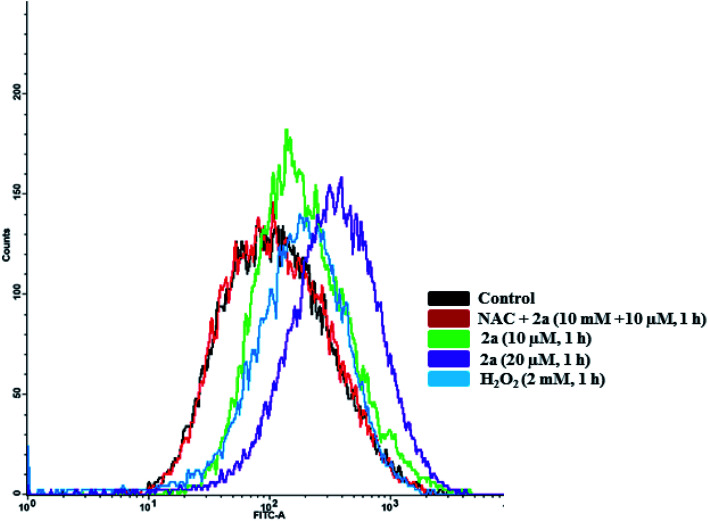
Intracellular ROS induced by **2a**. Compound **2a** induces significant increase in ROS at 10 μM in 1 h. ROS accumulation in MDA-MB-231 cells were monitored with DCF-DA over 1 h. Cells were seeded at a density of 5 × 10^5^ and added compounds from a 5 mM stock solution in DMSO. H_2_O_2_ was used a positive control (30 minutes, 1 mM). Cells were pre-treated with 10 mM *N*-acetyl cysteine (NAC) for 2 h prior to addition of the compounds.

To quantify the amount of ROS produced, we subjected MDA-MB-231 cells to **2a** at 10 and 20 μM to assess concentration dependence. Within 1 hour of treatment, we observed a 1.3× increase in ROS in cells treated with **2a***versus* DMSO (Fig. 11). At twice the concentration, 20 μM, we observed a 1.5× increase in ROS. This shows a slight concentration dependence as well as very fast imbalance of the cell homeostasis. When compared to the H_2_O_2_ control, **2a** produces similar ROS levels in MDA-MB-231 cells (Fig. 11). This suggests that ROS production has a key role in the cell death pathway of these complexes. To further solidify that the DCF-DA fluorescence is from ROS and not other reactive species such as RNS, we pre-treated cells with 10 mM *N*-acetyl cysteine for 2 h. NAC is a natural ROS scavenger and a key component in the formation of glutathione (GSH), which is a powerful antioxidant.^105,106^ After pre-treatment, the cells were subjected to the same concentration of **2a** (10 μM) for 1 h. We observed using FACS that there was no increase in ROS levels in comparison to the control. This reveals that there is a significant amount of ROS being produced in the cells upon treatment and could be a product of OXPHOS inhibition.

### Cell cycle analysis

**Fig. 12:**
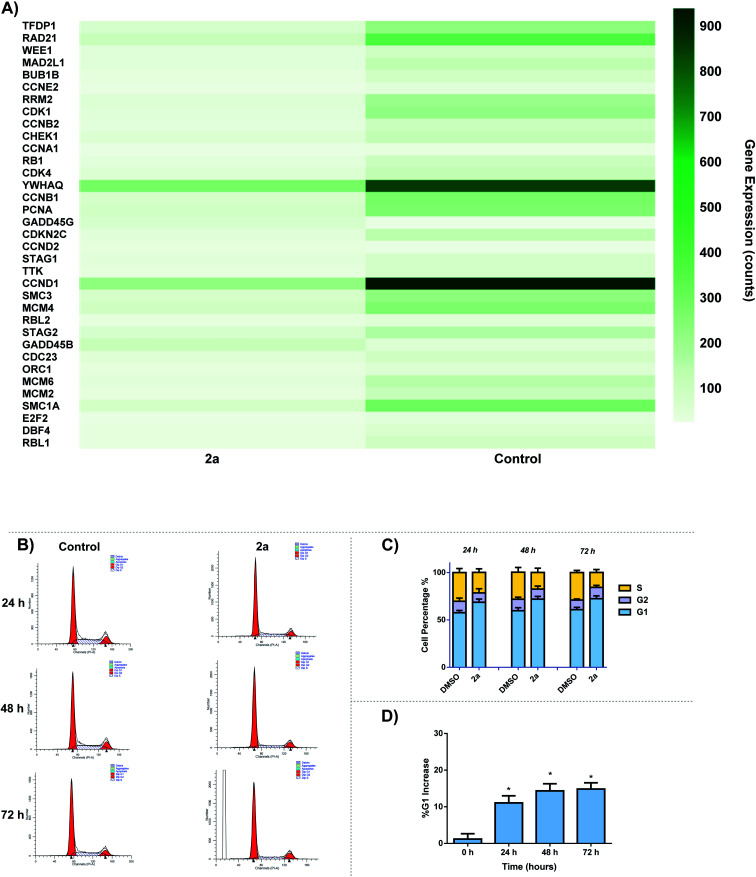
Cell-cycle effect by **2a**. (A) DEGs associated with cell cycle in response to **2a**, (B) representative histogram of the cycle; control (DMSO vehicle) *vs.***2a**, MDA-MB-231 cells were seeded at a density of 2 × 10^5^ cells per well and treated with **2a** at 0.1 μM for 24, 48, and 72 h. (C) Bar graph detailing the change in G1, G2/M, and S phase over 24, 48, and 72 h, data are plotted as the mean ± s.e.m (*n* = 3), (D) bar graph illustrating the % increase in G1 over 72 h after treatment with **2a**, data are plotted as the mean ± s.e.m (*n* = 3) *p < 0.05.

The effect of **2a** on cell cycle was studied by flow cytometry. We observed a time-dependent increase in G0/G1 cell cycle population over 24–72 h period. Analysis of RNA-seq data revealed the down regulation of several cell cycle related genes including cyclin D1 and cyclin dependent kinases (CDK1, CDK4, CCND1) (Fig. 12A). There are several small molecule drug candidates in clinical trials as inhibitors of CDK and induce G1 cell cycle arrest.^107–110^ There are several small molecule drug candidates in clinical trials as inhibitors of CDK4/6 and induce G1 cell cycle arrest.^107–110^ Thus, our finding is relevant for the design of gold based therapies for refractory tumors such as TNBC.^107–110^ After 24 h, a 10% increase can be seen for G1 cell population as well as a 6% decrease in S phase in comparison to the control (Fig. 12B and C). Over the course of 72 h, there is a significant increase in G1 phase, suggesting that **2a** is arresting the cell cycle at the G1 phase. This data enables us to glean insights into the mechanism of action of gold dithiocarbamates, which is clearly differentiated from cisplatin.^111^ The mitochondria control cellular ATP production, it is therefore possible that inhibition of mitochondria respiration and consequently low ATP levels lead to cell cycle arrest.^112–114^

## Conclusion

We have taken advantage of structural diversity of organogold(iii) dithiocarbamates to investigate their anticancer potential and mechanism of action. Solution chemistry reveal that the active stable specie of these compounds in the presence of biological thiols is a neutral organogold(i), which was characterized by NMR spectroscopy, Mass spectrometry and electrochemistry. This points to a mode of action that may involve reactivity to cysteine thiols. These complexes exhibit high *in vitro* cytotoxicity across a panel of cancer cell lines, with inhibitory concentration in the range of 500–2900 nM. The compounds display a 30-fold selectivity for cancer cells over the normal RPE-Neo cells. In addition, the compounds display high cellular uptake in cancer cells of >1000 pmol per million cells. Using whole-cell transcriptomics, we explored the global effects of lead compound **2a** on MDA-MB-231 cells and found that mitochondrial processes related to oxidative phosphorylation, cell cycle, and organelle fission processes were impacted. We utilized Seahorse XF96, to measure oxygen consumption rate of cancer or normal cells in response to **2a** and found selective inhibition of mitochondrial respiration in cancer cells over normal cells (MRC5) at low concentrations (0.1–3 μM). Thus, these agents will be useful as targeted therapy for cancers that shift metabolism and rely on OXPHOS for proliferation. We further validate the cell death pathway by investigating key biological markers including, cell cycle, ROS production, apoptosis, and MMP depolarization. All of these combine to give a unique outlook on the mechanism of action of Au(iii) complexes that has not yet been reported. Overall, this in-depth study provides key information for a foundation to build upon in designing future Au(iii) drug candidates**.**

## Funding sources

We are grateful to the University of Kentucky for funding. The authors acknowledge support of the Center for Pharmaceutical Research and Innovation (NIH P20 GM130456).

## Conflicts of interest

The authors declare no competing financial interest.

## Supplementary Material

SC-011-D0SC03628E-s001

SC-011-D0SC03628E-s002
