# Supplementary material for: Cancer cell-selective modulation of mitochondrial respiration and metabolism by potent organogold(iii) dithiocarbamates
Source: Chem Sci. 2020 Sep 18;11(38):10465–82. doi: 10.1039/d0sc03628e (PMC8162438; doi:10.1039/d0sc03628e)
Supplement: SC-011-D0SC03628E-s001 [file SC-011-D0SC03628E-s001.pdf]

Electronic Supplementary Material (ESI) for Chemical Science.
This journal is © The Royal Society of Chemistry 2020

Supporting Information for

Cancer cell-selective modulation of mitochondrial respiration and metabolism by potent organogold(III) dithiocarbamates

Randall T. Mertens, Sean Parkin, and Samuel G. Awuah,*

Department of Chemistry, College of Arts and Sciences, University of Kentucky, 505 Rose Street, Lexington, KY, 40506-0055, USA.

* awuah@uky.edu

Table of contents **Page #s**

Synthesis and General Experimental Details S2-12
Physical and Chemical Characterization S12-15
***In vitro* Biological Assays** S16-22
Supplementary Figures S23
- **X-ray Tables** S23-27
- **NMR Spectra** S28-63
- **HRMS Spectra** S64-68
- **HPLC Spectra** S69-73
- **UV-vis Spectra** S74-98
- **LC-MS Chromatogram** S99
- **2a + GSH (^{1}H NMR Spectra)** S100-101
- **Cyclic Voltammetry** S102-103
- **Cell Viability** S104-113
- **Mitochondrial Membrane Potential** S114
- **Seahorse XF96** S115
- **GF-AAS Calibration Curves** S116-117

References S118

Synthesis and General Experimental Details

General Experimental Details: All reactions were carried under ambient conditions in air unless otherwise noted. Solvents were of ACS grade (Pharmco-Aaper) and used as is. The starting Au(III) cyclometalated complexes; [2-benzylpyridine]Au(III)Cl_2 and [2-benzoylpyridine]Au(III)Cl_2 were prepared according to a modified procedure from our lab.[1] Sodium dimethyl dithiocarbamate and sodium diethyl dithiocarbamate salts were purchased from Alfa Aesar. 1-(4-bromophenyl)piperazine and 1-(4-Methoxyphenyl)piperazine were purchased from Sigma Aldrich. Ammomium hexafluorophosphate was purchased from Matrix Scientific. Carbon disulfide was purchased from Millipore Sigma and distilled prior to use. Sodium hydroxide pellets were purchased from VWR. Deuterated solvents were purchased from Cambridge Isotope Laboratories (Andover, MA). 3-(4,5-dimethylthiazol-2-yl)-2,5-diphenyltetrazolium bromide (MTT) and 2',7' –dichlorofluorescin diacetate (DCF-DA), and JC-1 were purchased from Cayman Chemicals. NMR spectra were recorded on a Bruker Avance NEO 400 MHz spectrometer and samples calibrated for: ^1H NMR (CD_3CN δ = 1.94 ppm and DMSO-d_6 δ = 2.50 ppm), ^{13}C NMR (CD_3CN δ = 118.26 and 1.32 ppm and DMSO-d_6 δ = 49.00 ppm), and ^{19}F NMR externally referenced to $CFCl_3$ δ = 0.00). Electrospray ionization mass spectrometry (ESI-MS) was performed on an Agilent Technologies 1100 series liquid chromatography/MS instrument. High-resolution mass spectra (HRMS) were obtained by direct flow injection (injection volume = 2 μL) using ElectroSpray Ionization (ESI) on a Waters Qtof API US instrument in the positive mode (CIC, Boston University). Typical conditions are as follows: capillary = 3000 kV, cone = 35 or 15, source temperature = 120 °C, and a desolvation temperature = 350 °C. In addition to spectroscopic characterization, the purity of all compounds was assessed by RP-HPLC using an Agilent Technologies 1100 series HPLC instrument and an Agilent Phase Eclipse Plus C18 column (4.6 mm × 100 mm; 3.5 μm particle size). All compounds were found to be ≥97% pure.

Synthesis:

Sodium piperazine dithiocarbamate:

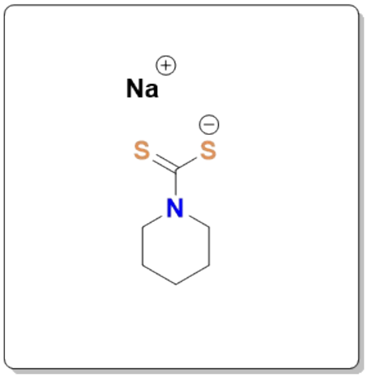

Prepared from a modified procedure in the literature.[2-3] A round bottom was charged with piperazine (850 mg, 10 mmol) and dissolved in 80 mL of ethanol. Sodium hydroxide pellets (400 mg, 10 mmol) were crushed and added portion wise. The mixture was sonicated (15 minutes) until all the NaOH was dissolved. The reaction was cooled to 0 °C using an ice-bath. Carbon disulfide (1.53 g, 20 mmol) was placed in an addition funnel and added dropwise over 10 minutes. The reaction was allowed to warm to room temperature naturally and stirred for 12 h. All solvent was removed *in vacuo* at 50 °C to leave a yellow/white solid. The solid was dissolved in 20 mL of acetone and 250 mL of ether was added to precipitate a white solid, which was vacuum filtered, washed with excess ether, and dried to afford the sodium dithiocarbamate salt. Yield 1.62g, 88%. ^{1}H NMR (400 MHz, DMSO-d_6) δ = 4.27 (t, J = 8 Hz, 4H), 1.52-1.58 (m, 2H), 1.38-1.44 (m, 4H); ^{13}C NMR (101 MHz, DMSO-d_6) δ = 213.09, 50.71, 26.22, 24.86.

Sodium 1-(4-bromophenyl)piperazine dithiocarbamate:

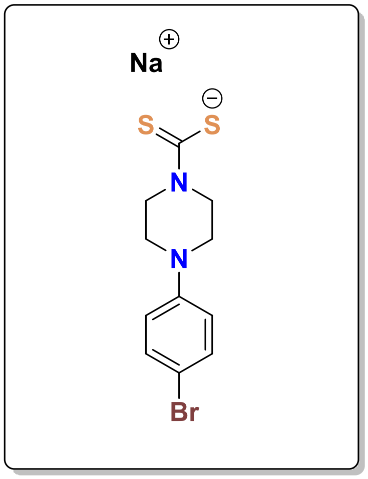

1-(4-bromophenyl)piperazine (241 mg, 1 mmol) and crushed NaOH pellets (40 mg, 1 mmol) were added in 25 mL of methanol and sonicated for 15 minutes to dissolve the NaOH pellets. The reaction was cooled to 0 °C. Carbon disulfide (77 mg, 1 mmol) was placed in an addition funnel and added dropwise over 5 minutes. The reaction was allowed to warm up to room temperature naturally and stirred for 2 h. The solvent was removed *in vacuo* at 70 °C to leave a white solid. The solid was dissolved in acetone (5 mL) and ether (75 mL) was added to precipitate a white solid, which was vacuum filtered, washed with ether, and dried. Yield 200 mg, 59%. ^{1}H NMR (400 MHz, DMSO-d_6) δ = 7.35 (d, J = 4 Hz, 2H,), 6.88 (d, J = 4 Hz, 2H), 4.45 (t, J = 8 Hz,

4H), 3.11 (t, J = 8, 4H); ^{13}C NMR (101 MHz, DMSO-d_6) δ = 214.73, 150.50, 131.86, 117.56, 48.88, 48.23.

Sodium 1-(4-methoxyphenyl)piperazine dithiocarbamate:

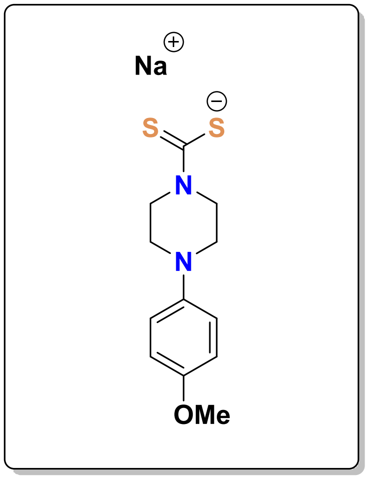

Prepared analogously to sodium 1-(4-bromophenyl)piperazine dithiocarbamate. 1-(4-methoxyphenyl)piperazine (192 mg, 1 mmol), NaOH (40 mg, 1 mmol), CS_2 (77 mg, 1 mmol). Yield 215 mg, 74%. ^{1}H NMR (400 MHz, DMSO-d_6) δ = 6.82 (d, J = 4 Hz, 2H), 6.61 (d, J = 4 Hz, 2H), 4.45 (t, J = 8 Hz, 4H), 3.68 (s, 3H), 2.86 (t, J = 8 Hz, 4H); ^{13}C NMR (101 MHz, DMSO-d_6) δ = 214.49, 153.45, 145.85, 118.03, 114.77, 55.69, 51.31, 50.30, 49.40, 46.12.

General Procedure for the Preparation of Au(III) [C^N] Dithiocarbamate Complexes 1a-e and 2a-e: Complexes were prepared following a slightly modified procedure from the literature.[4] For complexes **1a-1e**, the corresponding Au(III) starting material (2-benzoylpyridine)Au(III)Cl_2 (50 mg, 0.11 mmol) was suspended in 20 mL of MeOH in a 250 mL Erlenmeyer flask and stirred at room temperature. A separate solution of the corresponding sodium dithiocarbamate salt (0.13 mmol) was dissolved in 10 mL of MeOH and added dropwise (500 μL/minute). The mixture gradually turned yellow upon addition of the dithiocarbamate solution. The reaction was stirred at room temperature for 10-12 h. A saturated solution of NH_4PF_6 in DI H_2O was made and added to the MeOH mixture. Excess DI H_2O was added until a precipitate was observed. The solution was filtered and the solid washed with DI H_2O (10 mL), excess Et_2O and vacuum dried for 30 minutes to afford the Au(III) complexes **1a-1e**. Complexes **2a-e** were prepared analogously to **1a-1e** but with the Au(III) starting material (2-benzylpyridine)Au(III)Cl_2 (50 mg, 0.11 mmol).

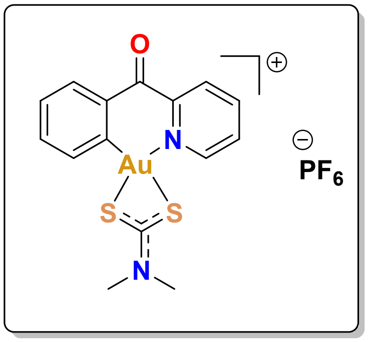

1a: Prepared as described in the general procedure. [(2-benzoylpyridine)Au(III)Cl_2] (50 mg, 0.11 mmol) and NaDMDTC (19 mg, 0.13 mmol). Yield 61 mg, 86%. ^{1}H NMR (400 MHz, MeCN-d_3) δ = 9.01 (d, J = 4 Hz, 1H), 8.51 (d, J = 4 Hz, 2H), 7.97 (quint., J = 8 Hz, 2H), 7.58 (sext., J = 12 Hz, 3H), 3.44 (d, J = 8 Hz, 6H); ^{13}C NMR (101 MHz, MeCN-d_3) δ = 194.11, 190.33, 152.69, 147.60, 145.03, 142.14, 136.13, 131.75, 131.58, 131.36, 130.09, 129.09, 42.18, 41.07; ^{19}F NMR (376 MHz, MeCN-d_3) δ = -131.02, -135.41, -139.81, -144.20, -148.59, -157.37. HRMS (*m/z*) calcd. 499.0213, found 499.0212 [M-PF_6]$^+$, Purity was demonstrated to be >97% by RP-HPLC: R_f = 6.20 minutes using the following method: Flow rate: 1 mL/min; λ = 260 nm; Eluent A = H_2O with 0.1% TFA; Eluent B = MeOH with 0.1% TFA; Solvent Gradient: 0 – 3 min (50:50 H_2O:MeOH), 5 min (40:60 H_2O:MeOH), 7 min (30:70 H_2O:MeOH), 9 min (0:100 H_2O:MeOH), 10 min (20:80 H_2O:MeOH) 12 min until end of run (100:0 H_2O:MeOH).

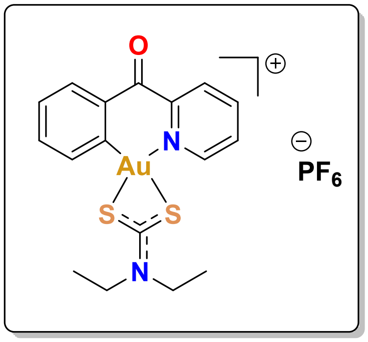

1b: Prepared as described in the general procedure. [(2-benzoylpyridine)Au(III)Cl_2] (50 mg, 0.11 mmol) and NaDEDTC (22 mg, 0.13 mmol). Yield 62 mg, 84%. ^{1}H NMR (400 MHz, MeCN-d_3) δ = 9.03 (d, J = 4 Hz, 1H), 8.54 (d, J = 4 Hz, 2H), 8.02 (quint., J = 8 Hz, 2H), 7.60 (sext., J = 12 Hz, 3H), 3.88 (dq, J = 20, 8 Hz, 4H), 1.39 (dt, J = 24, 8 Hz, 6H); ^{13}C NMR (101 MHz, MeCN-d_3) δ = 193.47, 190.30, 152.60, 147.61, 145.02, 142.32, 136.12, 131.75, 131.56, 131.35, 130.09,

129.09, 49.02, 47.67, 47.47, 12.31, 12.07, 12.02; ^{19}F NMR (376 MHz, MeCN-d$_3$) δ = -131.02, -135.41, -139.81, -144.20, -148.59, -157.37. HRMS (*m/z*) calcd. 527.0526, found 527.0516 $[M-PF_6]^+$, Purity was demonstrated to be >97% by RP-HPLC: R_f = 7.00 minutes using the following method: Flow rate: 1 mL/min; λ = 260 nm; Eluent A = H_2O with 0.1% TFA; Eluent B = MeOH with 0.1% TFA; Solvent Gradient: 0 – 3 min (50:50 H_2O:MeOH), 5 min (40:60 H_2O:MeOH), 7 min (30:70 H_2O:MeOH), 9 min (0:100 H_2O:MeOH), 10 min (20:80 H_2O:MeOH), 12 min until end of run (100:0 H_2O:MeOH).

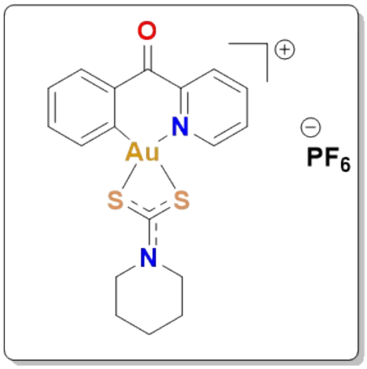

1c: Prepared as described in the general procedure. [(2-benzoylpyridine)Au(III)Cl_2] (50 mg, 0.11 mmol) and NaPipDTC (24 mg, 0.13 mmol). Yield 55 mg, 73%. ^{1}H NMR (400 MHz, MeCN-*d$_3$*) δ = 8.99 (d, *J* = 4, 1 Hz H), 8.51 (d, *J* = 4 Hz, 2H), 7.98 (quint., *J* = 8 Hz, 2H), 7.58 (sext., *J* = 12 Hz, 3H), 3.90 (dt, *J* = 16, 4 Hz, 4H), 1.74-1.82 (m, 6H); ^{13}C NMR (101 MHz, MeCN-*d$_3$*) δ = 191.37, 190.34, 152.64, 147.60, 145.01, 142.37, 136.12, 131.76, 131.64, 131.37, 130.07, 129.09, 52.99, 51.16, 25.97, 24.02; ^{19}F NMR (376 MHz, MeCN-*d$_3$*) δ = -130.85, -135.24, -139.63, -144.02, -148.41, -157.20. HRMS (*m/z*) calcd. 539.0526, found 539.0527 $[M-PF_6]^+$, Purity was demonstrated to be >97% by RP-HPLC: R_f = 7.12 minutes using the following method: Flow rate: 1 mL/min; λ = 260 nm; Eluent A = H_2O with 0.1% TFA; Eluent B = MeOH with 0.1% TFA; Solvent Gradient: 0 – 3 min (50:50 H_2O:MeOH), 5 min (40:60 H_2O:MeOH), 7 min (30:70 H_2O:MeOH), 9 min (0:100 H_2O:MeOH), 10 min (20:80 H_2O:MeOH), 12 min until end of run (100:0 H_2O:MeOH).

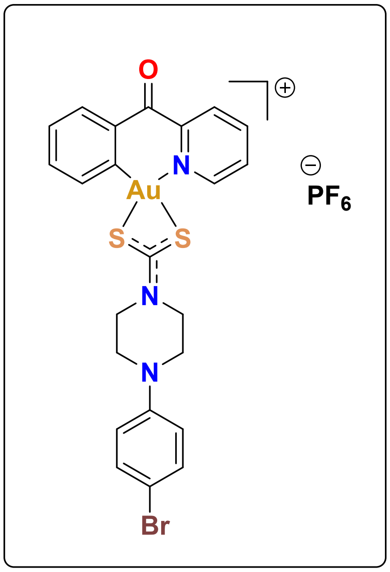

1d: Prepared as described in the general procedure. 2-benzoylpyridine)Au(III)Cl_2] (50 mg, 0.11 mmol) and Na(4-bromophenylpiperazine)DTC (44 mg, 0.13 mmol). Yield 30 mg, 32%. ^{1}H NMR (400 MHz, MeCN-*d₃*) δ = 9.04 (d, *J* = 4 Hz, 1H), 8.54 (d, *J* = 4 Hz, 2H), 8.01 (quint., *J* = 8 Hz, 2H), 7.63 (sext., *J* = 12 Hz, 3H), 7.44 (d, *J* = 8 Hz, 2H), 6.95 (d, *J* = 8 Hz, 2H), 4.12 (t, *J* = 8 Hz, 4H), 3.48 (t, *J* = 12 Hz, 4H); ^{13}C NMR (101 MHz, MeCN-d_3) δ = 194.06, 152.66, 149.61, 144.92, 136.11, 132.58, 131.67, 131.64, 131.43, 130.05, 129.03, 112.64, 50.78, 49.14, 48.07; ^{19}F NMR (376 MHz, MeCN-d_3) δ = -130.66, -135.05, -139.44, -143.84, -148.23, -152.62. HRMS (*m/z*) calcd. 693.9897, found 693.9899 [M-PF_6]$^+$, Purity was demonstrated to be >97% by RP-HPLC: R_f = 9.26 minutes using the following method: Flow rate: 1 mL/min; λ = 260 nm; Eluent A = H_2O with 0.1% TFA; Eluent B = MeOH with 0.1% TFA; Solvent Gradient: 0 – 3 min (50:50 H_2O:MeOH), 5 min (40:60 H_2O:MeOH), 7 min (30:70 H_2O:MeOH), 9 min (0:100 H_2O:MeOH), 10 min (20:80 H_2O:MeOH), 12 min until end of run (100:0 H_2O:MeOH).

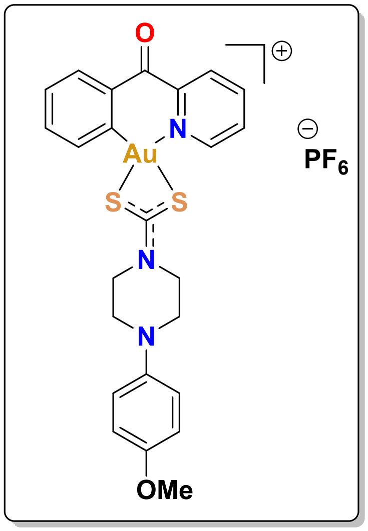

1e: Prepared as described in the general procedure. [(2-benzoylpyridine)Au(III)Cl_2] (50 mg, 0.11 mmol) and Na(4-methoxyphenylpiperazine)DTC (39 mg, 0.13 mmol). Yield 42 mg, 48%. ^{1}H NMR (400 MHz, MeCN-d_3) δ = 8.87 (d, *J* = 8 Hz, 1H), 8.31 (t, *J* = 8 Hz, 2H), 7.82 (t, *J* = 12 Hz, 1H), 7.61 (d, *J* = 8 Hz, 1H), 7.50 (t, *J* = 8 Hz, 1H), 7.43 (d, *J* = 8 Hz, 4H), 7.26 (d, *J* = 10 Hz, 1H), 6.97 (d, *J* = 8 Hz, 2H), 6.88 (d, *J* = 8 Hz, 2H), 4.01 (dt, *J* = 20, 8 Hz, 4H), 3.74 (s, 3H), 3.28 (quint., *J* = 8 Hz, 4H); ^{13}C NMR (101 MHz, MeCN-d_3) δ = 203.55, 155.21, 151.22, 144.80, 134.96, 132.54, 132.36, 128.49, 127.39, 119.54, 115.05, 55.64, 50.69, 50.08, 49.10; ^{19}F NMR (376 MHz, MeCN-d_3) δ = -135.41, -139.80, -144.19, -148.58, -152.98, -157.36. HRMS (*m/z*) calcd. 646.0897, found 646.0896 [M-PF_6]$^+$, Purity was demonstrated to be >97% by RP-HPLC: R_f = 7.14 minutes using the following method: Flow rate: 1 mL/min; λ = 260 nm; Eluent A = H_2O with 0.1% TFA; Eluent B = MeOH with 0.1% TFA; Solvent Gradient: 0 – 3 min (50:50 H_2O:MeOH), 5 min (40:60 H2O:MeOH), 7 min (30:70 H_2O:MeOH), 9 min (0:100 H_2O:MeOH), 10 min (20:80 H_2O:MeOH), 12 min until end of run (100:0 H_2O:MeOH).

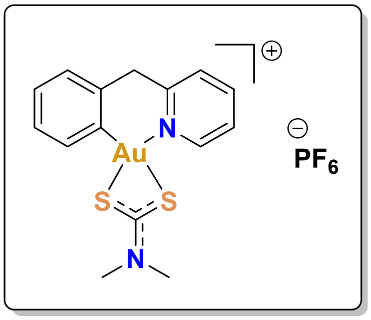

2a: Prepared as described in the general procedure. [(2-benzylpyridine)Au(III)Cl_2] (50 mg, 0.11 mmol) and NaDMDTC (19 mg, 0.13 mmol). Yield 64 mg, 92%. ^{1}H NMR (400 MHz, MeCN-d_3)

δ = 8.86 (d, *J* = 8 Hz, 1H), 8.22 (t, *J* = 8 Hz, 1H), 7.95 (d, *J* = 8 Hz, 1H), 7.62 (t, *J* = 8 Hz, 1H), 7.42 (t, *J* = 8 Hz, 1H), 7.32 (t, *J* = 8 Hz, 1H), 7.19 (t, *J* = 8 Hz, 1H), 4.43 (s, 2H), 3.45 (d, *J* = 4 Hz, 6H); ^{13}C NMR (101 MHz, MeCN-d_3) δ = 195.79, 157.04, 152.22, 146.36, 144.17, 13357, 131.11, 129.61, 129.52, 129.23, 127.73, 126.21, 47.31, 42.12, 40.89; ^{19}F NMR (376 MHz, MeCN-d_3) δ = -130.53, -134.92, -139.31, -143.70, -148.10, -152.48. HRMS (*m/z*) calcd. 485.0421, found 485.0418 [M-PF$_6$]$^+$, Purity was demonstrated to be >97% by RP-HPLC: R$_f$ = 6.26 minutes using the following method: Flow rate: 1 mL/min; λ = 260 nm; Eluent A = H_2O with 0.1% TFA; Eluent B = MeOH with 0.1% TFA; Solvent Gradient: 0 – 3 min (50:50 H_2O:MeOH), 5 min (40:60 H_2O:MeOH), 7 min (30:70 H_2O:MeOH), 9 min (0:100 H_2O:MeOH), 10 min (20:80 H_2O:MeOH), 12 min until end of run (100:0 H_2O:MeOH).

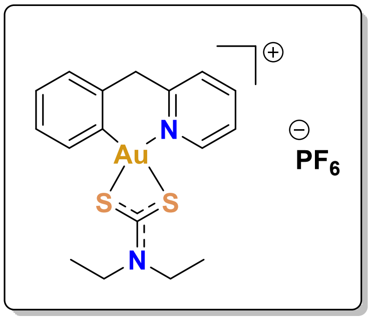

2b: Prepared as described in the general procedure. [(2-benzylpyridine)Au(III)Cl$_2$] (50 mg, 0.11 mmol) and NaDEDTC (22 mg, 0.13 mmol). Yield 64 mg, 88%. ^{1}H NMR (400 MHz, MeCN-d_3)δ = 8.85 (d, *J* = 8 Hz, 1H), 8.21 (t, *J* = 8 Hz, 1H), 7.91 (d, *J* = 8 Hz, 1H), 7.60 (t, *J* = 8 Hz, 1H), 7.40 (t, *J* = 8 Hz, 1H), 7.31 (t, *J* = 8 Hz, 1H), 7.16 (t, *J* = 8 Hz, 1H), 4.45 (s, 2H), 3.65 (td, *J* = 24, 16, 8 Hz, 4H), 1.32-1.48 (m, 6H); ^{13}C NMR (101 MHz, MeCN-d_3) δ = 195.12, 157.03, 152.17, 146.48, 144.16, 133.56, 131.09, 129.60, 129.50, 129.22, 127.75, 126.23, 48.87, 47.37, 47.30, 12.32, 12.04; ^{19}F NMR (376 MHz, MeCN-d_3) δ = -134.25, -138.64, -143.03, -147.42, -151.81, -160.60. HRMS (*m/z*) calcd. 513.0734, found 513.0740 [M-PF$_6$]$^+$, Purity was demonstrated to be >97% by RP-HPLC: R$_f$ = 7.34 minutes using the following method: Flow rate: 1 mL/min; λ = 260 nm; Eluent A = H_2O with 0.1% TFA; Eluent B = MeOH with 0.1% TFA; Solvent Gradient: 0 – 3 min (50:50 H_2O:MeOH), 5 min (40:60 H_2O:MeOH), 7 min (30:70 H_2O:MeOH), 9 min (0:100 H_2O:MeOH), 10 min (20:80 H_2O:MeOH), 12 min until end of run (100:0 H_2O:MeOH).

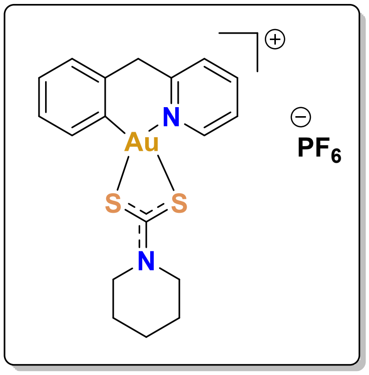

2c: Prepared as described in the general procedure. [(2-benzylpyridine)Au(III)Cl_2] (50 mg, 0.11 mmol) and NaPipDTC (24 mg, 0.13 mmol). Yield 51 mg, 69%. ^{1}H NMR (400 MHz, MeCN-*d*$_3$) δ = 8.83 (d, *J* = 8 Hz, 1H), 8.23 (t, *J* = 8 Hz, 1H), 7.96 (d, *J* = 8 Hz, 1H), 7.62 (t, *J* = 8 Hz, 1H), 7.42 (t, *J* = 8 Hz, 1H), 7.32 (t, *J* = 8 Hz, 1H), 7.20 (t, *J* = 8 Hz, 1H), 4.44 (s, 2H), 3.94 (dt, *J* = 12, 4 Hz, 4H), 1.81-1.85 (m, 6H); ^{13}C NMR (101 MHz, MeCN-*d*$_3$) δ = 193.10, 157.05, 152.22, 146.55, 144.14, 133.59, 131.18, 129.60, 129.49, 129.22, 127.74, 126.22, 52.81, 50.80, 47.33, 26.01, 25.78, 24.03; ^{19}F NMR (376 MHz, MeCN-*d*$_3$) δ = -128.20, -132.59, -136.98, -141.38, -145.77, -150.16. HRMS (*m/z*) calcd. 525.0734, found 525.0731 [M-PF_6]$^+$, Purity was demonstrated to be >97% by RP-HPLC: R_f = 7.48 minutes using the following method: Flow rate: 1 mL/min; λ = 260 nm; Eluent A = H_2O with 0.1% TFA; Eluent B = MeOH with 0.1% TFA; Solvent Gradient: 0 – 3 min (50:50 H_2O:MeOH), 5 min (40:60 H_2O:MeOH), 7 min (30:70 H_2O:MeOH), 9 min (0:100 H_2O:MeOH), 10 min (20:80 H_2O:MeOH), 12 min until end of run (100:0 H_2O:MeOH).

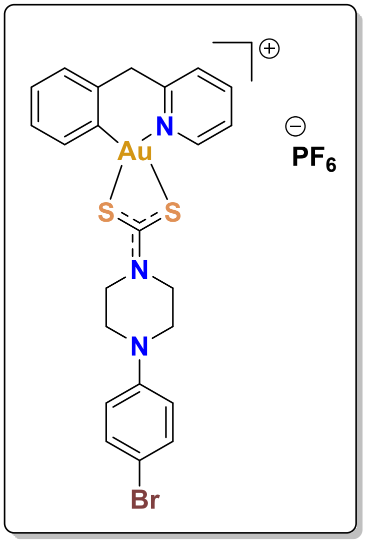

2d: Prepared as described in the general procedure. [(2-benzylpyridine)Au(III)Cl_2] (50 mg, 0.11 mmol) and Na(4-bromophenylpiperazine)DTC (44 mg, 0.13 mmol). Yield 39 mg, 43%. ^{1}H NMR (400 MHz, MeCN-*d$_3$*) δ = 8.86 (d, *J* = 8 Hz, 1H), 8.21 (t, *J* = 8 Hz, 1H), 7.94 (d, *J* = 8 Hz, 1H), 7.61 (t, *J* = 8 Hz, 1H), 7.39-7.45 (m, 4H), 7.33 (t, *J* = 8 Hz, 1H), 7.19 (t, *J* = 8 Hz, 1H), 6.92 (q, *J* =12 Hz, 2H), 4.42 (s, 2H), 4.06-4.11 (m, 4H), 3.42-3.48 (m, 4H); ^{13}C NMR (101 MHz, MeCN-*d$_3$*) δ = 195.05, 156.34, 151.65, 149.05, 145.68, 143.57, 132.87, 131.97, 130.53, 129.04, 128.64, 127.14, 125.60, 118.20, 112.02, 50.07, 48.25, 47.54, 46.65; ^{19}F NMR (376 MHz, MeCN-*d$_3$*) δ = -131.38, -135.77, -140.16, -144.55, -148.94, -157.73. HRMS (*m/z*) calcd. 680.0104, found 680.0104 [M-PF_6]$^+$, Purity was demonstrated to be >97% by RP-HPLC: R_f = 9.40 minutes using the following method: Flow rate: 1 mL/min; λ = 260 nm; Eluent A = H_2O with 0.1% TFA; Eluent B = MeOH with 0.1% TFA; Solvent Gradient: 0 – 3 min (50:50 H_2O:MeOH), 5 min (40:60 H_2O:MeOH), 7 min (30:70 H_2O:MeOH), 9 min (0:100 H_2O:MeOH), 10 min (20:80 H_2O:MeOH), 12 min until end of run (100:0 H_2O:MeOH).

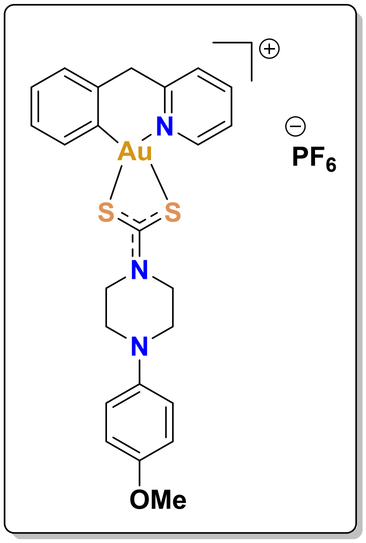

2e: Prepared as described in the general procedure. [(2-benzylpyridine)Au(III)Cl_2] (57 mg, 0.11 mmol) and Na(4-bromophenylpiperazine)DTC (39 mg, 0.13 mmol). Yield 49 mg, 43%. ^{1}H NMR (400 MHz, MeCN-*d$_3$*) δ = 8.86 (d, *J* = 4 Hz, 1H), 8.21 (t, *J* = 8 Hz, 1H), 7.93 (d, *J* = 8 Hz, 1H), 7.61 (t, *J* = 8 Hz, 1H), 7.40 (t, *J* = 8 Hz, 2H), 7.30 (t, *J* = 8 Hz, 1H), 7.18 (t, *J* = 8 Hz, 1H), 6.98 (d, *J* =12 Hz, 2H), 6.89 (d, *J* = 8 Hz, 2H), 4.42 (s, 2H), 4.06-4.12 (m, 4H), 3.75 (s, 3H), 3.28-3.33 (m, 4H); ^{13}C NMR (101 MHz, MeCN-*d$_3$*) δ = 194.74, 156.43, 154.69, 151.74, 145.79, 144.26, 143.64, 132.98, 130.63, 129.12, 129.01, 128.72, 127.21, 125.67, 119.01, 114.53, 55.13, 50.70, 49.61, 48.83, 46.72; ^{19}F NMR (376 MHz, MeCN-*d$_3$*) δ = -130.66, -135.05, -139.44, -

143.84, -148.23, -152.62. HRMS (*m/z*) calcd. 632.1105, found 632.1100 $[M-PF_6]^+$, Purity was demonstrated to be >97% by RP-HPLC: R_f = 8.48 minutes using the following method: Flow rate: 1 mL/min; λ = 260 nm; Eluent A = H_2O with 0.1% TFA; Eluent B = MeOH with 0.1% TFA; Solvent Gradient: 0 – 3 min (50:50 H_2O:MeOH), 5 min (40:60 H_2O:MeOH), 7 min (30:70 H_2O:MeOH), 9 min (0:100 H_2O:MeOH), 10 min (20:80 H_2O:MeOH), 12 min until end of run (100:0 H_2O:MeOH).

Physical and Chemical Characterization

X-ray Crystallography. Crystals of all complexes were grown from slow diffusion of Et_2O into a concentrated solution of MeCN at room temperature. All crystals were mounted using polyisobutene oil on the end of a glass fibre, which had been mounted to a copper pin using an electrical solder. It was placed directly in the cold gas stream of a liquid nitrogen cryostat[5-6] A Bruker D8 Venture diffractometer with graded multilayer focused MoKα X-rays (λ = 0.71073 Å) was used to collect diffraction. Raw data were integrated, scaled, merged, and corrected for Lorentz-polarization effects using the APEX3 package.[7-9] Space group determination and structure solution and refinement were carried out with SHELXT and SHELXL respectively.[10-11] All non-hydrogen atoms were refined with anisotropic displacement parameters. Hydrogen atoms were placed at calculated positions and refined using a riding model with their isotropic displacement parameters (Uiso) set to either 1.2Uiso or 1.5Uiso of the atom to which they were attached. Ellipsoid plots were drawn using SHELXTL-XP.[12] The structures, deposited in the Cambridge Structural Database, were checked for missed symmetry, twinning, and overall quality with PLATON,[13] an R-tensor,[14] and finally validated using CheckCIF.[13]

UV-vis Spectrometry of Complexes 1a-1e and 2a-2e. All spectra were recorded on a Shimadzu UV-1280 model instrument. All complexes were prepared as a 1 mM stock in DMSO. The solutions were freshly prepared prior to use. The solutions were then diluted to a final concentration of 50 µM x 4 mL with DMSO. Prior to spectra acquisition the instrument was blanked with DMSO. Spectra were recorded from 600 nm to 200 nm. The spectra were normalized to maximum absorbance and plotted in GraphPad Prism 6. The representative graph can be seen in the main text (Figure 2).

UV-vis Stability in DMEM, PBS and RPMI-1640 at 37 °C. All spectra were recorded on a Shimadzu UV-1280 model instrument. DMEM was used as purchased from Corning® (4.5 g/L glucose, L-glutamine and sodium pyruvate). PBS was used as received from Corning® (without calcium or magnesium). RPMI-1640 was purchased from Corning© and used as is. Each medium was warmed to 37 °C prior to dilution of the complexes. All complexes were freshly prepared prior to use as a 1 mM stock in DMSO. The solutions were then diluted to 50 µM x 4 mL with the respective biological medium. The amount of DMSO in each solution was 5%, therefore the instrument was blanked with either a 5% DMSO/(PBS/DMEM/RPMI-1640) solution prior to each scan. The solutions were kept in an incubator at a controlled temperature of 37 °C. Time periods of t = 0 h (after preparation of sample), 1 h, 6 h, 12 h, and 24 h were recorded. Prior to each time point, the instrument was blanked with corresponding 5% DMSO solution. The absorbance profile was scanned from 650 nm to 240 nm for each scan. For each complex, the data were normalized to the highest absorbance at t = 0 h and plotted in GraphPad Prism 6. Each respective graph can be seen in supplementary Figures S57-S83.

Reactivity with GSH (UV-vis Spectroscopy). Stock solutions of the complexes were prepared by dissolving enough compound to achieve 1 mM x 1 mL in DMSO. The stock solutions were diluted down to 50 µM by taking 50 µL of the stock and diluting to 1 mL with PBS. A separate stock solution of GSHwas prepared as a 1 mM stock x 5 mL. This stock solution was then diluted to 50 µM in PBS. All spectra were recorded on a Shimadzu UV-1280 model instrument. A blank was prepared by mixing 50 µL of DMSO and 1.95 mL of PBS. Time intervals of 5 minutes and 12 h were recorded. Prior to each recording the instrument was blanked. The equimolar solutions of the complex (50 µM) and GSH(50 µM) were mixed in a 1:1 ratio to achieve a final concentration of 25 µM the UV-vis spectra recorded at the indicated time intervals. For each reaction, the spectrum was normalized to the highest absorbance and plotted in GraphPad Prism 6. Each respective graph can be seen in supplementary Figures S84-S93.

Reactivity with NAC (UV-vis Spectroscopy). The experimental procedure was performed identically to that of the reaction involving GSH noted above. For each reaction, the spectrum

was normalized to the highest absorbance and plotted in GraphPad Prism 6. Each respective graph can be seen in supplementary Figures S94-S103.

Reactivity with GSH (HPLC). All solvents used were of HPLC grade. All spectra were recorded using an Agilent Technologies 1100 series HPLC instrument and an Agilent Phase Eclipse Plus C18 column (4.6 mm × 100 mm; 3.5 μm particle size). Compound **2a** was prepared as a 5 mM stock in MeCN. **GSH** was prepared as a 5 mM stock in H_2O. The stocks were diluted to a final concentration of 2.5 mM with the appropriate solvent and each respective HPLC spectrum recorded. For the reaction of **2a** + **GSH**, 1 mL from each of the 5 mM stocks were mixed in a 1:1 ratio to achieve a 2 mL solution with a final concentration of 2.5 mM. The sample was then subjected to HPLC analysis. All spectra were recorded using the following method: Flow rate: 1 mL/min; λ = 240 nm; Eluent A = H_2O with 0.1% TFA; Eluent B = MeOH with 0.1% TFA; Solvent Gradient: 0 – 3 min (50:50 H_2O:MeOH), 5 min (40:60 H_2O:MeOH), 7 min (30:70 H_2O:MeOH), 9 min (0:100 H_2O:MeOH), 10 min (20:80 H_2O:MeOH), 12 min until end of run (100:0 H_2O:MeOH). Data were plotted using GraphPad Prism 6. Each respective graph can be seen in the main text (Figure 4).

LC-MS Analysis of the 2a + GSH Adduct. After performing the reaction of **2a** + **GSH**, a single new peak was observed. The same solution was then subjected to LC-MS analysis (2.5 mM) using an Agilent 1200 HPLC with a direct flow injection with a HPLC auto sampler without a column, λ = 280 nm, (injection volume: 40 μL, flow rate: 0.2 mL/ min). ESI positive mode was taken with a source temperature of 120 °C, desolvation temperature of 300 °C, Capillary Vat 3.5 kV while Cone was set at 35. Data was plotted and analyzed using Mestrenova. The respective graph can be seen in the main text (Figure 4). The chromatogram can be found in Figure S104.

Reactivity with GSH (^{1}H NMR spectroscopy). Stock solutions comprised of a 1 mL, 20 mM solution of **2a** or **L-GSH** in DMSO-d_6 was prepared. The solution of **GSH** was sonicated for 5 minutes to dissolve all of the reagent. 500 μL of each solution were mixed in a 1:1 ratio to produce a final concentration of 10 mM in DMSO-d_6. The solution was then analyzed by ^{1}H NMR spectroscopy. The solution was further analyzed at different time intervals, t = 1 h, 6 h, 12 h, and 24 h. Spectra were plotted using Mestrenova. (Figure 4 and Figure S105-S106)

Cyclic Voltammetry. All electrochemical measurements were recorded with a scan rate of 0.1 V/s with a three-segment sweep and a sample interval of 0.001 V. The quiet time was set to 2 seconds and sensitivity and 1 x10^{-5} A/V. All solutions were freshly prepared prior to use. All spectra were recorded using a CH instruments 650E potentiostat. The electrodes used were all 3 mm: glassy carbon working electrode (CHI104), Ag/AgCl reference electrode (CHI111), and a platinum wire counter electrode (CHI115). Compound **2a** (8.8 mg/ 7 mL), NaDMDTC (2 mg/ 7 mL), and (2-benzylpyridine)$AuCl_2$ (6.1 mg/ 7 mL) were prepared as a 2 mM solution in anhydrous DMSO using 0.1 M NBu_4PF_6 (271 mg/ 7 mL) as the electrolyte. The samples were purged with nitrogen for 15 minutes and recorded. GSH (4.3 mg/ 7 mL) was prepared as a 2 mM solution in deoxygenated DI H_2O using 0.1 M anhydrous $NaClO_4$ (85 mg / 7 mL) as the electrolyte. Due to poor solubility of GSH in DMSO, the reaction between **2a** and GSH was performed in a mix of solvents. Compound **2a** (8.8 mg/ 7 mL) was dissolved in anhydrous DMSO while GSH (4.3 mg/ 7 mL) was dissolved in deoxygenated DI H_2O. 3.5 mL of each solution were mixed in a 1:1 ration to achieve a final concentration of 1 mM x 7 mL. To this was then added 0.1 M NBu_4PF_6 as the electrolyte (271 mg). All data were plotted in GraphPad Prism 6. The graphs for **2a** and **2a + GSH** can be found in the main text (Figure 4). Graphs for NaDMDTC, GSH, and (2-benzylpyridine)$AuCl_2$ can be found in supplementary Figures (S107-S109). Each graph is representative of 3 individual experiments.

In Vitro Biological Assays

Cell Culture. All cell lines were purchased from ATCC and routinely grown in a humidified incubator at 37 °C with 5-10% CO_2. MDA-MB-231, MDA-MB-175, MRC5, RPE-NEO, H460, and K562 were grown in DMEM supplemented with 10% FBS, 1% amphotericin and 1% penicillin/streptomycin. A2780 cells were grown in RPMI supplemented with 10% FBS, 1% amphotericin, and 1% penicillin/streptomycin, and 4 mM glutamine. All supplements along with PBS and trypsin-EDTA were purchased from Corning Inc. and used as is.

Cell Viability of 2a + GSH Adduct in MDA-MB-231. Cells were grown to confluency and added trypsin to collect the cells. The cells were washed with 2 mL of PBS and suspended in 10 mL of DMEM. The cells were centrifuged at 2000 rpm for 5 minutes. The pellet was washed with 2 mL of PBS and suspended in 5 mL of DMEM. The cells were then plated in three separate 96-well clear bottom plate at a density of 2,000 cells/well. The cells were allowed to adhere overnight. The adduct was prepared by taking a 10 mM stock of **2a** in DMSO and **GSH** in DMEM and mixing in a 1:1 ratio to achieve a 5 mM stock of the adduct. The adduct solution was diluted to 100 μM working concentration with DMEM. The adduct was then added at a 3x dilution starting at 50 μM for the highest concentration and the cells incubated for 72 h at 37 °C with 5-10% CO_2. The medium was removed and a solution of MTT (100 μL, prepared by dissolving MTT at 5 mg/mL and diluting by 10x with DMEM) was added to each well and incubated for 4 h at 37 °C with 5-10% CO_2. The dye was removed from each well and 100 μL of DMSO was added to induce cell lysis. The plates were read using a Genios plate reader (λ = 570 nm). The experiment was performed in triplicate. Data are plotted as the mean ± s.e.m. (n = 3) (Figure S110).

Cell Viability of 1a-1e and 2a-2e (Adherent Cell Lines). The cell viability of all 10 complexes were performed in MDA-MB-231, MDA-MB-175, A2780, RPE-NEO and MRC5. The cell viability of compound **2a** was also determined in H460. Cells were grown to confluency and trypsin was added to detach and harvest cells. The cells were washed with 2 mL of PBS and suspended in 10 mL of the appropriate media. The cells were centrifuged at 2000 rpm for 5 minutes and the pellet washed with 2 mL of PBS then suspended in 5 mL of the appropriate

media. The cells were plated at a density of 2,000 cells/well in a 96-well clear bottom plate and allowed to adhere overnight at 37 °C with 5-10% CO_2. The compounds were prepared as a stock in DMSO and used fresh. The compounds were added at seven concentrations with a 3x serial dilution starting at 50 μM for the highest concentration and incubated at 37 °C for 72 h with 5-10% CO_2. The medium was removed and a solution of MTT (100 μL, prepared by dissolving MTT at 5 mg/mL and diluting by 10x with DMEM) was added to each well and incubated for 4 h at 37 °C with 5-10% CO_2. The dye was removed from each well and 100 μL of DMSO was added to induce cell lysis. The plates were read using a Genios plate reader (λ = 570 nm). The experiment was performed in triplicate and data are plotted as the mean ± s.e.m. (n = 3). Data for **2a** can be found in the main text (Figure 6) and the other complexes can be seen in the supplementary figures (S111 – S129).

Cell Viability of 1a-1e and 2a-2e (Suspended Cell Lines). The cell viability of **2a** was determined in K562. Cells were grown to confluency and centrifuged at 2000 rpm for 5 minutes to collect the cell pellet. The cells were washed with 5 mL of PBS, suspended in 5 mL of DMEM, and centrifuged again at 2000 rpm for 5 minutes to collect the pellet. The pellet was then washed with 2 mL of PBS and suspended in 5 mL of DMEM. The cells were plated at density of 2,000 cells/well in a 96-well white bottom plate. **2a** was prepared as a stock solution in DMSO and used fresh. The compounds were added at seven concentrations with a 3x serial dilution starting at 50 μM for the highest concentration and incubated at 37 °C for 72 h with 5-10% CO_2. The cells were removed from the incubator and allowed to rest at room temperature for 30 minutes. To each designated well was then added 20 μL of CellTiter-Glo solution and orbitally shaken for 5 minutes and the luminescence (1000 ms integration and 150 ms gain) acquired on a Genios plate reader. The experiment was performed in triplicate. Data are plotted as the mean ± s.e.m. (n = 3) as seen in the main text (Table 2 and Figure 6).

Apoptosis Analysis. MDA-MB-231 cells were seeded at a density of 5 x 10^5 cells/well in a 6 well clear bottom plate with a final media volume of 2 mL. The cells were allowed to adhere overnight at 37 °C. A stock of **2a** was prepared fresh in DMSO and added to the desired well at a concentration of 1 μM with a final volume of 2.5 mL and incubated for 4 h at 37 °C. A stock of H_2O_2 was prepared in PBS and the cells treated at a final concentration of 2 mM for 1 hour as a

positive control. When ready for analysis, the media were removed and the wells washed with 5 mL of PBS. The cells were trypsinized (1 mL), 5 mL of DMEM were added to each well, and total volume collected and centrifuged to pellet the cells. The cells were resuspended in 2 mL of fresh media, counted, and reconstituted to a concentration of 1 x 10^5 cells/mL. The cells were centrifuged again, and the pellet suspended in 500 µL of Annexin binding buffer. To each sample was added 5 µl of Annexin V-FITC and 5 µl PI and incubated in the dark at room temperature for 5 minutes. The samples were then subjected to FACS analysis. Graphs are representative of three technical replicates. Percentages are plotted as the mean ± s.e.m. (n = 3) as seen in the main text (Figure 10).

Immunoblotting. MDA-MB-231 cells were seeded at density of 5 x 10^5 cells/well in a 6 well clear bottom plate with a final volume of 2 mL and allowed to adhere overnight at 37 °C. Compound **2a** was prepared as a stock in DMSO and added to the respective wells at the specified concentrations of 0.1, 1, and 10 µM and treated for 12 h. The cells were washed with PBS (3 x 3 mL) and were scraped into SDS-PAGE loading bufer (64 mM Tris-HCl (pH 6.8)/9.6% glycerol/2% SDS/5% β-mercaptoethanol/0.01% bromophenol blue) and incubated at 95 °C on a heat block for 10 min. The samples were cooled and stored at −20 °C until ready for use. Whole cell lysates were resolved by 4–20% sodium dodecylsulfate polyacrylamide gel electrophoresis (SDS-PAGE; 100 V for 35 min) followed by electro transfer to a PVDF (350 mA for 1 h). Membranes were blocked using 3% (w/v) bovine serum albumin (BSA) in PBST (PBS/0.1% Tween 20) and incubated with specific primary antibodies (Cell Signaling Technology) overnight at 4 °C. On the following day, after washing with PBST (3 ×5 mL), the membrane was incubated with horseradish peroxidase-conjugated secondary antibodies (Cell Signaling Technology) in freshly prepared BSA blocking solution. Immuno complexes were detected with the ECL detection reagent (BioRad) and analyzed using a BioRad imager with a chemiluminescence filter. Blots can be found in the main text (Figure 10C)

Cellular Uptake. For whole cellular uptake, MDA-MB-231 cells were seeded at a density of 1 x 10^6 cells/mL in a 6 well clear bottom plate with a volume of 2.5 mL and allowed to adhere overnight at 37 °C. Compounds were prepared as a stock in DMSO and added to each well at a final concentration of 5 µM and treated for 24 h. Auranofin was used as a comparative control

and treated at a final concentration of 5 μM and treated for 24 hours as well. The cells were then collected by trypsinization and centrifuged at 2000 rpm for 5 minutes to form a pellet. The pellet was suspended in 1 mL of DMEM, transferred to a 1.5 mL Eppendorf tube, and centrifuged again at 2000 rpm for 5 minutes. The media were removed, washed with PBS twice, and the pellet stored at -20 °C until analysis. Prior to analysis, the pellets were suspended in 0.5 mL of concentrated HNO_3 and agitated for 1 minute. The solution was transferred to a 15 mL Falcon tube and then 4.5 mL of DI H_2O was added. The samples were then subjected to analysis with ICP-OES. Data is represented as the mean ± s.e.m. (n = 3) as seen in the main text (Figure 5A).

Cellular fraction uptake was conducted in a similar manner per the instructions from the RayBio® Nuclear Extraction Kit Protocol (RayBiotech, Inc.). MDA-MB-231 cells were seeded at a density of 2 x 10^6 in 100 mm petri dishes and incubated overnight at 37 °C. **2a** was added at 5 μM for 24 hours. Once the fractions had been isolated, the concentrate was subjected to GF-AAS analysis. Prior to analysis, a calibration curve using Au in varying concentrations (0, 10, 20, 50 μg/mL) was performed. Data is represented as the mean ± s.e.m. (n = 3) as seen in the main text (Figure 5B). The calibration curve for these samples can be found in Fig. S133.

Whole cellular uptake with pre-incubation of uptake inhibitors was performed as follows. MDA-MB-231 cells were seeded at a density of 3 x 10^5 in clear-bottomed six-well plates and incubated at 37 °C overnight. The cells were then pre-treated with the following inhibitors: NaN_3 (1 mM), methyl-β-cyclodextrin (5 mM), chlorpromazine hydrochloride (28 nM), wortmannin (50 nM), genistein (200 μM), for 1 hour. For the +4 °C sample, the cells were cooled to +4 °C prior to addition of **2a**. Following pre-treatment, the media were removed and washed with PBS (3 x 2 mL). The cells were then incubated with **2a** (5 μM for 24 hours) at 37 °C and the +4 °C sample incubated at the same concentration at the indicated lower temperature. After treatment, the cells were collected both, the medium and tryspsinzed cells combined, centrifuged at 2000 rpm for 5 minutes to form a pellet. The pellet was suspended in 1 mL of DMEM, transferred to a 1.5 mL Eppendorf tube, and centrifuged again at 2000 rpm for 5 minutes. The media were removed, washed with PBS twice, and the pellet stored at -20 °C until analysis. Prior to analysis, the pellets were suspended in 300 μL of 70% HNO_3. 100 μL aliquot of this stock was then diluted to 1 mL and subjected to GF-AAS. A calibration curve using Au in varying concentrations (0, 10, 20, 50 μg/mL) was performed. After every 10 samples, the calibration was performed again for quality control assurance. Data is represented as the mean ± s.e.m. (n = 3) as seen in the main

text (Figure 5C). Samples 37 °C, NaN_3, methyl-β-cyclodextrin, and chlorpromazine hydrochloride were calculated using calibration curve Fig. S134 and samples wortmannin, genistein, and +4 °C were calculated using calibration curve Fig. S135.

Differential Gene Expression using RNA-Sequencing. MDA-MB-231 cells were seeded on petri dish (100 mm x 15 mm) and allowed to grow to 85% confluency. The cells were then treated with **2a** at a concentration of 1 µM for 12 h at 37 °C. Cells were harvested and 1 x 10^7 cells were collected. High quality RNA was isolated using RNA Qiagen kit following manufacturer's protocol and subsequently sent to Novogene® for RNA-sequencing and analysis. Prior to analysis samples were required to pass three tests before library construction: 1) nanodrop for RNA purity (OD_{260}/OD_{280}), 2) agarose gel electrophoresis for RNA integrity and potential contamination, and 3) Agilent 2100 check RNA integrity. Next, the NEB library was constructed from mRNA enrichment and fragmentation, followed by reverse transcription, second strand cDNA synthesis, end repair, addition of adaptor, and finally amplification with PCR. After library construction, qPCR was used to accurately quantify the library effective concentration (> 2 nM), in order to ensure the library quality. Raw reads were removed *via* the following parameters: 1) remove reads containing adaptors, 2) remove reads containing N > 10% (N represents bases that could not be determined), 3) the Qscore (Quality value) of over 50% bases of the read was ≤ 5. Novogene® then uses STAR to accomplish the mapping reads to the reference genome. Gene expression level is then estimated by the abundance of transcripts (count of sequencing) that mapped to genome or exon where read counts are proportional to gene expression level, gene length and sequencing depth. Samples are then subjected to analysis using Pearson's correlation coefficient and principal component analysis for statistical significance.

Mitochondrial Membrane Potential (JC-1). MDA-MB-231 cells were plated at a density of 5 x 10^5 cells/plate using a glass bottom petri dish fitted with a #1.5 cover slip with a final volume of 1.5 mL and allowed to adhere overnight at 37 °C. Compound **2a** was prepared as a stock in DMSO and added at a final concentration of 10 µM. The cells were treated for 6 h at this concentration. CCCP was prepared as a stock in DMSO and added at a final concentration of 100 µM and the cells treated for 1 h. This was used as a positive control. After the indicated treatment time, a working solution of the JC-1 dye (Cayman Chemicals) was prepared by adding

100 µL of dye into 900 µL of DMEM. Note: the working solution of JC-1 should always be prepared fresh and not stored for long-term use. Then, 100 µL/mL of DMEM were added to the cells and incubated at 37 °C for 20 minutes. Prior to imaging, the media was removed and replaced with room temperature PBS (2 mL). The cells were then visualized using confocal microscopy on a Nikon A1R Inverted Confocal Microscope. J-aggregates were imaged with (excitation/emission: 510/ 590 nm) and J-monomers with (excitation/emission: 488/525 nm). Each image is representative of three technical replicates as seen in the main text (Figure 8). A field of view (FOV) image can be found in the supplementary figures (Figure S130).

ROS Analysis (DFC-DA). MDA-MB-231 cells were seeded at a density of 5 x 10^5 cells/well in a 6-well clear bottom plate with a final volume of 2 mL and allowed to adhere overnight at 37 °C. Compound **2a** was prepared as a stock in DMSO and added to the desired wells at a final concentration of both 5 and 10 µM and treated for 1 h. For the positive control, H_2O_2 was used and diluted with PBS and added at a final concentration of 1 mM with a treatment time of 1 h. For the wells that were pre-treated with NAC, a stock solution of NAC in DMEM was added at a concentration of 10 mM for 2 h prior to the addition of **2a**. Following treatment, the media were removed, cells were washed with 5 mL of PBS, and collected via trypsinization by centrifuging at 2000 rpm for 5 minutes. The pellet was then suspended in 500 µL of a 10 µM DCF-DA solution in PBS and incubated for 30 minutes at 37 °C in the dark. The cells were then centrifuged again at 2000 rpm for 5 minutes, the pellet washed with PBS (3 x 1 mL) and suspended in 500 µL of PBS. The cells were then analyzed using FACS with the FITC channel (excitation, λ = 488 nm). Each data set is representative of three technical replicates as seen in the main text (Figure 11).

Mitochondrial Metabolism Analysis with Seahorse XF96 Analysis. The initial step of Seahorse XF96 analysis included optimization of the cell density (Figure S110). In this stage MDA-MB-231 cells were seeded at a range of densities from 2000 cells/well to 100,000 cells/well, followed by optimization of the FCCP injection concentration (Figure S131-132) used (0.6 µM of 1.2 µM). The optimum conditions were determined to be 30,000 cells/well and an FCCP injection concentration of 0.6 µM. All Seahorse XF96 experiments with MDA-MB-231 were performed under these conditions. The cells were seeded the night prior to the experiment

with a final volume of 100 µL and incubated overnight at 37 °C. Compound **2a** was prepared as a stock in DMSO and diluted to a working concentration of 200 µM with Seahorse XF96 assay buffer and then subsequently serial diluted by 3x to achieve multiple concentrations. The assay was performed using a pneumatic injection method of **2a**, with the final injection concentrations of 0.1, 1, 3 , and 11 µM. This was followed by injection of oligomycin (1.5 µM), FCCP (0.6 µM) and rotenone/ antimycin A (0.5 µM). The metabolic parameters are calculated as seen in the supplementary information of the following papers.[15-16]

The Seahorse XF96 analysis for MRC5 was performed in an identical manner to MDA-MB-231 except that MRC5 cells were seeded at a density of 50,000 cells/well and an FCCP injection concentration of 0.6 µM was used.

Cell Cycle Analysis. MDA-MB-231 cells were seeded at a density of 2 x 10^5 cells/well in a 6 well clear bottom plate with a final media volume of 2 mL and allowed to adhere overnight 37 °C. Compound **2a** was prepared fresh as a stock in DMSO and added at a final concentration of 0.1 µM with a final volume of 2.5 mL. Cells were treated with **2a** for time periods of 24 h, 48 h, and 72 h. After the desired treatment period, the medium was removed and added to a 15 mL Falcon tube. The wells were washed with 5 mL of PBS and added to the Falcon tube. The cells were trypsinized (1 mL) and added 5 mL of fresh DMEM. All media were combined, and the tube centrifuged at 2000 rpm for 5 minutes to collect the pellet. The media were decanted, and the pellet suspended in 1 mL of PBS, which was then transferred to a 1 mL Eppendorf tube, centrifuged at 2000 rpm for 5 minutes and suspended in 70% EtOH/PBS solution. These solutions were stored at 4 °C until ready for analysis. Once all treatments had been collected, the cells were collected by centrifuging at 2000 rpm for 5 minutes. The cells were washed twice with PBS (1 mL) and suspended in a 50 µL of RNase solution (100 mg/mL) and 200 µL of a 50 mg/mL PI solution. The solutions were then filtered through a 5 mL polystyrene round-bottom tube fit with a cell-strainer cap. The samples were then analyzed with FACS. Data are representative of three technical replicates with percentages plotted as the mean ± s.e.m. (n = 3) as seen in the main text (Figure 12).

Supplementary Figures and Tables:

X-Ray Crystallographic Details:

Table S1: X-ray Parameters of **1c**

X-ray Structural Data and Crystal Refinement	
	**1c**
**Empirical Formula**	$C_{18}H_{18}AuF_6N_2OPS_2$
**Molecular Weight (g/mol)**	684.4
**Temperature (K)**	90.0(2)
**X-ray Radiation (Å)**	Mo Kα (0.71073 Å)
**Crystal System, Space Group**	Monoclinic, P2(1)/c
**Unit Cell Dimensions (A), (o)**	a =24.858(5) Å alpha = 90<br>b = 7.8812(16) Å beta = 97.33(3)<br>c = 21.855(4) Å gamma = 90
**Volume**	4246.6(15) Å^3
**Z**	8
**Absorption Coefficient**	2.141 mm^{-1}
**F(000)**	2624
**Crystal Size (mm)**	0.080 x 0.070 x 0.050
**Theta Range**	2.478 to 27.559
**Completeness to Theta = 25.242**	99.90%
**F^2**	1.021
**Final R indices [I>2sigma(I)]**	R1 = 0.0153, wR2 = 0.0323

Table S2: X-ray Parameters of **2a**

X-ray Structural Data and Crystal Refinement

	**2a**
**Empirical Formula**	$C_{15}H_{16}AuF_6N_2PS_2$
**Molecular Weight (g/mol)**	630.35
**Temperature (K)**	90.0(2)
**X-ray Radiation (Å)**	Mo Kα (0.71073 Å)
**Crystal System, Space Group**	Orthorhombic, P2(1)2(1)2(1)
**Unit Cell Dimensions (A), (o)**	a = 7.7242(2) Å alpha = 90<br>b = 11.1154(2) Å beta = 90<br>c = 12.8365(4) Å gamma = 90
**Volume**	1874.73(7) $Å^3$
**Z**	4
**Absorption Coefficient**	2.233 mm^{-1}
**F(000)**	1200
**Crystal Size (mm)**	0.100 x 0.080 x 0.040
**Theta Range**	2.615 to 27.495
**Completeness to Theta = 25.242**	99.90%
**F^2**	0.89
**Final R indices [I>2sigma(I)]**	R1 = 0.0117, wR2 = 0.0246

Table S3: X-ray Parameters of **2b**

X-ray Structural Data and Crystal Refinement

	**2b**
**Empirical Formula**	$C_{17}H_{20}AuF_6N_2PS_2$
**Molecular Weight (g/mol)**	658.41
**Temperature (K)**	90.0(2)
**X-ray Radiation (Å)**	Mo Kα (0.71073 Å)
**Crystal System, Space Group**	Orthorhombic, Pna2/1
**Unit Cell Dimensions (A), (o)**	a = 14.1989(4) Å alpha = 90<br>b = 18.1306(5) Å beta = 90<br>c = 8.1046(2) Å gamma = 90
**Volume**	2086.4(1) $Å^3$
**Z**	4
**Absorption Coefficient**	2.096 mm^{-1}
**F(000)**	1264
**Crystal Size (mm)**	0.110 x 0.030 x 0.020
**Theta Range**	2.666 to 27.462
**Completeness to Theta = 25.242**	99.90%
**F^2**	1.304
**Final R indices [I>2sigma(I)]**	R1 = 0.0238, wR2 = 0.0517

Table S4: X-ray Parameters of **2c**

X-ray Structural Data and Crystal Refinement

	**2c**
**Empirical Formula**	$C_{18}H_{20}AuF_6N_2PF_6S_2$
**Molecular Weight (g/mol)**	670.42
**Temperature (K)**	90.0(2)
**X-ray Radiation (Å)**	Mo Kα (0.71073 Å)
**Crystal System, Space Group**	Orthorhombic, Pna2/1
**Unit Cell Dimensions (A), (o)**	a = 14.4750(4) Å alpha = 90<br>b = 17.9946(5) Å beta = 90<br>c = 8.0979(2) Å gamma = 90
**Volume**	2109.27(11) $Å^3$
**Z**	4
**Absorption Coefficient**	7.309 mm^{-1}
**F(000)**	1288
**Crystal Size (mm)**	0.090 x 0.090 x 0.050
**Theta Range**	3.034 to 27.528
**Completeness to Theta = 25.242**	99.80%
**F^2**	1.055
**Final R indices [I>2sigma(I)]**	R1 = 0.0144, wR2 = 0.0345

Table S5: X-ray Parameters of **2e**

X-ray Structural Data and Crystal Refinement

	2e
**Empirical Formula**	$C_{26}H_{30}AuF_6N_3O_{1.5}PS_2$
**Molecular Weight (g/mol)**	814.58
**Temperature (K)**	90.0(2)
**X-ray Radiation (Å)**	Mo Kα (0.71073 Å)
**Crystal System, Space Group**	Trigonal, P-1
**Unit Cell Dimensions (A), (o)**	a = 9.7833(2) Å alpha = 104.794(1)<br>b = 10.9241(2) Å beta = 90.616(1)<br>c = 13.6127(3) Å gamma = 93.105(1)
**Volume**	1404.09(5) Å^3
**Z**	2
**Absorption Coefficient**	1.927 mm^{-1}
**F(000)**	798
**Crystal Size (mm)**	0.110 x 0.100 x 0.060
**Theta Range**	2.566 to 27.493
**Completeness to Theta = 25.242**	99.90%
**F^2**	1.089
**Final R indices [I>2sigma(I)]**	R1 = 0.0147, wR2 = 0.0344

NMR Spectra of the Sodium dithiocarbamate salts:

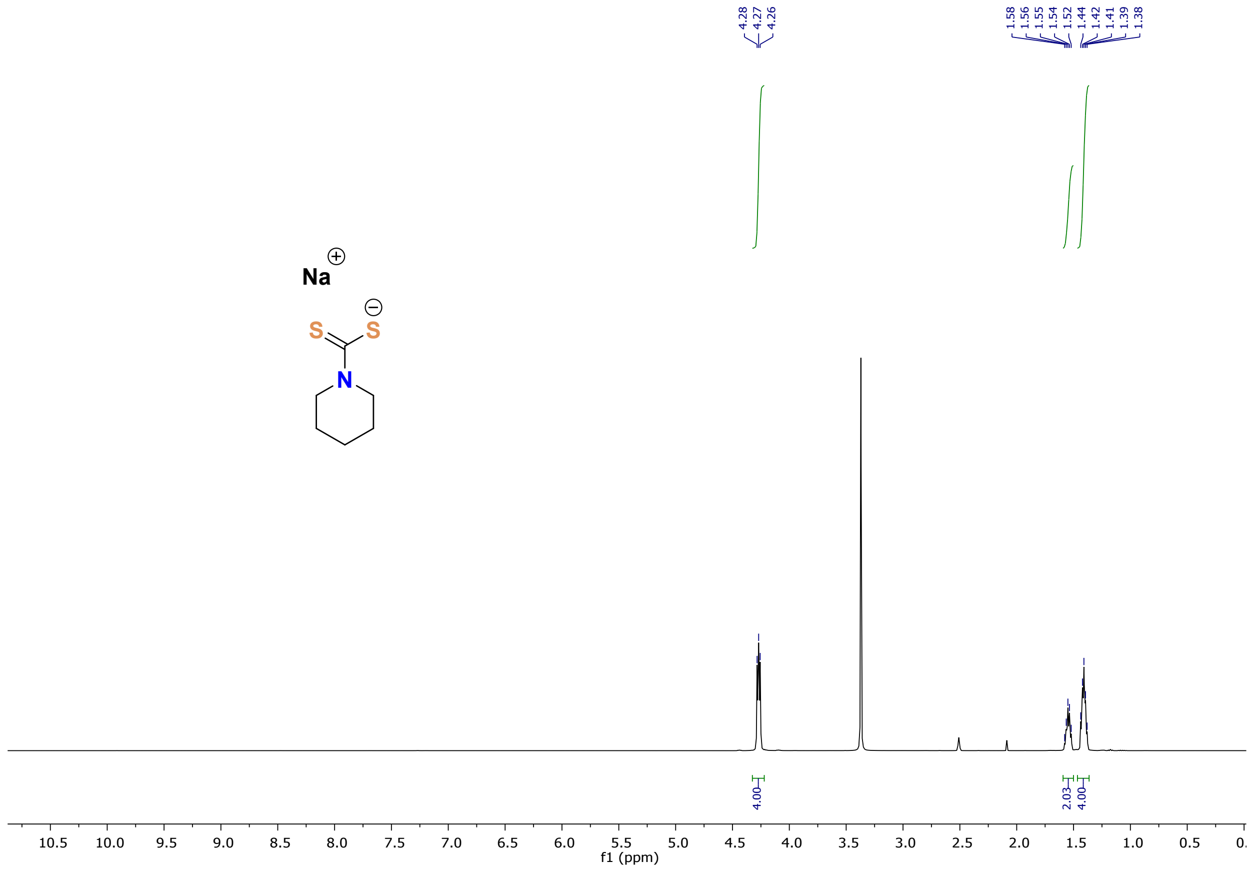

Fig. S1. ^{1}H NMR spectrum of **sodium piperadine dithiocarbamate** in DMSO-d_6 at 298K.

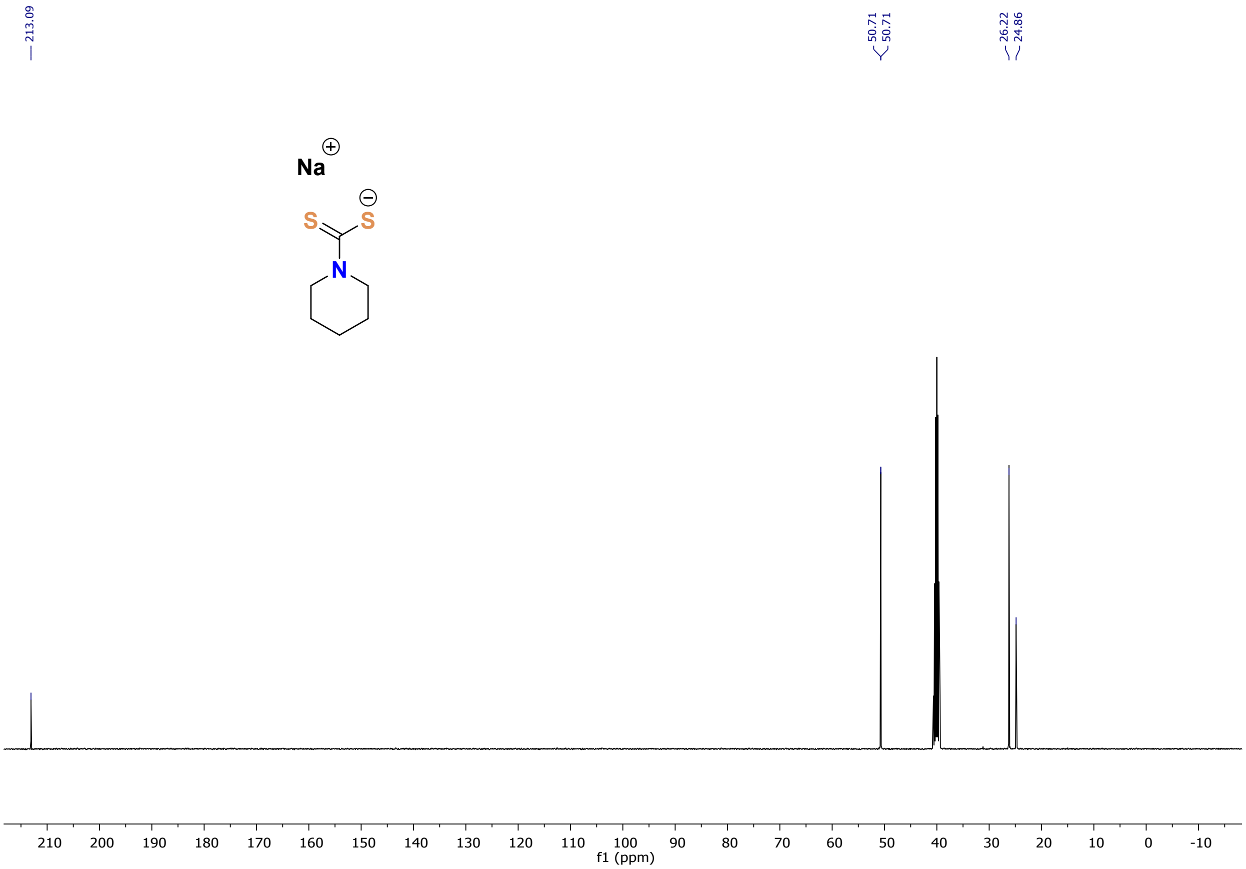

Fig. S2. ^{13}C NMR spectrum of **sodium piperadine dithiocarbamate** in DMSO-d_6 at 298K.

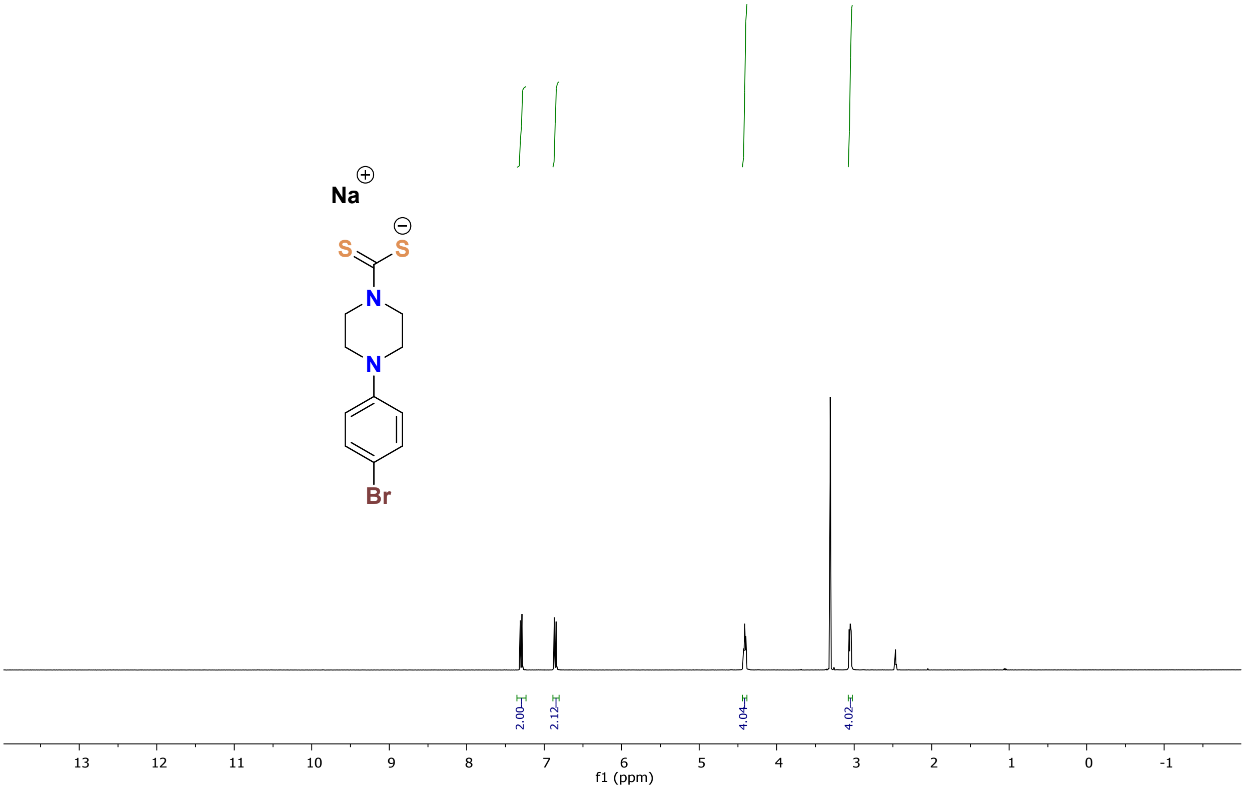

Fig. S3. ^{1}H NMR spectrum of **sodium 1-(4-bromophenyl)piperazine dithiocarbamate** in DMSO-d_6 at 298K.

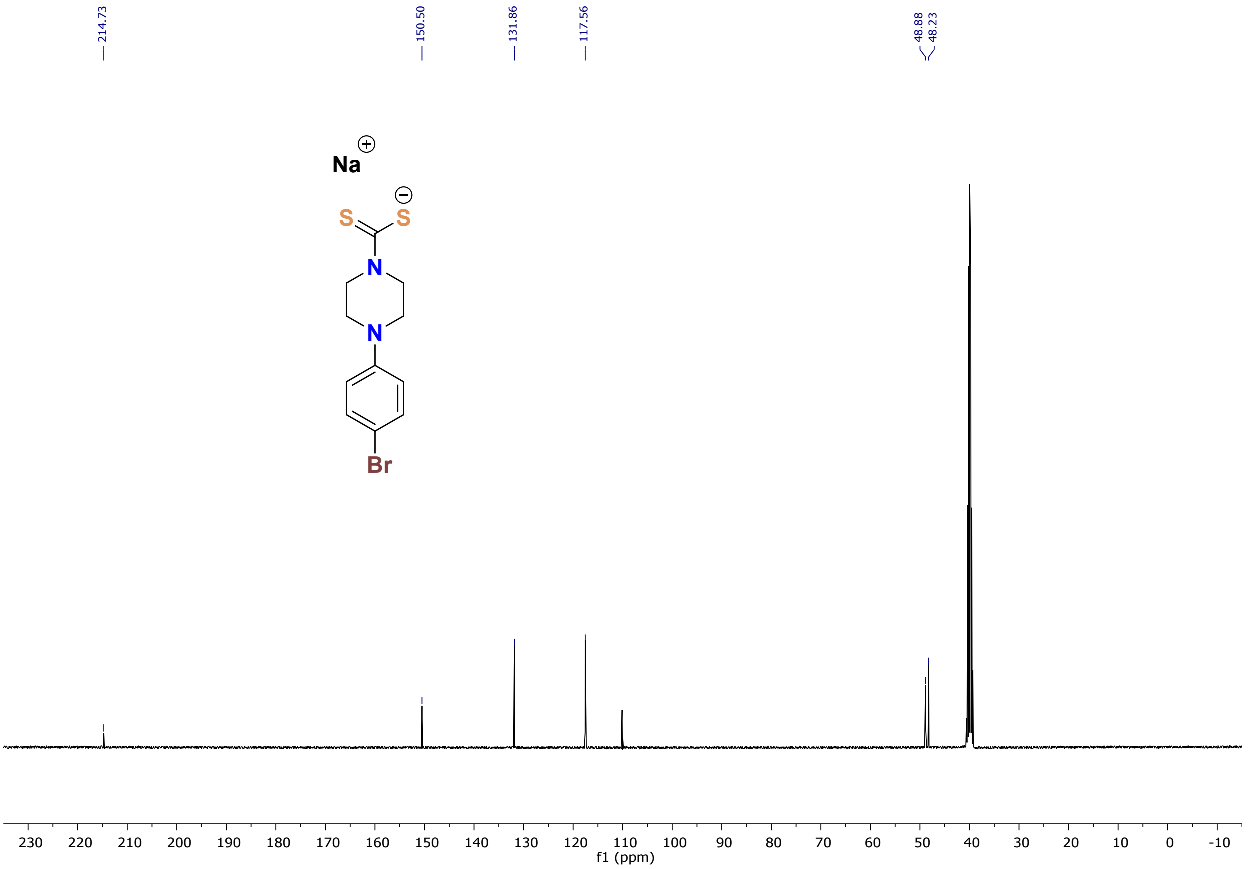

Fig. S4. ^{13}C NMR spectrum of **sodium 1-(4-bromophenyl)piperazine dithiocarbamate** in DMSO-d_6 at 298K.

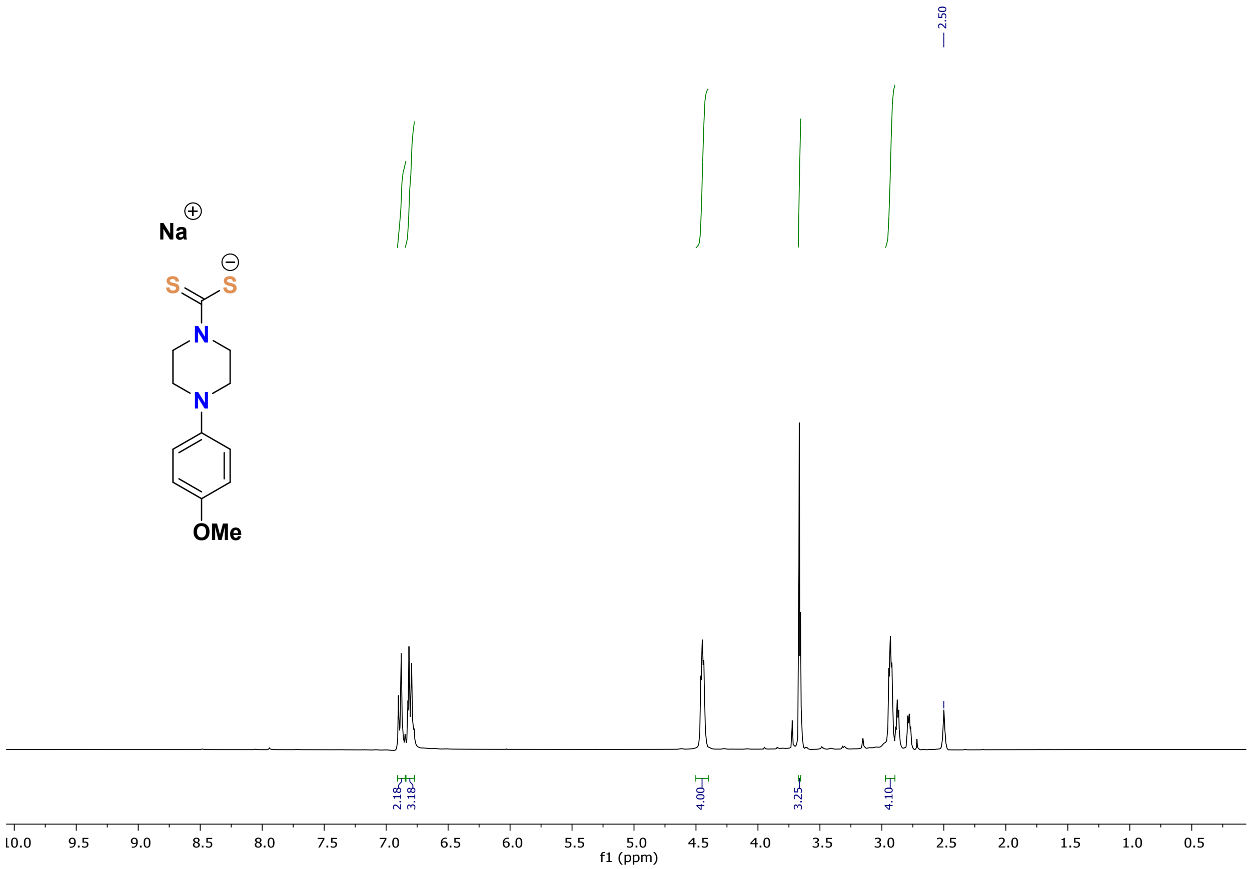

Fig. S5. ^{1}H NMR spectrum of **sodium 1-(4-methoxyphenyl)piperazine dithiocarbamate** in DMSO-d_6 at 298K.

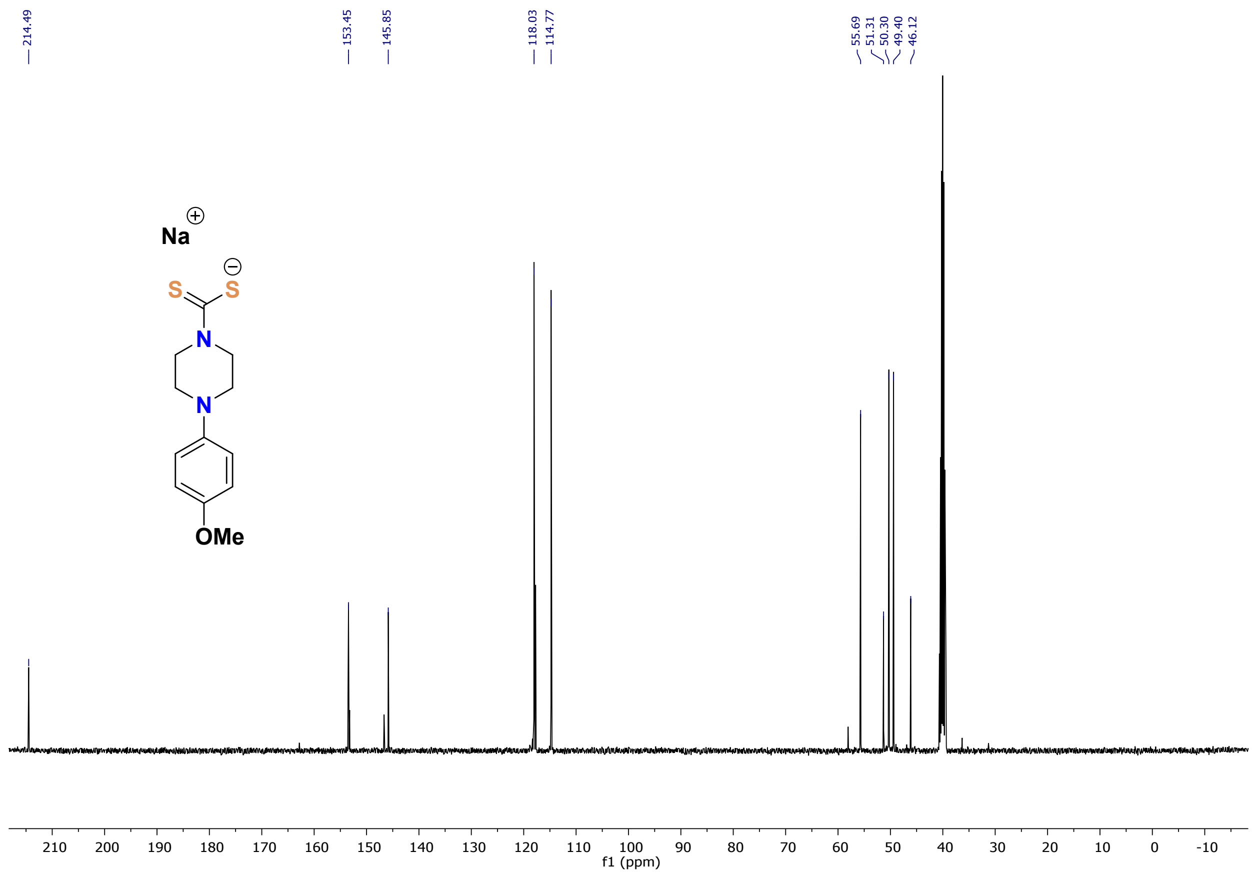

Fig. S6. ^{13}C NMR spectrum of **sodium 1-(4-methoxyphenyl)piperazine dithiocarbamate** in DMSO-d_6 at 298K.

NMR Spectra of 1a-1e and 2a-2e:

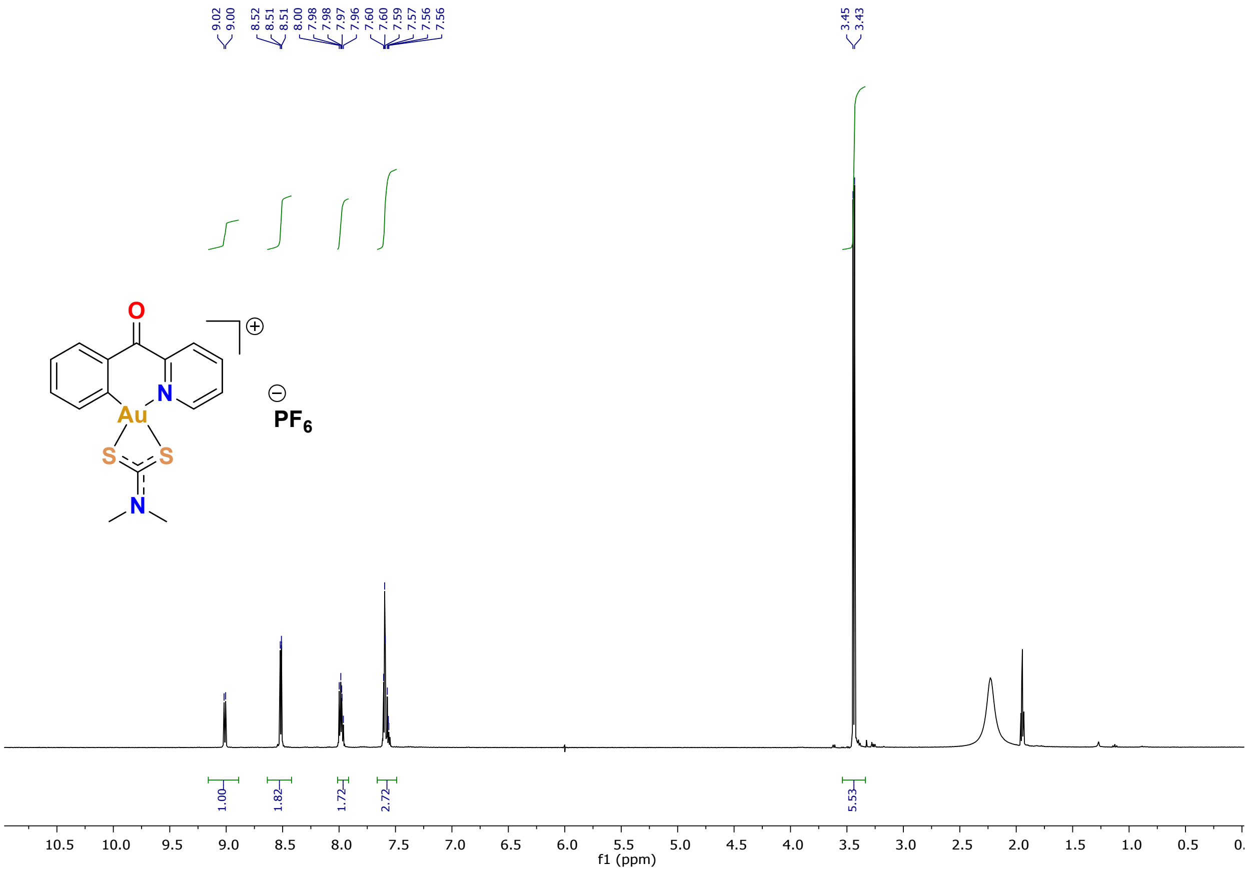

Fig. S7. ^{1}H NMR spectrum of **1a** in CD_3CN at 298K.

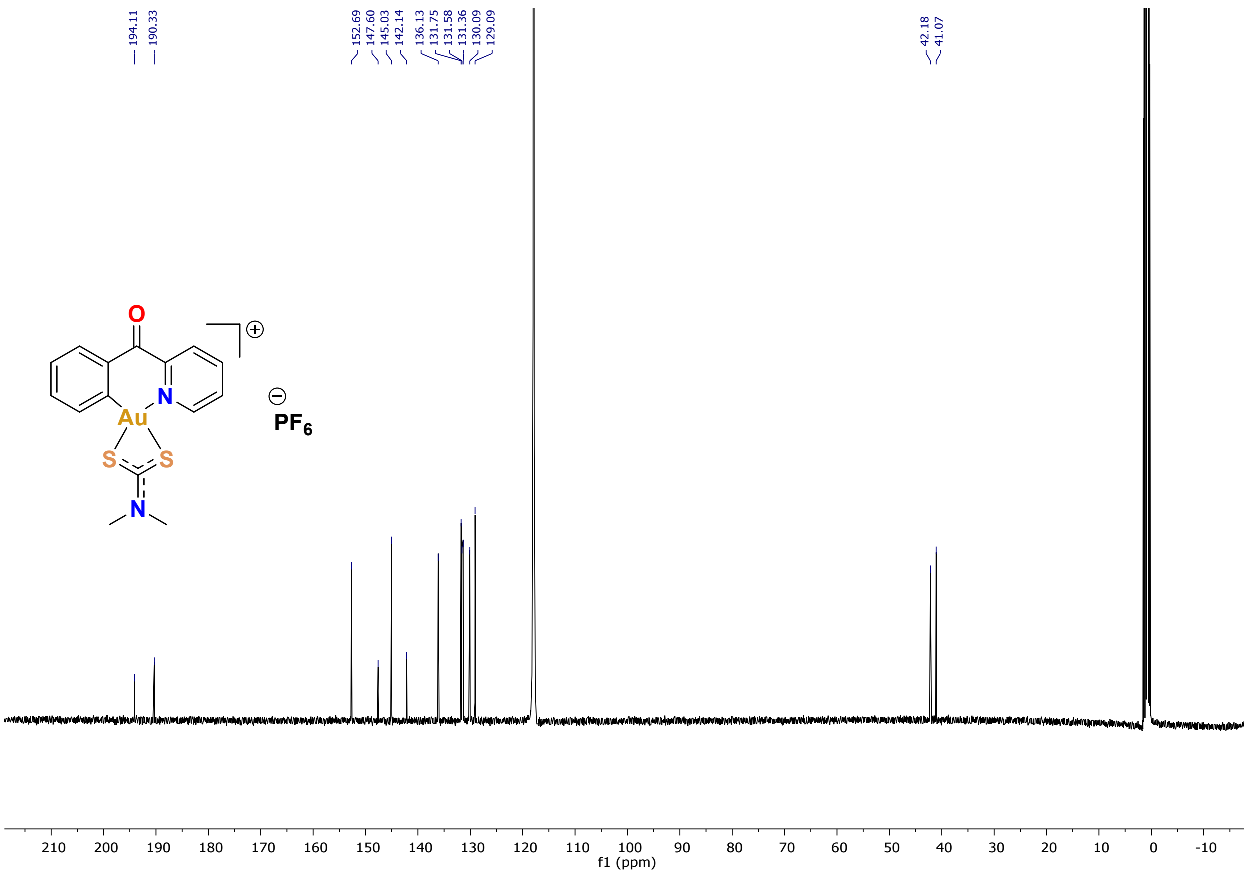

Fig. S8. ^{13}C NMR spectrum of **1a** in CD_3CN at 298K.

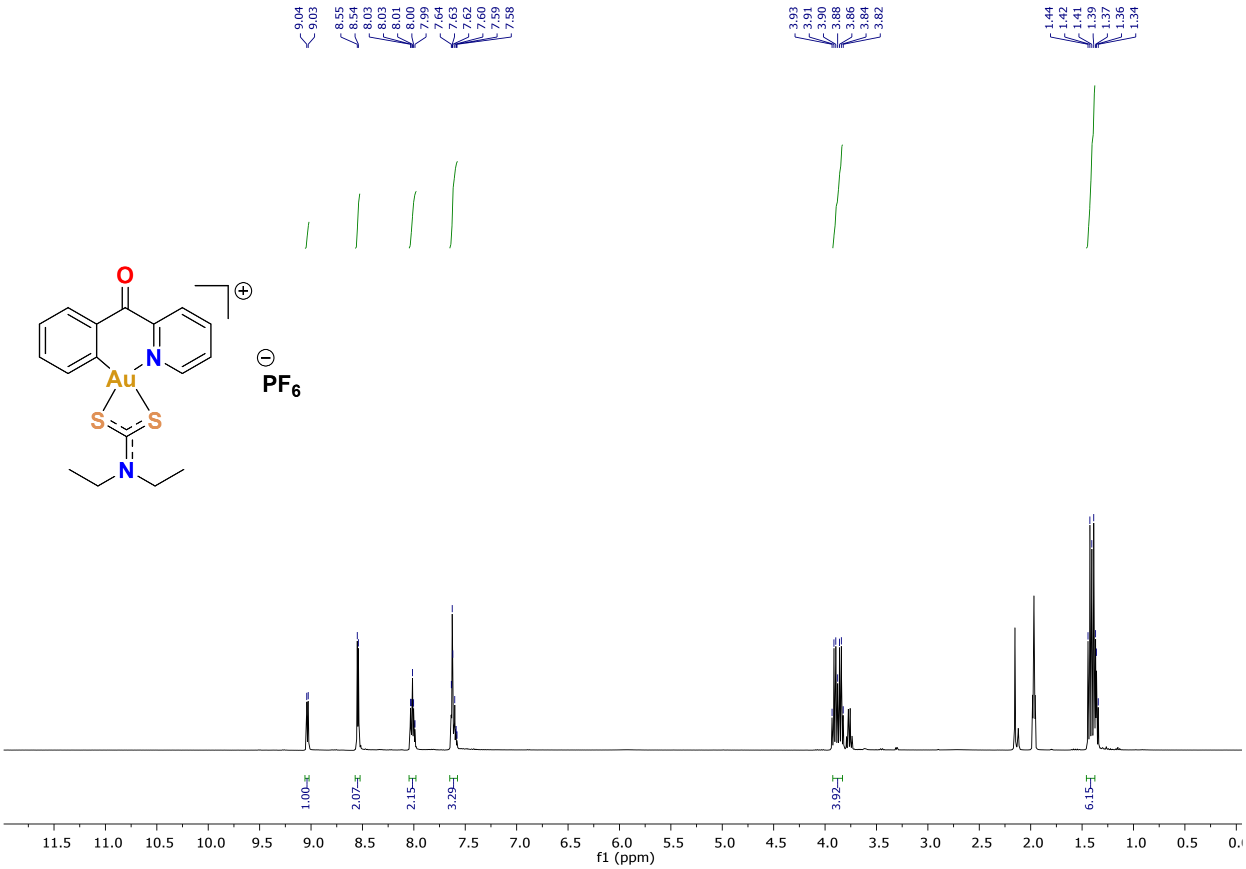

Fig. S9. ^{1}H NMR spectrum of **1b** in CD_3CN at 298K.

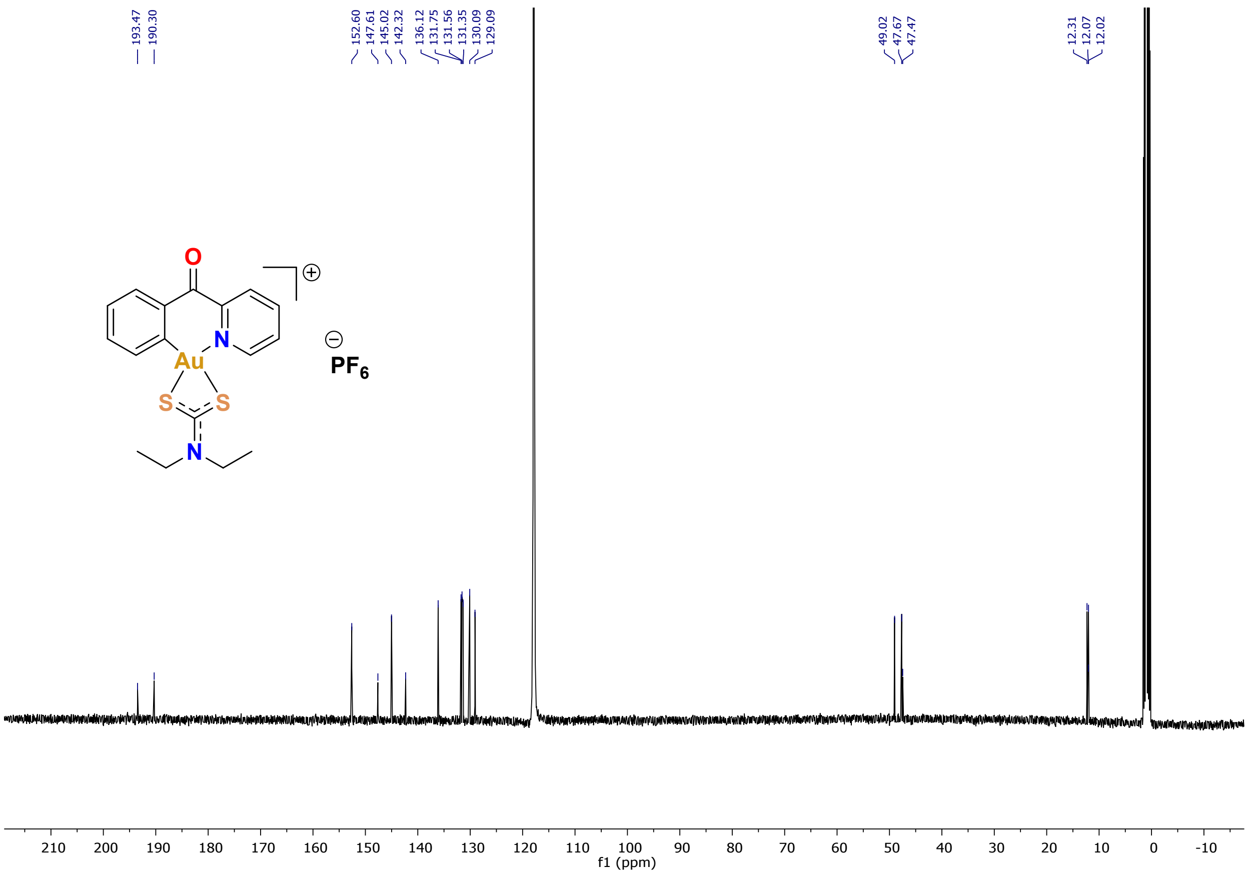

Fig. S10. ^{13}C NMR spectrum of **1b** in CD_3CN at 298K.

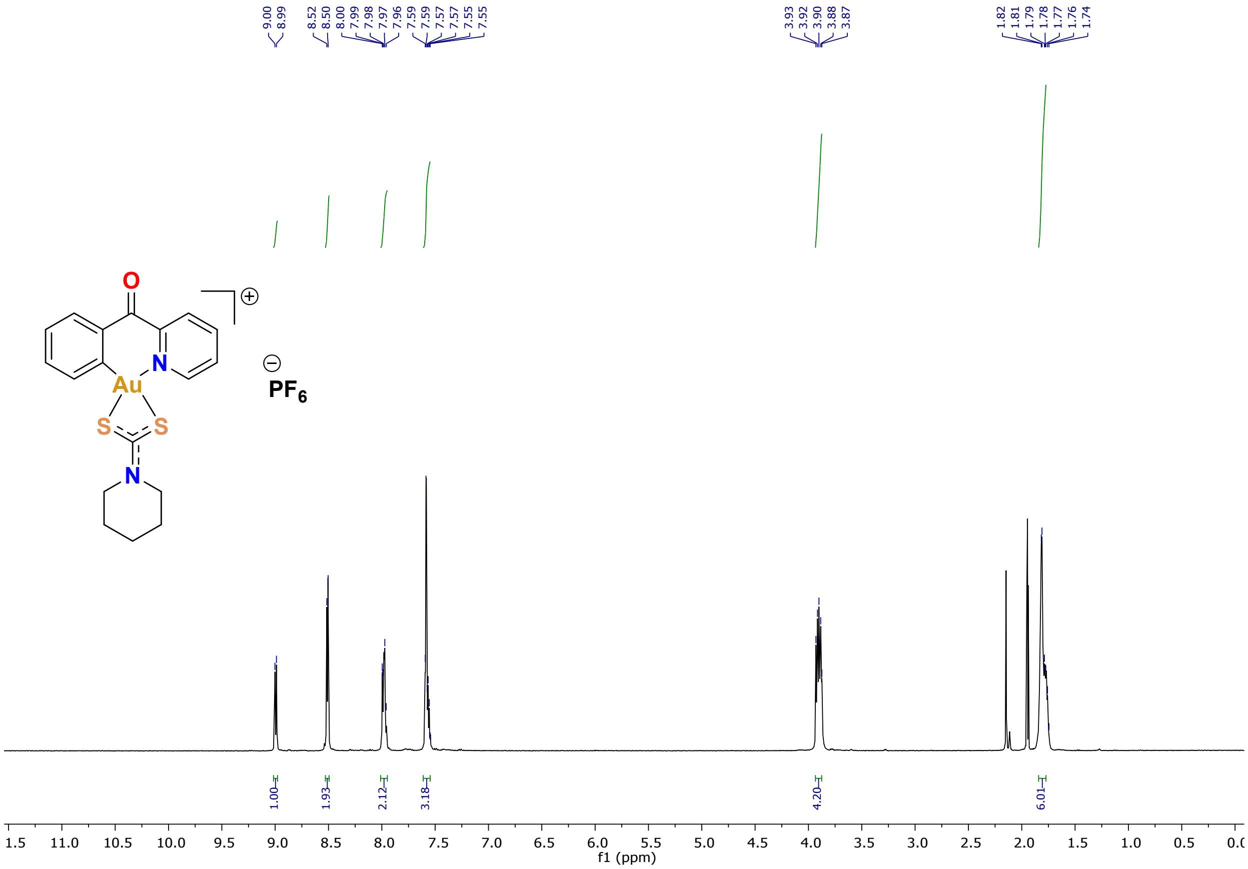

Fig. S11. ^{1}H NMR spectrum of **1c** in CD_3CN at 298K.

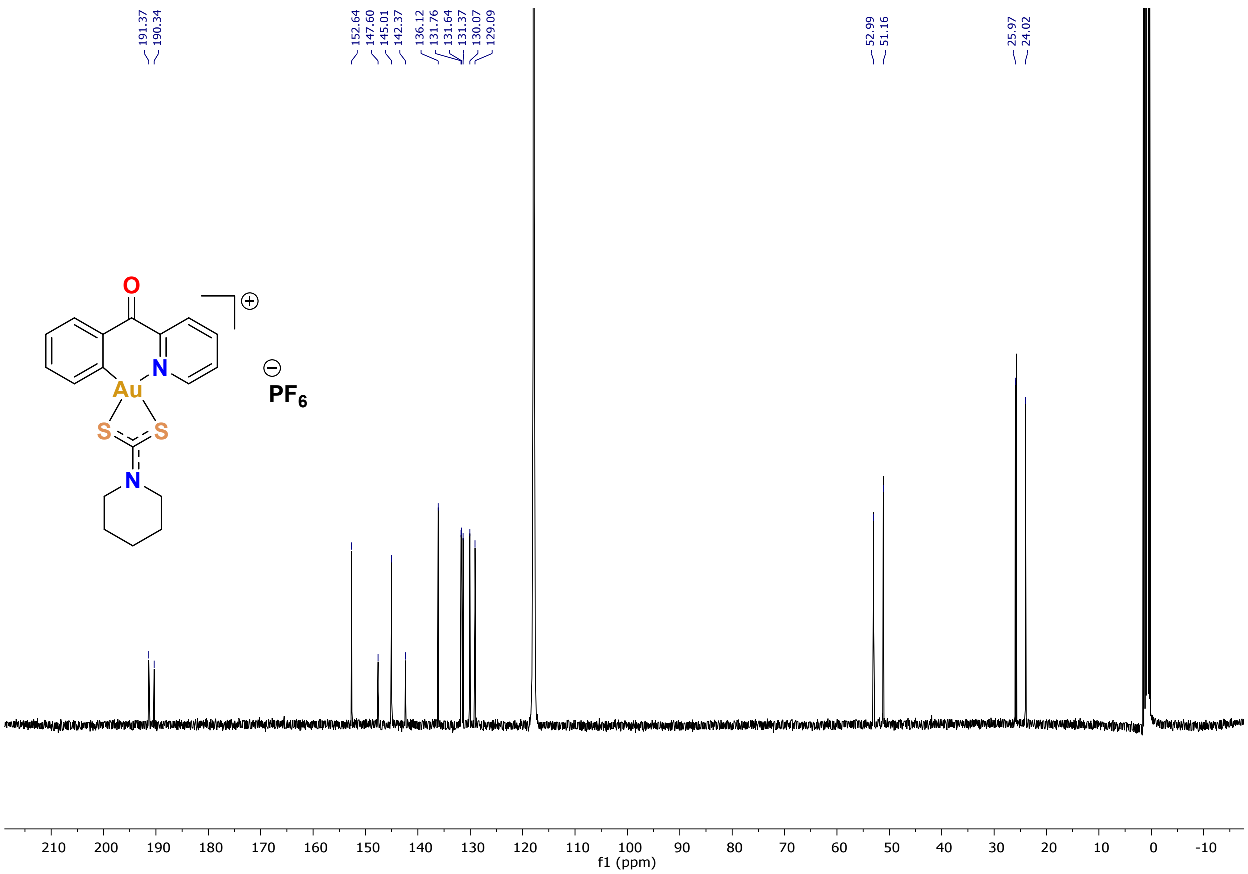

Fig. S12. ^{13}C NMR spectrum of **1c** in CD_3CN at 298K.

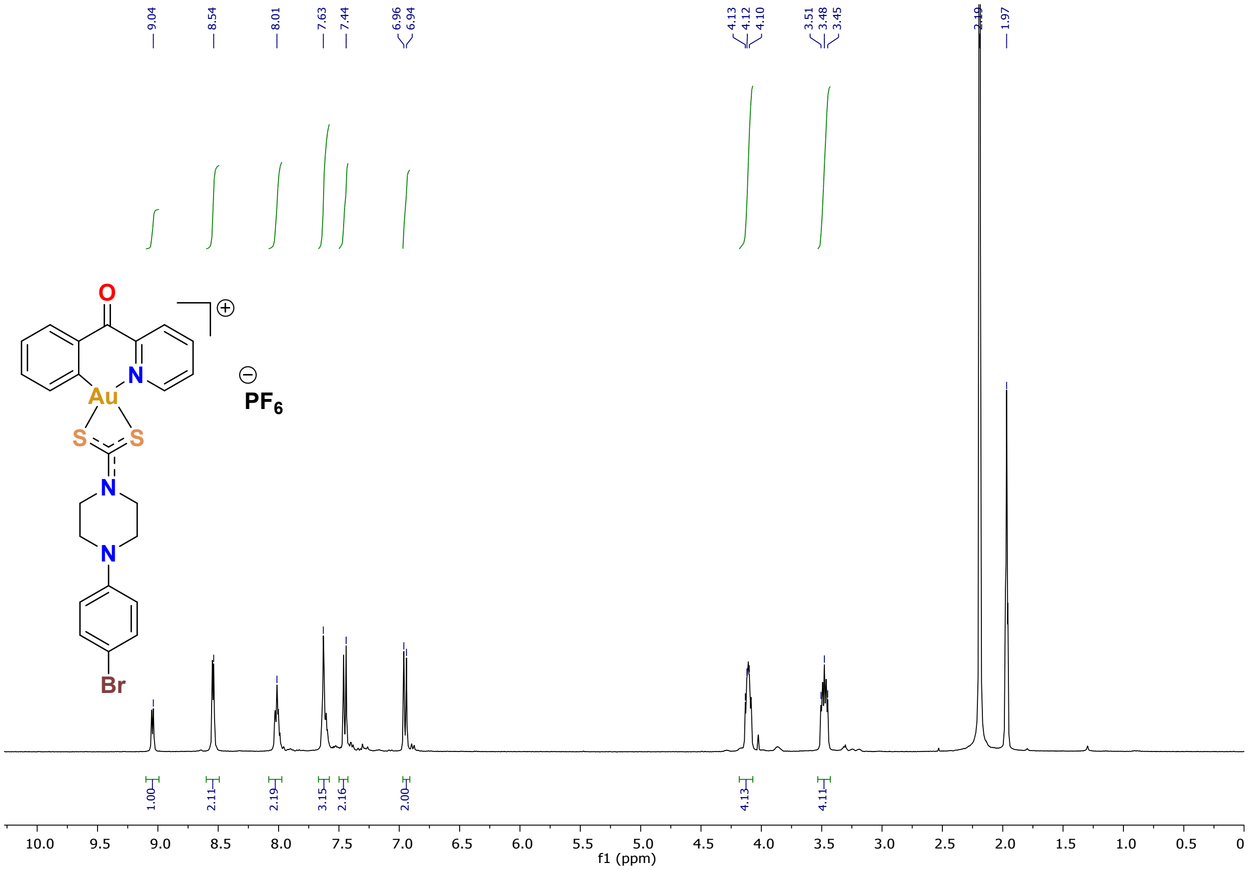

Fig. S13. ^{1}H NMR spectrum of **1d** in CD_3CN at 298K.

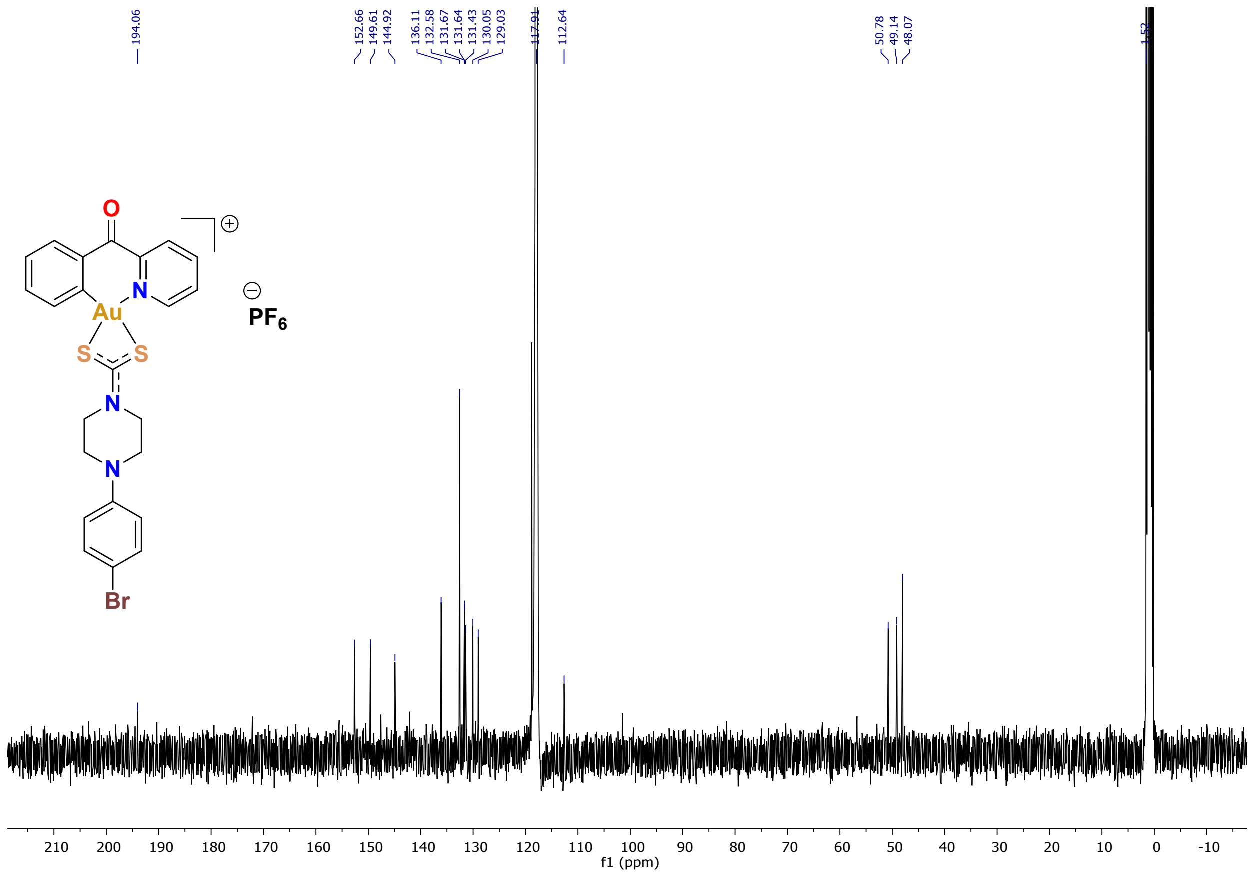

Fig. S14. ^{13}C NMR spectrum of **1d** in CD_3CN at 298K.

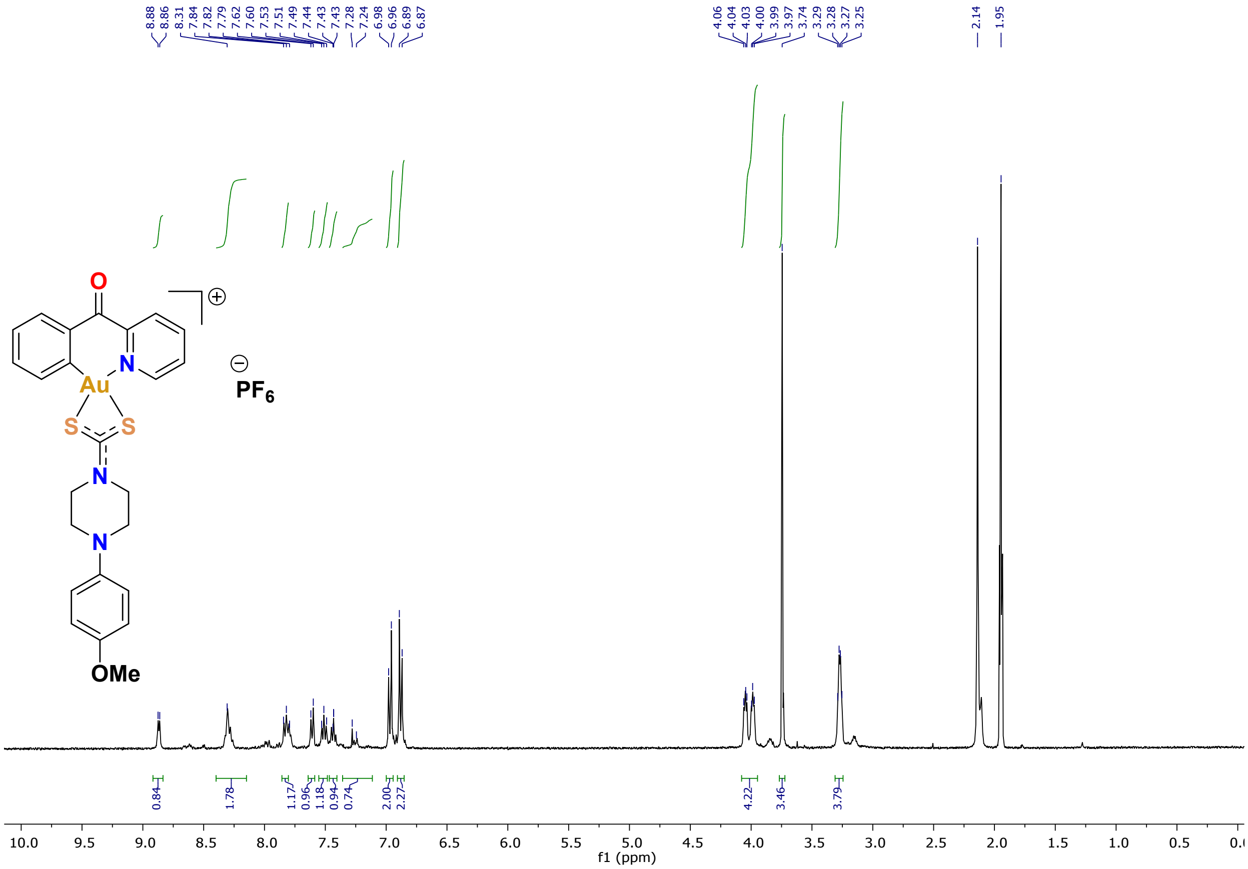

Fig. S15. ^{1}H NMR spectrum of **1e** in CD_3CN at 298K.

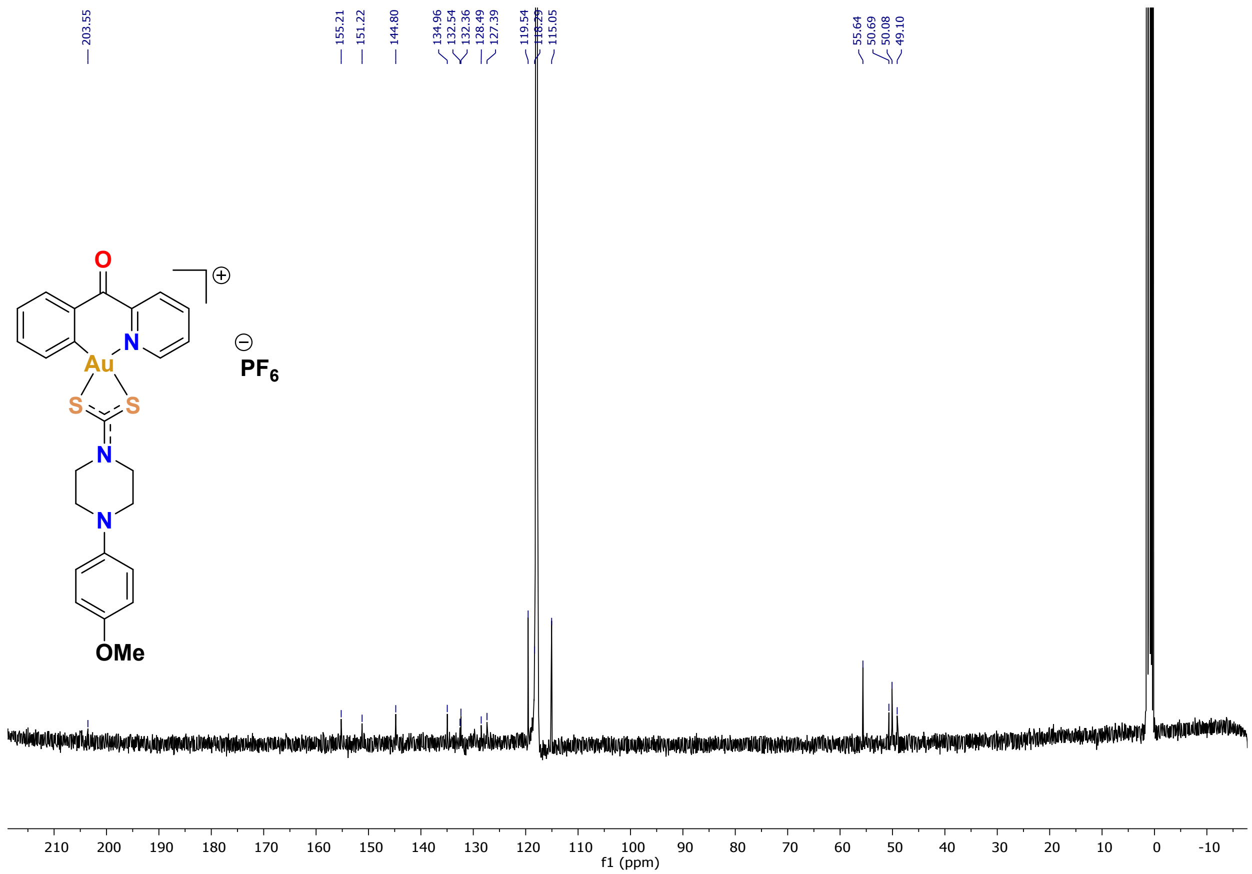

Fig. S16. ^{13}C NMR spectrum of **1e** in CD_3CN at 298K.

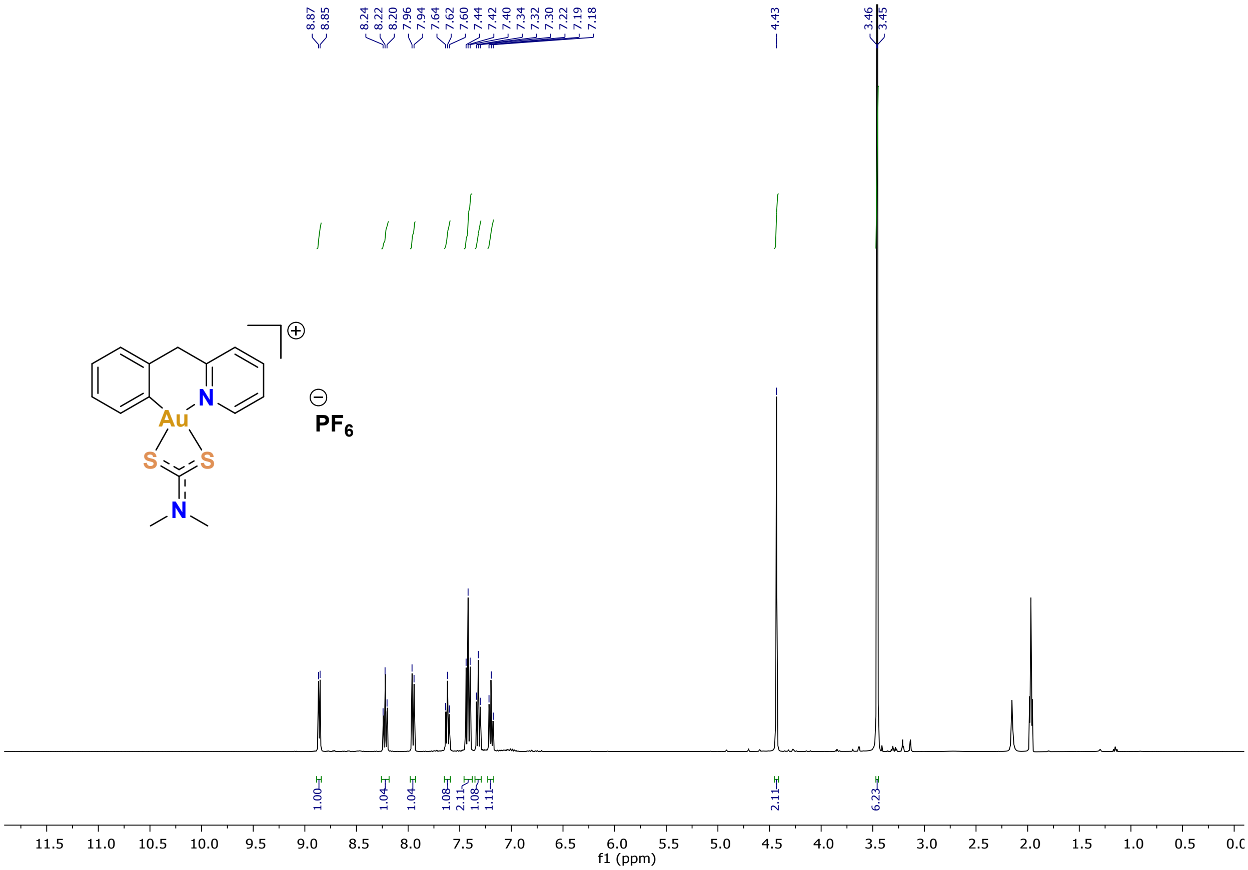

Fig. S17. ^{1}H NMR spectrum of **2a** in CD_3CN at 298K.

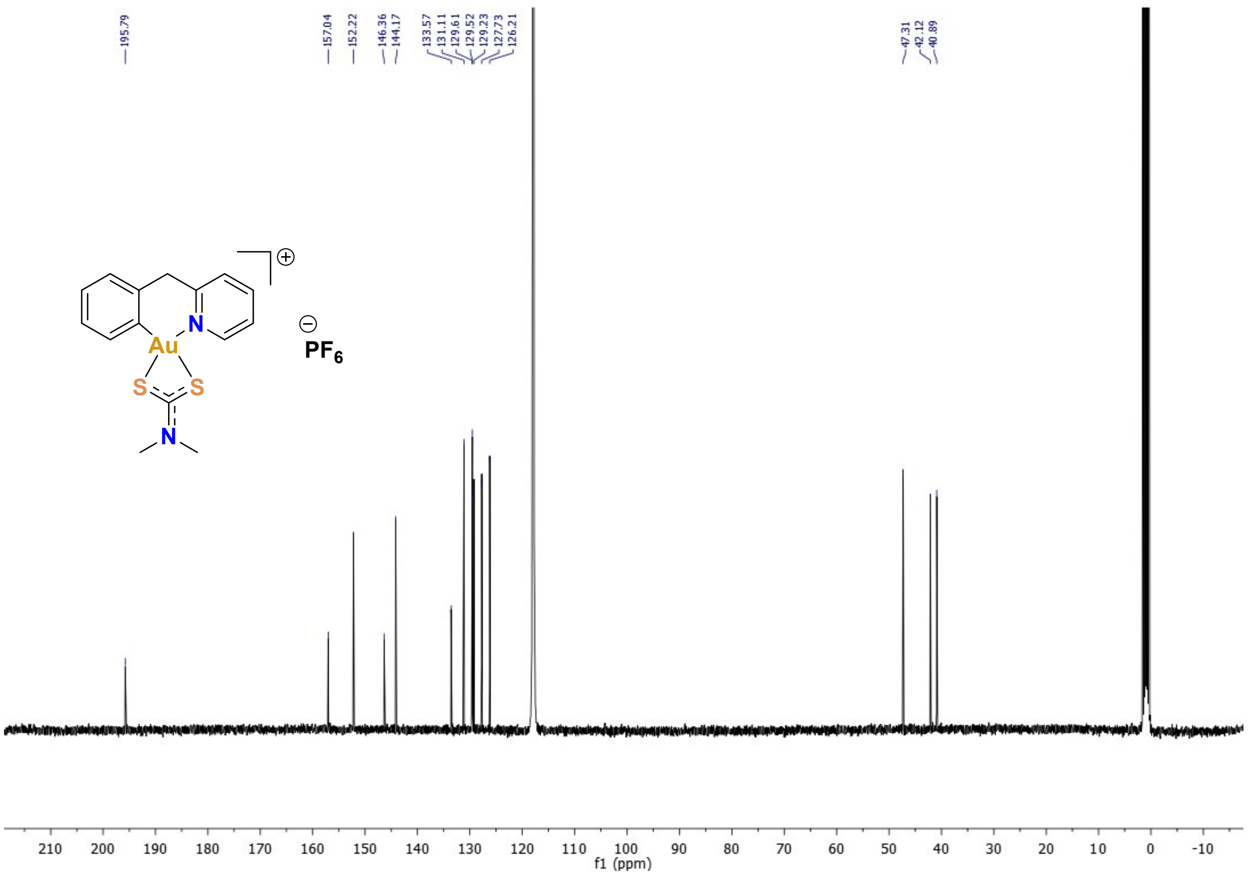

Fig. S18. ^{13}C NMR spectrum of **2a** in CD_3CN at 298K.

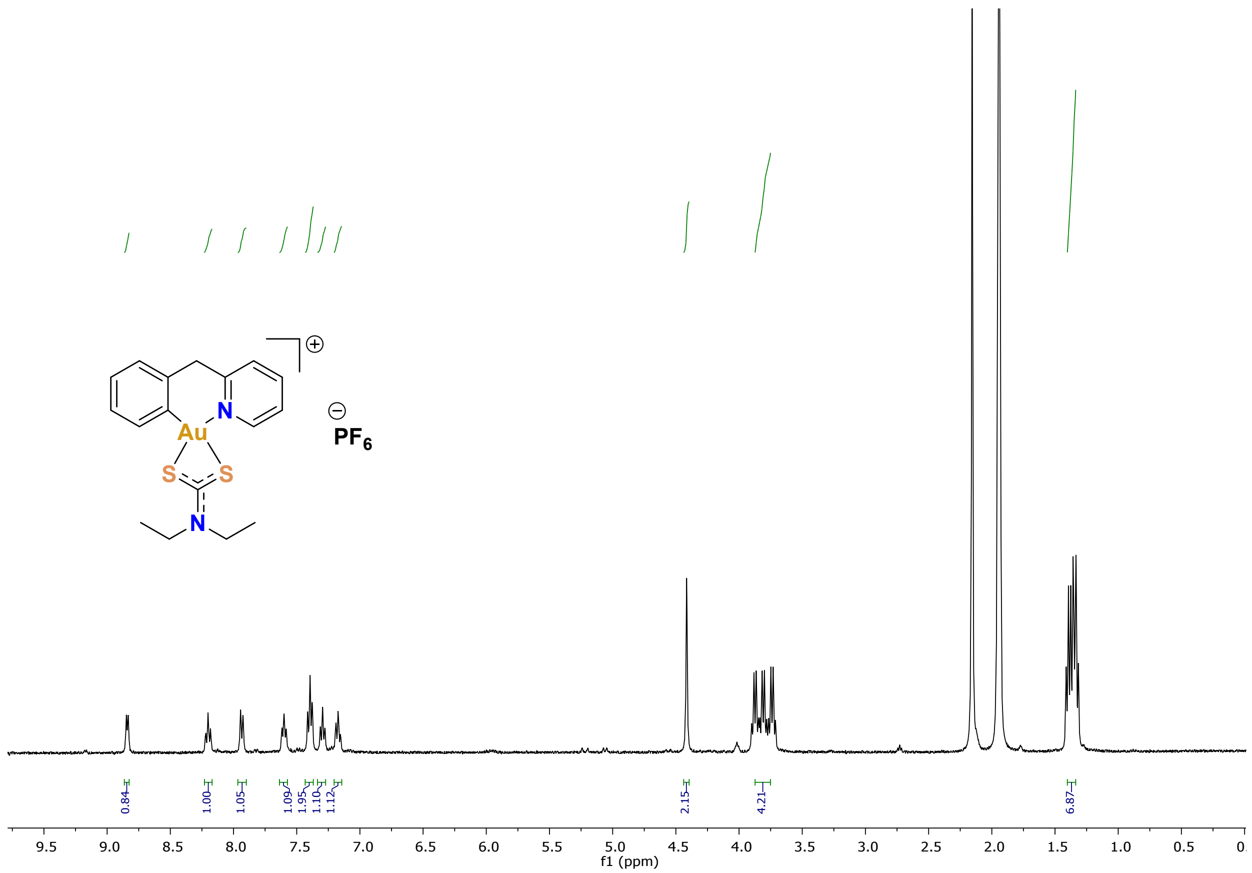

Fig. S19. ^{1}H NMR spectrum of **2b** in CD_3CN at 298K.

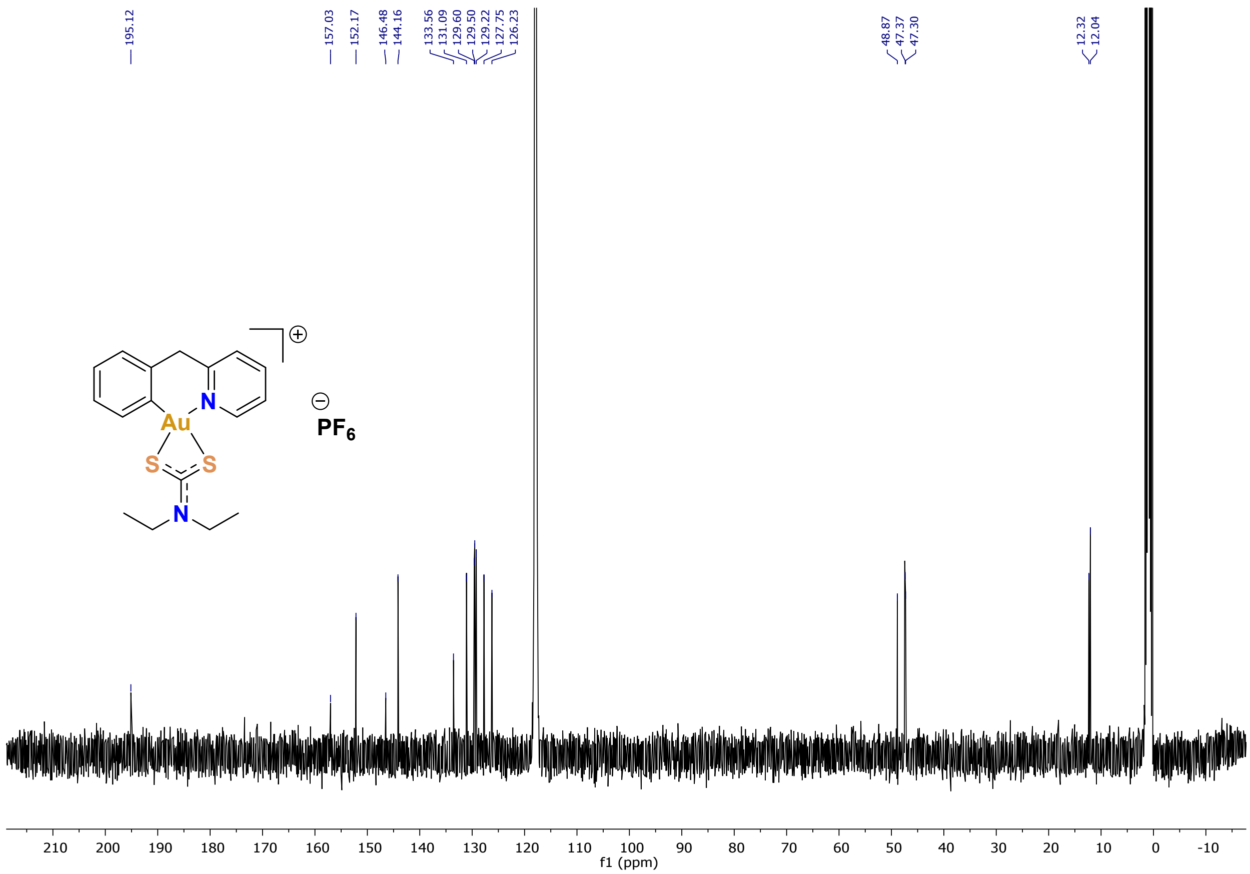

Fig. S20. ^{13}C NMR spectrum of **2b** in CD_3CN at 298K.

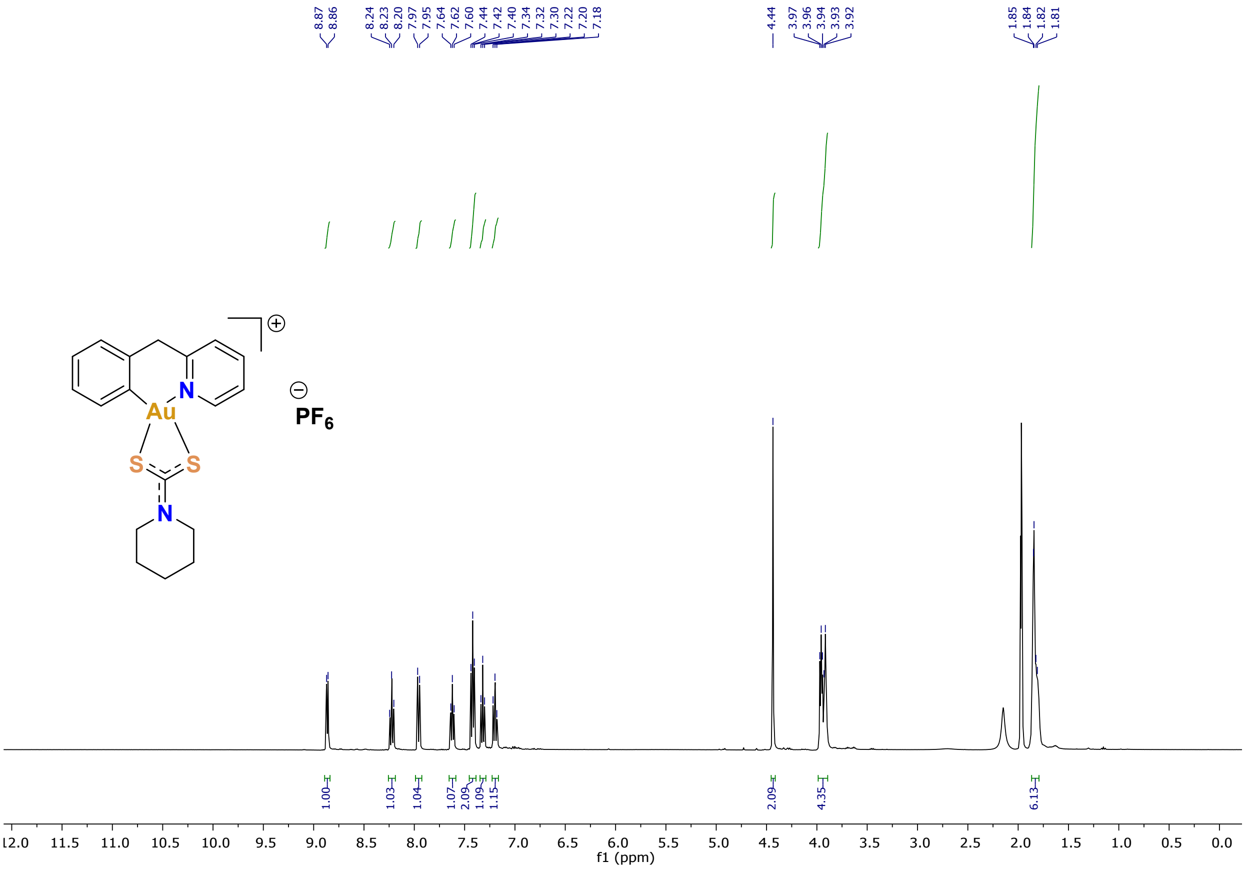

Fig. S21. ^{1}H NMR spectrum of **2c** in CD_3CN at 298K.

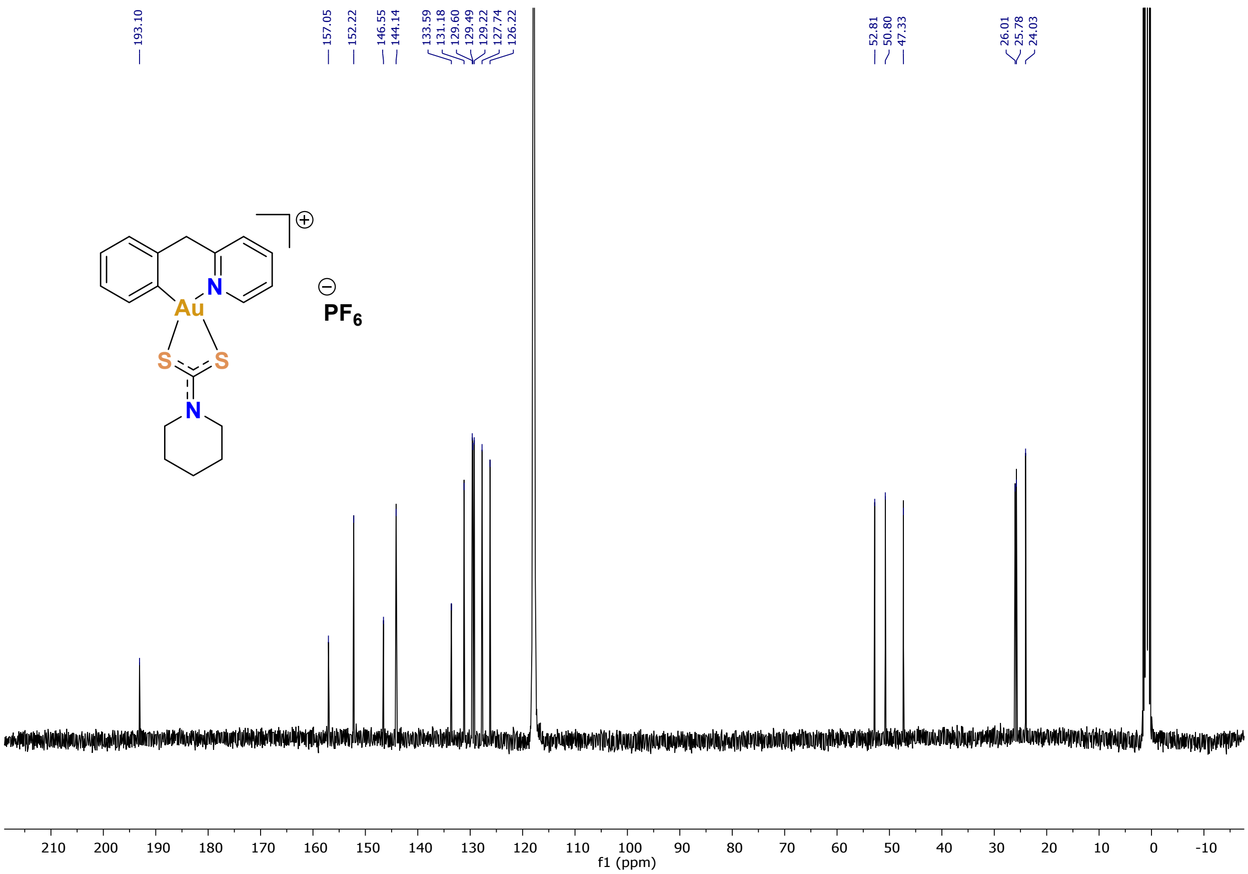

Fig. S22. ^{13}C NMR spectrum of **2c** in CD_3CN at 298K.

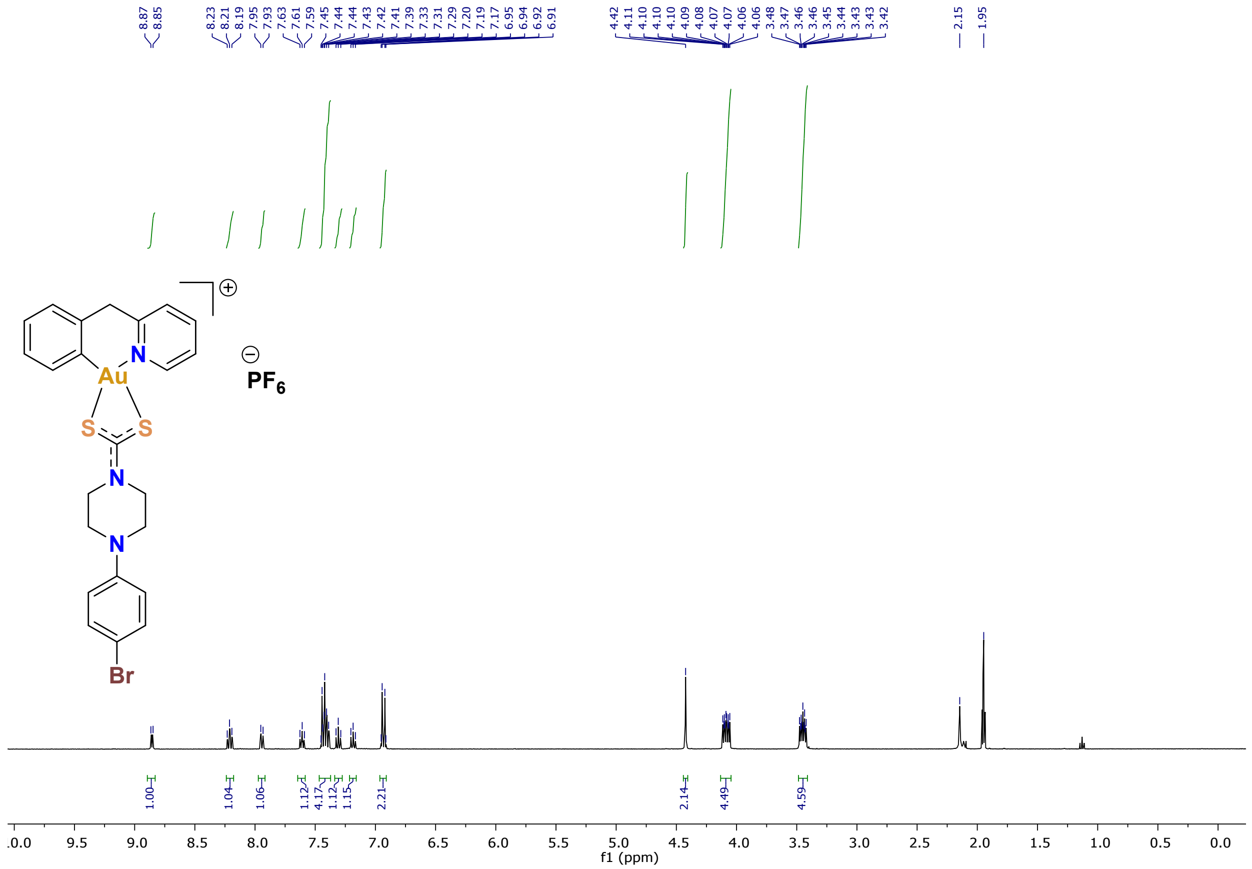

Fig. S23. ^{1}H NMR spectrum of **2d** in CD_3CN at 298K.

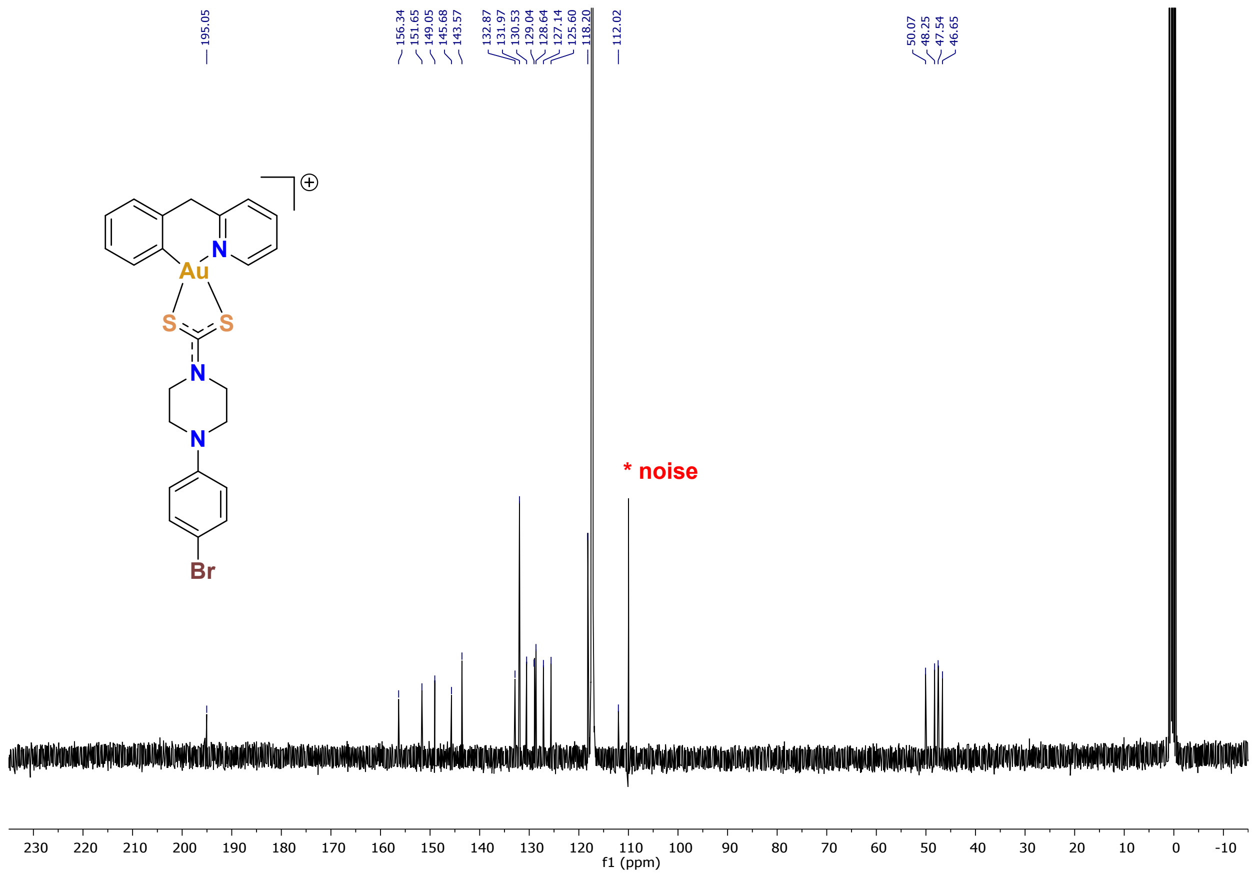

Fig. S24. ^{13}C NMR spectrum of **2d** in CD_3CN at 298K.

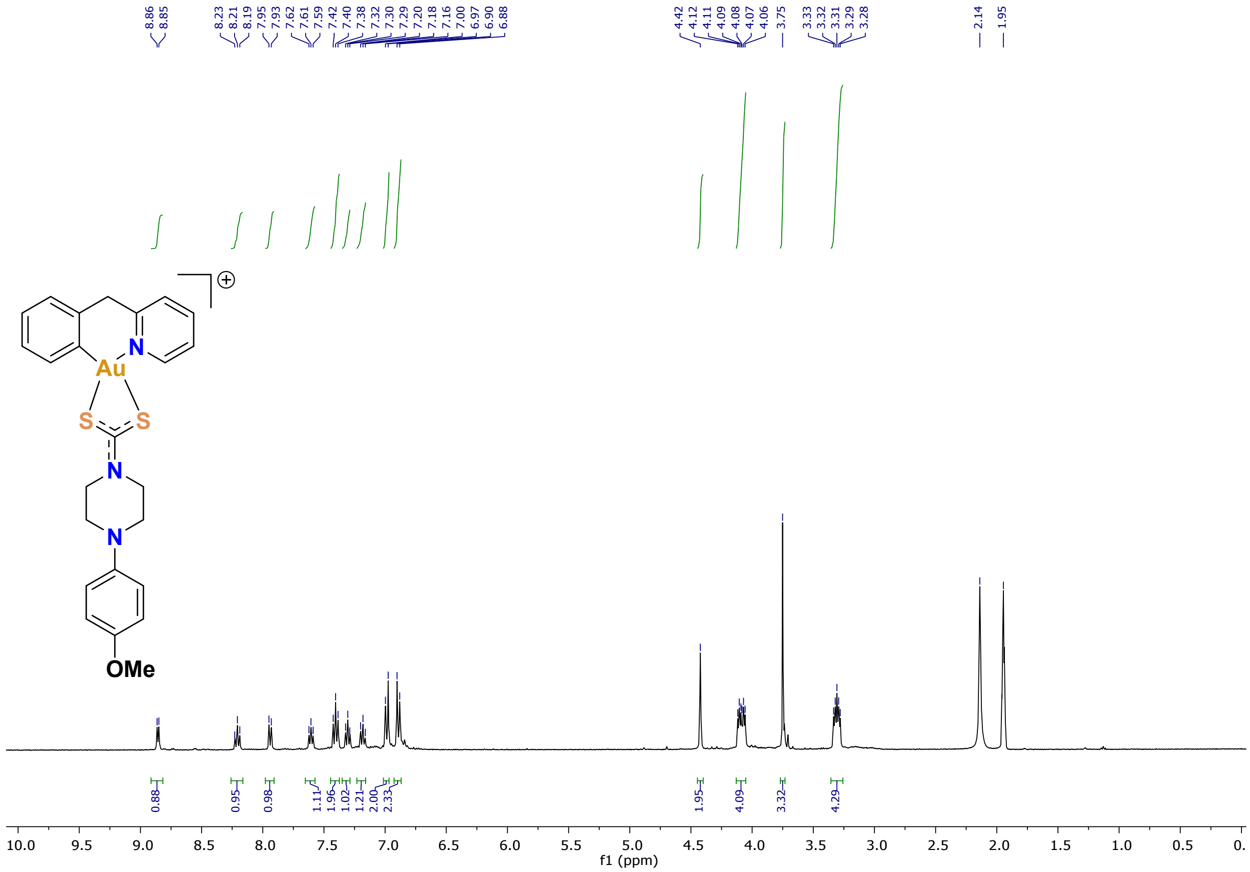

Fig. S25. ^{1}H NMR spectrum of **2e** in CD_3CN at 298K.

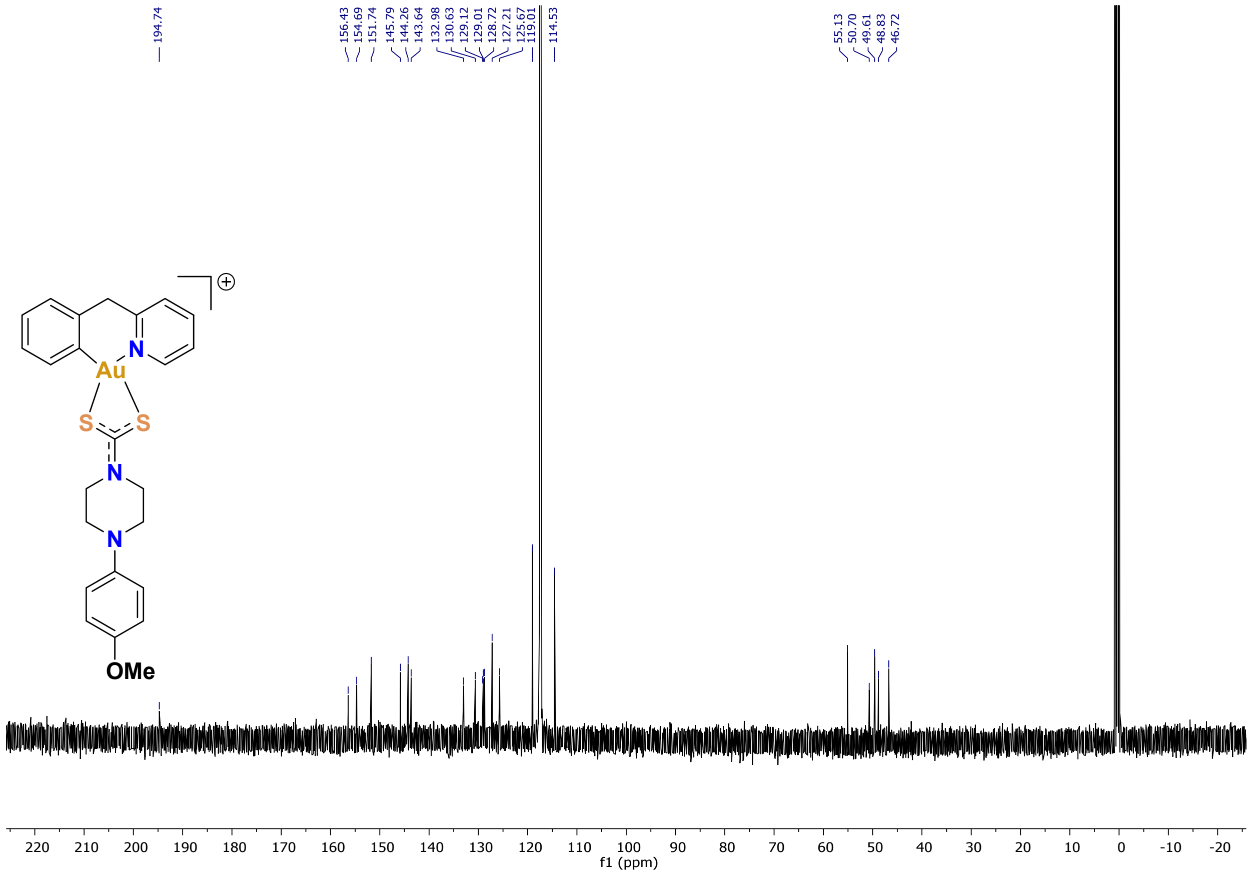

Fig. S26. ^{13}C NMR spectrum of **2e** in CD_3CN at 298K.

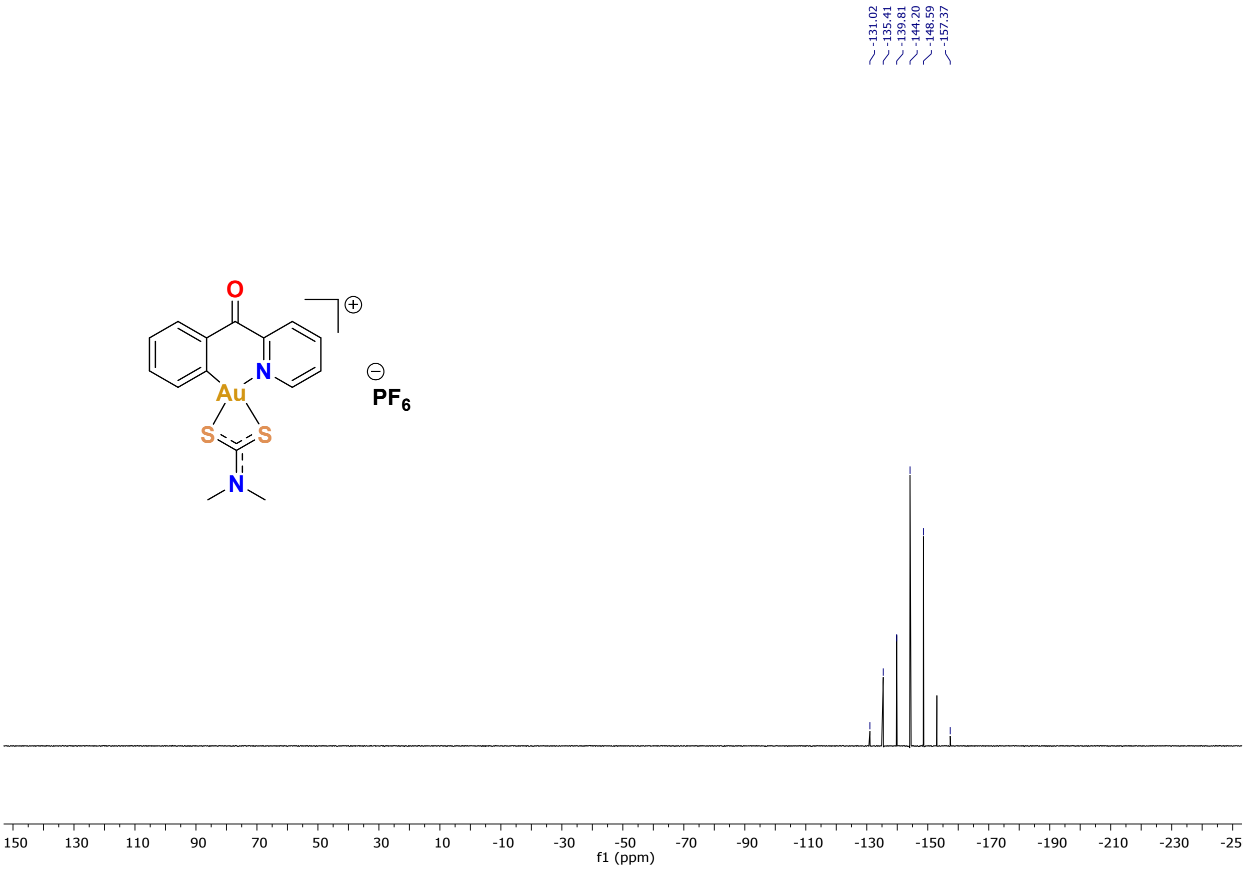

Fig. S27. ^{19}F NMR spectrum of **1a** in CD_3CN at 298K.

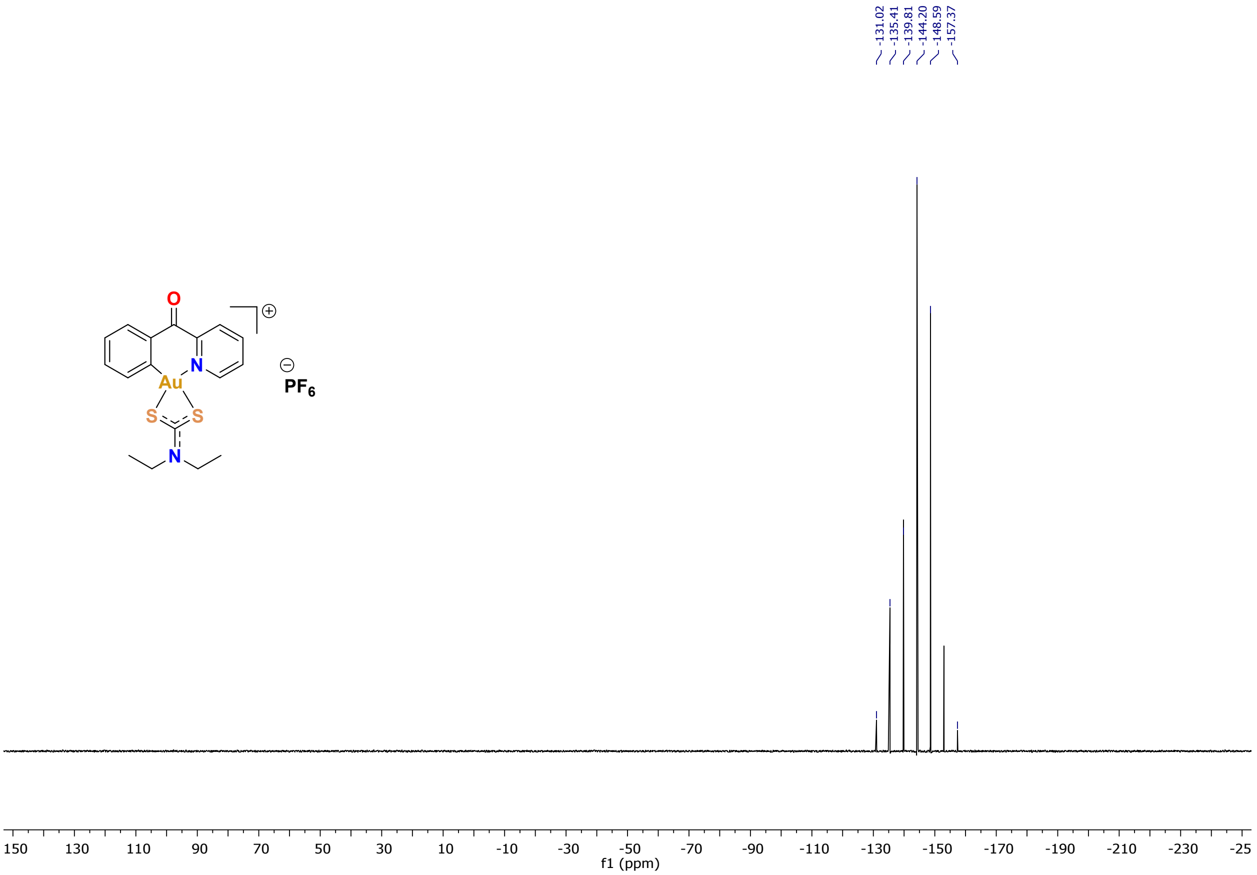

Fig. S28. ^{19}F NMR spectrum of **1b** in CD_3CN at 298K.

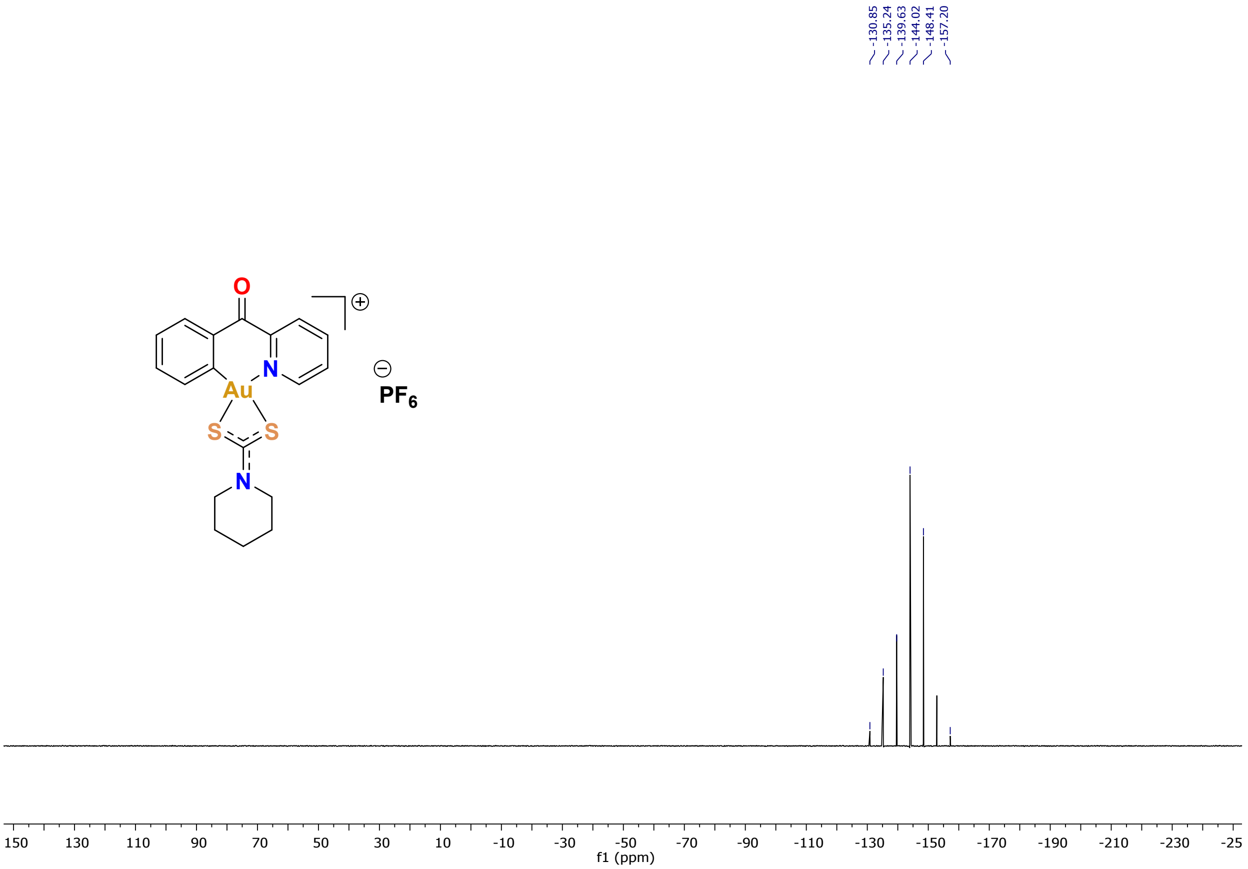

Fig. S29. ^{19}F NMR spectrum of **1c** in CD_3CN at 298K.

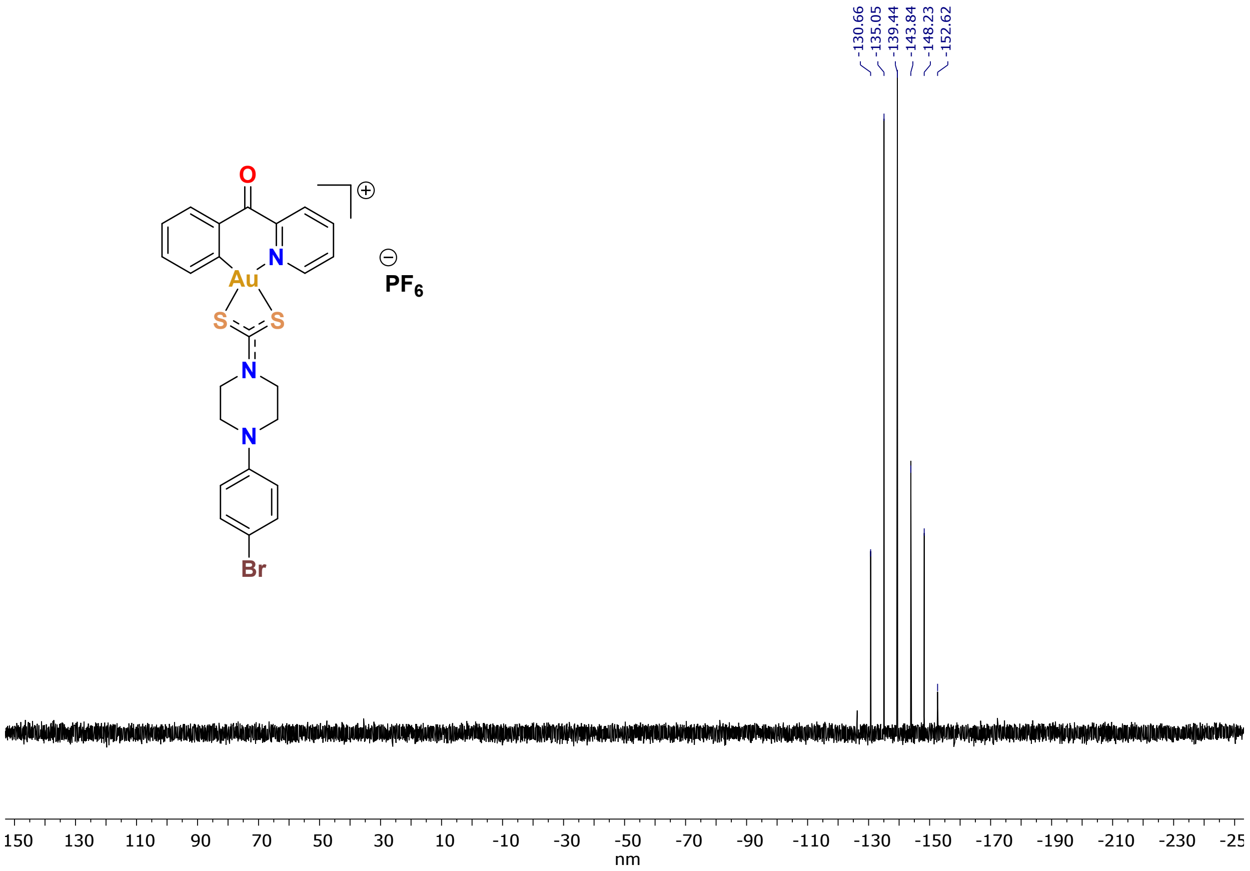

Fig. S30. ^{19}F NMR spectrum of **1d** in CD_3CN at 298K.

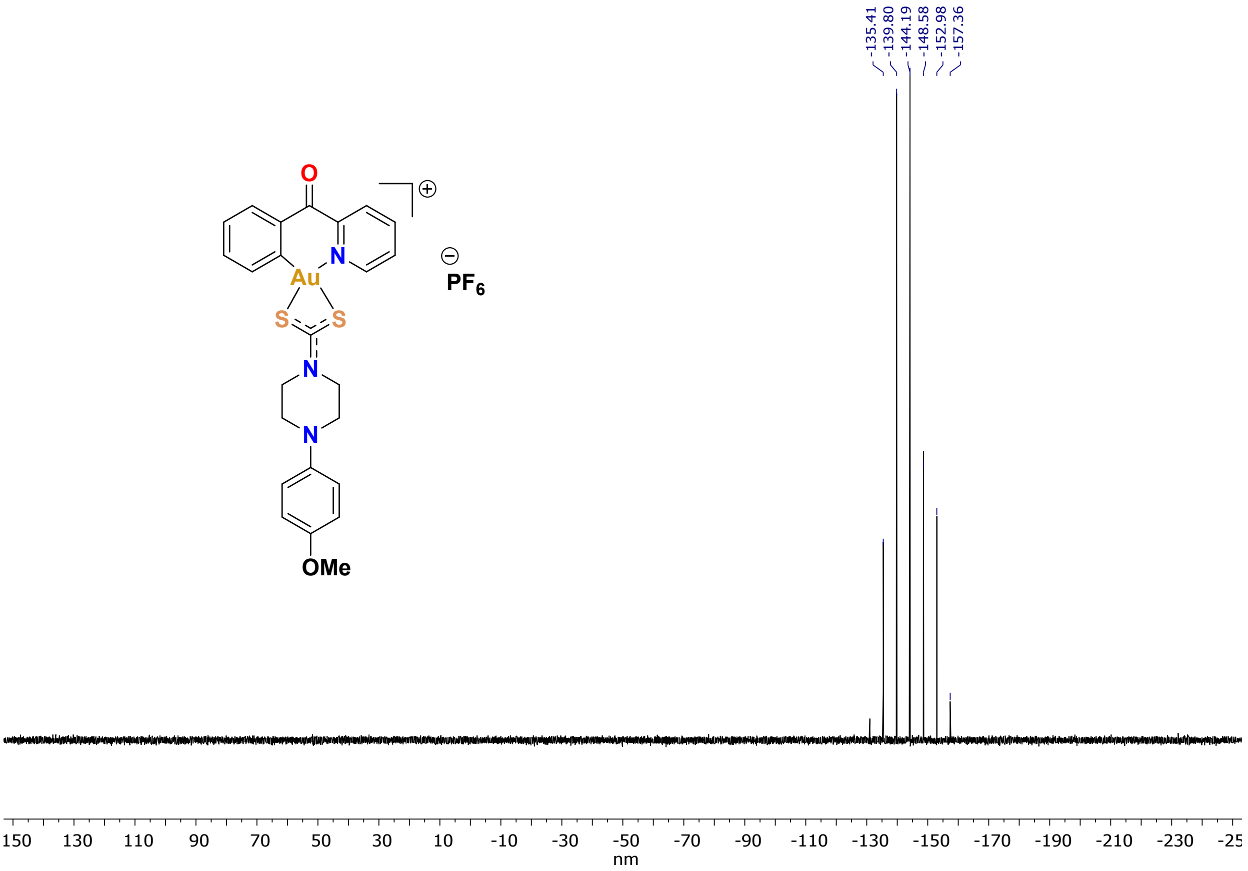

Fig. S31. ^{19}F NMR spectrum of **1e** in CD_3CN at 298K.

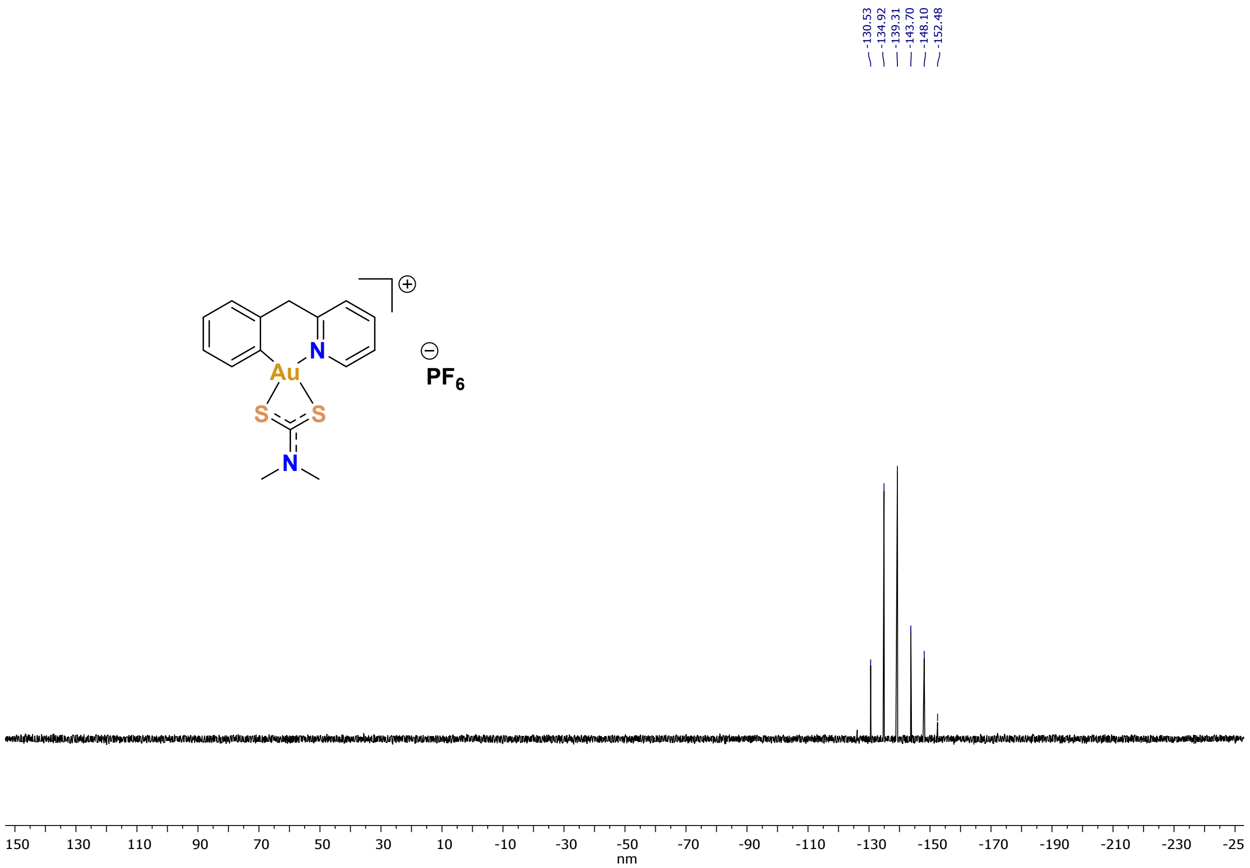

Fig. S32. ^{19}F NMR spectrum of **2a** in CD_3CN at 298K.

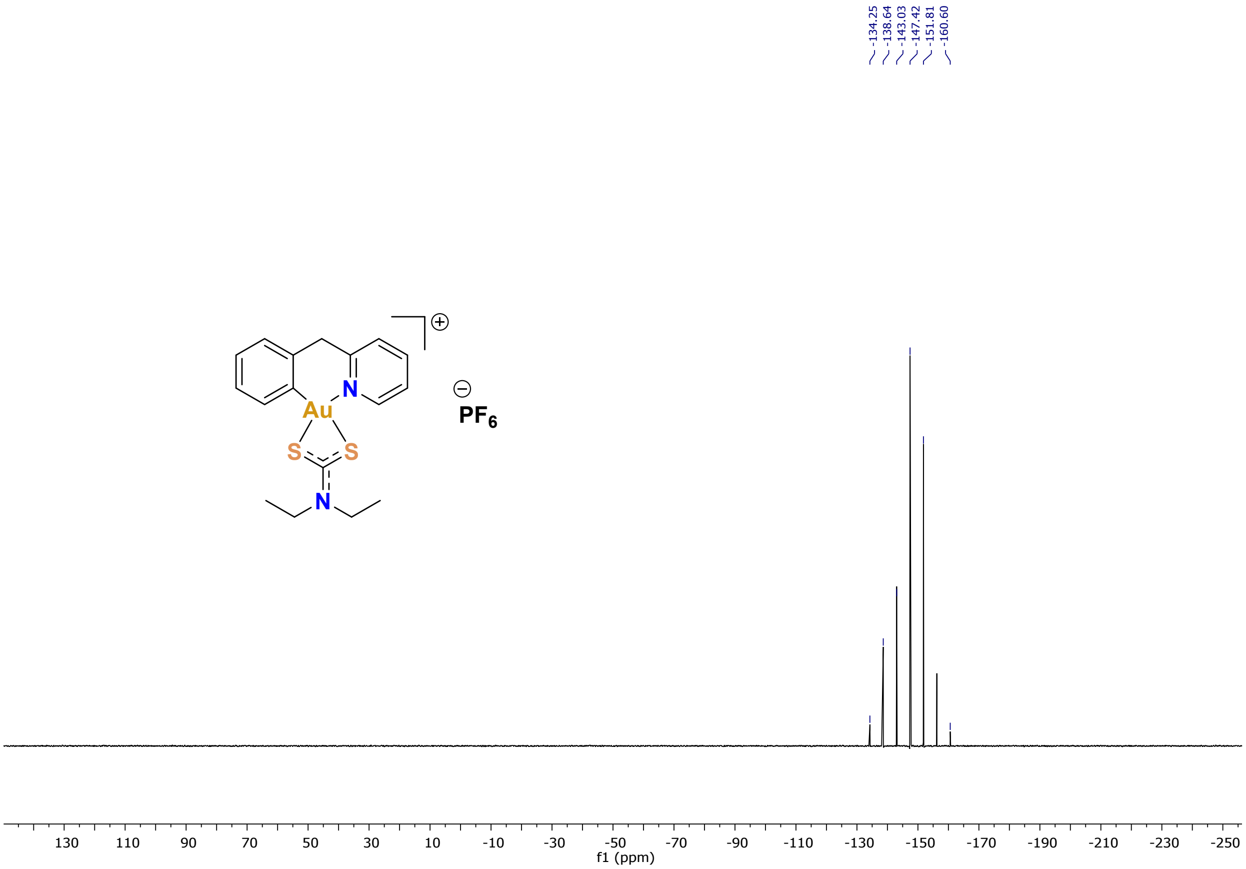

Fig. S33. ^{19}F NMR spectrum of **2b** in CD_3CN at 298K.

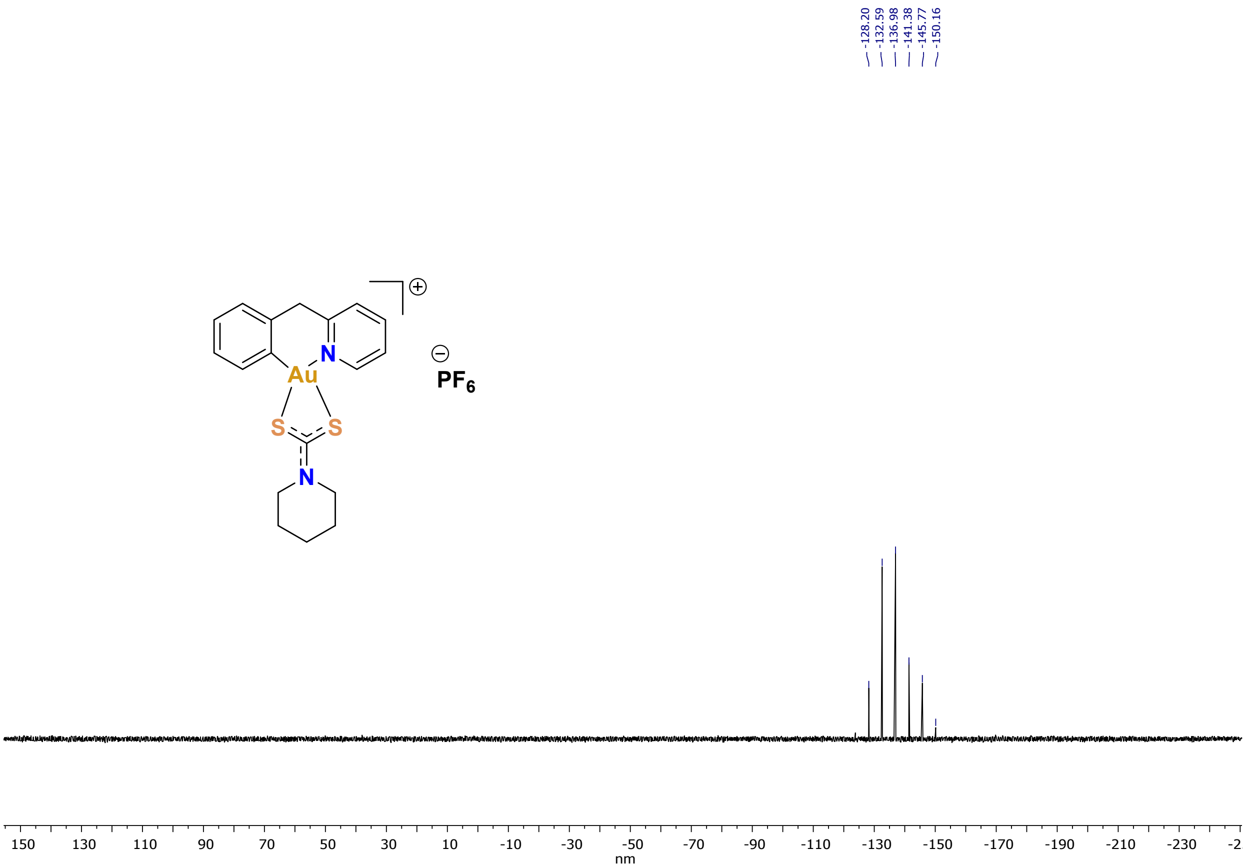

Fig. S34. ^{19}F NMR spectrum of **2c** in CD_3CN at 298K.

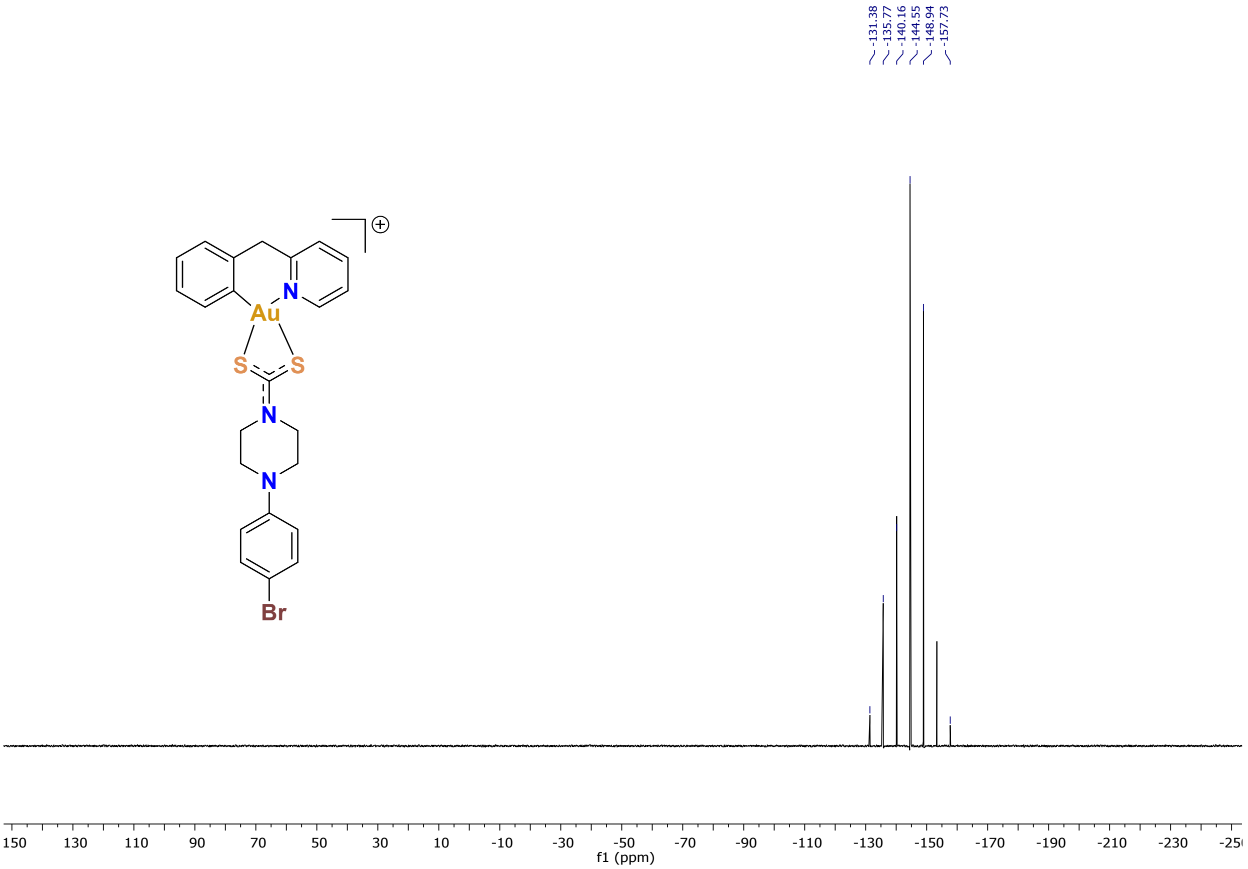

Fig. S35. ^{19}F NMR spectrum of **2d** in CD_3CN at 298K.

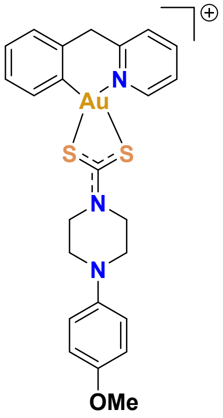

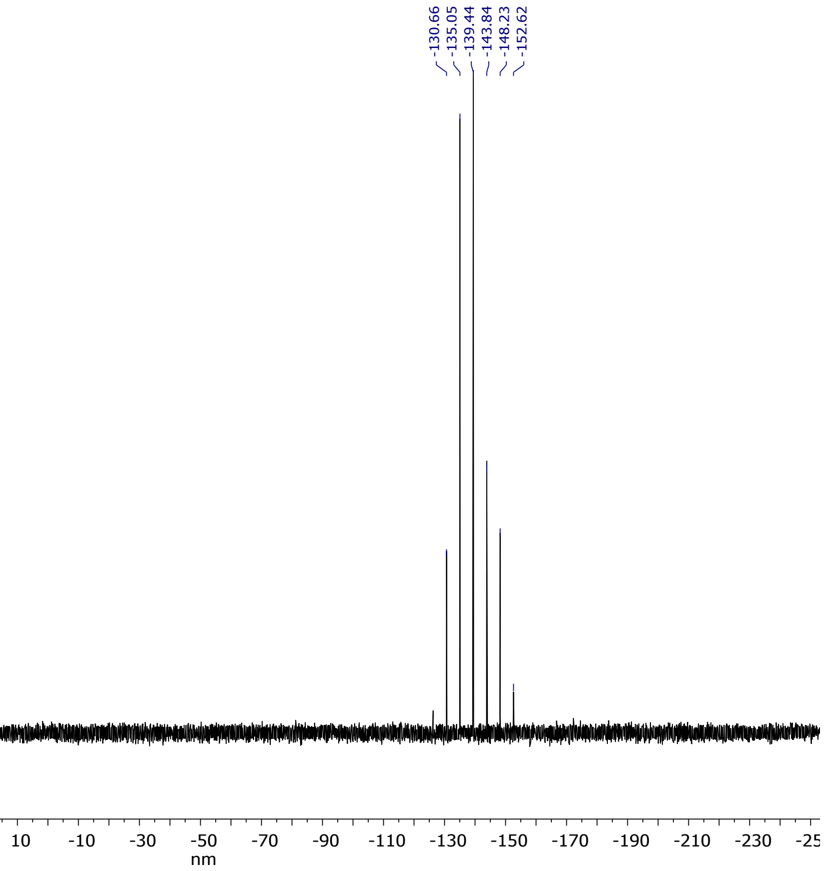

Fig. S36. ^{19}F NMR spectrum of **2e** in CD_3CN at 298K.

HRMS of 1a-1e and 2a-2e:

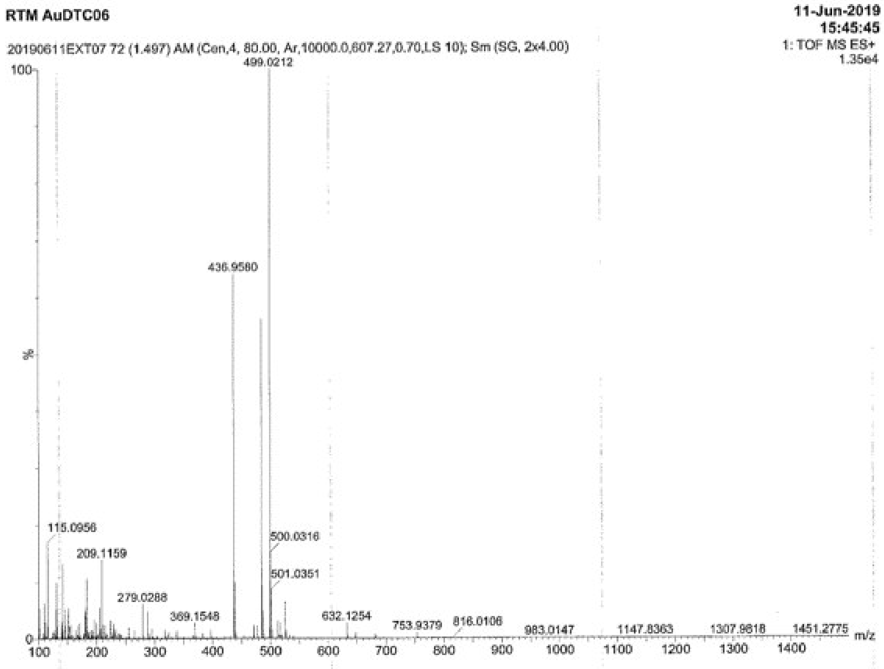

Fig. S37. HRMS of **1a** $[M - PF_6]^+$.

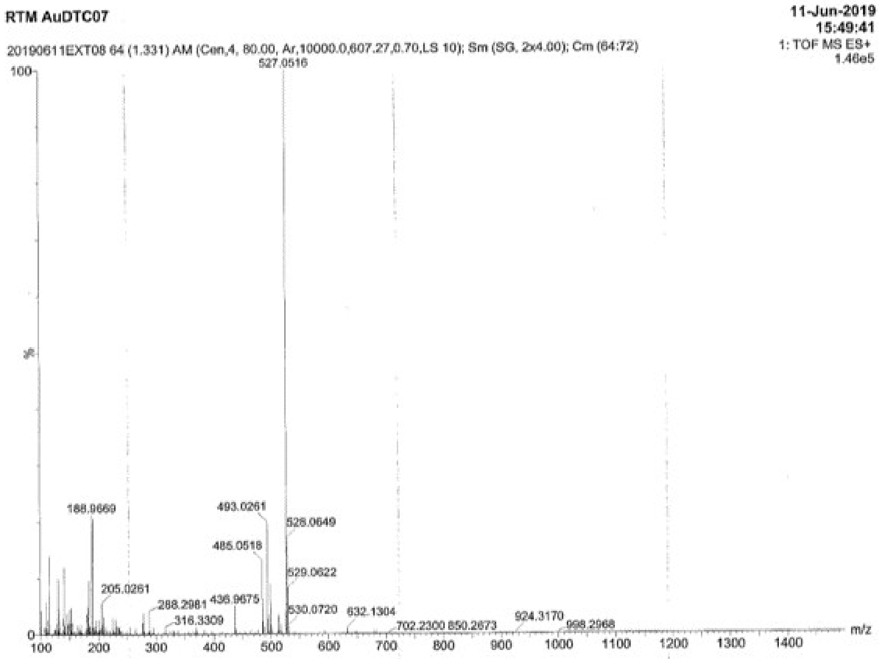

Fig. S38. HRMS of **1b** $[M - PF_6]^+$.

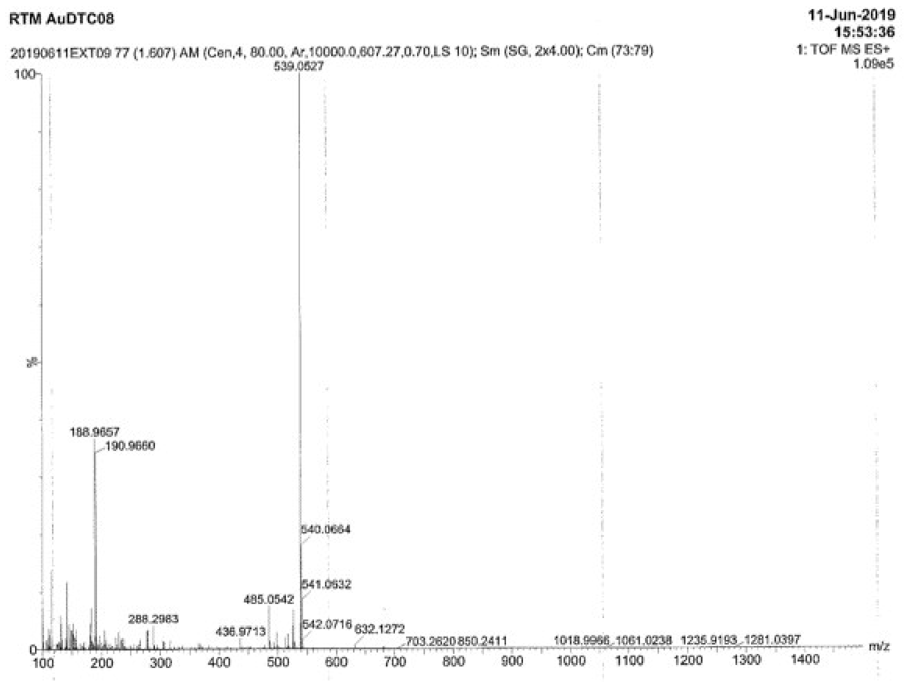

Fig. S39. HRMS of **1c** [M – PF_6]$^+$.

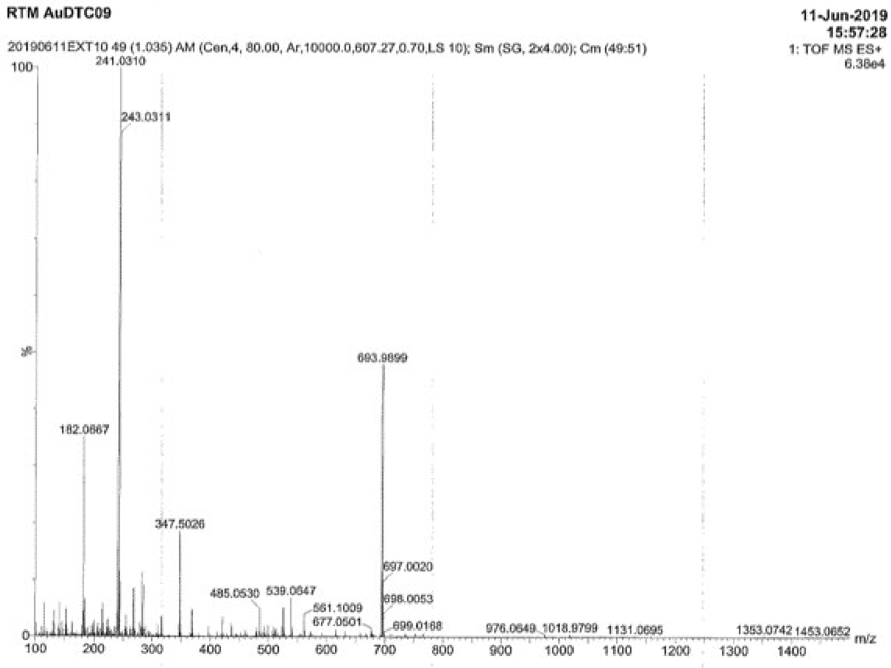

Fig. S40. HRMS of **1d** [M – PF_6]$^+$.

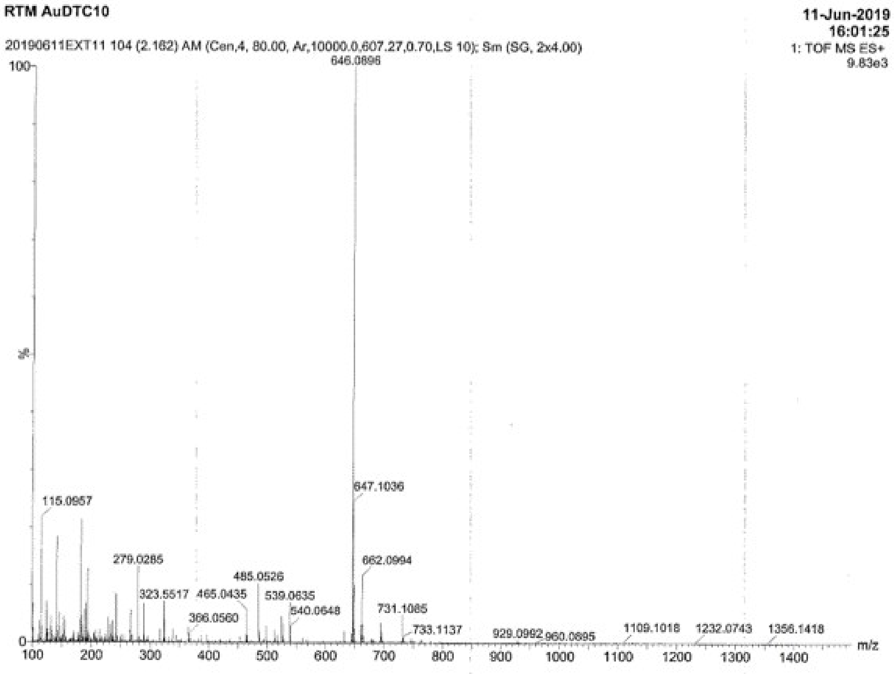

Fig. S41. HRMS of **1e** $[M - PF_6]^+$.

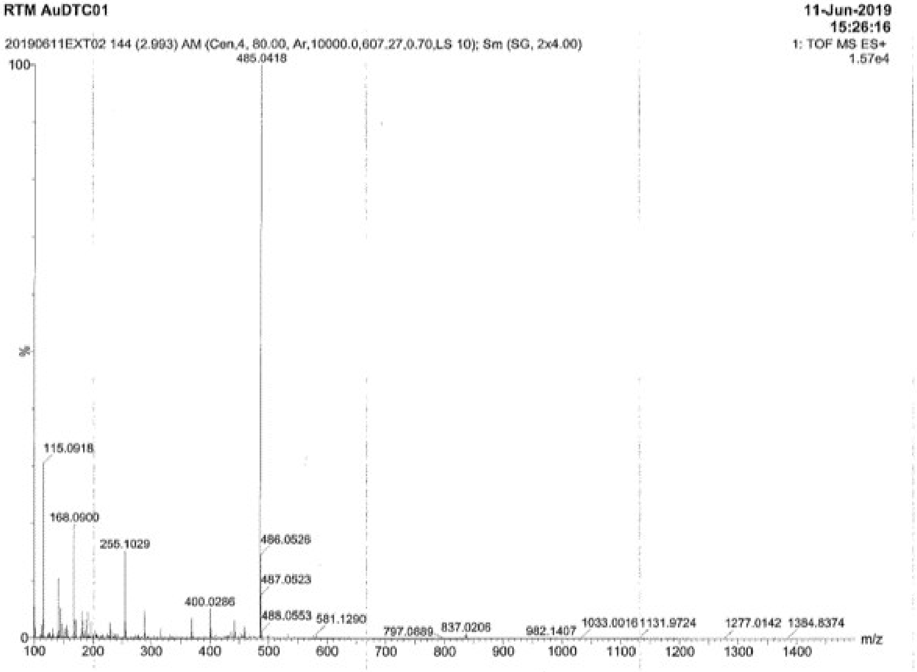

Fig. S42. HRMS of **2a** $[M - PF_6]^+$.

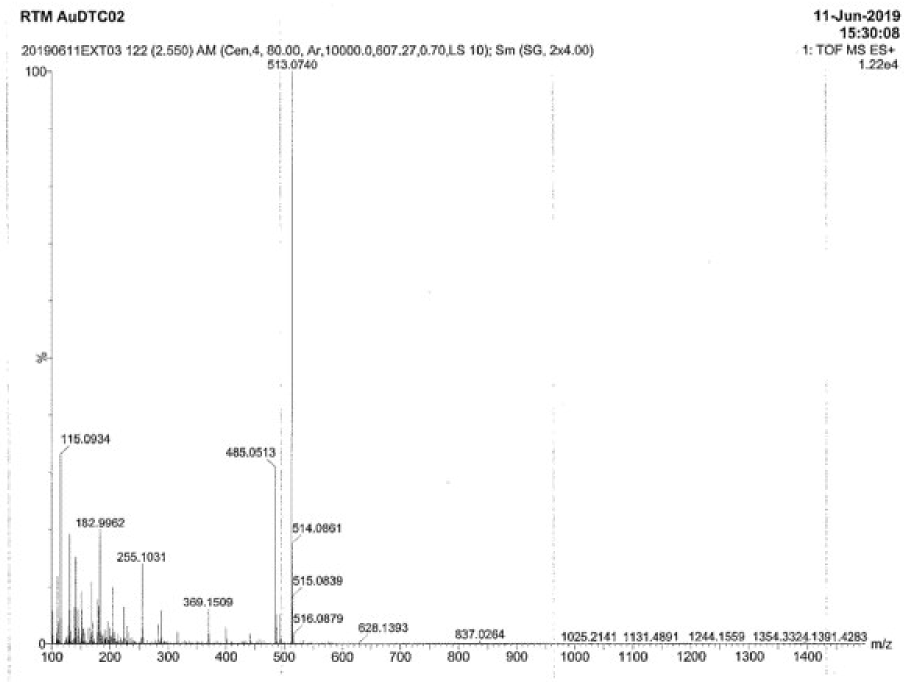

Fig. S43. HRMS of **2b** $[M - PF_6]^+$.

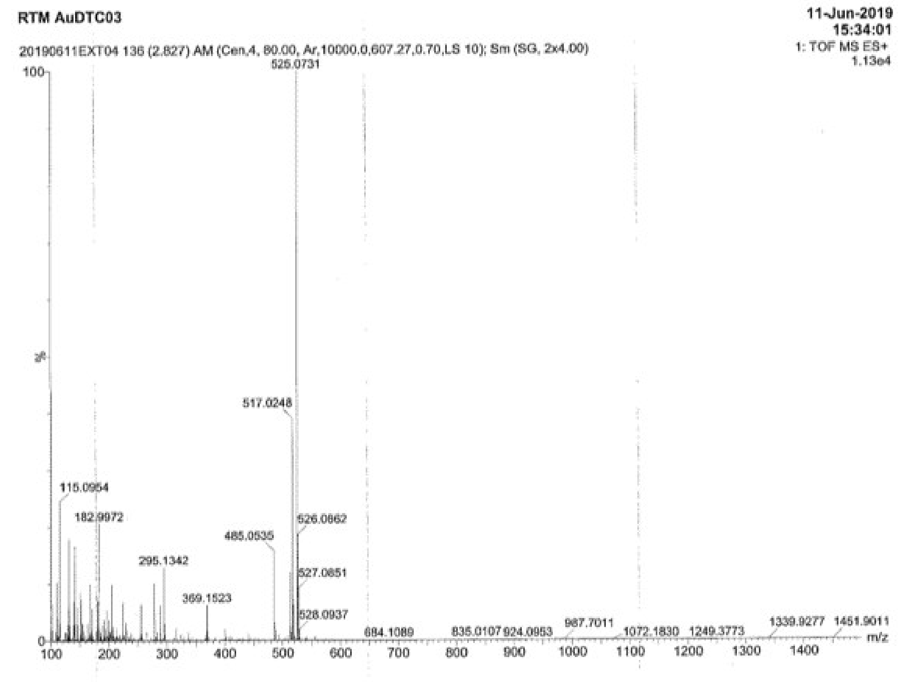

Fig. S44. HRMS of **2c** $[M - PF_6]^+$.

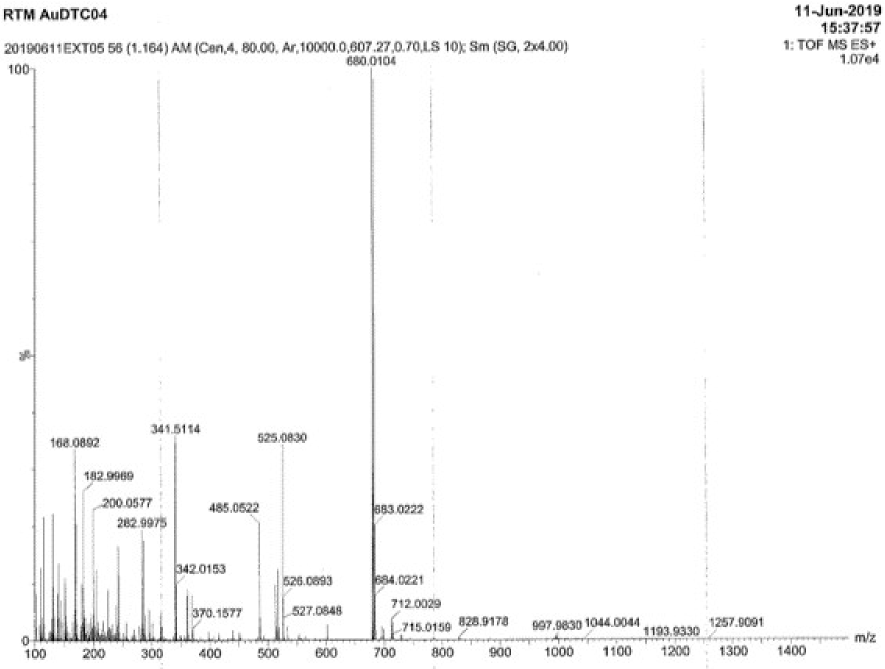

Fig. S45. HRMS of **2d** $[M - PF_6]^+$.

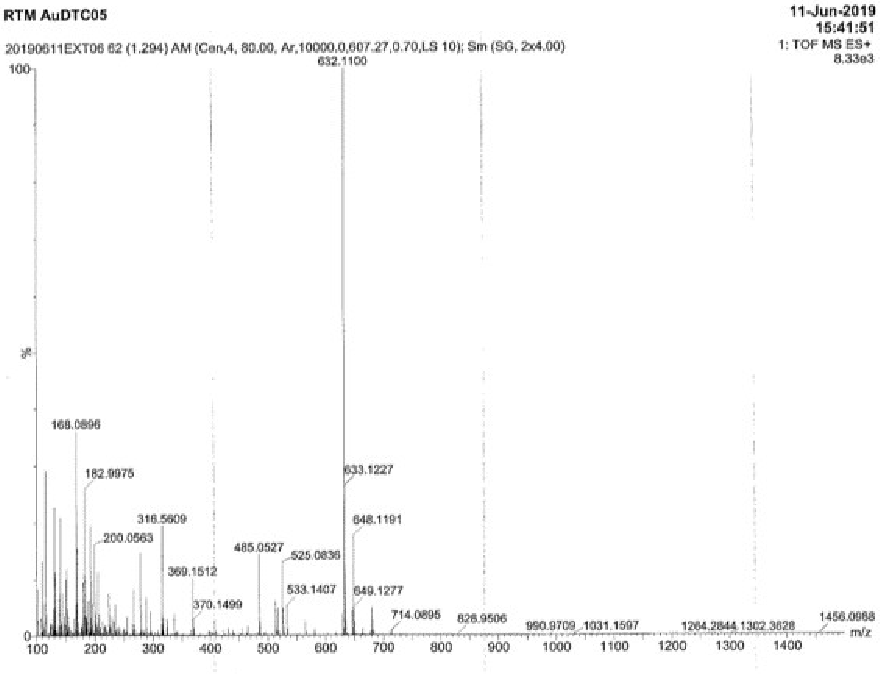

Fig. S46. HRMS of **2e** $[M - PF_6]^+$.

HPLC Trace of 1a-1e and 2a-2e:

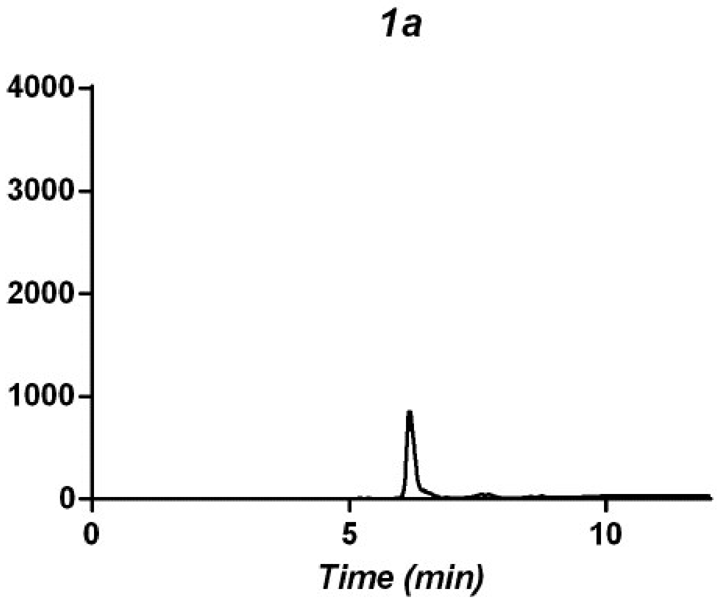

Fig. S47. HPLC chromatogram of **1a,** (λ = 280 nm).

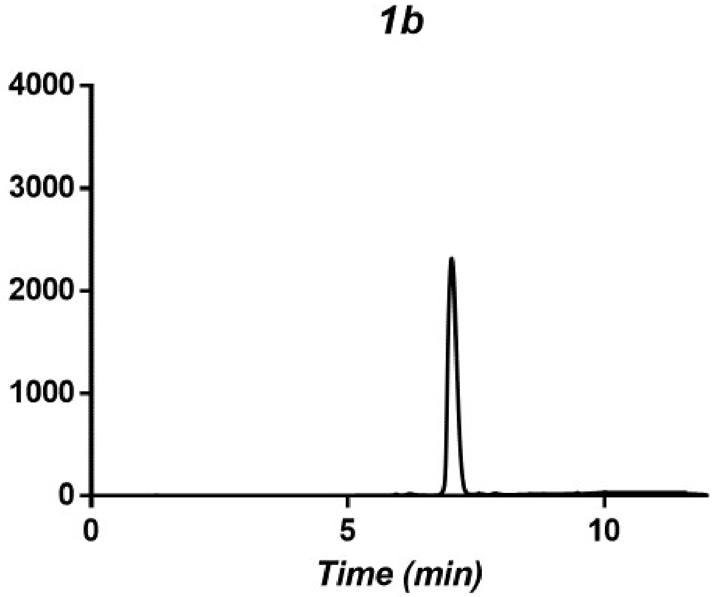

Fig. S48. HPLC chromatogram of **1b,** (λ = 280 nm).

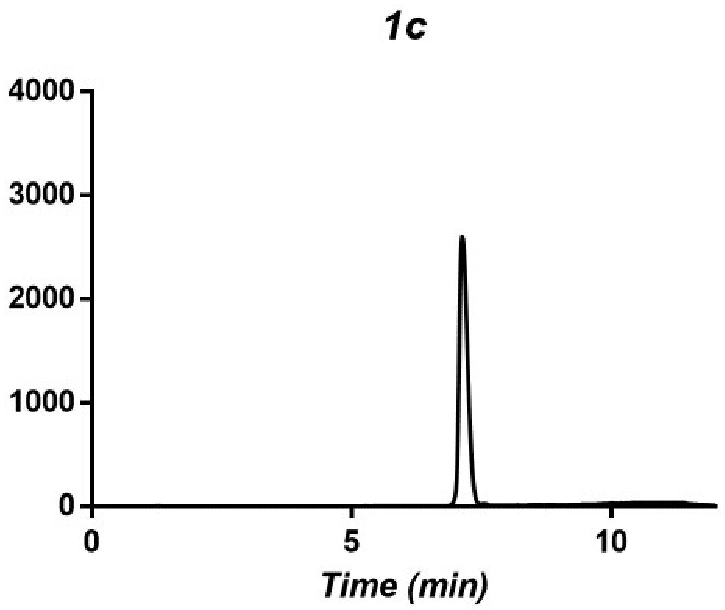

Fig. S49. HPLC chromatogram of **1c,** (λ = 280 nm).

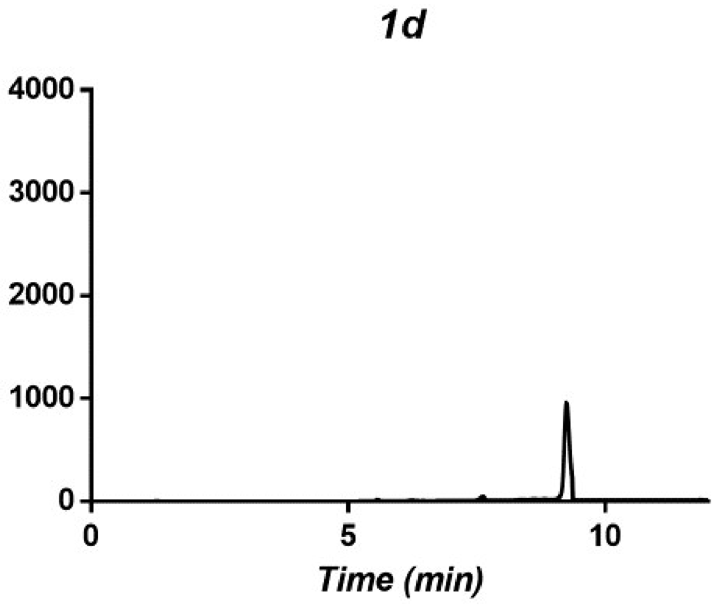

Fig. S50. HPLC chromatogram of **1d,** (λ = 280 nm).

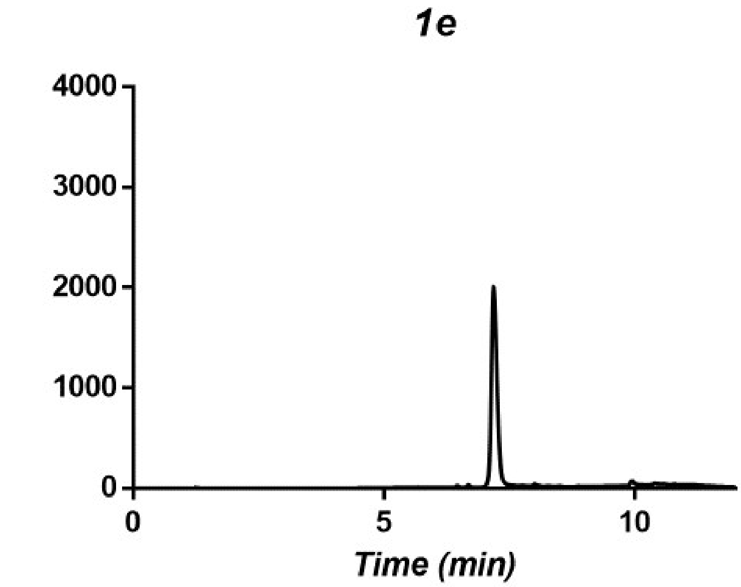

Fig. S51. HPLC chromatogram of **1e,** (λ = 280 nm).

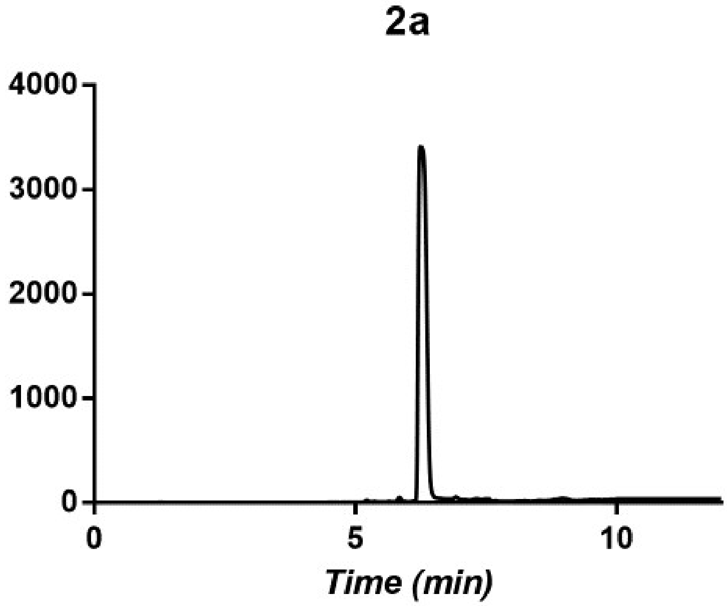

Fig. S52. HPLC chromatogram of **2a,** (λ = 280 nm).

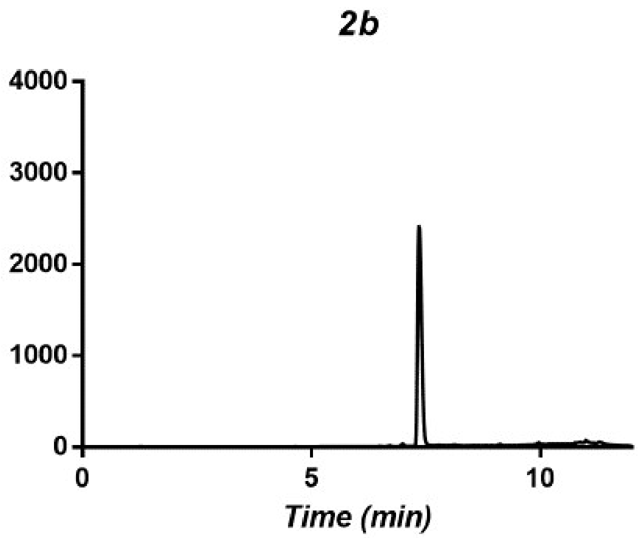

Fig. S53. HPLC chromatogram of **2b,** (λ = 280 nm).

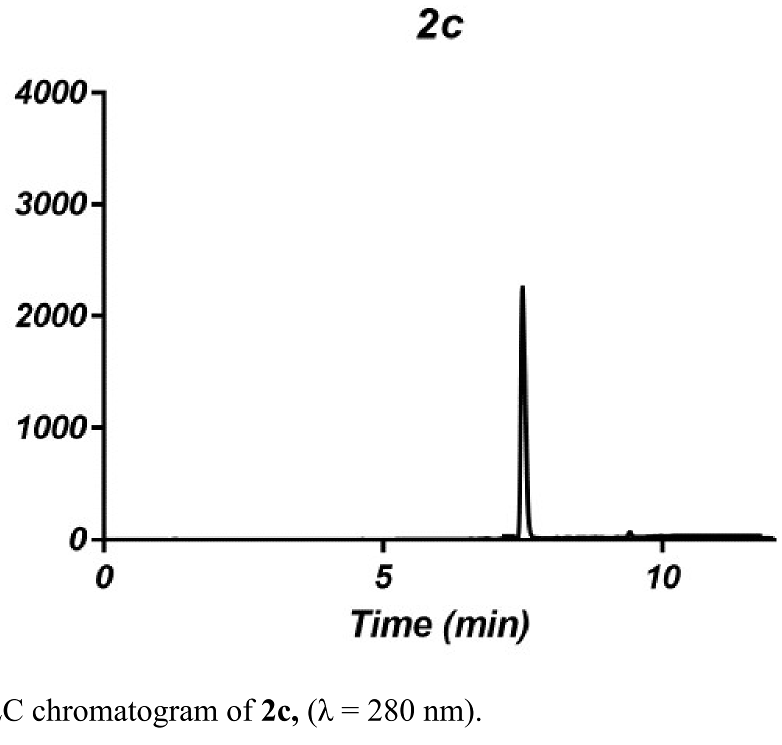

Fig. S54. HPLC chromatogram of **2c,** (λ = 280 nm).

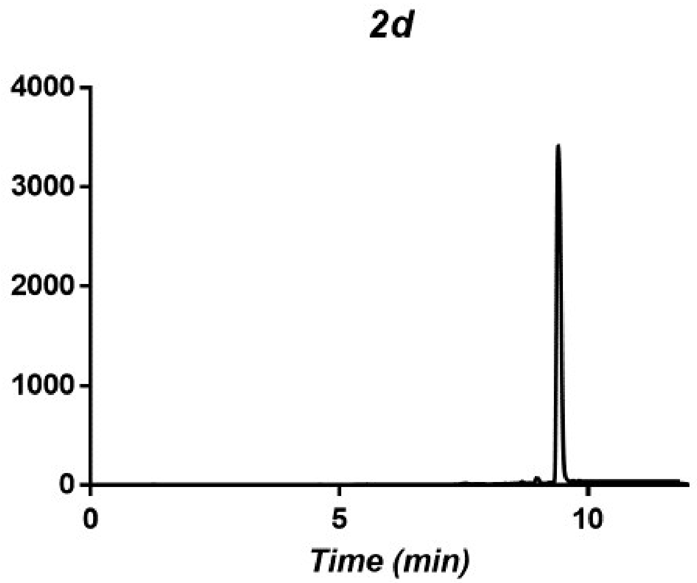

Fig. S55. HPLC chromatogram of **2d,** (λ = 280 nm).

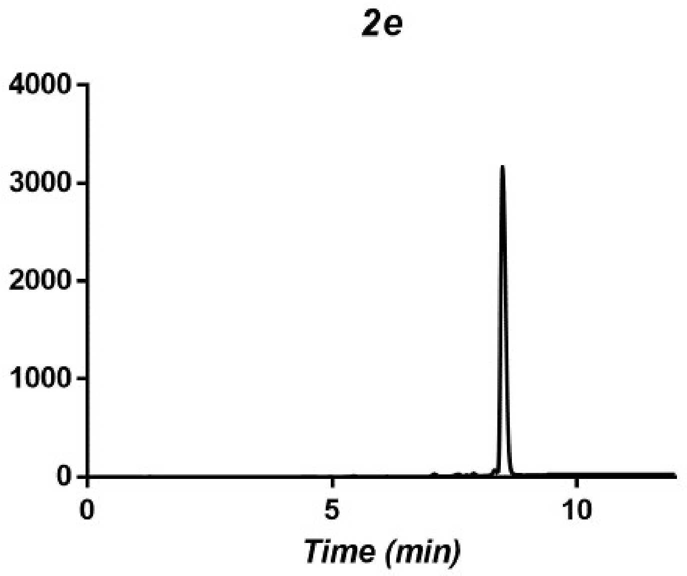

Fig. S56. HPLC chromatogram of **2e,** (λ = 280 nm).

UV-vis Stability in PBS of 1a-1e and 2b-2e:

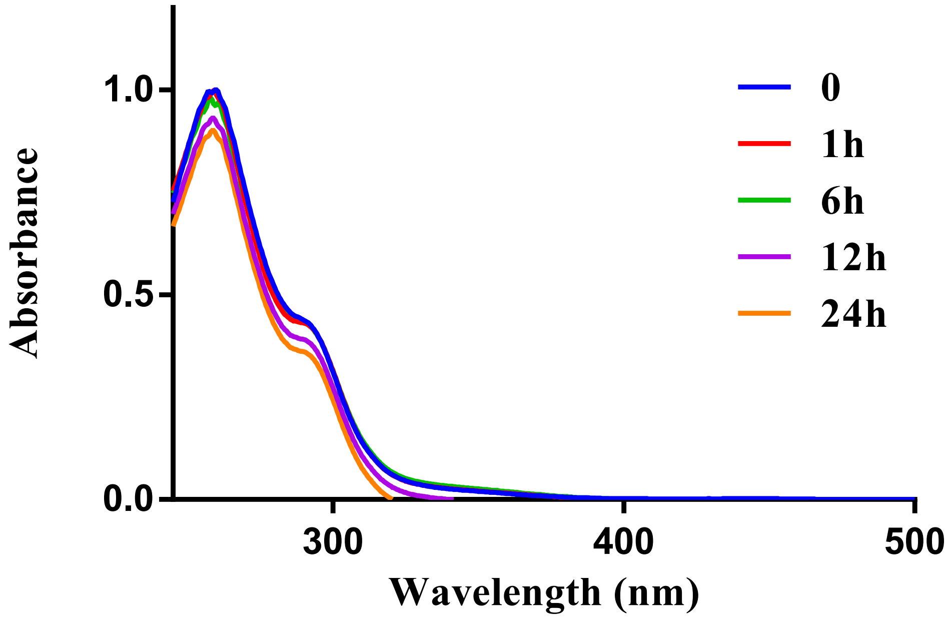

Fig. S57. UV-vis of **1a** in PBS (50 μM).

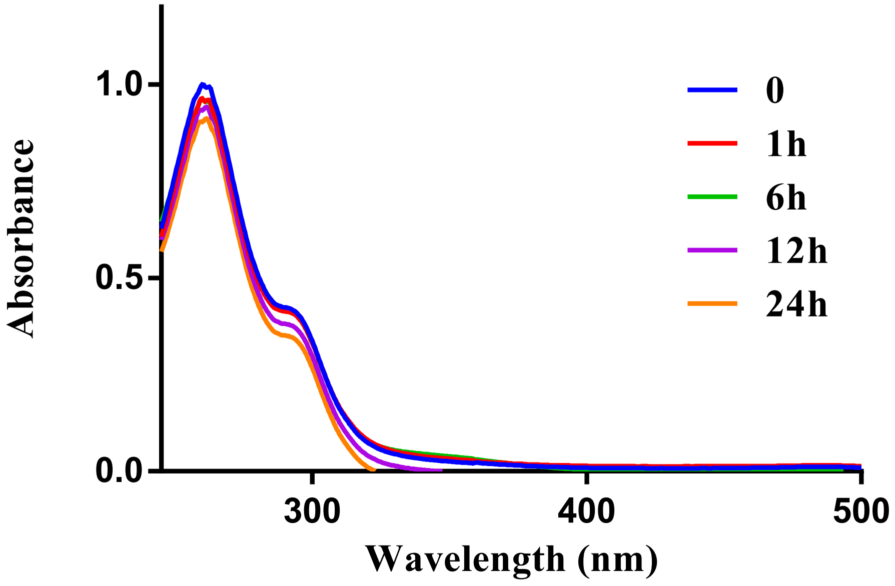

Fig. S58. UV-vis of **1b** in PBS (50 μM).

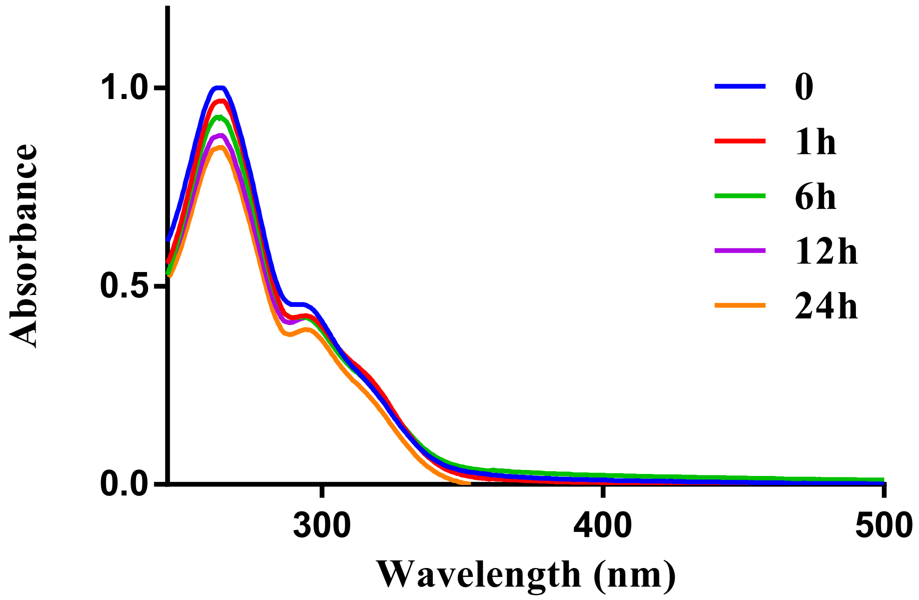

Fig. S59. UV-vis of **1c** in PBS (50 μM).

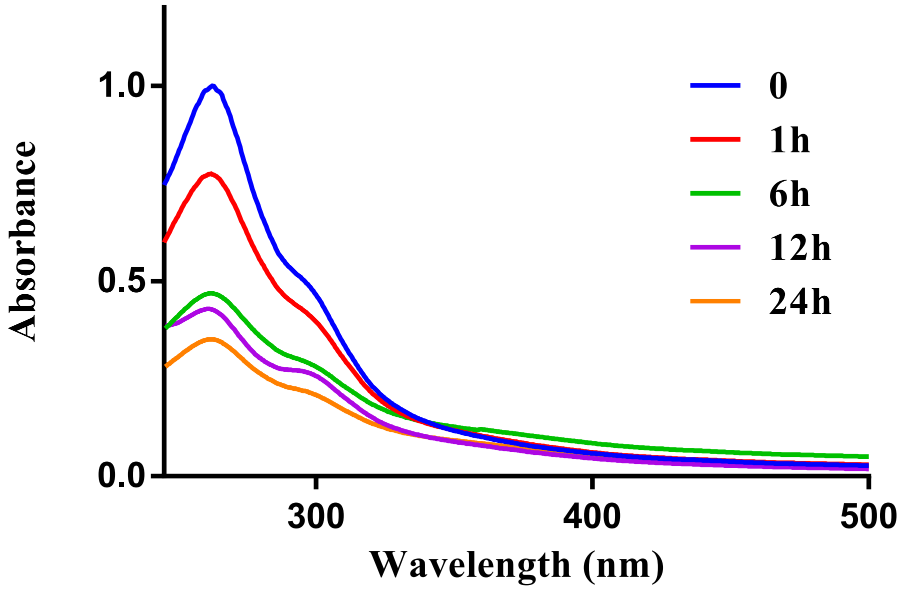

Fig. S60. UV-vis of **1d** in PBS (50 μM).

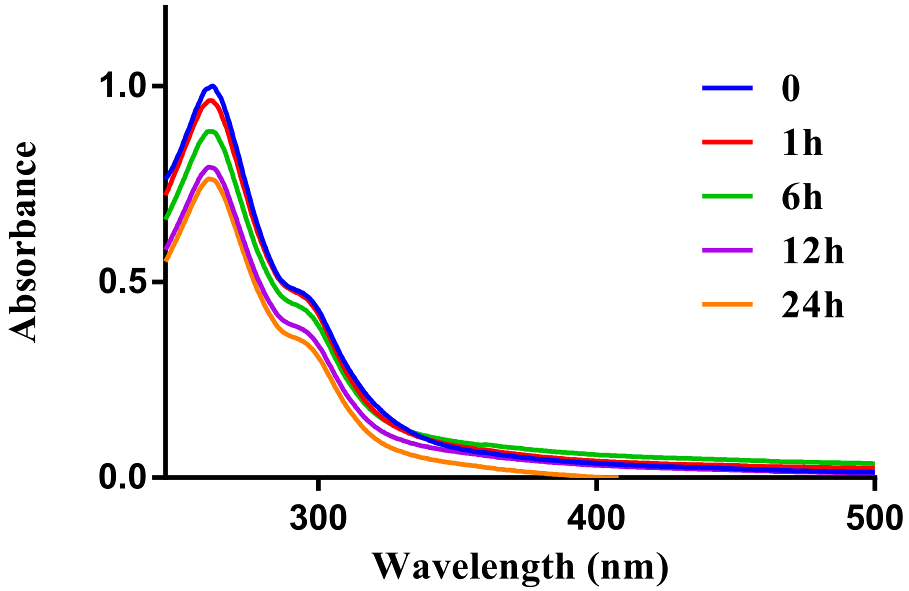

Fig. S61. UV-vis of **1e** in PBS (50 μM).

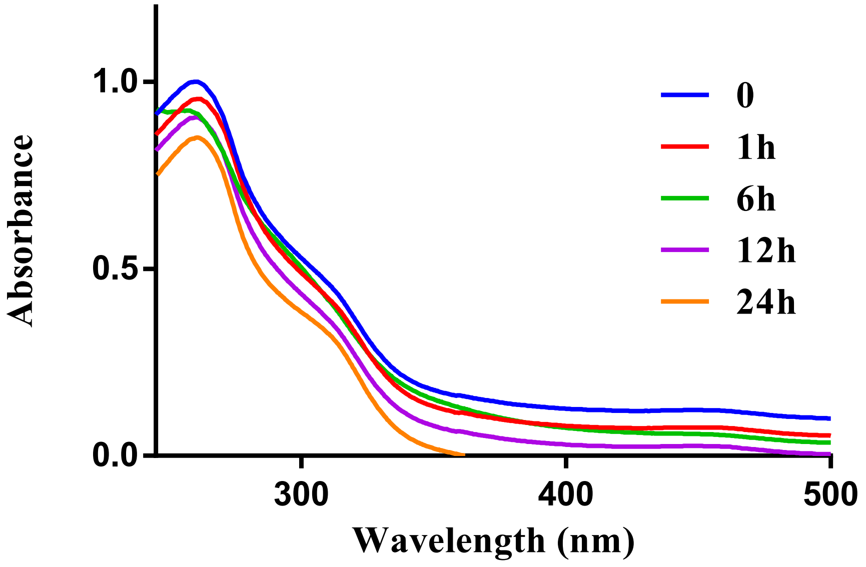

Fig. S62. UV-vis of **2b** in PBS (50 μM).

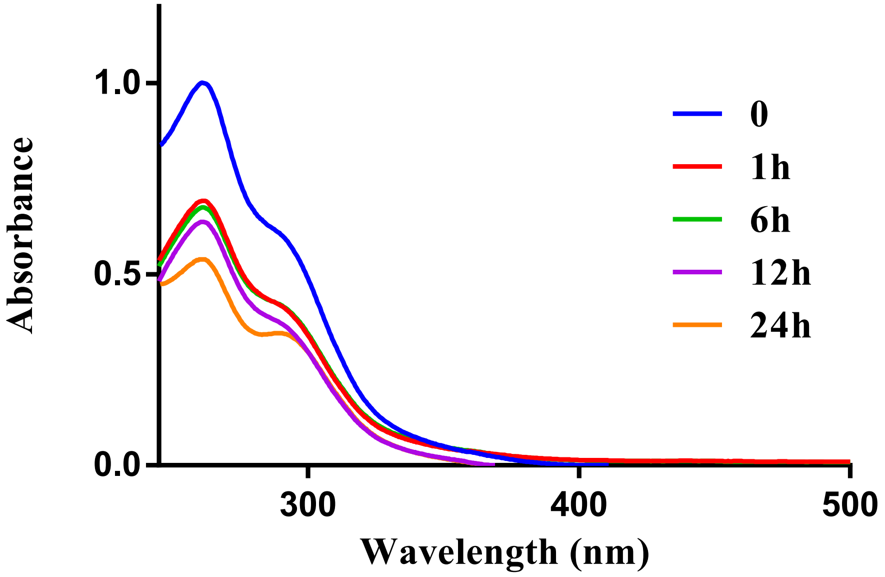

Fig. S63. UV-vis of **2c** in PBS (50 μM).

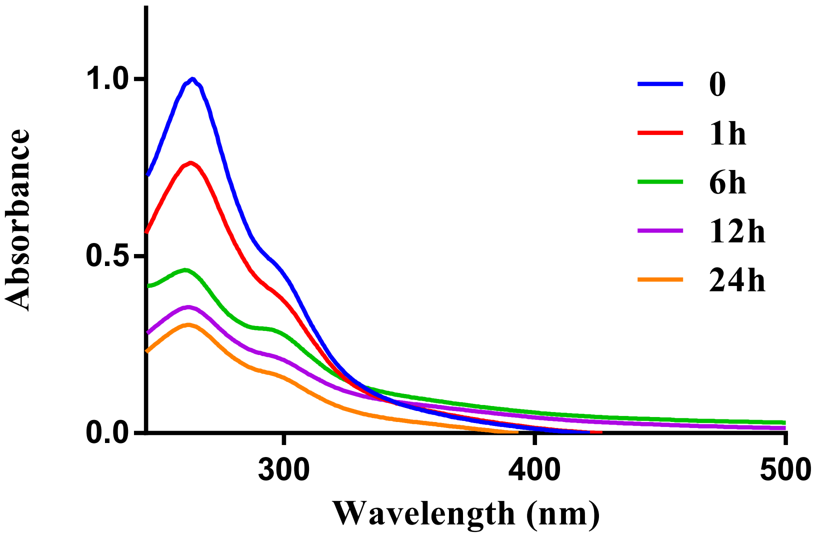

Fig. S64. UV-vis of **2d** in PBS (50 μM).

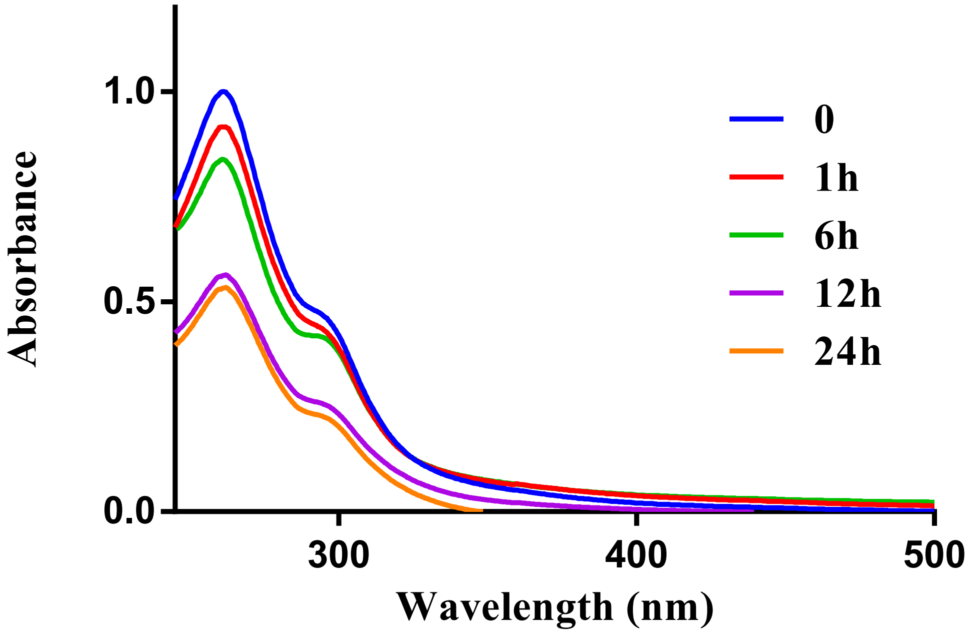

Fig. S65. UV-vis of **2e** in PBS (50 μM).

UV-vis of 1a-1e and 2b-2e in DMEM:

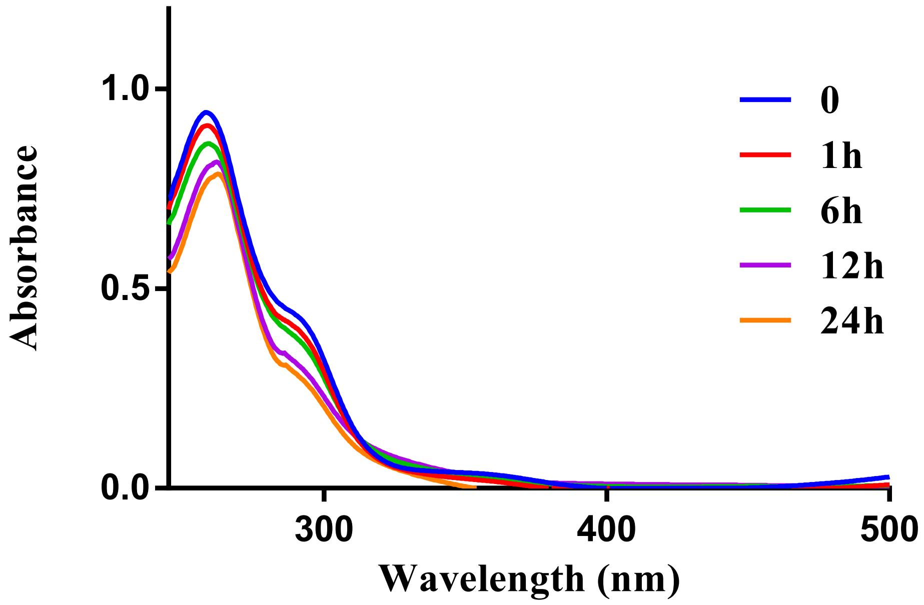

Fig. S66. UV-vis of **1a** in DMEM (50 μM).

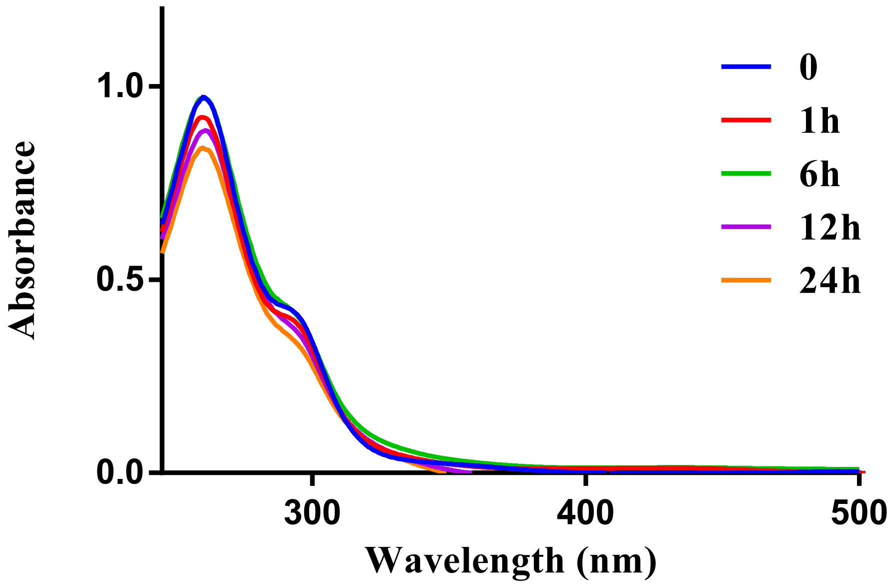

Fig. S67. UV-vis of **1b** in DMEM (50 μM).

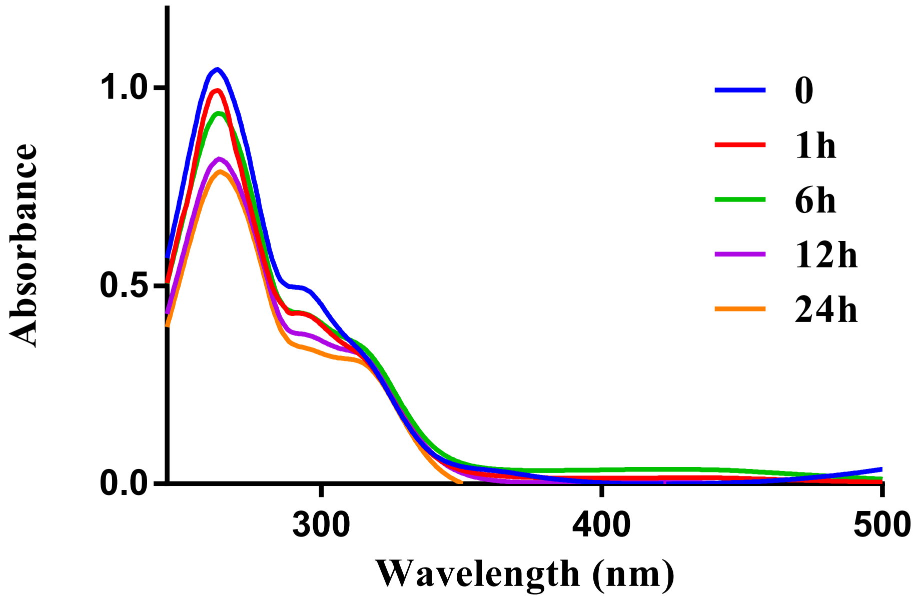

Fig. S68. UV-vis of **1c** in DMEM (50 μM).

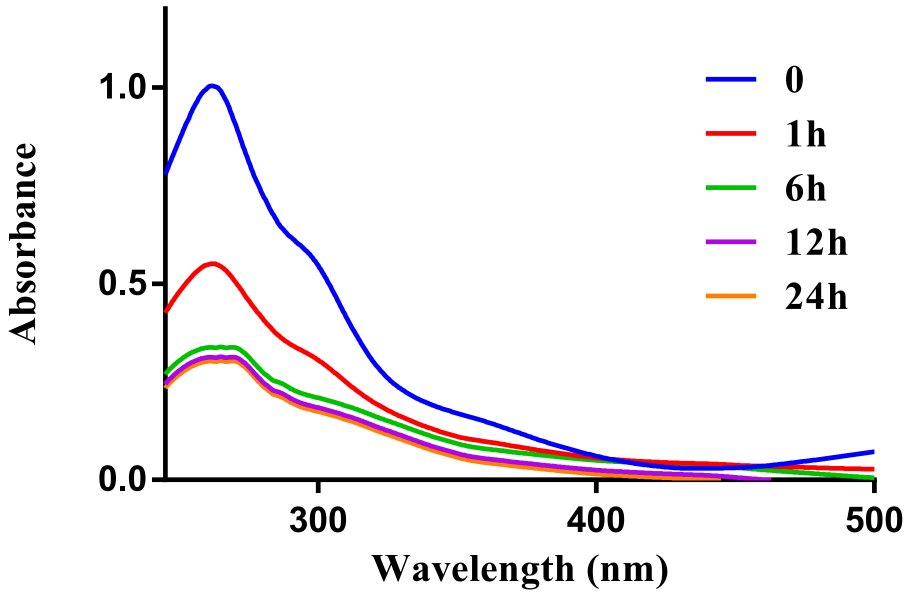

Fig. S69. UV-vis of **1d** in DMEM (50 μM).

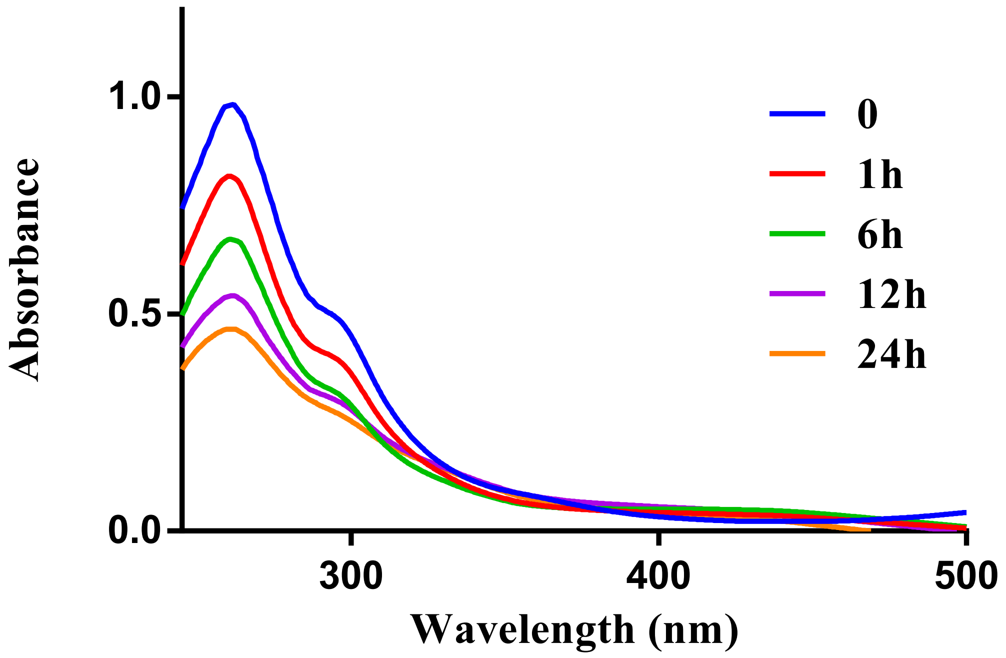

Fig. S70. UV-vis of **1e** in DMEM (50 μM).

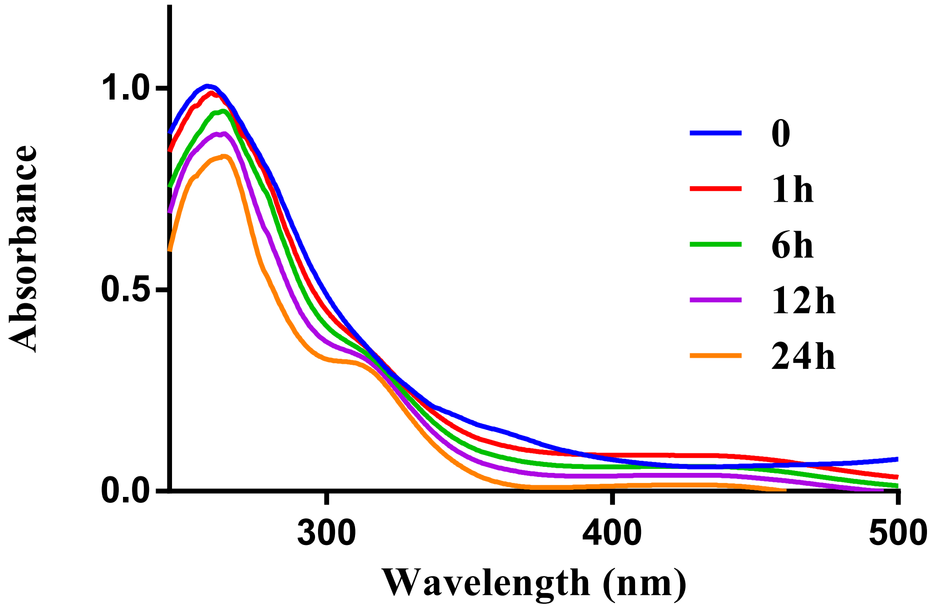

Fig. S71. UV-vis of **2b** in DMEM (50 μM).

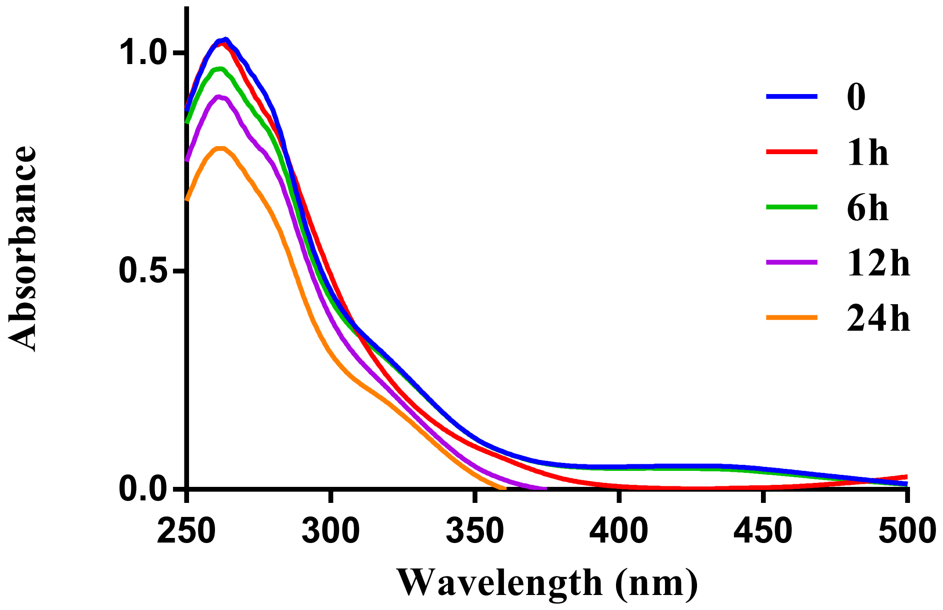

Fig. S72. UV-vis of **2c** in DMEM (50 μM).

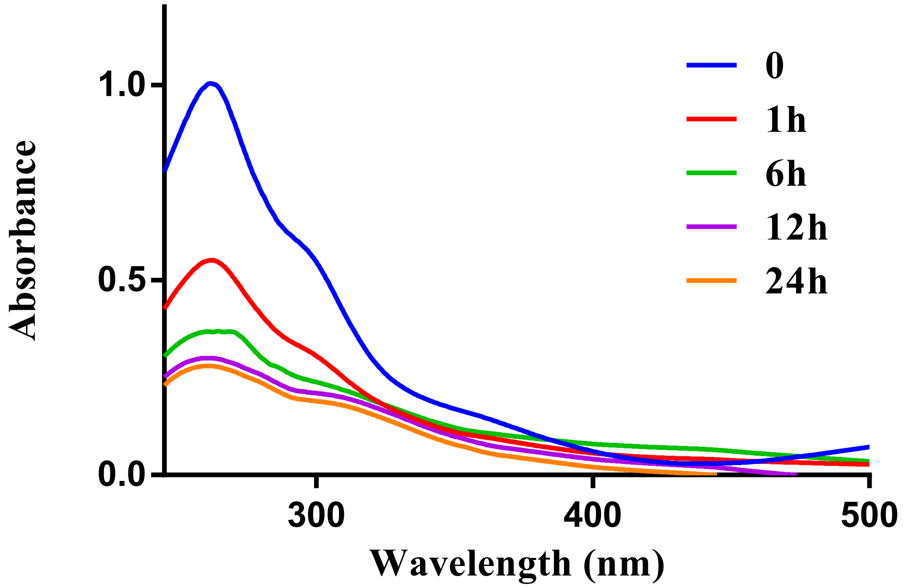

Fig. S73. UV-vis of **2d** in DMEM (50 μM).

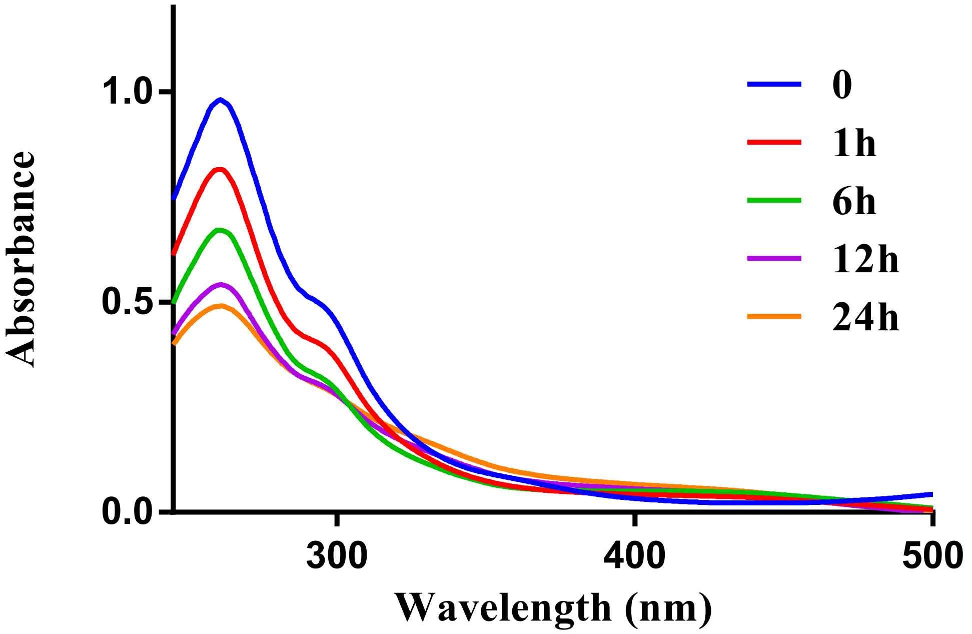

Fig. S74. UV-vis of **2e** in DMEM (50 μM).

UV-vis Stability in RPMI-1640 of 1a-1e and 2b-2e:

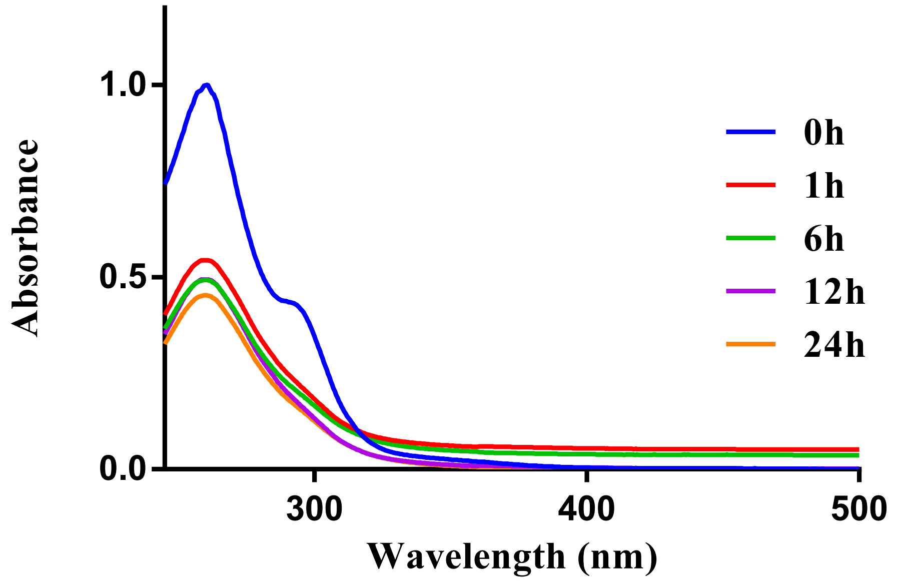

Fig. S75. UV-vis of **1a** in RPMI-1640 (50 μM).

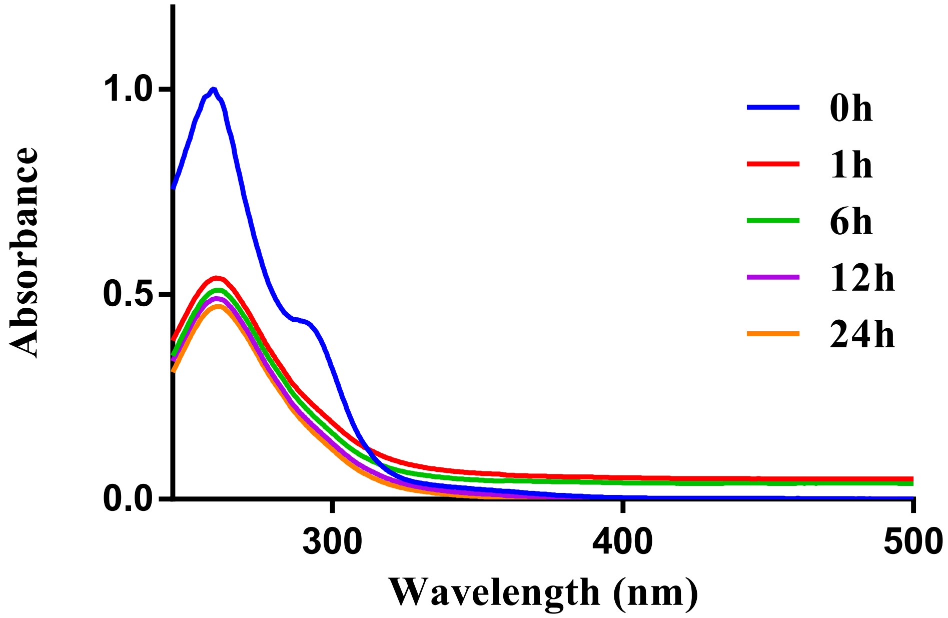

Fig. S76. UV-vis of **1b** in RPMI-1640 (50 μM).

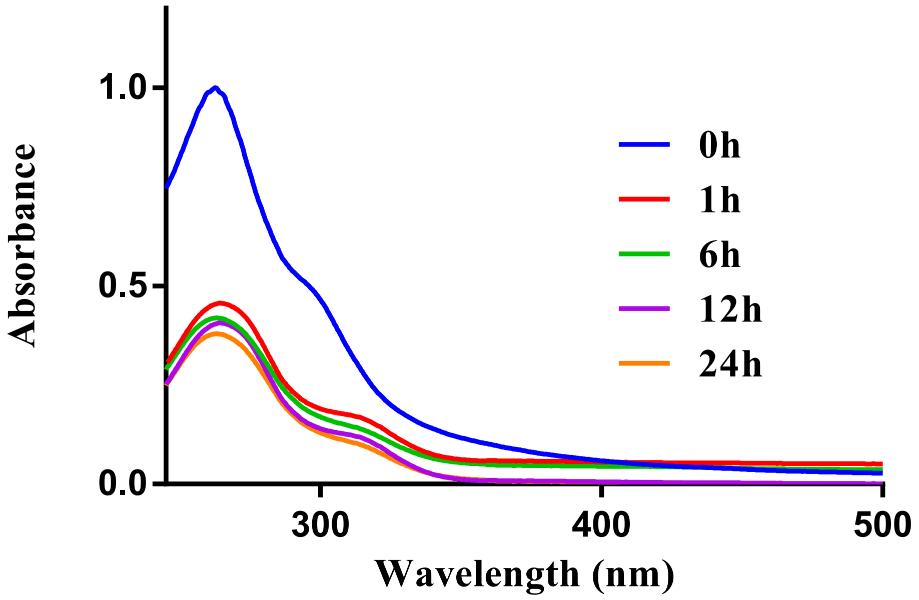

Fig. S77. UV-vis of **1c** in RPMI-1640 (50 μM).

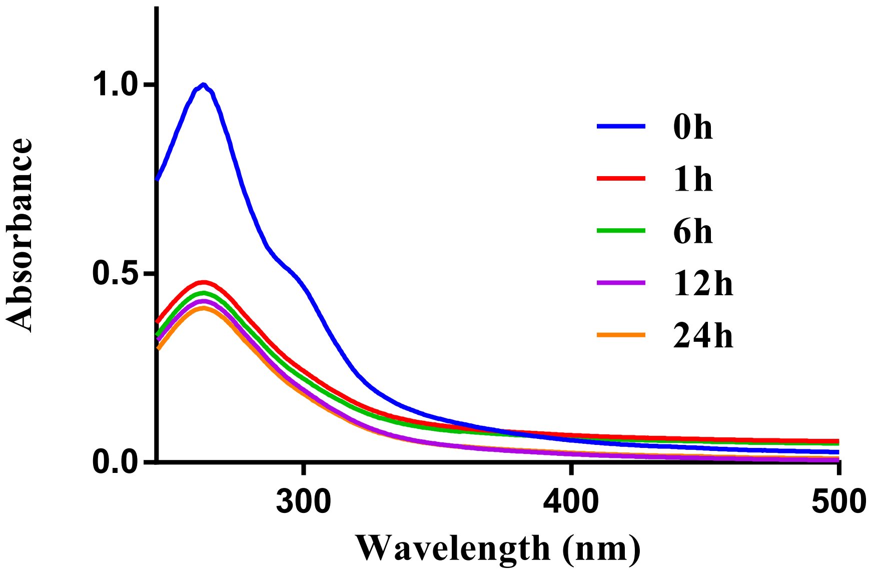

Fig. S78. UV-vis of **1d** in RPMI-1640 (50 μM).

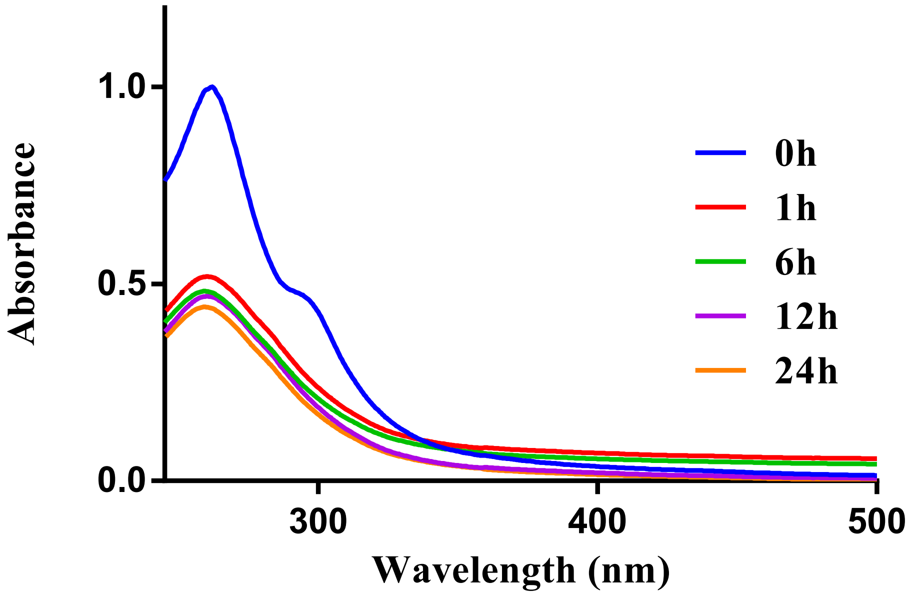

Fig. S79. UV-vis of **1e** in RPMI-1640 (50 μM).

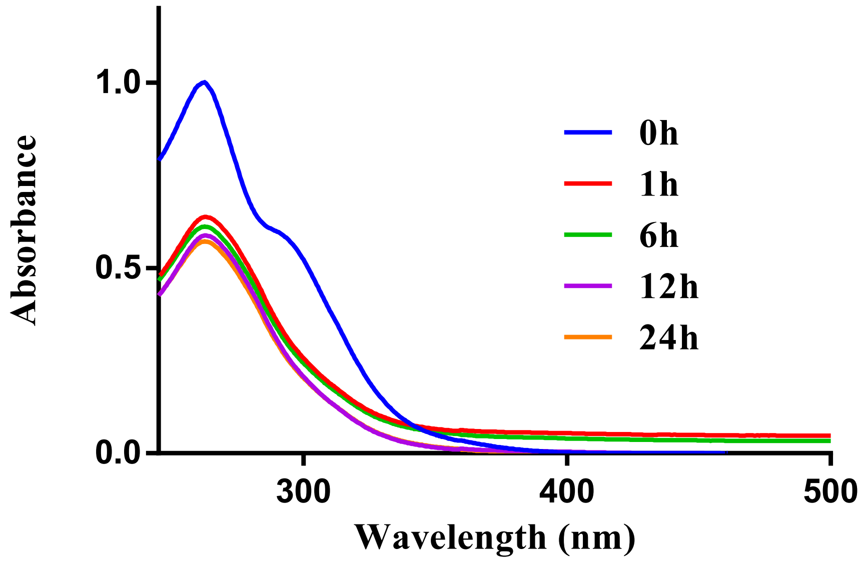

Fig. S80. UV-vis of **2b** in RPMI-1640 (50 μM).

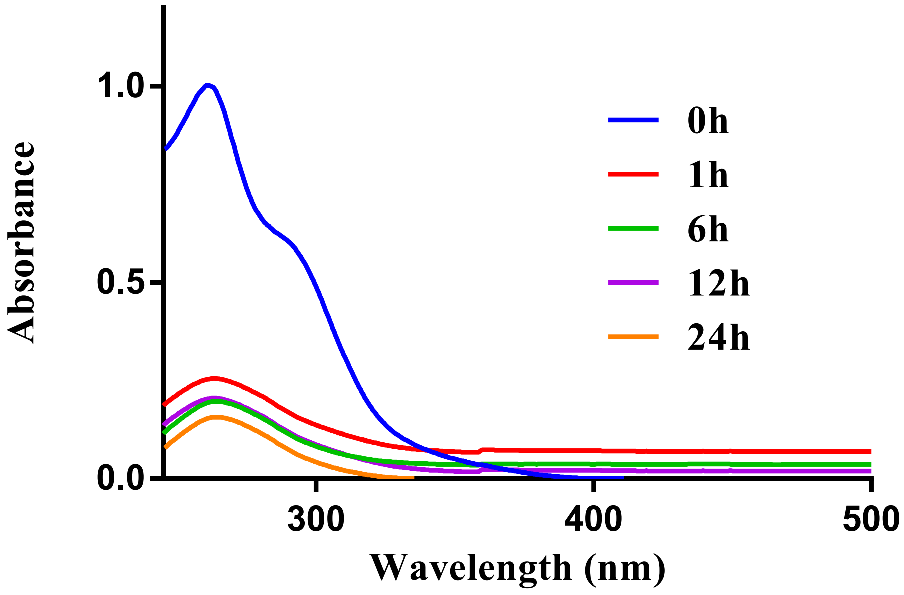

Fig. S81. UV-vis of **2c** in RPMI-1640 (50 μM).

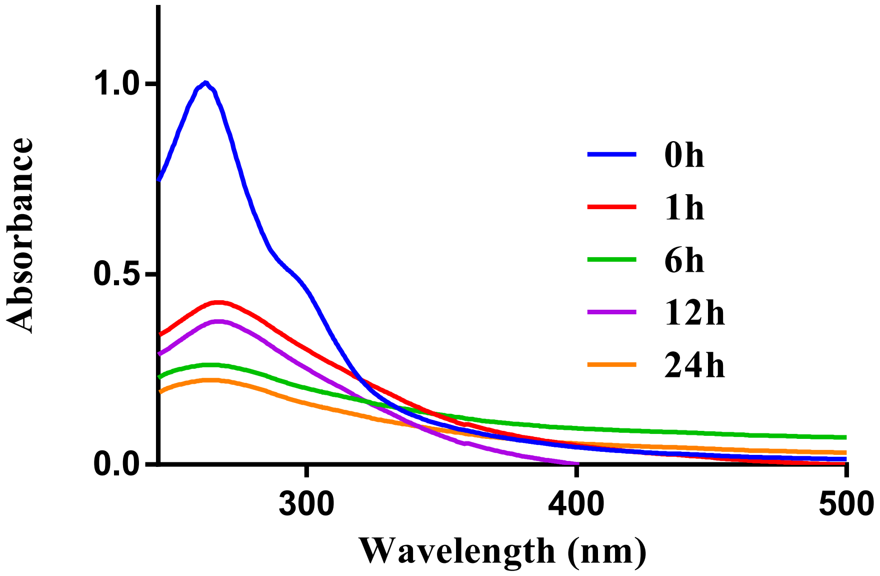

Fig. S82. UV-vis of **2d** in RPMI-1640 (50 μM).

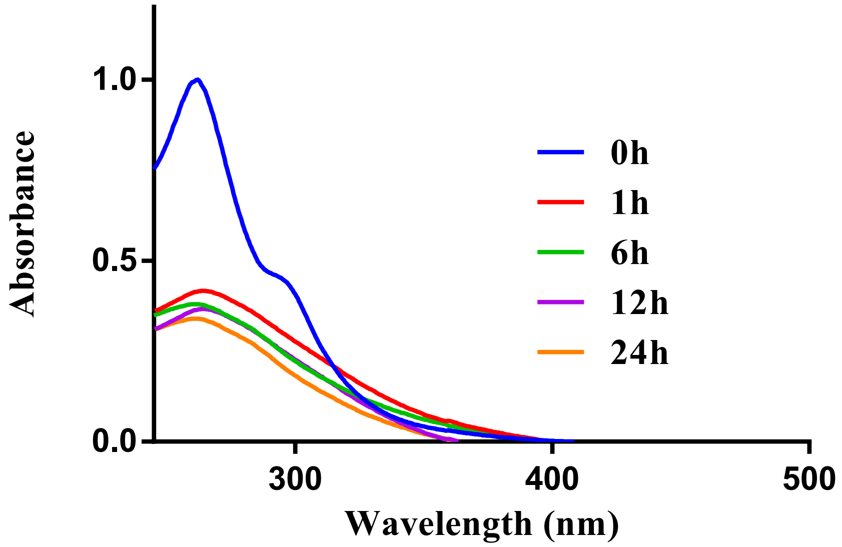

Fig. S83. UV-vis of **2e** in RPMI-1640 (50 μM).

Reactivity of 1a-1e and 2a-2e with GSH (Monitored *via* UV-vis Spectroscopy):

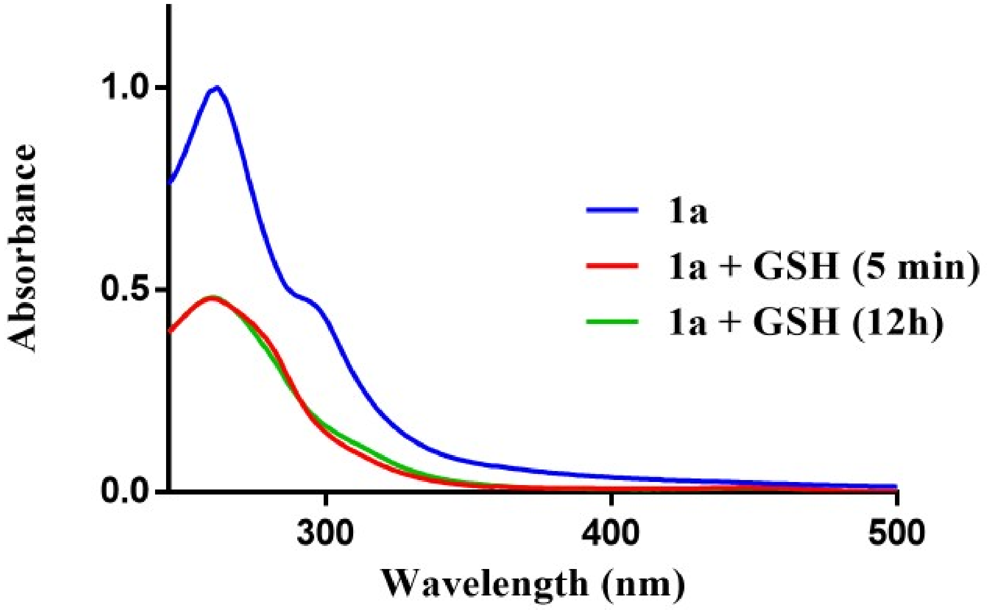

Fig. S84. UV-vis of **1a + GSH** (1:1 ratio, 25 µM) in PBS.

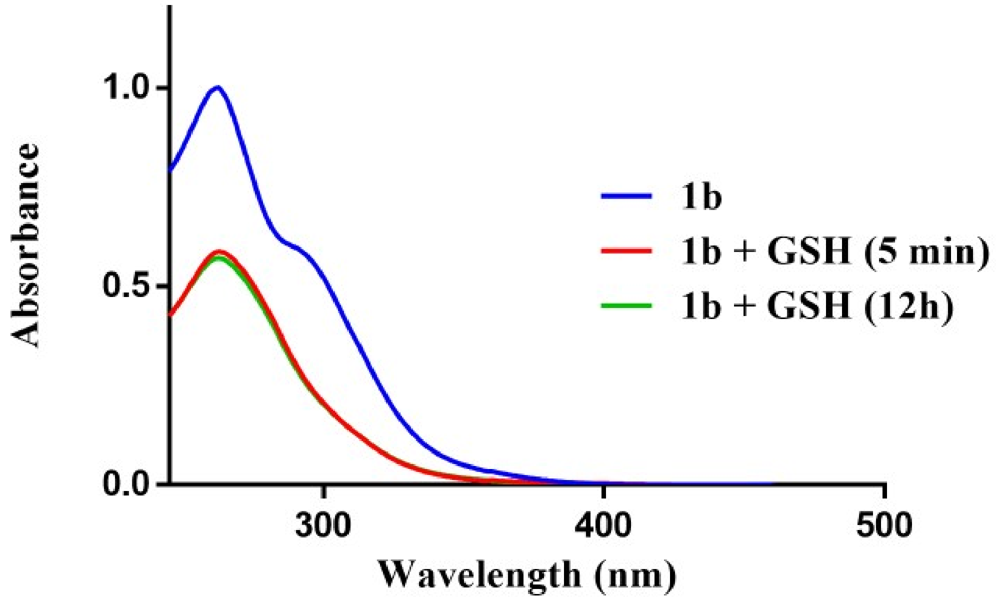

Fig. S85. UV-vis of **1b + GSH** (1:1 ratio, 25 µM) in PBS.

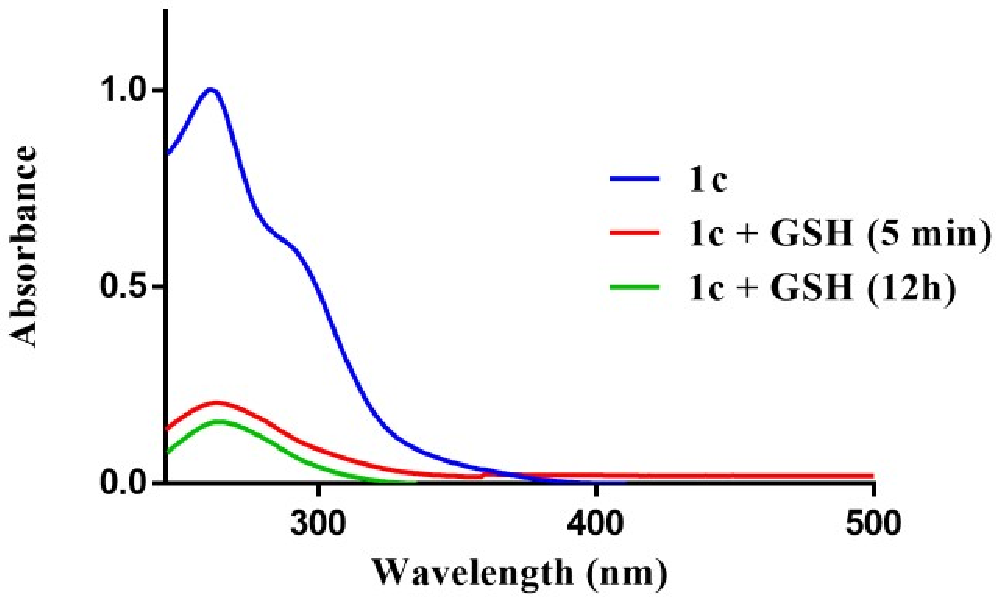

Fig. S86. UV-vis of **1c + GSH** (1:1 ratio, 25 µM) in PBS.

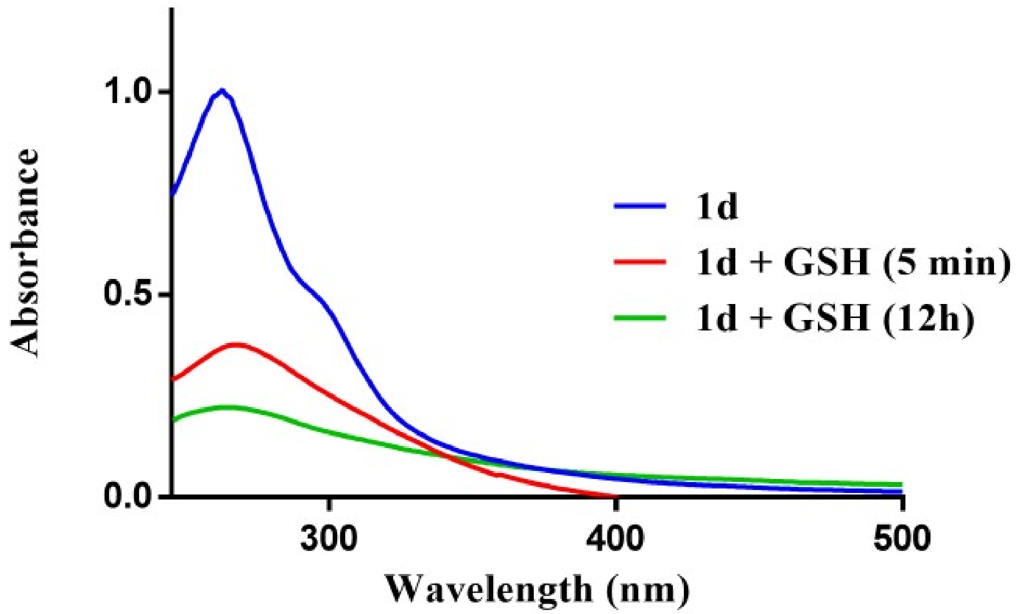

Fig. S87. UV-vis of **1d + GSH** (1:1 ratio, 25 µM) in PBS.

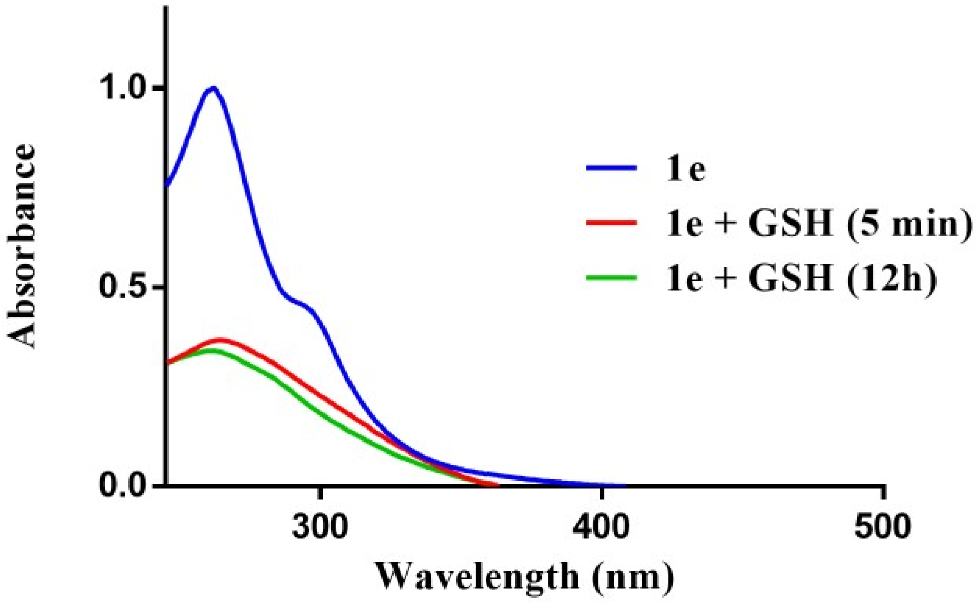

Fig. S88. UV-vis of **1e + GSH** (1:1 ratio, 25 μM) in PBS.

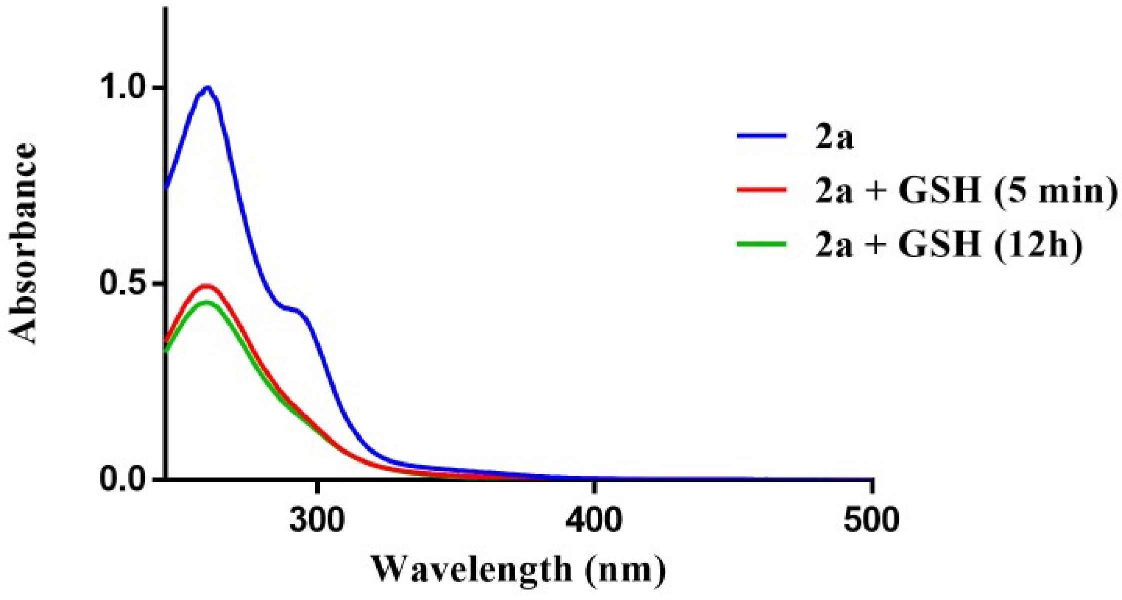

Fig. S89. UV-vis of **2a + GSH** (1:1 ratio, 25 μM) in PBS.

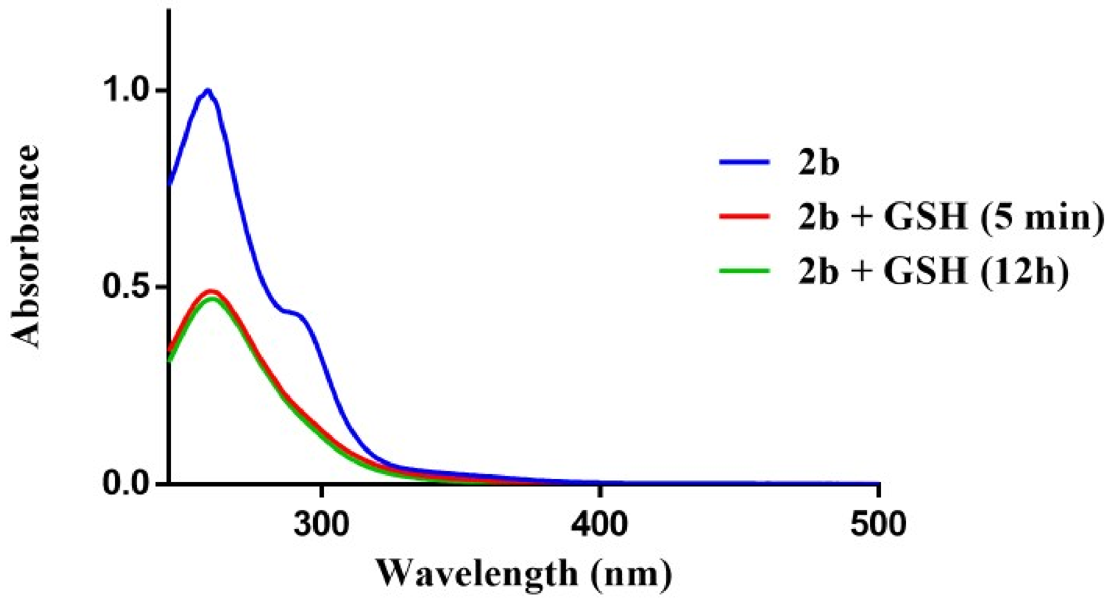

Fig. S90. UV-vis of **2b + GSH** (1:1 ratio, 25 μM) in PBS.

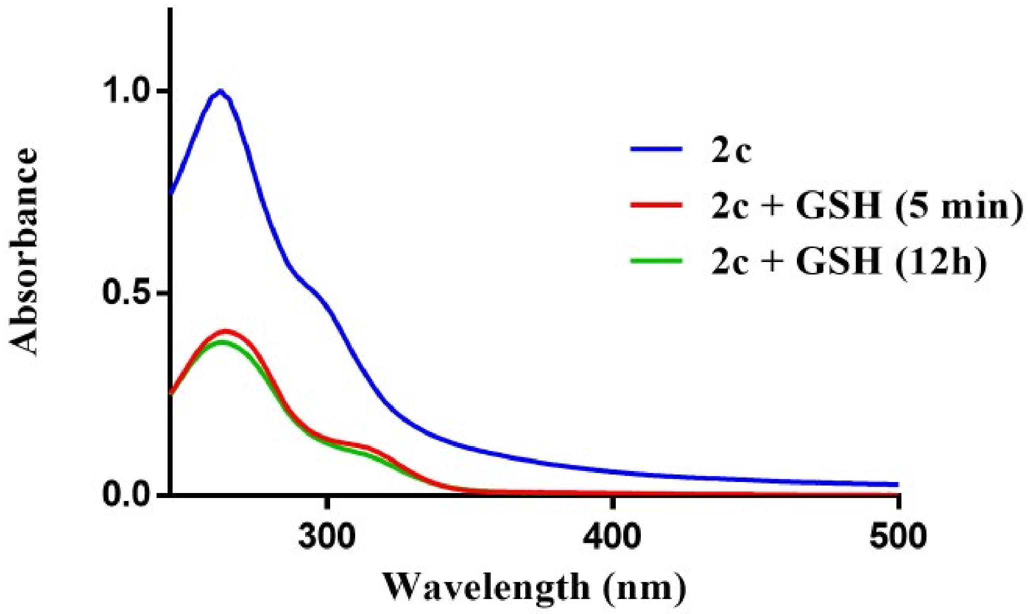

Fig. S91. UV-vis of **2c + GSH** (1:1 ratio, 25 μM) in PBS.

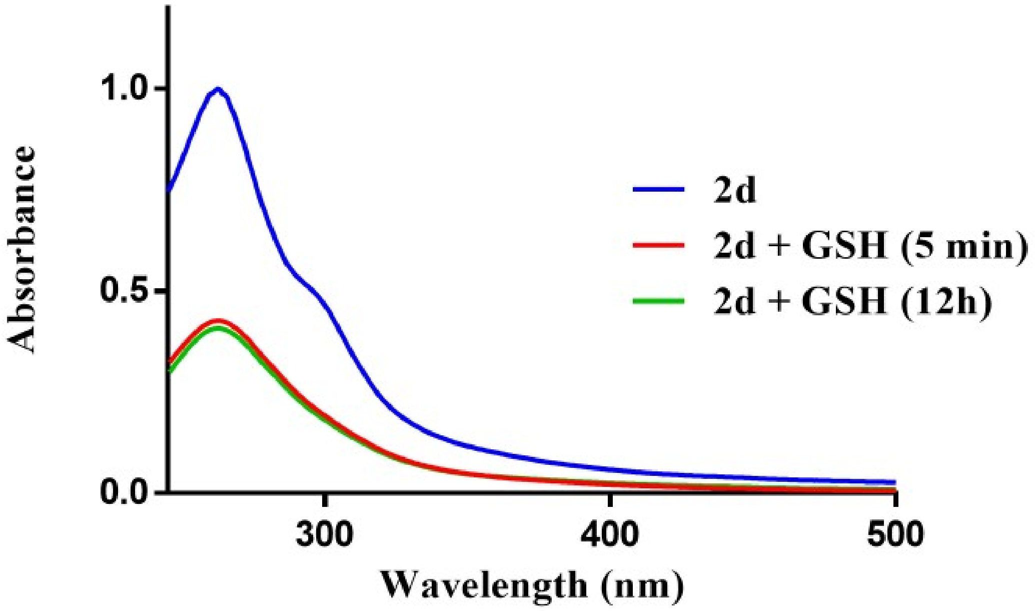

Fig. S92. UV-vis of **2d + GSH** (1:1 ratio, 25 μM) in PBS.

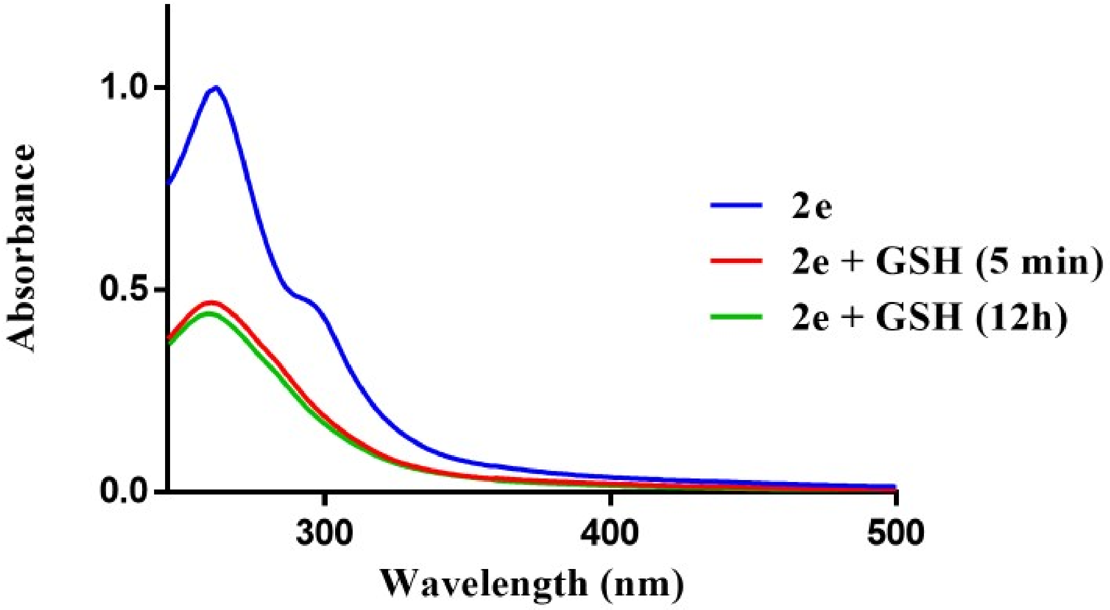

Fig. S93. UV-vis of **2e + GSH** (1:1 ratio, 25 μM) in PBS.

Reactivity of 1a-1e and 2a-2e with NAC (Monitored *via* UV-vis Spectroscopy):

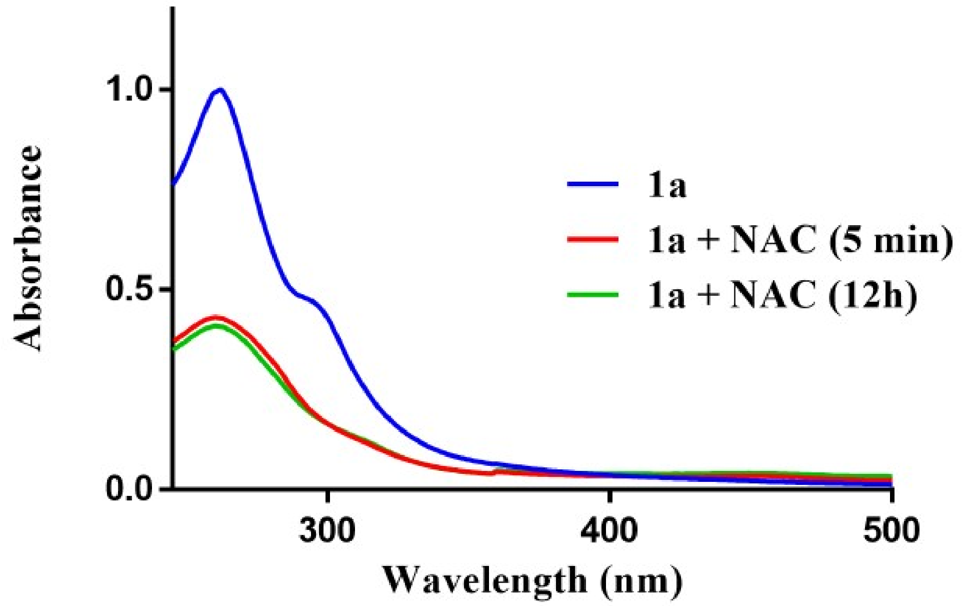

Fig. S94. UV-vis of **1a + NAC** (1:1 ratio, 25 μM) in PBS.

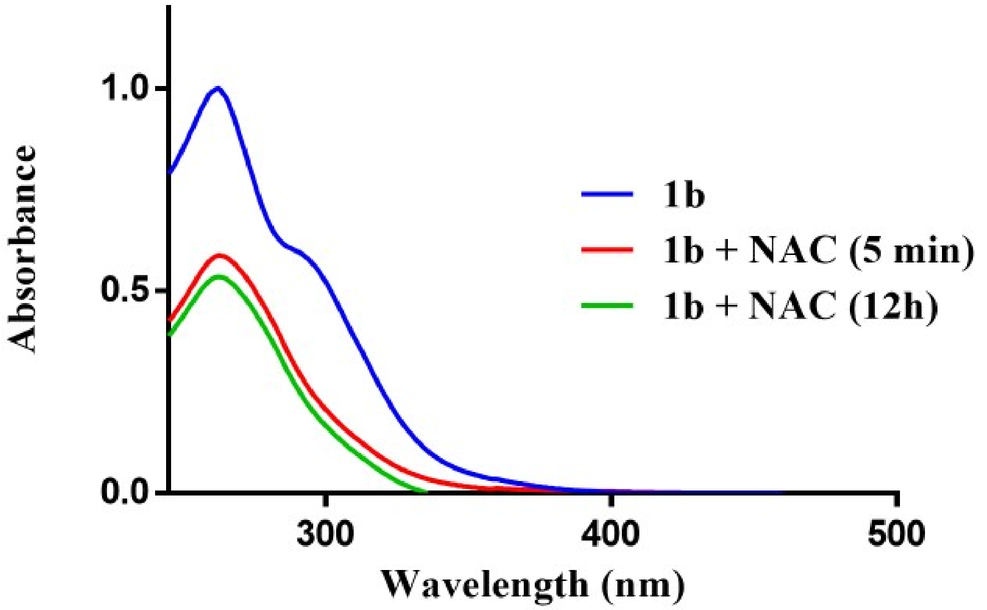

Fig. S95. UV-vis of **1b + NAC** (1:1 ratio, 25 μM) in PBS.

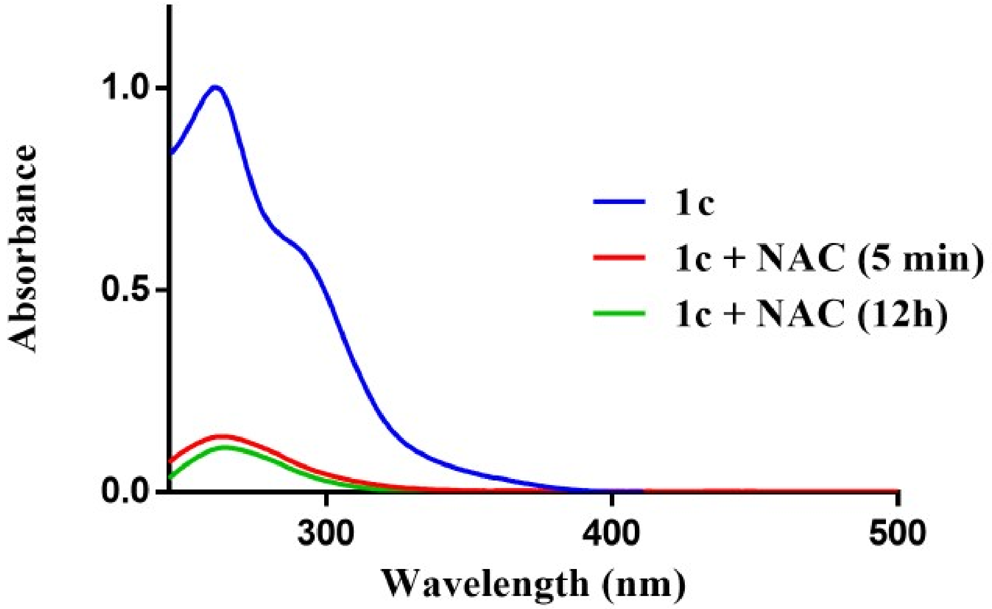

Fig. S96. UV-vis of **1c + NAC** (1:1 ratio, 25 μM) in PBS.

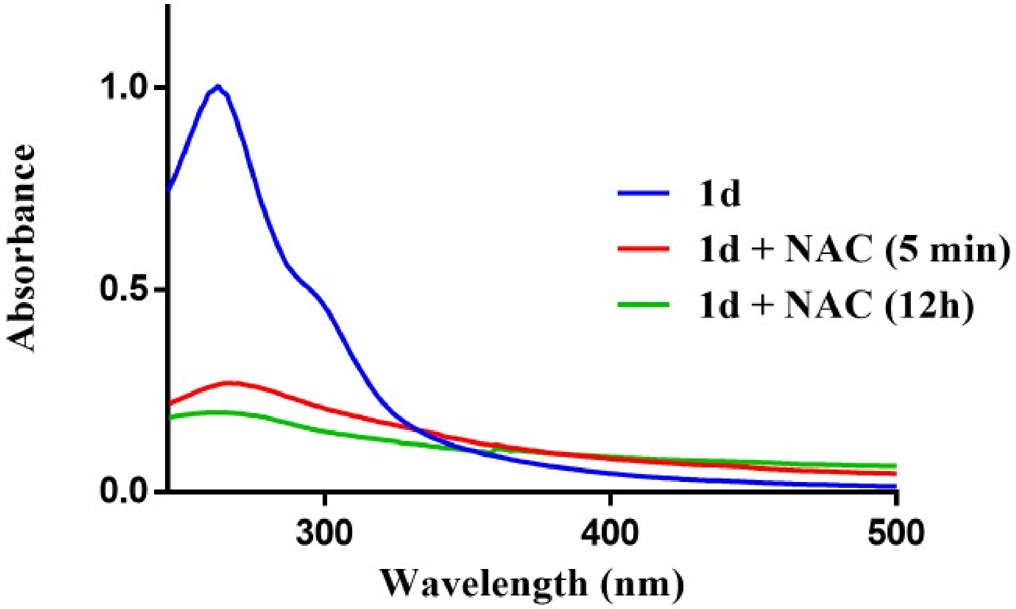

Fig. S97. UV-vis of **1d + NAC** (1:1 ratio, 25 μM) in PBS**.**

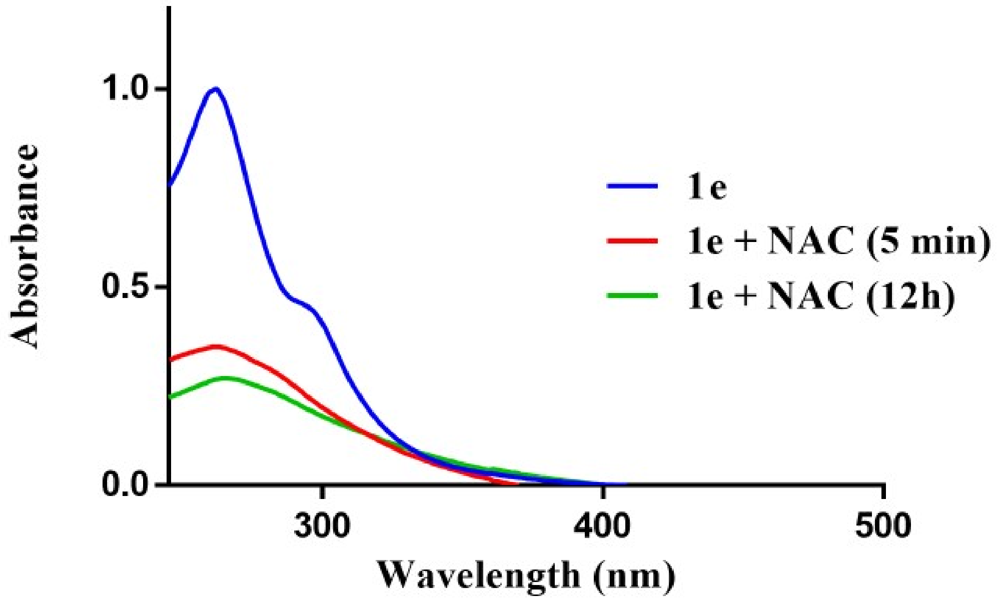

Fig. S98. UV-vis of **1e + NAC** (1:1 ratio, 25 μM) in PBS.

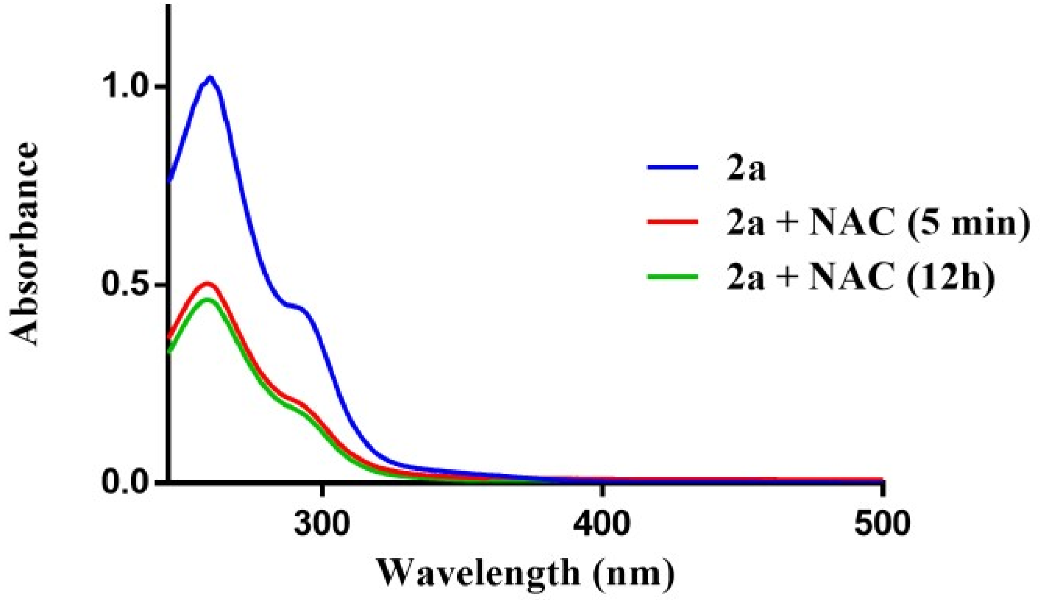

Fig. S99. UV-vis of **2a + NAC** (1:1 ratio, 25 μM) in PBS.

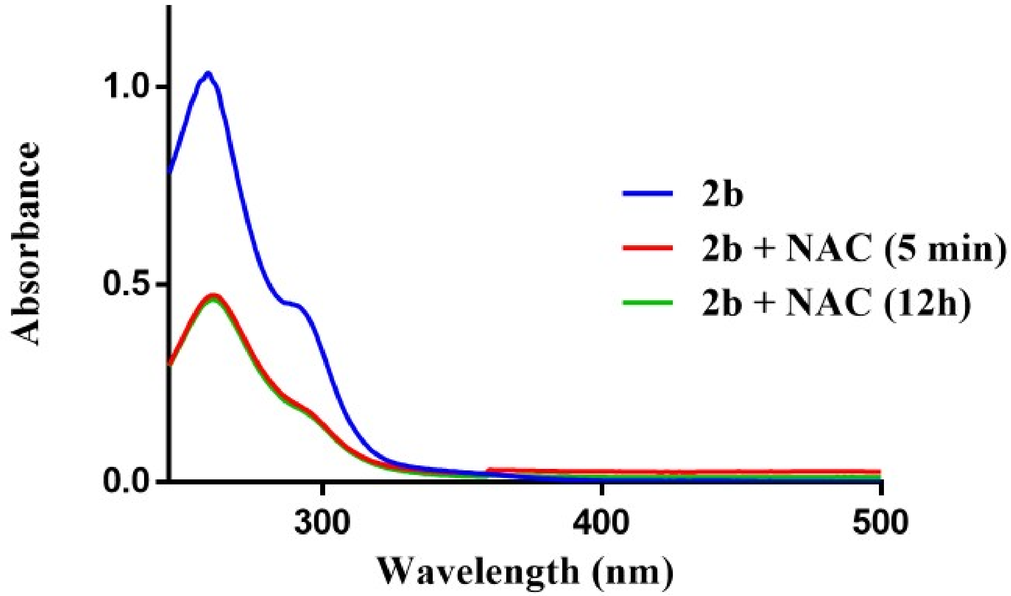

Fig. S100. UV-vis of **2b** + **NAC** (1:1 ratio, 25 μM) in PBS.

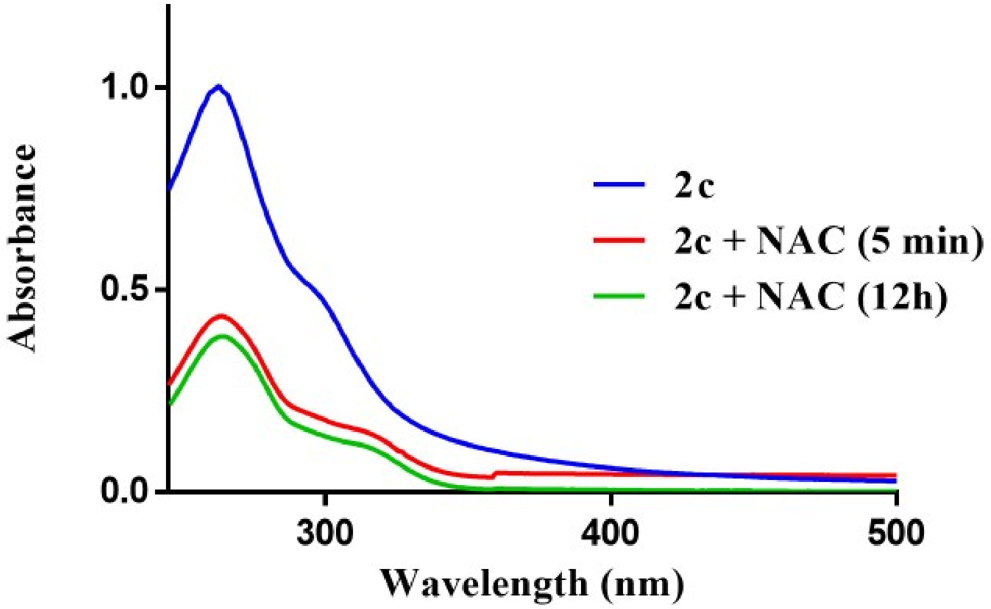

Fig. S101. UV-vis of **2c + NAC** (1:1 ratio, 25 μM) in PBS.

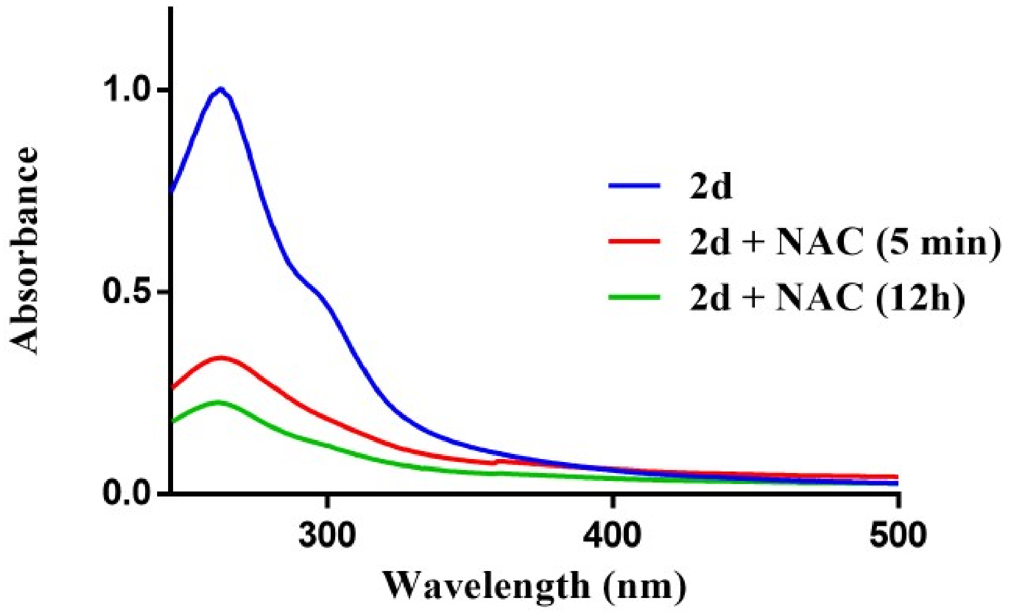

Fig. S102. UV-vis of **2d + NAC** (1:1 ratio, 25 μM) in PBS.

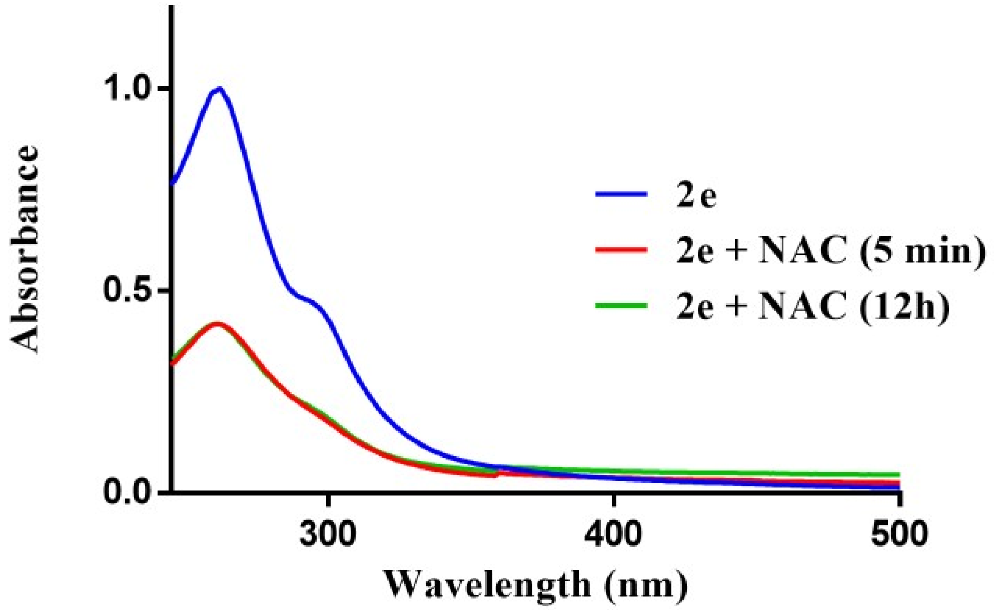

Fig. S103. UV-vis of **2e + NAC** (1:1 ratio, 25 μM) in PBS.

LC-MS of 2a + GSH:

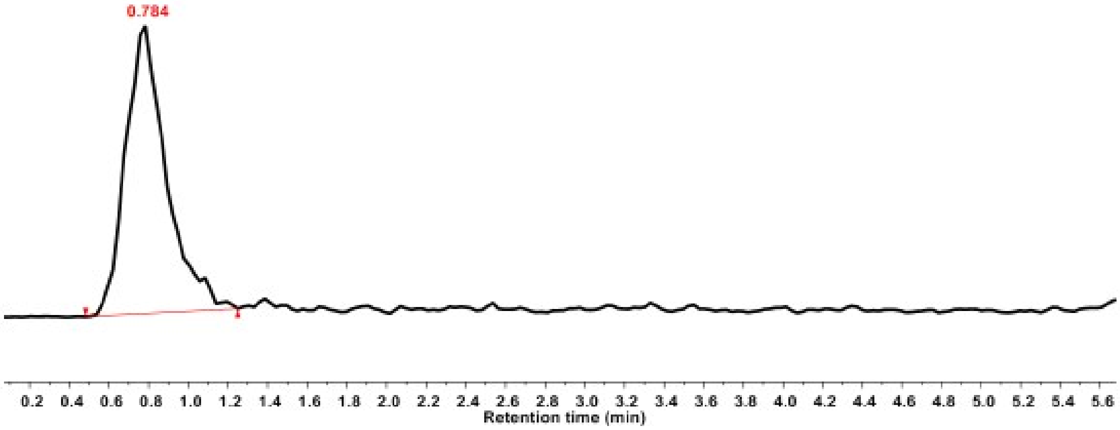

Fig. S104. LC-MS chromatogram of the adduct formed between **2a** + **GSH**, (2.5 mM, λ = 280 nm).

Reactivity with GSH (^{1}H NMR Spectroscopy:

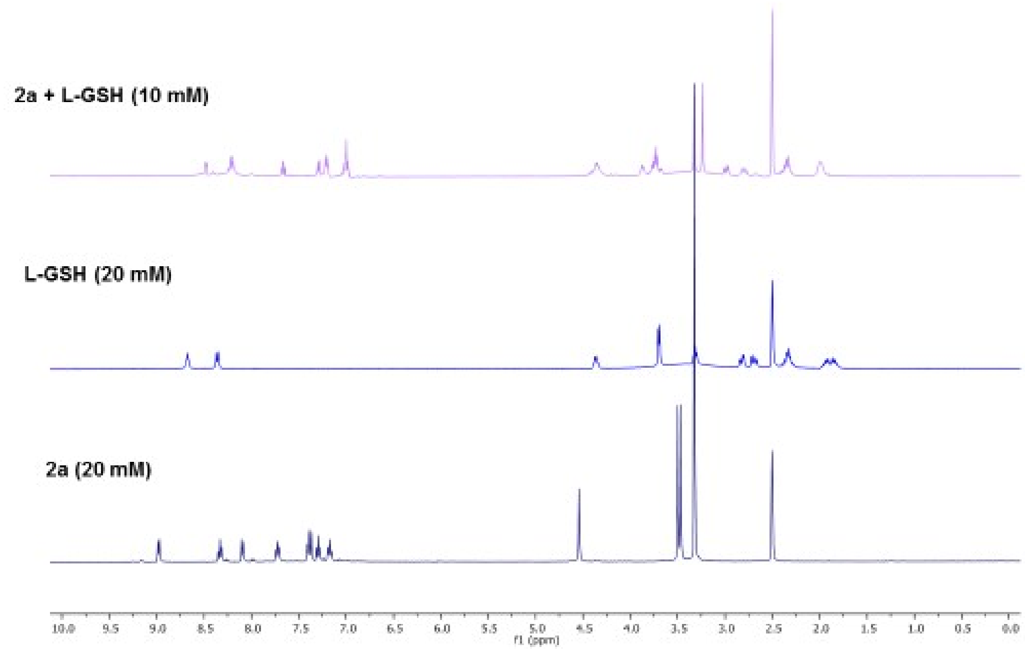

Fig. S105. Full ^{1}H NMR spectrum (400 MHz, DMSO-d_6) of **2a** (20 mM), **GSH** (20 mM), and **2a** + **GSH** (10 mM).

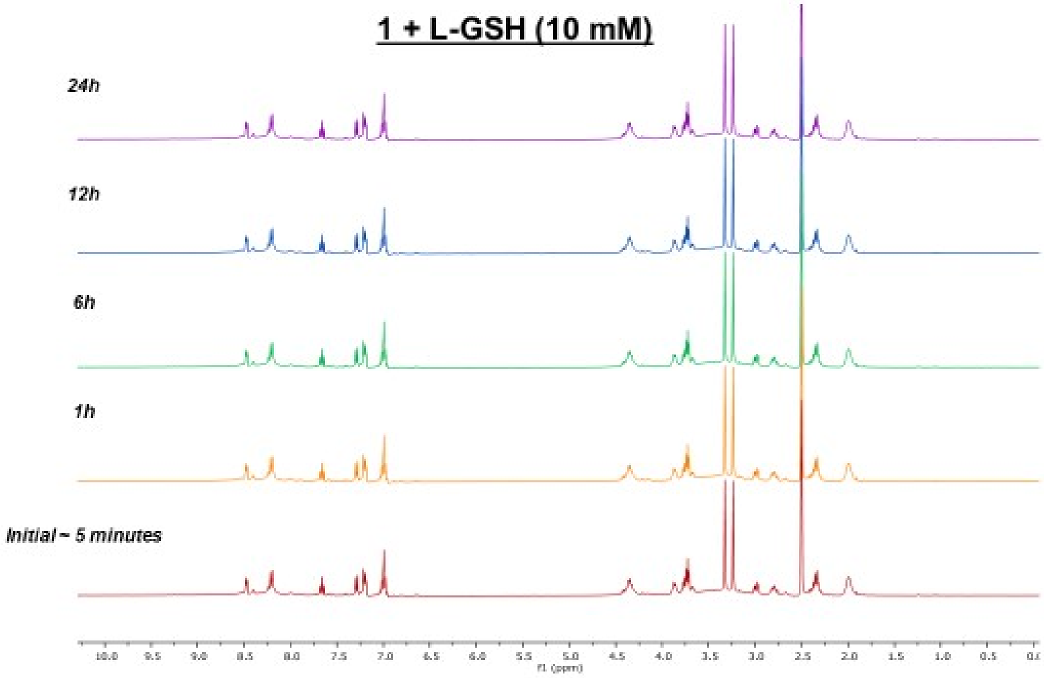

Fig. S106. Full ^{1}H NMR spectrum (400 MHz, DMSO-d_6) of **2a** + **GSH** (10 mM) at different time intervals. The spectrum is consistent across all time periods indicating the reaction occurs immediately and the adduct is stable in solution.

Cyclic Voltammetry:

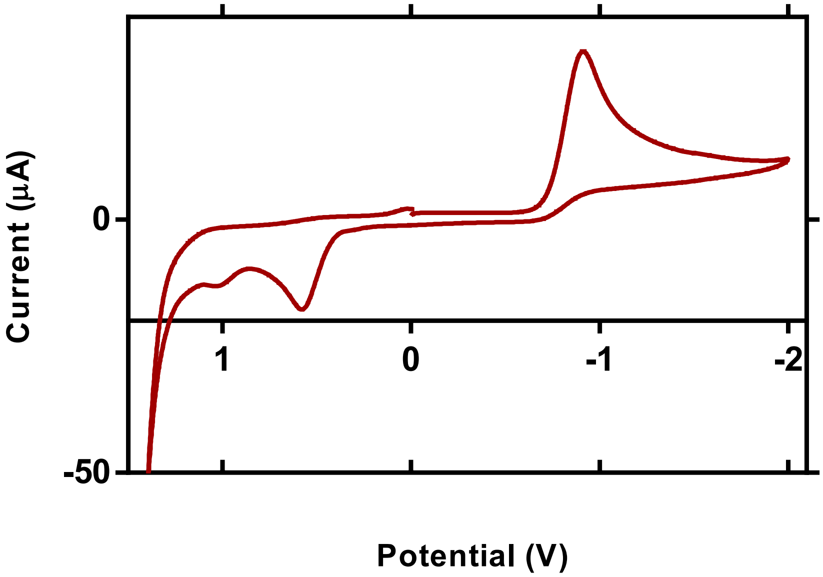

Fig. S107. Cyclic voltammogram of (2-benzylpyridine)$AuCl_2$ (2 mM) in DMSO with NBu_4PF_6 (0.1 M) as the electrolyte.

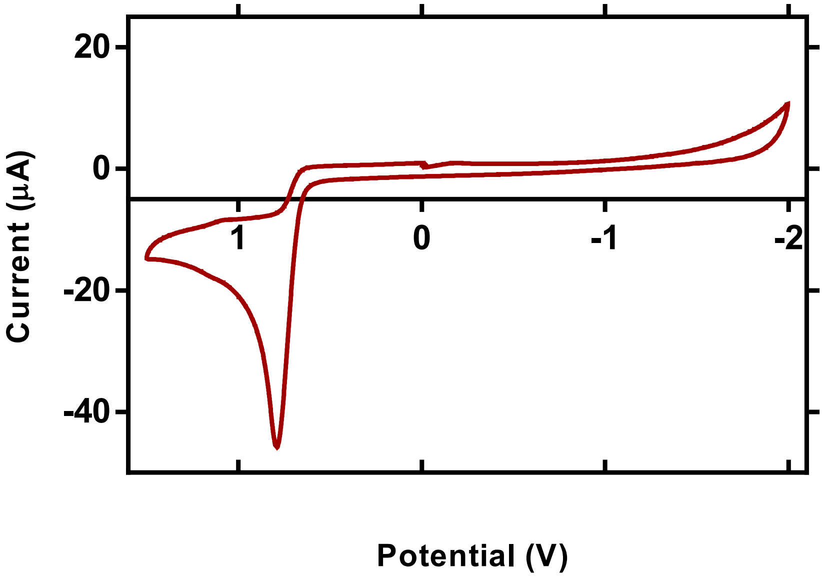

Fig. S108. Cyclic voltammogram of sodium diethyldithiocarbamate (2 mM) in DMSO with NBu_4PF_6 (0.1 M) as the electrolyte.

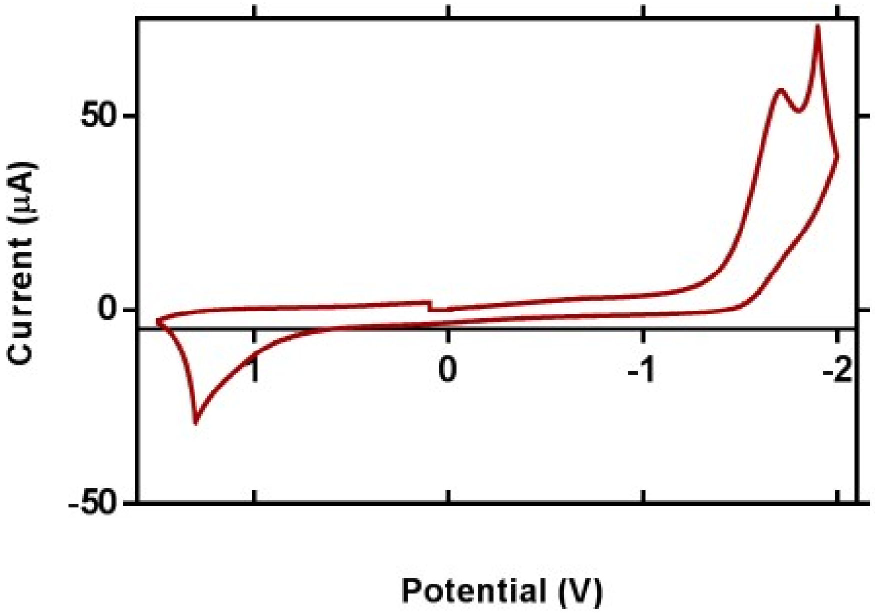

Fig. S109. Cyclic voltammogram of GSH (2 mM) in DI H_2O with $NaClO_4$ (0.1 M) as the electrolyte.

Cell Viability of 1a + GSH in MDA-MB-231:

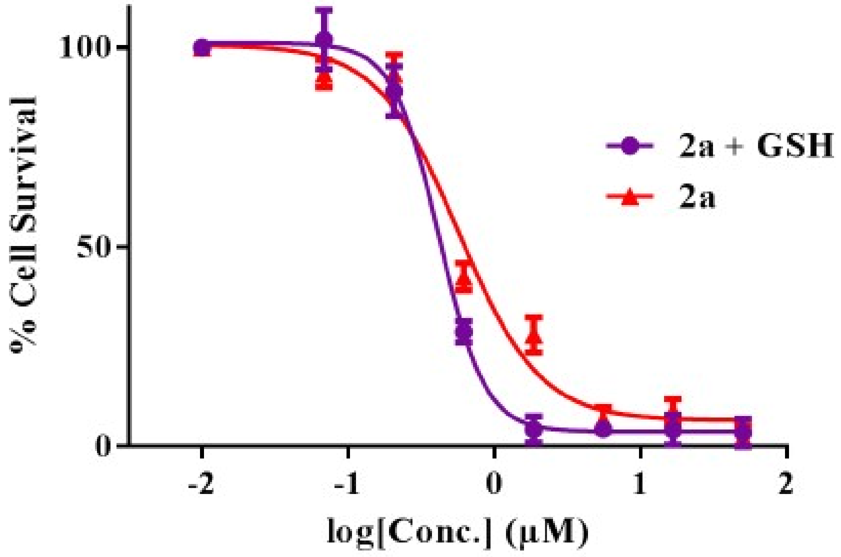

Fig. S110. %Cell survival of **2a** and **2a** + **GSH** over 72 hours, data are plotted as the mean ± s.e.m. (n = 3).

Cell Viability of 1a-1e and 2b-2e:

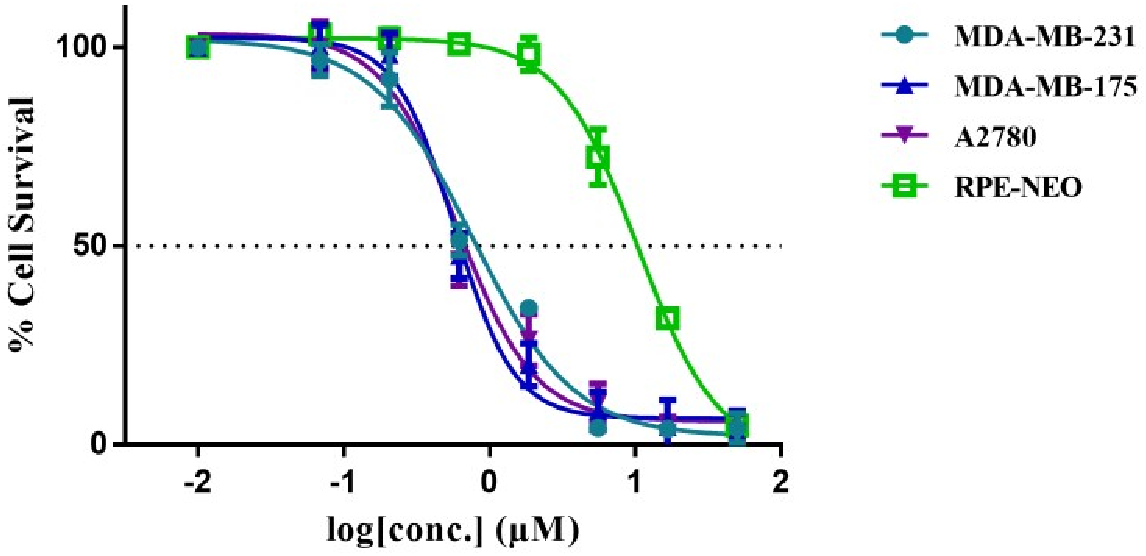

Fig. S111. %Cell survival of **1a**, data are plotted as the mean ± s.e.m. (n = 3).

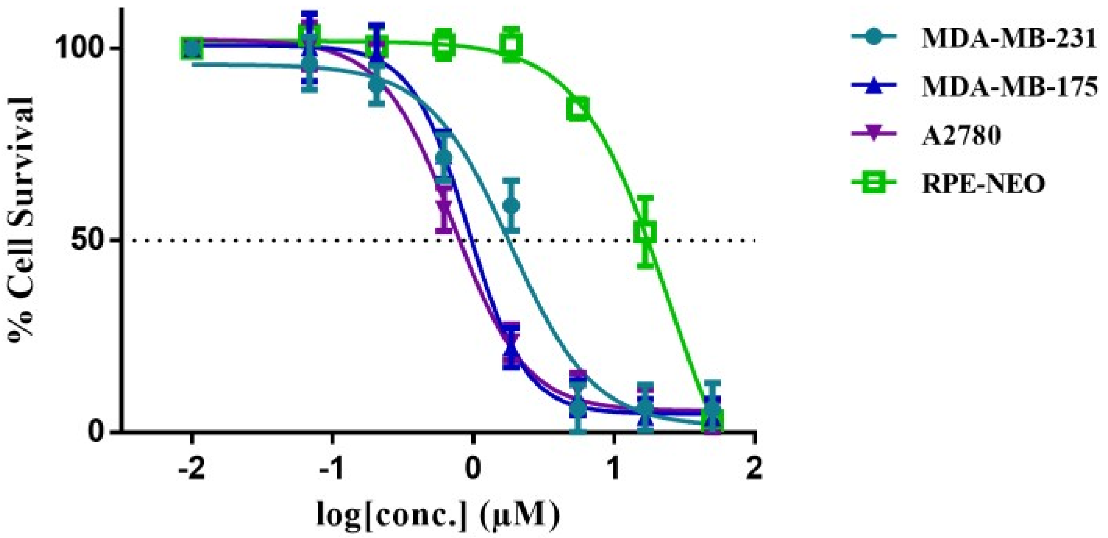

Fig. S112. %Cell survival of **1b**, data are plotted as the mean ± s.e.m. (n = 3).

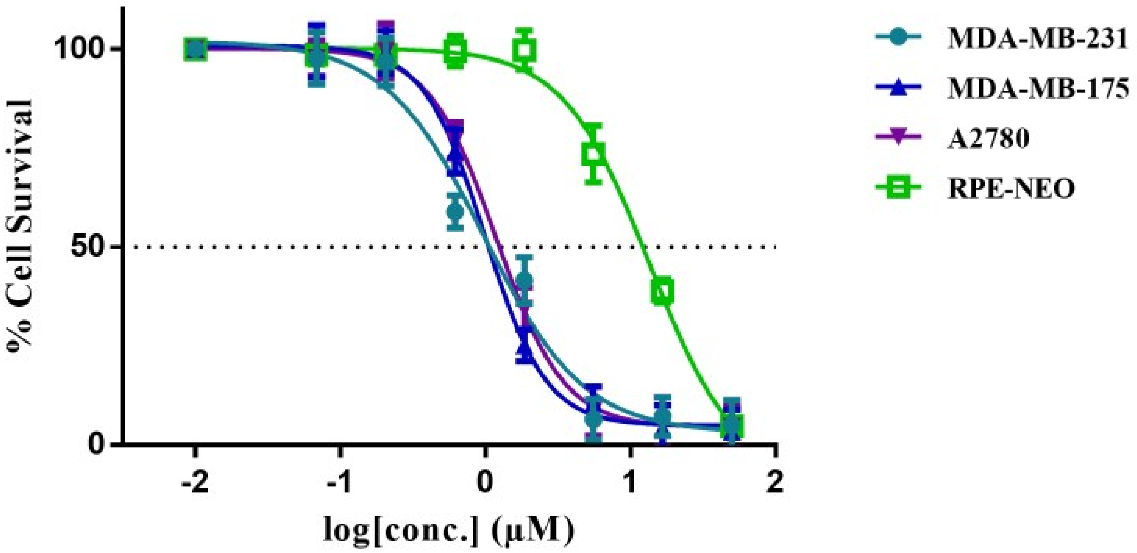

Fig. S113. %Cell survival of **1c**, data are plotted as the mean ± s.e.m. (n = 3).

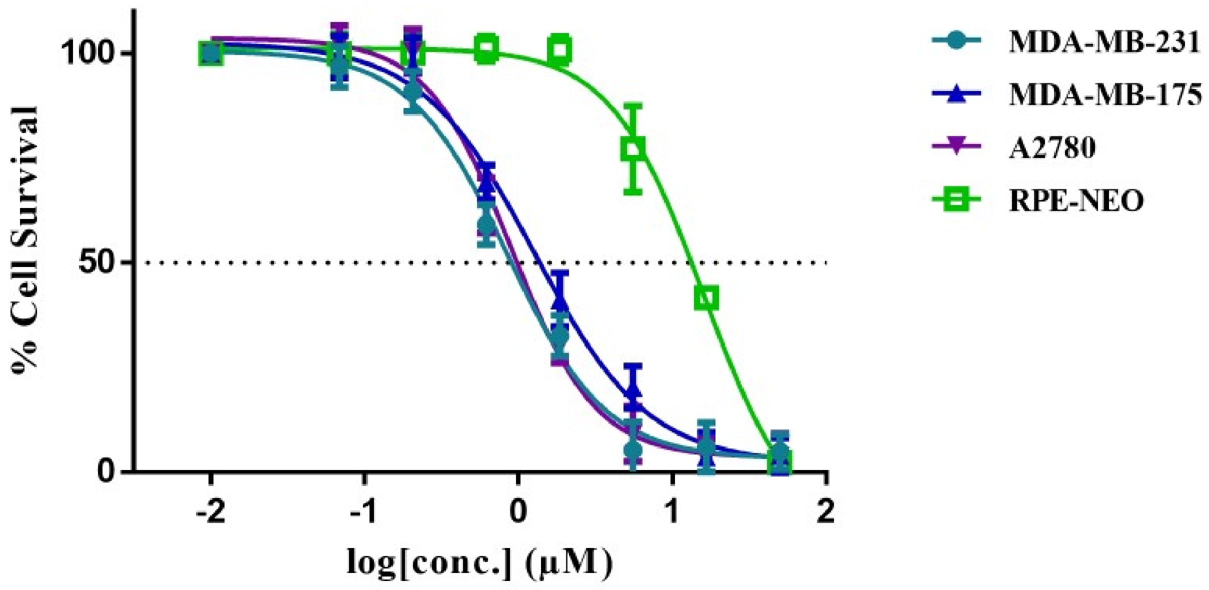

Fig. S114. %Cell survival of **1d**, data are plotted as the mean ± s.e.m. (n = 3).

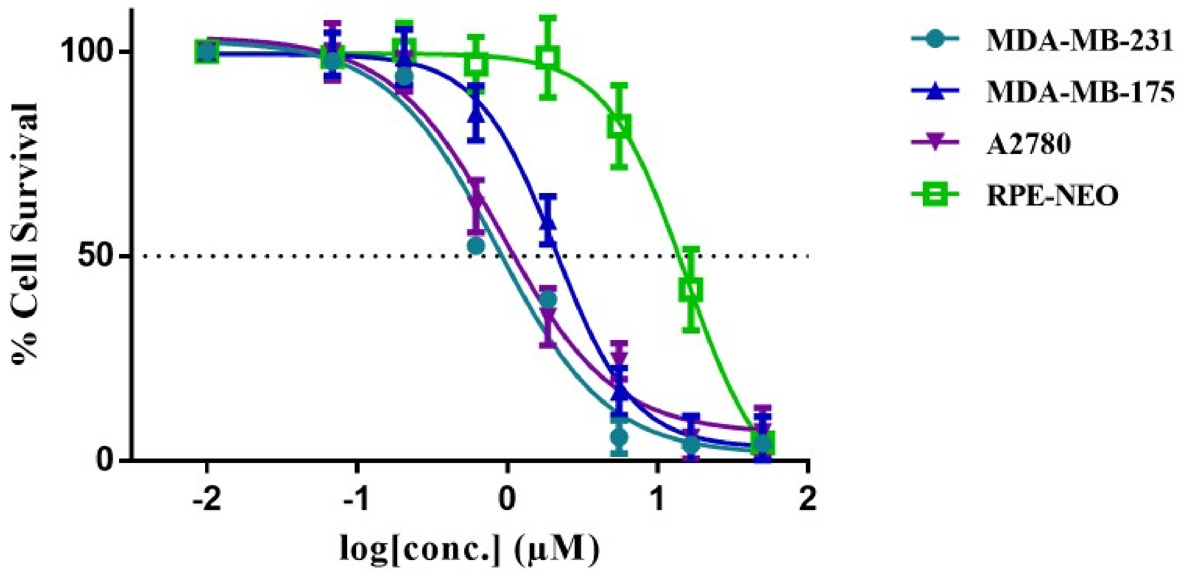

Fig. S115. %Cell survival of **1e**, data are plotted as the mean ± s.e.m. (n = 3).

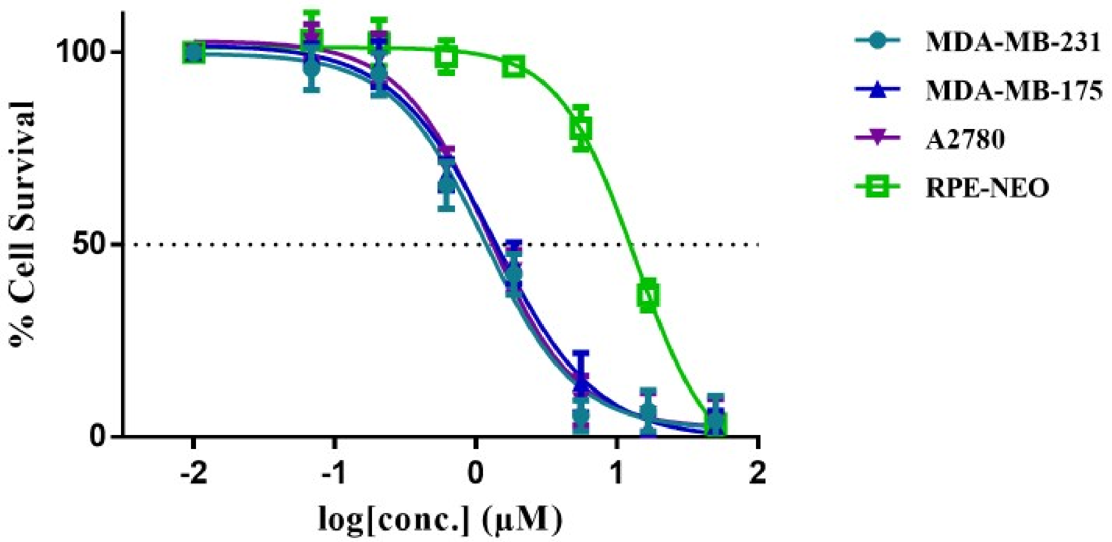

Fig. S116. %Cell survival of **2b**, data are plotted as the mean ± s.e.m. (n = 3).

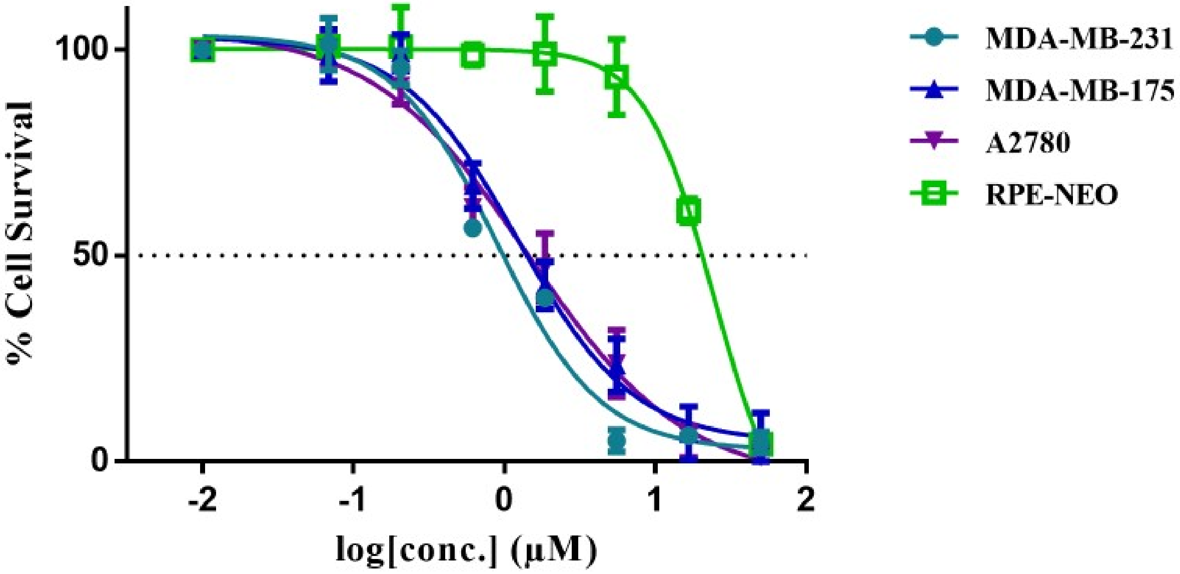

Fig. S117. %Cell survival of **2c**, data are plotted as the mean ± s.e.m. (n = 3).

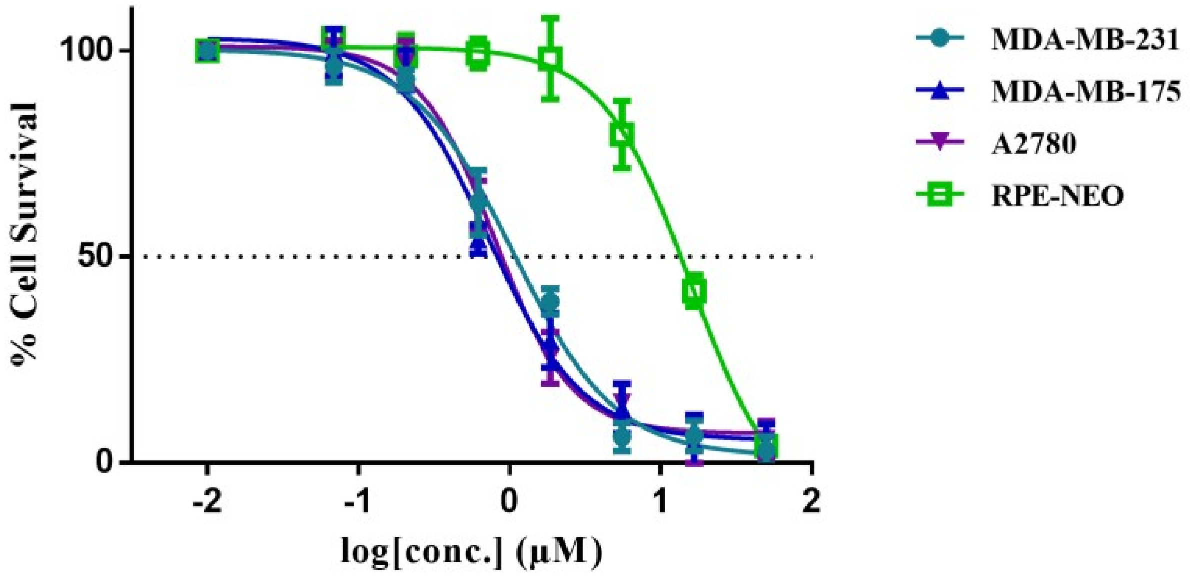

Fig. S118. %Cell survival of **2d**, data are plotted as the mean ± s.e.m. (n = 3).

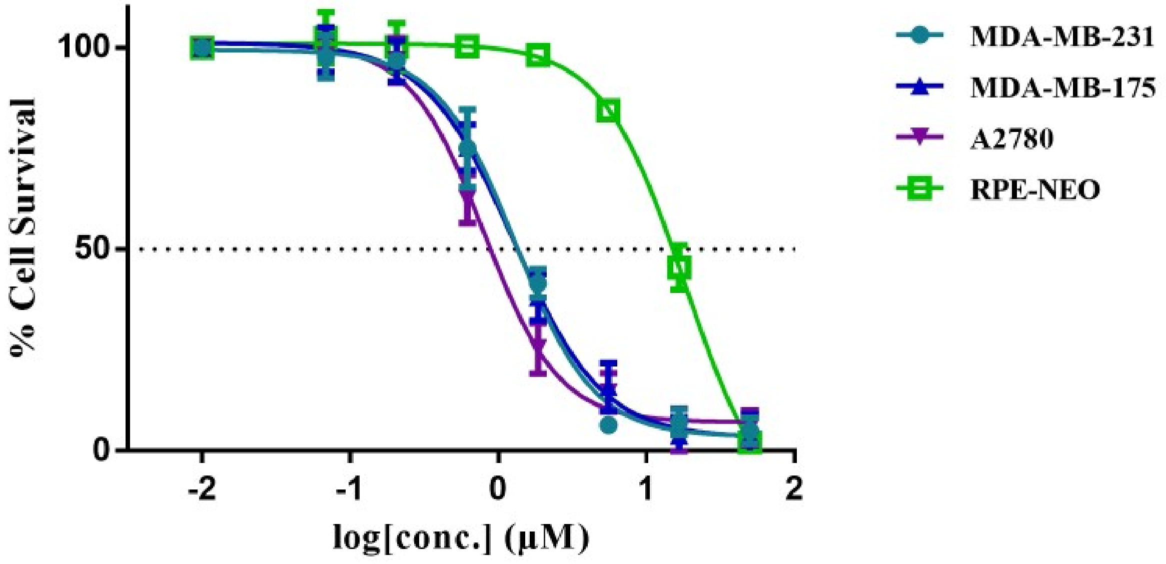

Fig. S119. %Cell survival of **2e**, data are plotted as the mean ± s.e.m. (n = 3).

Cell Viability of 1a -1e and 2a-2b in MRC5:

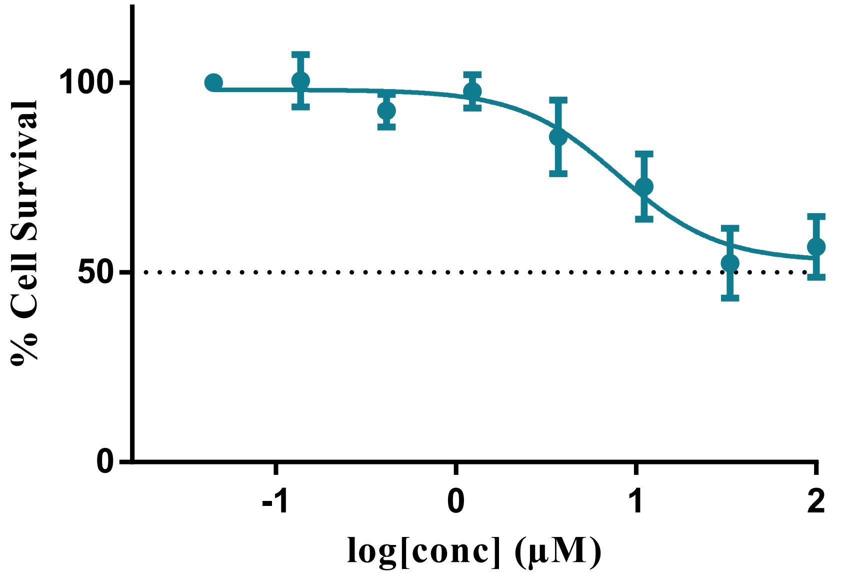

Fig. S120. %Cell survival of **1a**, data are plotted as the mean ± s.e.m. (n = 3).

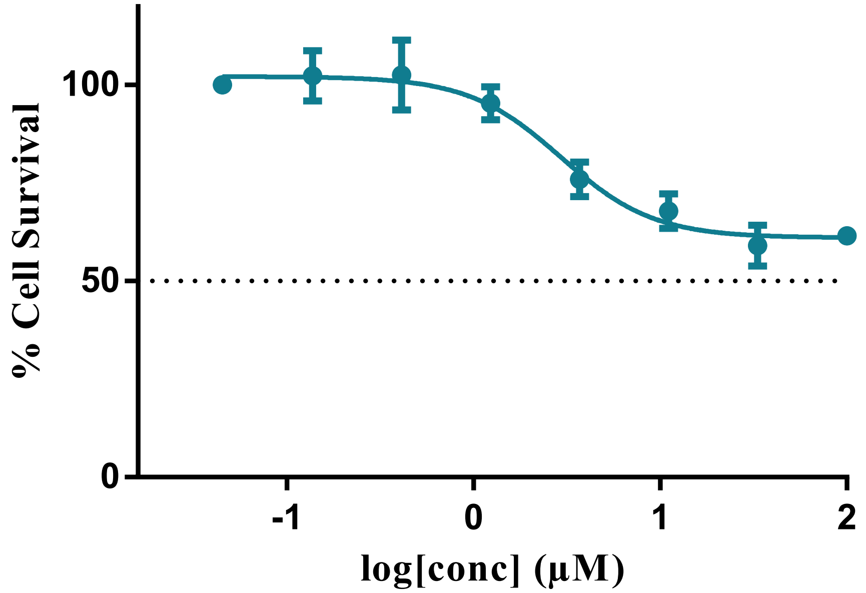

Fig. S121. %Cell survival of **1b**, data are plotted as the mean ± s.e.m. (n = 3).

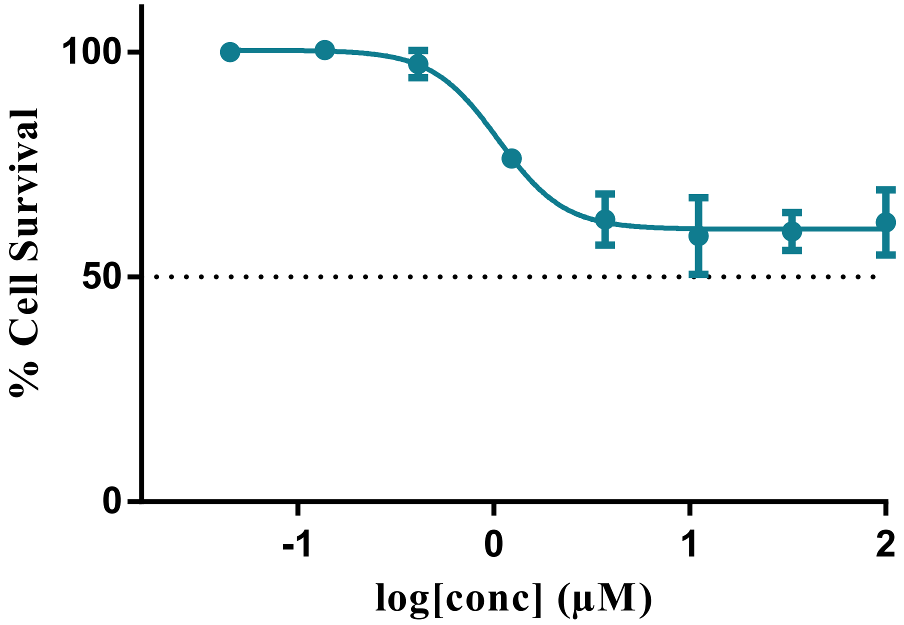

Fig. S122. %Cell survival of **1c**, data are plotted as the mean ± s.e.m. (n = 3).

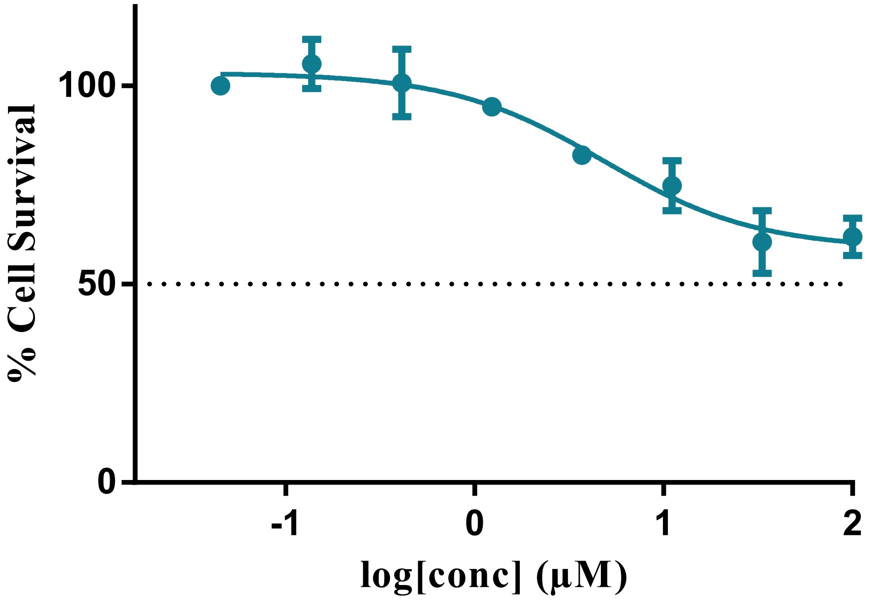

Fig. S123. %Cell survival of **1d**, data are plotted as the mean ± s.e.m. (n = 3).

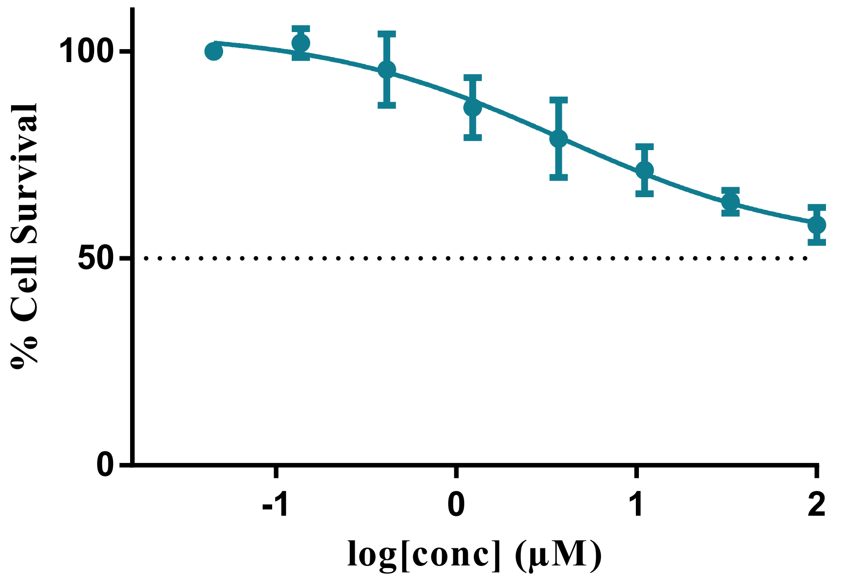

Fig. S124. %Cell survival of **1e**, data are plotted as the mean ± s.e.m. (n = 3).

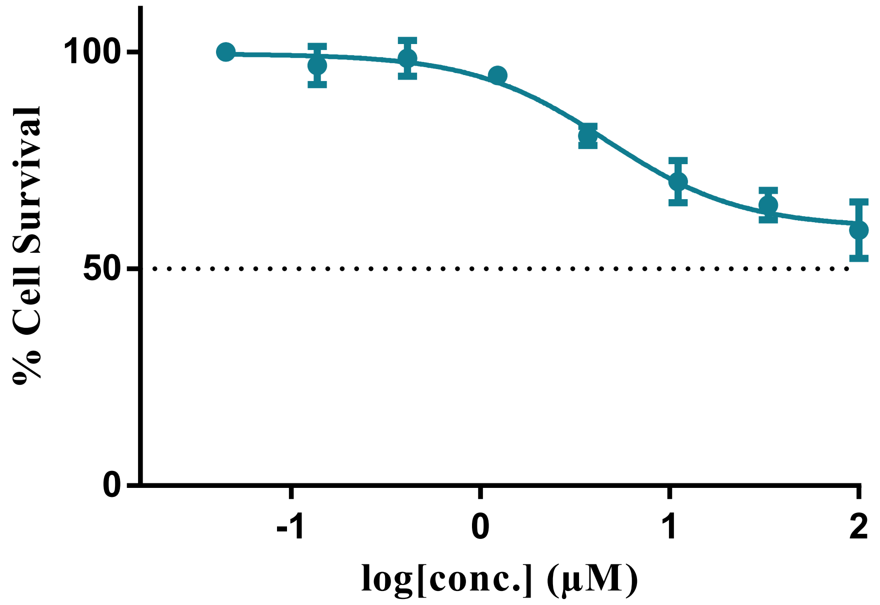

Fig. S125. %Cell survival of **2a**, data are plotted as the mean ± s.e.m. (n = 3).

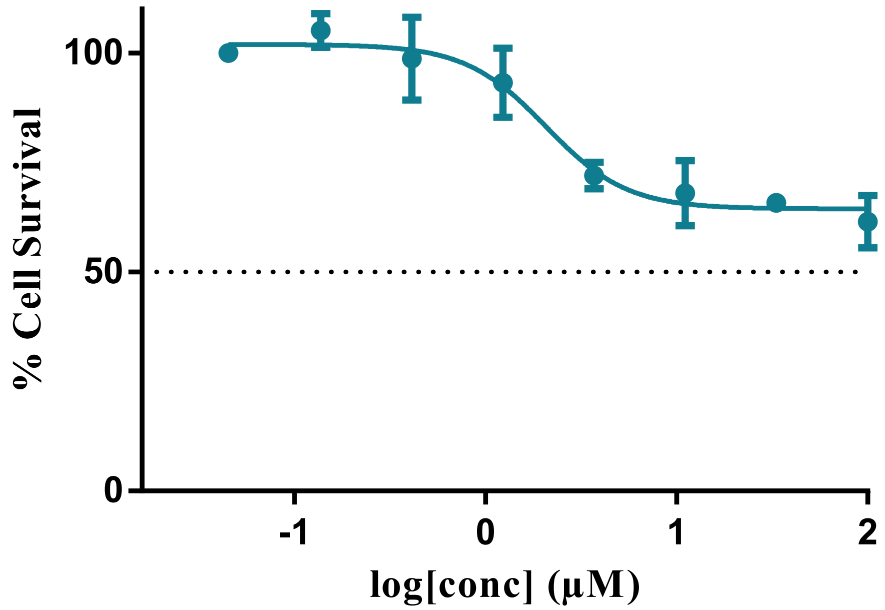

Fig. S126. %Cell survival of **2b**, data are plotted as the mean ± s.e.m. (n = 3).

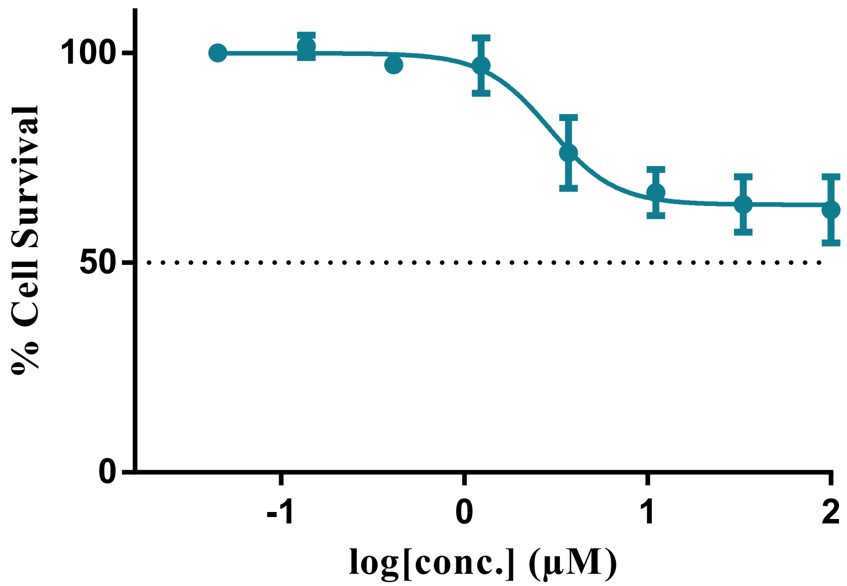

Fig. S127. %Cell survival of **2c**, data are plotted as the mean ± s.e.m. (n = 3).

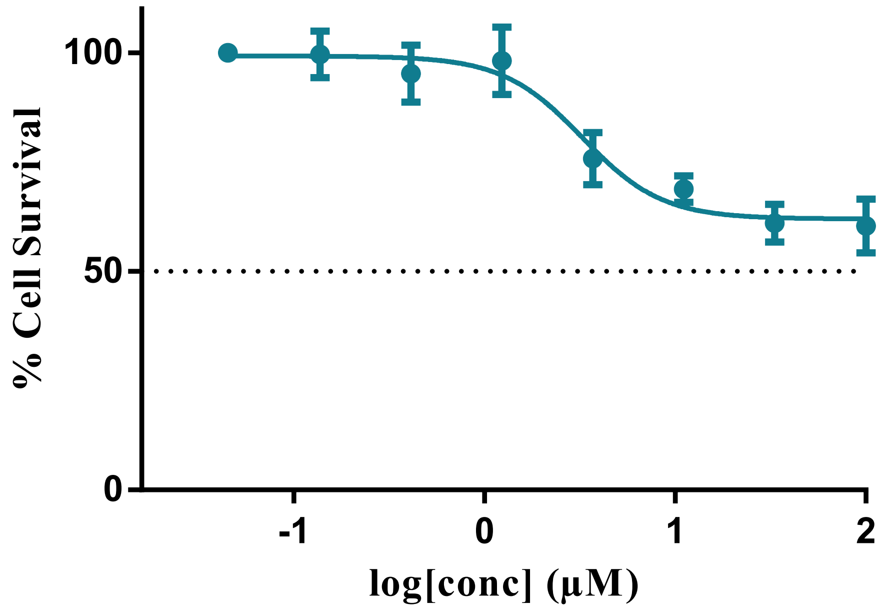

Fig. S128. %Cell survival of **2d**, data are plotted as the mean ± s.e.m. (n = 3).

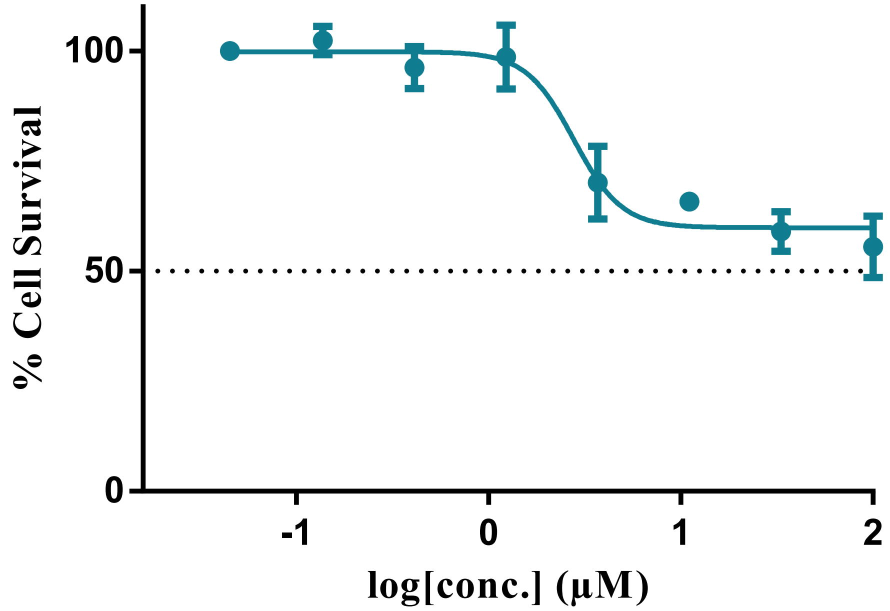

Fig. S129. %Cell survival of **2e**, data are plotted as the mean ± s.e.m. (n = 3).

Mitochondrial Membrane Potential FOV:

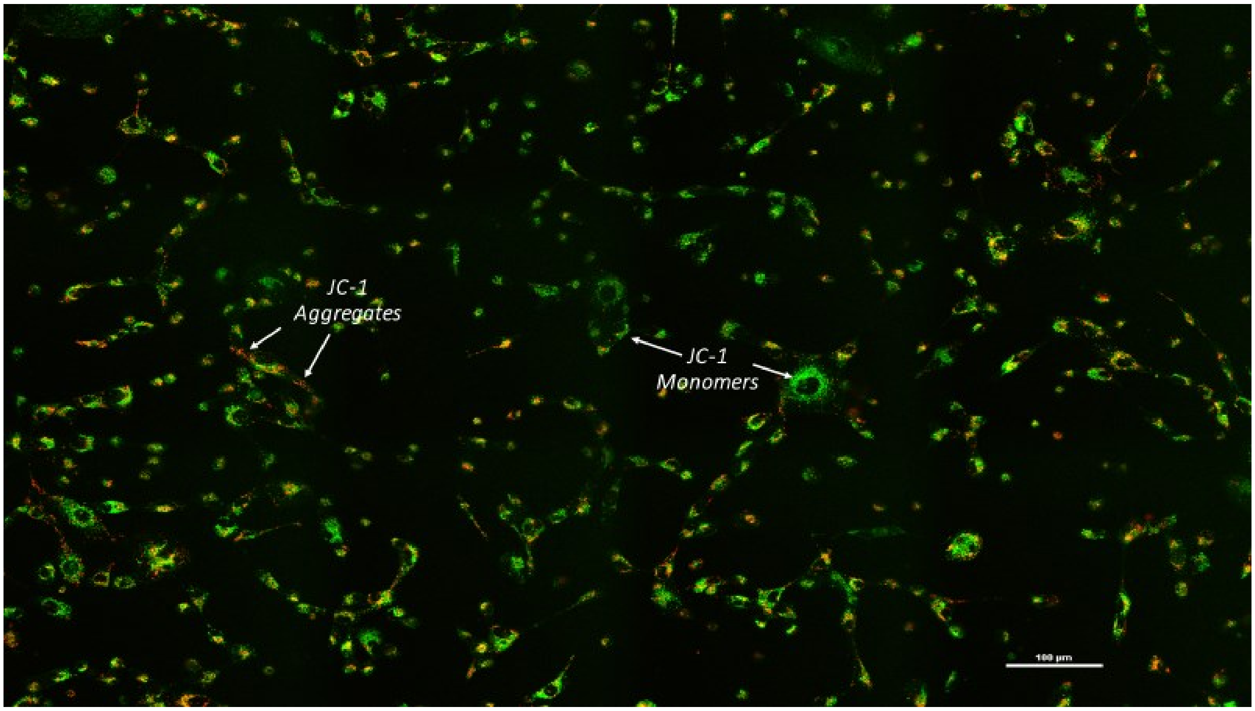

Fig. S130. 2a was treated in MDA-MB-231 at 10 µM for 6 hours. J-aggregates were imaged with (excitation/emission: 510/ 590 nm) and J-monomers with (excitation/emission: 488/525 nm).

Optimization of Seahorse XF96 parameters (MDA-MB-231):

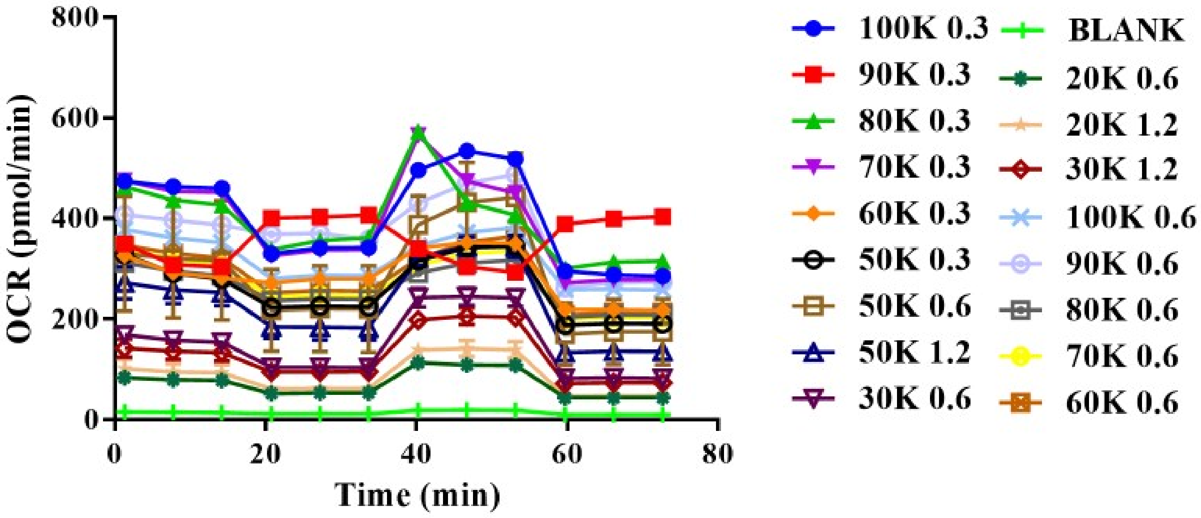

Fig. S131. Optimization of the cell seeding density for MDA-MB-231.

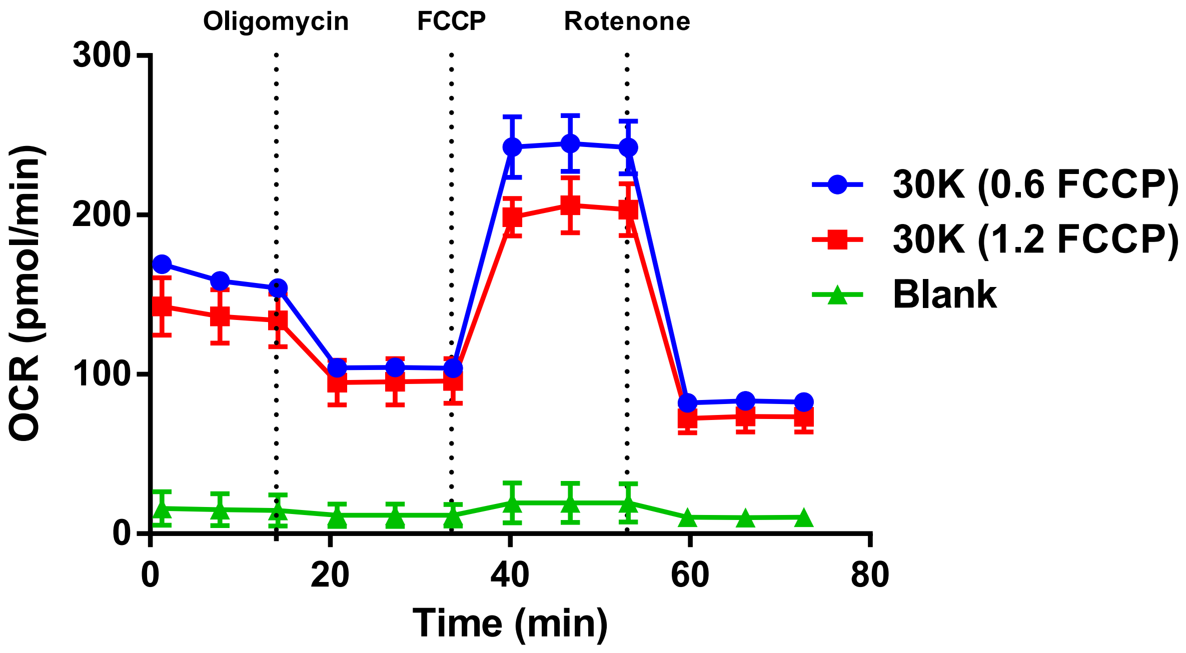

Fig. S132. Optimization of the FCCP injection concentration (μM).

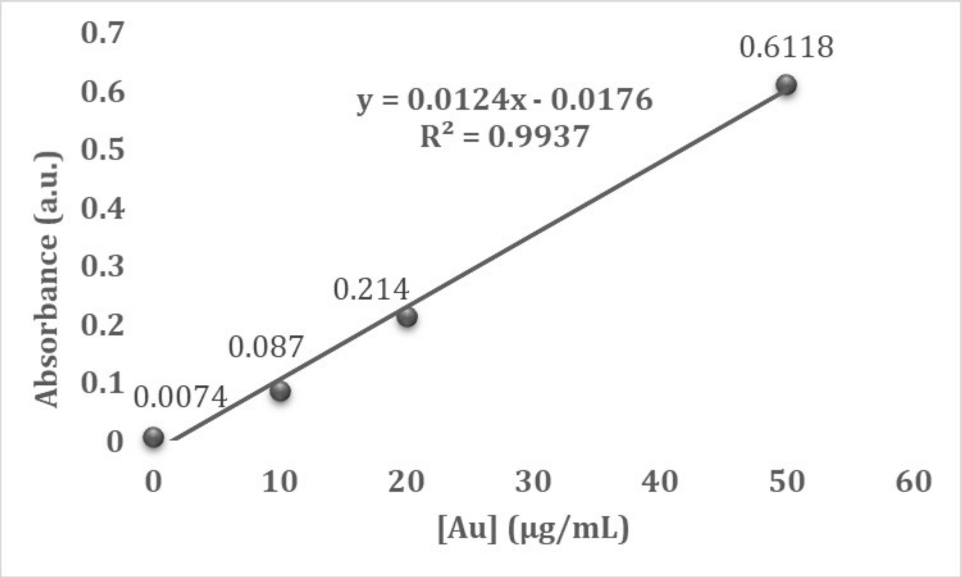

Fig. S133. GF-AAS standard curve with Au concentrations of 0, 10, 20 and 50 μg/mL. Data points are representative of triplicate and plotted as the mean ± s.d.

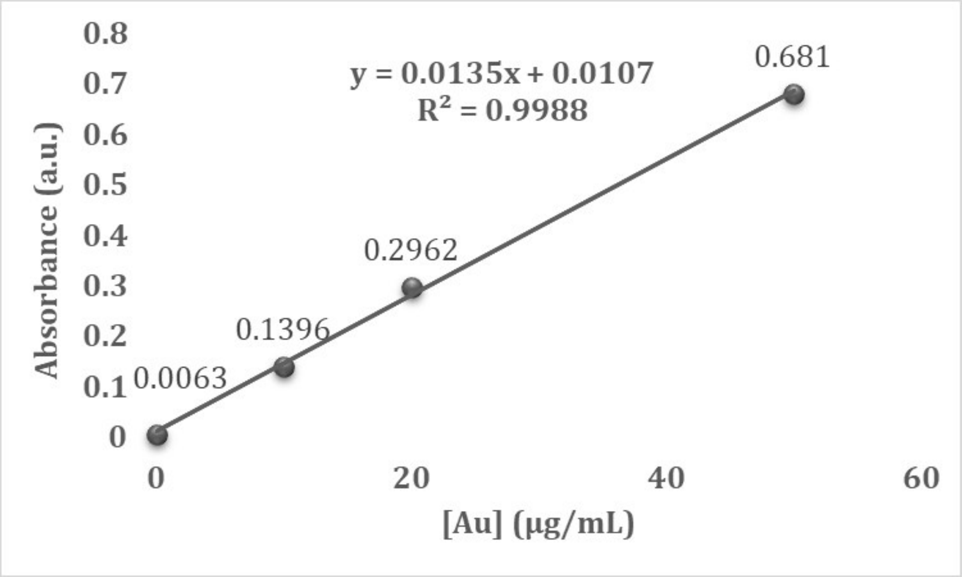

Fig. S134. GF-AAS standard curve with Au concentrations of 0, 10, 20 and 50 μg/mL. Data points are representative of triplicate and plotted as the mean ± s.d.

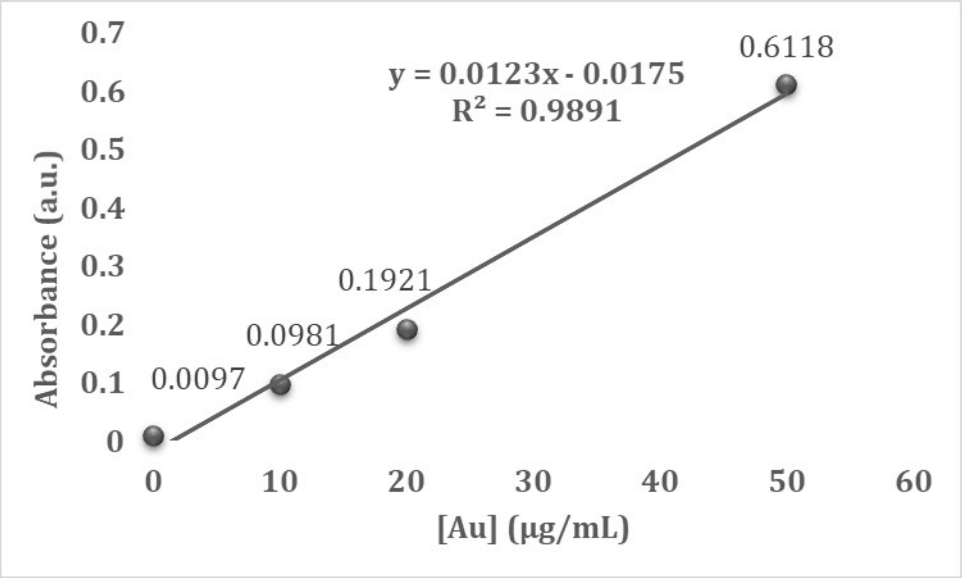

Fig. S135. GF-AAS standard curve with Au concentrations of 0, 10, 20 and 50 μg/mL. Data points are representative of triplicate and plotted as the mean ± s.d.

References

1. Kim, J. H.; Mertens, R. T.; Agarwal, A.; Parkin, S.; Berger, G.; Awuah, S. G., Direct intramolecular carbon(sp2)–nitrogen(sp2) reductive elimination from gold(iii). *Dalton Trans.* **2019,** *48* (18), 6273-6282.
2. Nyamen, L. D.; Rajasekhar Pullabhotla, V. S. R.; Nejo, A. A.; Ndifon, P. T.; Warner, J. H.; Revaprasadu, N., Synthesis of anisotropic PbS nanoparticles using heterocyclic dithiocarbamate complexes. *Dalton Trans.* **2012,** *41* (27), 8297-8302.
3. Bolzati, C.; Cavazza-Ceccato, M.; Agostini, S.; Refosco, F.; Yamamichi, Y.; Tokunaga, S.; Carta, D.; Salvarese, N.; Bernardini, D.; Bandoli, G., Biological in Vitro and in Vivo Studies of a Series of New Asymmetrical Cationic [99mTc(N)(DTC-Ln)(PNP)]+ Complex (DTC-Ln = Alicyclic Dithiocarbamate and PNP = Diphosphinoamine). *Bioconjugate Chem.* **2010,** *21* (5), 928-939.
4. Williams, M. R. M.; Bertrand, B.; Hughes, D. L.; Waller, Z. A. E.; Schmidt, C.; Ott, I.; O'Connell, M.; Searcey, M.; Bochmann, M., Cyclometallated Au(iii) dithiocarbamate complexes: synthesis, anticancer evaluation and mechanistic studies. *Metallomics* **2018,** *10* (11), 1655-1666.
5. Parkin, S.; Hope, H., Macromolecular Cryocrystallography: Cooling, Mounting, Storage and Transportation of Crystals. *J. Appl. Cryst.* **1998,** *31* (6), 945-953.
6. Hope, H., x-ray crystallography - a fast, first-resort analytical tool. *Prog. Inorg. Chem. Vol 41* **1994,** *41*, 1-19.
7. Bruker, *"APEX2" Bruker-AXS.* Madison, WI. USA, 2006.
8. Krause, L.; Herbst-Irmer, R.; Sheldrick, G. M.; Stalke, D., Comparison of silver and molybdenum microfocus X-ray sources for single-crystal structure determination. *J. Appl. Cryst.* **2015,** *48* (Pt 1), 3-10.
9. Sheldrick, G. M., *SADABS, Program for bruker area detector absorption correction.* University of Gottingen, Gottingen, 1997.
10. Sheldrick, G. M., Crystal structure refinement with SHELXL. *Acta Cryst. C Struct. Chem.* **2015,** *71* (Pt 1), 3-8.
11. Sheldrick, G. M., SHELXT - integrated space-group and crystal-structure determination. *Acta Cryst. A. Found. Adv.* **2015,** *71* (Pt 1), 3-8.
12. Sheldrick, G., A short history of SHELX. *Acta Cryst. Sec. A.* **2008,** *64* (1), 112-122.
13. Spek, A. L., Structure validation in chemical crystallography. *Acta Cryst. D. Bio. Cryst.* **2009,** *65* (Pt 2), 148-55.
14. Parkin, S., Expansion of scalar validation criteria to three dimensions: the R tensor. Erratum. *Acta Cryst. A.* **2000,** *56* (Pt 3), 317.
15. van Oosterwijk, J. G.; Li, C.; Yang, X.; Opferman, J. T.; Sherr, C. J., Small mitochondrial Arf (smArf) protein corrects p53-independent developmental defects of *Arf* tumor suppressor-deficient mice. *PNAS***2017,** *114* (28), 7420-7425.
16. Wang, G.; McCain, M. L.; Yang, L.; He, A.; Pasqualini, F. S.; Agarwal, A.; Yuan, H.; Jiang, D.; Zhang, D.; Zangi, L.; Geva, J.; Roberts, A. E.; Ma, Q.; Ding, J.; Chen, J.; Wang, D.-Z.; Li, K.; Wang, J.; Wanders, R. J. A.; Kulik, W.; Vaz, F. M.; Laflamme, M. A.; Murry, C. E.; Chien, K. R.; Kelley, R. I.; Church, G. M.; Parker, K. K.; Pu, W. T., Modeling the mitochondrial cardiomyopathy of Barth syndrome with induced pluripotent stem cell and heart-on-chip technologies. *Nat. Med.* **2014,** *20* (6), 616-623.
